# Halo-perfluoroalkoxylation of *gem*-difluoroalkenes with short-lived alkali metal perfluoroalkoxides in triglyme†‡

**DOI:** 10.1039/d4sc02084g

**Published:** 2024-05-22

**Authors:** Koki Kawai, Yoshimitsu Kato, Taichi Araki, Sota Ikawa, Mai Usui, Naoyuki Hoshiya, Yosuke Kishikawa, Jorge Escorihuela, Norio Shibata

**Affiliations:** a Department of Nanopharmaceutical Sciences, Nagoya Institute of Technology Gokiso, Showa-ku Nagoya 466-8555 Japan nozshiba@nitech.ac.jp; b Department of Life Science and Applied Chemistry, Nagoya Institute of Technology Gokiso, Showa-ku Nagoya 466-8555 Japan; c Technology Innovation Center, DAIKIN Industries, Ltd 1-1 Nishi-Hitotsuya, Settsu Osaka 566-8585 Japan; d Departamento de Química Orgánica, Universitat de València Avda. Vicente Andrés Estellés S/N, Burjassot 46100 Valencia Spain

## Abstract

Alkali metal alkoxides play a pivotal role in nucleophilic alkoxylation reactions, offering pathways for the synthesis of ethers, including the increasingly sought-after trifluoromethyl ethers. However, the synthesis of long-chain perfluoroalkyl ethers remains a substantial challenge in this field. Through the innovative use of triglyme to encapsulate potassium ions, we enhanced the stability of short-lived, longer-chain perfluoroalkoxy anions, thereby facilitating efficient nucleophilic perfluoroalkoxylation reactions. This method provides a new precedent for the halo-perfluoroalkoxylation of *gem*-difluoroalkenes and offers a versatile tool for the design of perfluoroalkyl ethers, including those containing complex moieties of heterocycles and drug molecules. We also demonstrated the utility of the resulting halo-perfluoroalkoxyl adducts through various chemical transformations to valuable diverse perfluoroalkyl ethers.

## Abstract 2

The synthesis of long-chain perfluoroalkyl ethers remains a challenge. The stabilization of longer-chain perfluoroalkoxides with triglyme-encapsulated potassium ions enabled the synthesis of bis(α,α-difluoro)ethers with *gem*-difluoroalkenes.

## Graphical abstract

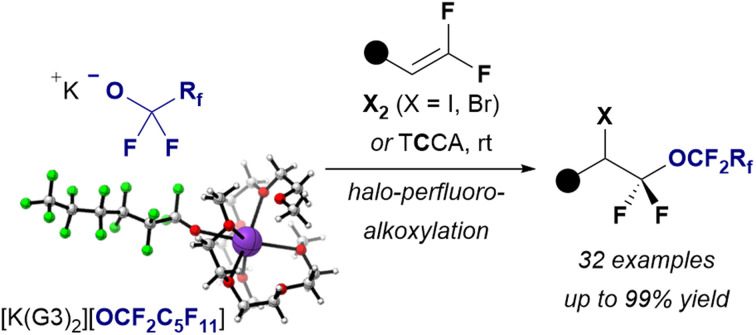

## Introduction

**Fig. 1:**
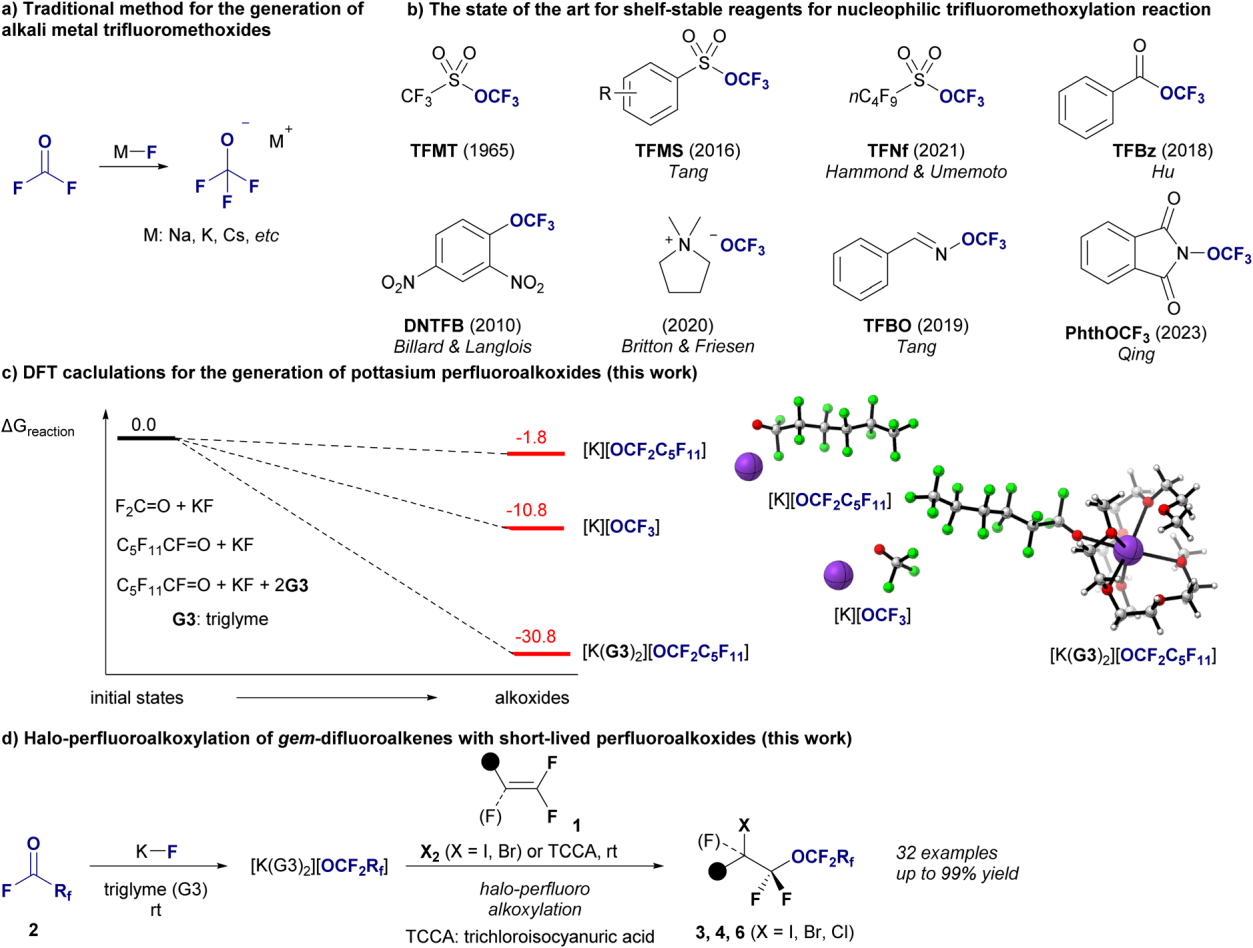
Background and strategy of nucleophilic perfluoroalkoxylations. (a) Traditional method for the preparation of alkali metal trifluoromethoxides. (b) Shelf-stable reagents for nucleophilic trifluoromethoxylation. (c) DFT-calculated reaction Gibbs free energy B3LYP-D3/6-311+G(d,p) in ether (SMD), in kcal mol^−1^ for the reactions of KF with F_2_C=O (−10.8 kcal mol^−1^), C_5_F_11_CF=O (−1.8 kcal mol^−1^) and C_5_F_11_CF=O with triglyme (−30.8 kcal mol^−1^) (this work). (d) Halo-perfluoroalkoxylation of olefins (this work).

Alkali metal alkoxides are recognized as key reagents in organic synthesis, especially for nucleophilic alkoxylation reactions that establish carbon–oxygen (C–O) bonds and produce ethers.^1^ Recent breakthroughs have thrusted this area into new territories, especially in the synthesis of trifluoromethyl ethers (R-OCF_3_),^2^ promising exciting developments in agrochemicals and pharmaceuticals.^3^ Traditionally, the production of alkali metal trifluoromethoxides [M][OCF_3_] requires fluorophosgene (carbonyl fluoride, F_2_C=O) and alkali metal fluorides (M–F, Fig. 1a),^4^ and the recent introduction of safer shelf-stable reagents for nucleophilic trifluoromethylation has advanced significantly (Fig. 1b).^5^ Perfluoroalkyl ethers are prized for their high lipophilicity, improved metabolic stability, and unparalleled thermal and chemical resilience, and they can be used in various industries.^6–8^ Most of the unique properties are due to the ether oxygens inserted between the perfluorinated carbon backbones.^7*b*^ For example, compared to perfluoroalkyl carboxylic acids, perfluoropolyether carboxylic acids allow easy formation of non-covalent hydrogen bonds with water, resulting in increased hydrophilicity.^8*a*^ The presence of ether linkages in perfluoroalkyl ether carboxylic acids results in increased bond dissociation energies for the adjacent carbon-fluorine bonds, which affects their reactivity and degradation results.^8*b*^ This structural feature generally promotes more effective degradation under reductive conditions than non-oxygenated variants by facilitating the cleavage of carbon–oxygen bonds and enhancing defluorination. There are also reports that perfluoroalkylether sulfonates degrade more effectively than perfluoroalkyl sulfonates in subcritical water in the presence of oxygen^8*c*^ due to their ether linkages. In addition, perfluoroalkyl compounds with long perfluorinated chains consisting of more than 10 perfluorinated carbons exhibit a crystalline nature,^9^ which would also be an additional advantage of perfluoroalkyl ethers.^10^ Despite rapid progress in nucleophilic trifluoromethoxylation, the synthesis of longer perfluoroalkyl ethers remains a significant challenge,^11,12^ particularly for ethers with perfluoroalkyl moieties on both sides, represented by RCF_2_–O–CF_2_R′.^12^ To understand the difficulty of nucleophilic longer-chain perfluoroalkoxylation reactions, our investigation began with density functional theory (DFT) calculations to assess the feasibility of generating perfluoroalkoxides from their precursors, represented by two critical reactions: the traditional generation of [K][OCF_3_] from O=CF_2_ and potassium fluoride (KF) and the formation of potassium perfluorohexanolate [K][OCF_2_C_5_F_11_] from perfluorohexanoyl fluoride (O=CF(C_5_F_11_)) and KF (Fig. 1c). Our results reveal a stark contrast in the reaction Gibbs free energy (Δ*G*_R_) between these processes (see ESI‡ for details). The formation of [K][OCF_3_] had a Δ*G*_R_ of −10.9 kcal mol^−1^, indicating a favorable reaction pathway. In contrast, the generation of [K][OCF_2_C_5_F_11_] had a Δ*G*_R_ of −1.8 kcal mol^−1^, suggesting a less favorable process. To overcome this discrepancy and enhance the synthetic feasibility of long-chain perfluoroalkyl ethers, we introduced a novel method that involves encapsulating K^+^ ions with two molecules of triglyme (G3). This approach significantly increases the stability of the OCF_2_C_5_F_11_ anion, facilitating the efficient synthesis of this complex molecule, [K(G3)_2_][OCF_2_C_5_F_11_], with a Δ*G*_R_ of −33.9 kcal mol^−1^, indicating a highly favorable reaction process. This result not only deepens our understanding of the stability of alkali metal perfluoroalkoxides but also realizes the halo-perfluoroalkoxylation of *gem*-difluoroalkenes 1 using [K(G3)_2_][OCF_2_Rf] generated *in situ* from perfluoroalkanoyl fluorides 2 (O=CFRf) with KF, yielding valuable difluoroalkyl perfluoroalkyl ethers 3 (X = I), 4 (X = Br) or 6 (X = Cl) with chain lengths from C_3_ to C_6_ (Fig. 1d). This halo-perfluoroalkoxylation reaction was achieved with regioselectivity, and various perfluoroalkyl ethers with functional groups relevant to materials and medicines were obtained in high yields. Finally, we explored the various chemical transformations of the resulting halo-perfluoroalkoxy products to demonstrate their potential as building blocks.

## Results and discussion

The halo-perfluoroalkoxylation reaction was optimized using *gem*-difluoroalkene 1a and alkali metal perfluoroalkoxide generated *in situ* from undecafluorohexanoyl fluoride 2a and inorganic fluoride as model substrates in the presence of halogen sources (Table 1). Glymes were used as the solvent to stabilize the labile perfluoroalkoxide by encapsulating metal cations, based on our previous research on trifluoromethylation reactions involving the labile trifluoromethyl anion.^13^ Initially, 2a was treated with sodium fluoride (NaF), KF, or cesium fluoride (CsF) in triglyme for 15 min at room temperature to generate metal perfluoroalkoxides. After fluoroalkene 1a was added to the perfluoroalkoxide solution, I_2_ was added to the reaction mixture. NaF treatment did not yield the desired product (entry 1), KF treatment produced 3aa in 38% yield (entry 2), and CsF treatment resulted in 25% yield (entry 3). A lower yield (4%) was obtained when *N*-iodosuccinimide (NIS) was used instead of I_2_ (entry 4). Switching the solvent to MeCN, DMF, Et_2_O, or THF using KF did not increase the yield (entries 5–8), even in the presence of 18-crown-6-ether (18-C-6, entry 9). Subsequently, we varied the equivalents of acyl fluorides 2a, KF, and I_2_. A yield of 38% was obtained when using 2.0 equiv. of I_2_ (entry 10). Using triglyme/THF solved (entry 11) and increasing the number of equivalents of 2a and KF to 2.0 equiv. (entry 12) led to decrease in yields. Remarkably, employing 2.0 equivalents of 2a, KF, and I_2_ substantially improved the yield to 70% (entry 13), with further enhancement to an outstanding 89% yield achieved by increasing the equivalents to 3.0 (entry 14). Encouraged by the success of iodo-perfluoroalkoxylation and subsequent bromo-perfluoroalkoxylation of 1a with 2a using bromine in the triglyme, we noted the significant impact of reactant quantities (entries 15–17). Notably, a high yield of the desired compound 4aa (88%) was obtained by employing 3.0 equivalents of reagents (entry 17). The addition of reagents is critical for successful transformation. When the generated alkoxide and Br_2_ were first mixed, followed by the addition of alkene 1a, the yield of the desired fluoroalkyl ether 4aa decreased to 56%, and 1a was recovered in 36% yield (entry 18).

**Table 1:** Optimization of reaction conditions for halo-perfluoroalkoxylation of *gem*-difluoroalkene^a^

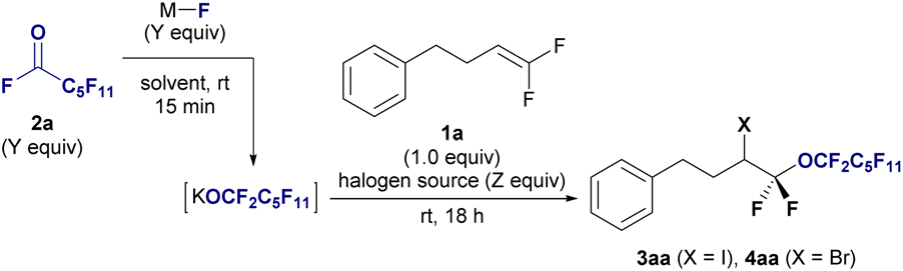						
Entry	2a (*Y* equiv.)	MF	Halogen source (*Z* equiv.)	Solvent (0.1 M)	Yield^b^3aa (%)	Yield^b^4aa (%)
1	1.0	NaF	I_2_ (1.0)	Triglyme	0	
2	1.0	KF	I_2_ (1.0)	Triglyme	38	
3	1.0	CsF	I_2_ (1.0)	Triglyme	25	
4	1.0	KF	NIS (1.0)	Triglyme	4	
5	1.0	KF	I_2_ (1.0)	MeCN	18	
6	1.0	KF	I_2_ (1.0)	DMF	9	
7	1.0	KF	I_2_ (1.0)	Et_2_O	0	
8	1.0	KF	I_2_ (1.0)	THF	0	
9^c^	1.0	KF	I_2_ (1.0)	THF	7	
10	1.0	KF	I_2_ (2.0)	Triglyme	38	
11	1.0	KF	I_2_ (2.0)	Triglyme/THF (1/2)	22	
12	2.0	KF	I_2_ (1.0)	Triglyme	31	
13	2.0	KF	I_2_ (2.0)	Triglyme	70	
14	3.0	KF	I_2_ (3.0)	Triglyme	89	
15	1.0	KF	Br_2_ (1.0)	Triglyme		48
16	2.0	KF	Br_2_ (2.0)	Triglyme		70
17^d^	3.0	KF	Br_2_ (3.0)	Triglyme		88^e^
18^f^	3.0	KF	Br_2_ (3.0)	Triglyme		56

a Reaction conditions: 1a (0.1 mmol), 2a, MF, halogen source, solvent (1.0 mL), room temperature for 18 h.

b ^19^F NMR yields were determined using C_6_F_6_ as an internal standard.

c 18-C-6 (1.0 equiv.) was added.

d 1a (0.3 mmol) was used for 6 h

e Isolated yield.

f 1a was added after stirring Br_2_ with the perfluoroalkoxide generated for 15 min.

**Fig. 2:**
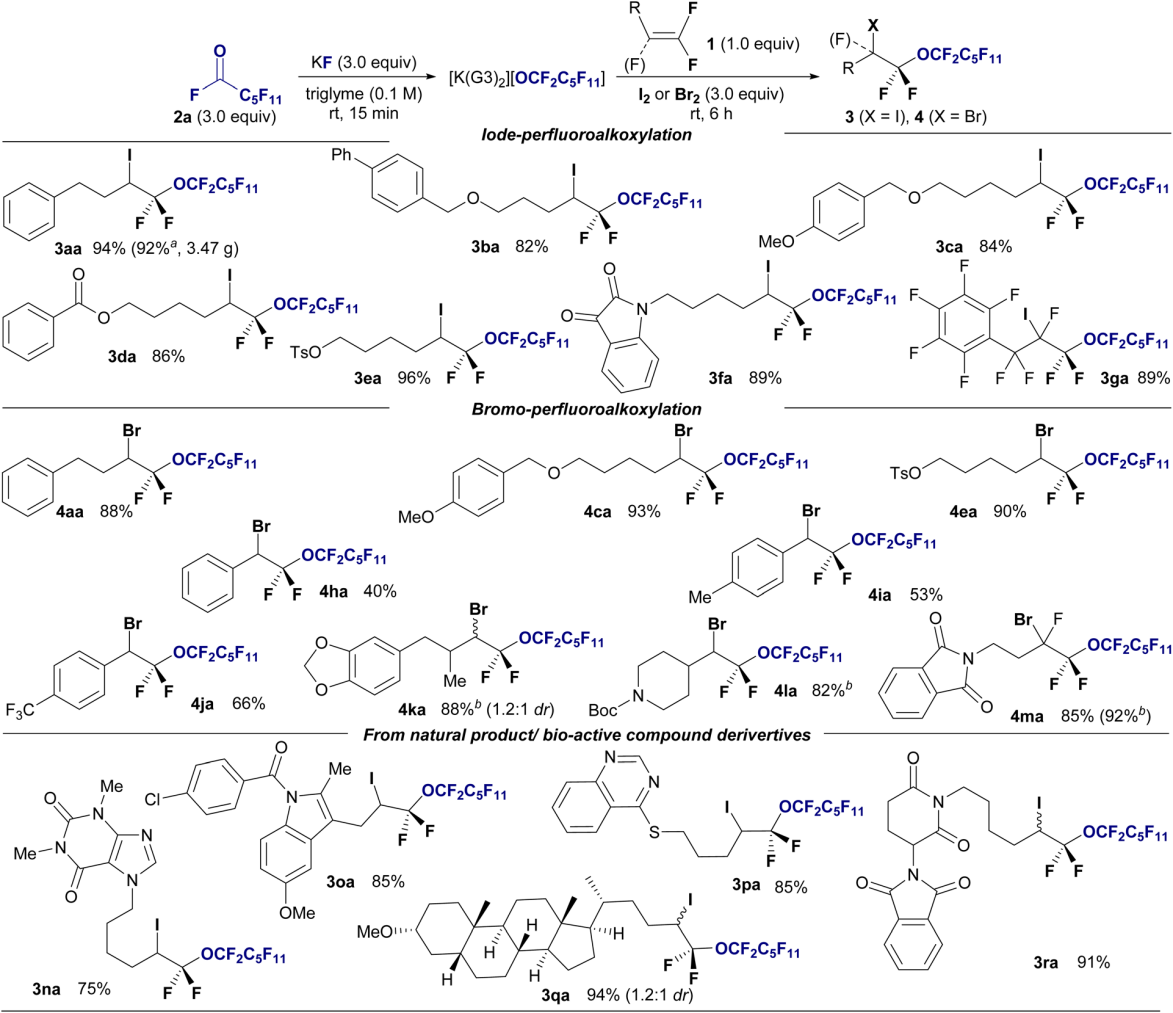
Substrate scope of the halo-perfluoroalkoxylation of *gem*-difluoroalkenes 1 using undecafluorohexanoyl fluoride 2a. Isolated yields are shown. Reaction conditions: 1 (0.3 mmol), 2a (0.9 mmol), KF (0.9 mmol), I_2_ or Br_2_ (0.9 mmol) in triglyme (3.0 mL), stirring under atmosphere of N_2_ at room temperature. (a) Gram scale reaction. 1 (6.0 mmol, 1.01 g) was used. (b) 1 (0.3 mmol), 2a (1.5 mmol), KF (1.5 mmol), Br_2_ (1.5 mmol) in triglyme (6.0 mL).

Next, we explored the substrate scope of haloperfluoroalkoxylation under optimal reaction conditions (Fig. 2). To demonstrate the functional tolerance of this method, various *gem*-difluoroalkenes with diverse reactive functionalities, including benzyl ether (1b), ether (1c), ester (1d), tosylate (1e), ketone, and amide groups (1f), were selected as substrates for iodo-perfluoro-hexyloxylation. All the reactions proceeded smoothly, furnishing desired α-perfluorohexyl α,α-difluoroalkyl ethers 3 in high yields (82–96%) and regioselectivity, irrespective of the functional group. Notably, chemoselectivity was observed in the iodo-perfluoro-hexyloxylation of perfluoroallylbenzene (1g). The desired perfluorinated ether 3ga was obtained in 81% yield without S_N_Ar reactions at the perfluorophenyl moiety. A gram-scale reaction was also conducted using perfluoroalkoxide on 6.0 mmol (1.01 g) of 1a under optimized conditions, resulting in the isolation of iodoperfluoroalkyl ether 3aa in 92% yield (Fig. 2, 3aa: 3.47 g isolated).

We expanded the substrate scope of bromoperfluoroalkoxylation using Br_2_. *Gem*-difluoroalkenes bearing benzyloxy and methoxy moieties (1c) and tosylate (1e) reacted effectively with potassium perfluorohexyloxide 2a, which was generated *in situ* in the presence of Br_2_, providing the corresponding perfluorohexyloxyl difluoroalkyl ethers in excellent yields (4aa, 88%; 4ca, 93%; 4ea, 90%). *Gem*-difluorostyrene derivatives (1h–1j) with nonsubstituted (H), electron-donating (Me), and electron-withdrawing (CF_3_) groups also interacted with perfluoroalkoxides under bromination conditions, affording the corresponding bromoperfluoroalkyl ethers in moderate to good yields (4ha: 40%, 4ia: 53%, 4ja: 66%). The bromo-perfluoroalkylation of *gem*-difluoroalkenes with a secondary alkyl *γ*-position furnished the corresponding ethers (4ka: 88% (1.2 : 1 dr), 4la: 82%). Significantly, the trifluorinated alkene 1m efficiently reacted with 2a, KF, and bromine, yielding bis(α,α-difluoro)ether in a high yield (4ma: 92%). Subsequently, reactions involving various *gem*-difluoroalkenes bearing natural products or biologically relevant moieties were explored. *Gem*-difluoroalkenes 1, containing theophylline (1n), indomethacin (1o), an insecticidal agent (1p), lithocholic acid (1q), and thalidomide (1r), underwent iodoperfluoroalkoxylation using 1a, KF, and I_2_ in the triglyme, resulting in the synthesis of drug-conjugated perfluoroalkyl diethyl ether in high yields and with excellent regioselectivity (3na: 75%; 3oa: 85%; 3pa: 85%; 3qa: 75%; 3ra: 91%). Biologically attractive molecules with perfluoroalkyl chains are expected to be used as decoy molecules in drug discovery.^14^

**Fig. 3:**
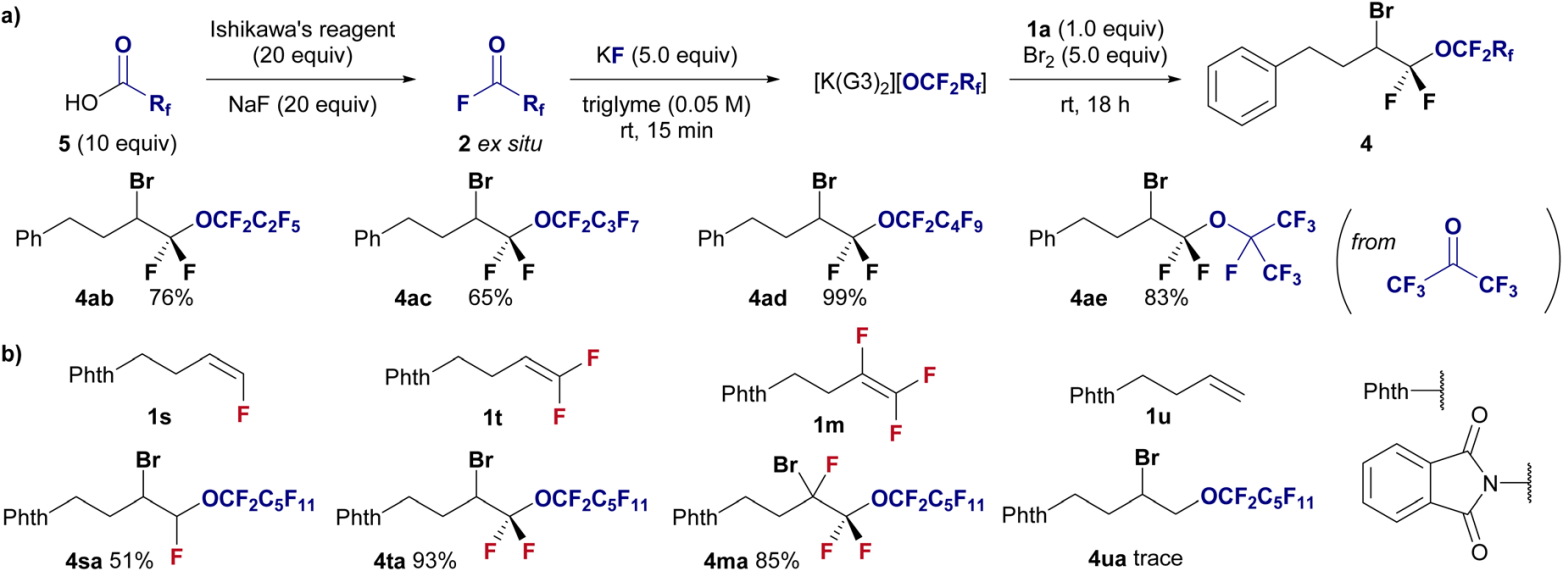
(a) Substrate scope of the halo-perfluoroalkoxylation of *gem*-difluoroalkene 1a using various perfluoroacylfluorides 2. Isolated yields are shown. Reaction conditions: 1a (0.3 mmol), 2 (excess), KF (1.5 mmol), Br_2_ (1.5 mmol) in triglyme (6.0 mL), stirring under atmosphere of N_2_ at room temperature. (b) Investigation of fluoroalkene selective reaction using undecafluorohexanoyl fluoride 2a under the standard conditions in Fig. 2.

This methodology was applied to halo-perfluoroalkoxylation reactions with shorter perfluoroalkoxy moieties ranging from C_3_ to C_5_ to demonstrate a modular approach (Fig. 3a). Perfluoroacyl fluorides (2b–2d) were generated *ex situ* from their corresponding perfluorocarboxylic acids (5) *via* deoxyfluorination using Ishikawa's reagent and NaF. The perfluoropropoxylation, perfluorobutyloxylation, and perfluoropentoxylation of fluoroolefines proceeded smoothly, affording the corresponding perfluoroalkoxyethers in good to high yields (4ab, 76%; 4ac, 65%; 4ad, 99%). Furthermore, perfluoro-isopropoxylation was performed using hexafluoroacetone and KF in triglyme with difluoroalkene 1a in the presence of Br_2_ to afford the desired branched perfluorinated alkyl ether 4ae in 83% yield.

We further investigated the effect of fluorine on the reactivity of alkenes in halo-perfluoroalkoxylation reactions under standard conditions (Fig. 3b). Notably, the effect of fluorine substitution is obvious, and mono- (1s), di- (1t), and tri-fluorinated (1m) alkenes are well accepted as substrates (4sa, 51%; 4ta, 93%; 4ma, 85%), whereas the nonfluorinated alkene 1u remains unreactive (4ua, trace). This highly fluoroalkene-selective transformation enhances the advantages of this methodology.

**Fig. 4:**
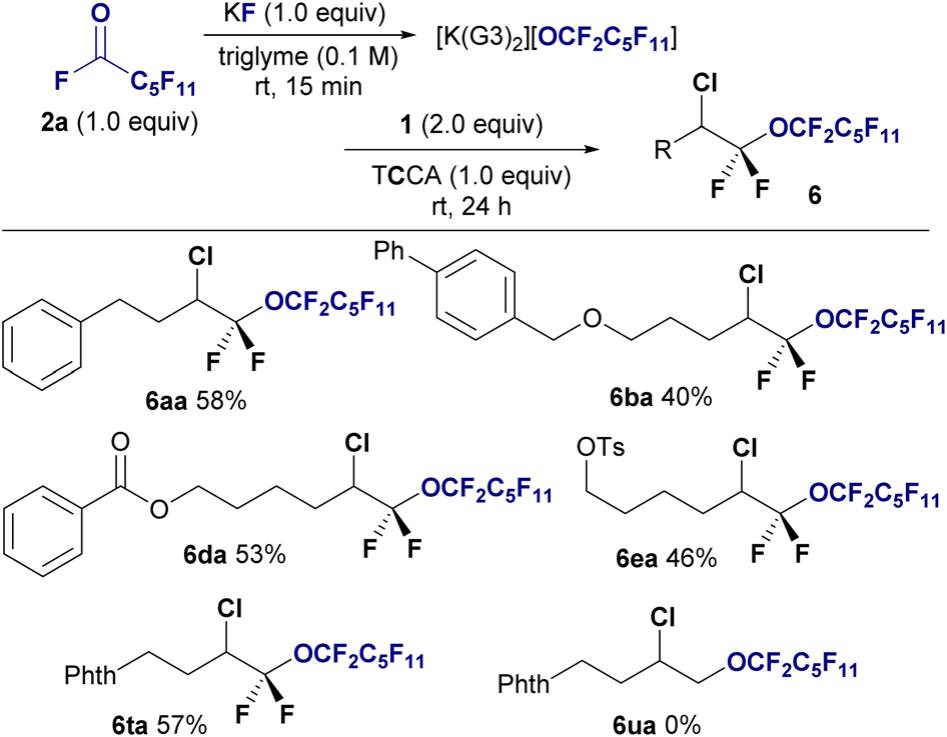
Substrate scope of the chloro-perfluoroalkoxylation of *gem*-difluoroalkenes 1 using undecafluorohexanoyl fluoride 2a. Isolated yields are shown. Reaction conditions: 1 (0.6 mmol), 2a (0.3 mmol), KF (0.3 mmol), TCCA (0.3 mmol) in triglyme (3.0 mL), stirring under atmosphere of N_2_ at room temperature.

Although I_2_ and Br_2_ are effective for this conversion, we did not investigate the use of chlorine due to its gasueous, highly oxidative nature, which makes it difficult to optimize the reaction conditions. Upon further investigation, trichloroisocyanuric acid (TCCA) was found to be a suitable chlorine source for chloro-perfluoro alkoxylation, allowing the production of bis(α,α)difluoro ethers 6 from 2a and 1 in yields ranging from to 40–58%. Chloro-perfluoroalkoxylation was also selective to fluorinated alkenes 1, with nonfluorinated alkene 1u remaining unreactive (6ua, 0%) (Fig. 4).

**Fig. 5:**
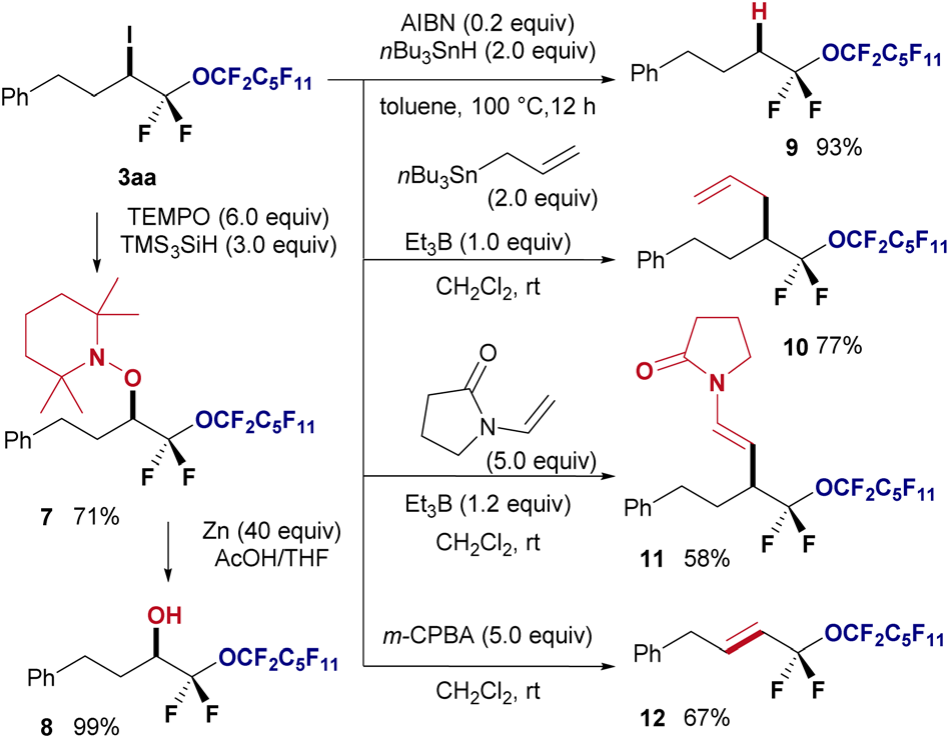
Synthetic applications.

Furthermore, several chemical transformations were performed to demonstrate the synthetic utility of the obtained halo-perfluoroalkyl ether products (Fig. 5). First, radical coupling of 3aa with TEMPO in the presence of TMS_3_SiH at room temperature resulted in the formation of the TEMPO adduct 7 in 71% yield. Subsequently, the piperidinyl protecting group of 7 was reductively removed using zinc and acetic acid, yielding oxygenated compound 8 in quantitative yield. Employing tributyl stannane facilitated the reduction of iodine in 3aa using AIBN, leading to hydrogenated product 9 in 93% yield. Additionally, the radical allylation of 3aa using triethylborane provided corresponding allylated product 10 in 77% yield. The Giese radical addition of 3aa yielded the desired olefin 11 in 58% yield. Finally, HI elimination from 3aa using *m*-CPBA afforded corresponding *E*-alkene 12 as a single isomer in 67% yield.

**Fig. 6:**
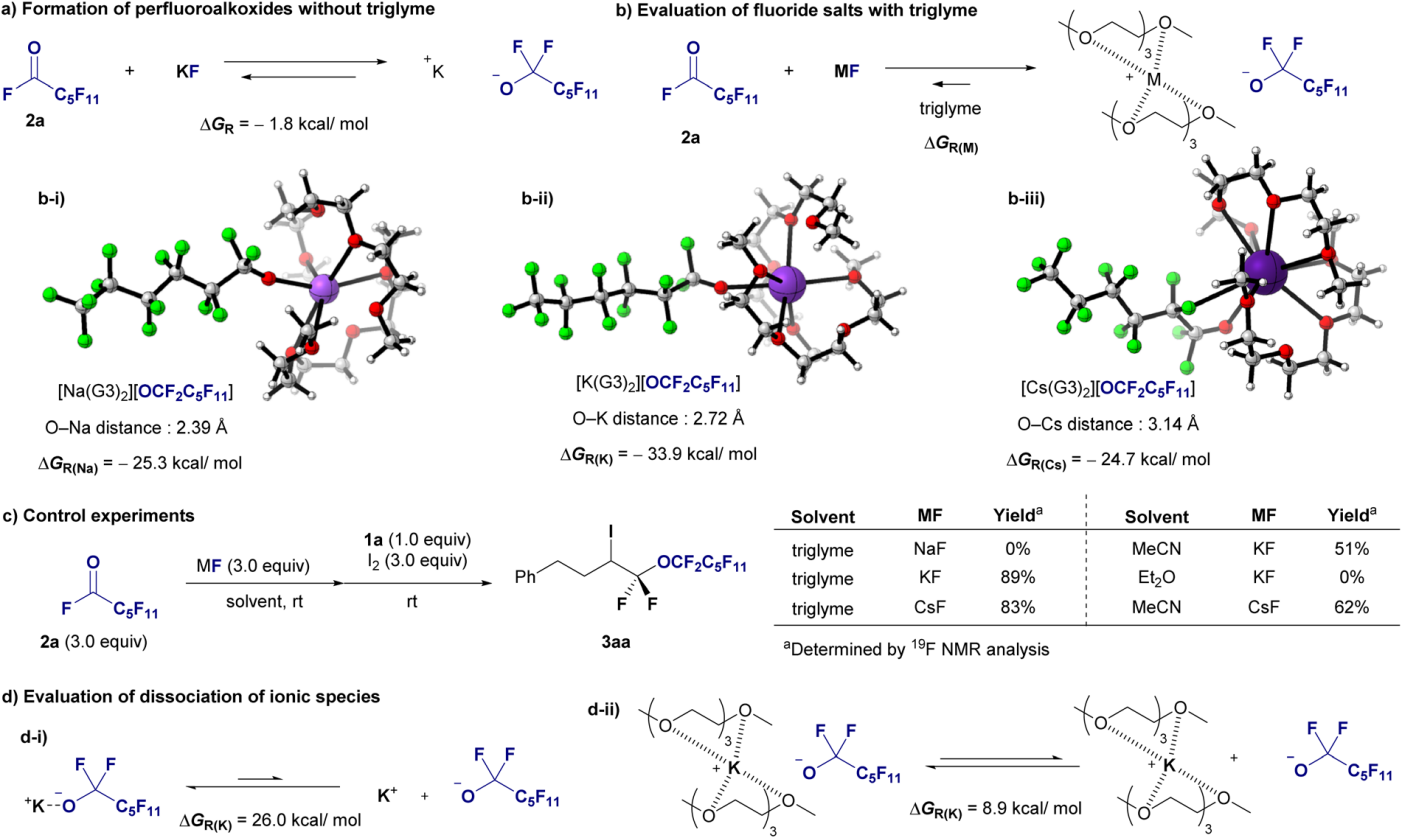
Mechanistic studies. (a) Formation of perfluoroalkoxides without triglyme. (b) Evaluation of the fluoride salt with triglyme. Optimized structures of fluoride salt with two triglyme molecules. (c) Control experiments. (d) Evaluation of dissociation of ionic species.

DFT calculations were conducted to assess the stabilization of perfluoroalkoxides in the presence of triglymes. The calculations were performed at the B3LYP-D3/6-311+G(d,p) (SDD for K and Cs) level of theory using Gaussian 16 software.^15^ As indicated in Fig. 1c. Initially, the formation of perfluoroalkoxides from KF and acylfluoride 2a in ether was calculated to be exothermic (Fig. 6a, −1.8 kcal mol^−1^). Complex formation involving two triglyme molecules (G3) coordinated to alkali metal cations resulted in complexes [Na(G3)_2_][OCF_2_C_5_F_11_], [K(G3)_2_][OCF_2_C_5_F_11_], and [Cs(G3)_2_][OCF_2_C_5_F_11_], indicating that the potassium cation had the greatest stabilizing effect (Fig. 6b, 33.9 kcal mol^−1^). The M^+^–O(G3) distances in the tetra-dentated G3 structures, that is, [Na(G3)_2_][OCF_2_C_5_F_11_], [K(G3)_2_][OCF_2_C_5_F_11_], and [Cs(G3)_2_][OCF_2_C_5_F_11_], ranged from 2.80–3.00, 2.90–3.10, and 3.20–3.40 Å, respectively. This implies that the deformation energy of G3 molecules, which is the energy increase due to the deformation of G3 geometries when G3 molecules are coordinated to the M^+^ cation, is less destabilizing in [K(G3)_2_][OCF_2_C_5_F_11_].^16^ Subsequently, a control experiment was conducted (Fig. 6c). Alkali metal fluoride salts were compared under optimal reaction conditions using triglyme: KF resulted in 89% yield, CsF yielded a slightly lower yield, and NaF did not react. The effects of solvents were further investigated. The yield decreased to 51% when the reaction was conducted in MeCN with KF, and no reaction occurred in Et_2_O. The yield of the CsF/MeCN system was greater than that of the KF/MeCN system but significantly lower than that of the CsF/triglyme system. Based on these calculations and experimental observations, the yields of the larger cation, cesium salt, were greater in MeCN, and potassium salt exhibited a greater stabilizing effect than the cesium salt in the presence of triglyme.

In addition, these outcomes might also be attributed to the enhanced reactivity of perfluoroalkoxide anions because of the better dissociation of the ionic species. We thus performed additional DFT calculations to evaluate the dissociation of ionic species (Fig. 6d). The dissociation of ionic pair [K][OCF_2_C_5_F_11_] had a Δ*G*_R_ of 26.0 kcal mol^−1^, indicating a not favourable process; whereas the process involving the encapsulation of K^+^ ions with two molecules of triglyme facilitates the dissociation of the ionic pair [K(G3)_2_][OCF_2_C_5_F_11_], with a Δ*G*_R_ of 8.9 kcal mol^−1^. As shown by the Δ*G*_R_ values, the process is more favourable in the presence of triglyme. Based on the results of Fig. 6a, b-ii, and d, both factors, stabilization and reactivity of the anion, resulted in diglyme playing important roles for the successful transformation. These two factors were more favourable in the presence of triglyme as inferred from the computed Gibbs energies, despite the dissociation is still an endergonic process. Also, the difference in Gibbs energies stabilization in the presence and the absence of triglyime (ΔΔ*G*_R_ = −33.9 − (−1.8) = −32.1 kcal mol^−1^) is more significant than that for the dissociation (ΔΔ*G*_R_ = 26.0–8.9 = 17.1 kcal mol^−1^).

## Conclusion

We designed a novel approach to enhance the stability of short-lived, longer-chain perfluoroalkoxy anions to facilitate efficient nucleophilic perfluoroalkoxylation reactions. This method involves regioselective halo-perfluoroalkoxylation of *gem*-difluoroalkenes using potassium perfluoroalkoxides generated *in situ* from KF and perfluoroacylfluorides in a triglyme with bromine, iodine, or TCCA at room temperature. This methodology is broadly applicable to substrates containing analogs derived from bioactive compounds, thus enabling the synthesis of difluoroalkyl perfluoroalkyl ethers with C_3_–C_6_ perfluoroalkyl chains. The reaction is performed under mild conditions at rt in an environmentally friendly solvent, triglyme.^17^ Moreover, it encompasses a wide range of *gem*-difluoroalkenes as substrates, producing halo-perfluoroalkoxyl adducts that are suitable for diverse chemical transformations. This methodology is highly chemoselective toward fluorinated alkenes, whereas nonfluorinated alkenes remain intact. The extension of this methodology to the synthesis of fluorinated polymers consisting of bis(α,α-difluoro)ethers (–CF_2_–O–CF_2_–) is currently under investigation.

## Data availability

The data that support the findings of this study are available within the article and the ESI.‡ Details about materials and methods, experimental procedures, characterization data, and NMR spectral are included.

## Author contributions

KK optimized the reaction conditions. KK, YK, TA, MU and SI surveyed the substrate scope, analyzed the data, and then discussed the results with NH, YK and NS. JE performed DFT calculation and analyzed the data. KK and NS wrote the manuscript. NS supervised the project. All authors contributed to the manuscript and have approved the final version of the manuscript.

## Conflicts of interest

There are no conflicts to declare.

## Supplementary Material

SC-015-D4SC02084G-s001
