# Supplementary material for: Halo-perfluoroalkoxylation of gem-difluoroalkenes with short-lived alkali metal perfluoroalkoxides in triglyme
Source: Chem Sci. 2024 May 22;15(25):9574–81. doi: 10.1039/d4sc02084g (PMC11205273; doi:10.1039/d4sc02084g)

Electronic Supplementary Material (ESI) for Chemical Science.
This journal is © The Royal Society of Chemistry 2024

Electronic supplementary information (ESI)

Halo-perfluoroalkoxylation of *gem*-difluoroalkenes with short-lived alkali metal perfluoroalkoxides in triglyme

Koki Kawai[1], Yoshimitsu Kato[2], Taichi Araki[2], Sota Ikawa[2], Mai Usui[2], Naoyuki Hoshiya[3], Yosuke Kishikawa[3], Jorge Escorihuela[4], and Norio Shibata[1,2]

[1] Department of Nanopharmaceutical Sciences, and Department of Life Science and Applied Chemistry, Nagoya Institute of Technology, Gokiso, Showa-ku, Nagoya 466-8555, Japan.

[2] Department of Life Science and Applied Chemistry Nagoya Institute of Technology Gokiso, Showa-ku, Nagoya 466-8555, Japan

[3] Technology Innovation Center DAIKIN Industries, Ltd. 1-1 Nishi-Hitotsuya, Settsu, Osaka 566-8585, Japan

[4] Departamento de Química Orgánica, Universitat de València Avda. Vicente Andrés Estellés s/n, Burjassot 46100, Valencia, Spain

*E-mail: nozshiba@nitech.ac.jp

CONTENTS

1. General information 1
2. Optimization for Chloro-perfluoroalkoxylation 2
3. Preparation of fluoroalkenes 2
4. Iode-perfluoroalkoxylation of *gem*-difluoroalkenes 1 5
5. Bromo-perfluoroalkoxylation of *gem*-difluoroalkenes 1 10
6. Bromo-perfluoroalkoxylation (C3 to C5) of *gem*-difluoroalkenes 1 14
7. Chloro-perfluoroalkoxylation of *gem*-difluoroalkenes 1 16
8. Synthetic application of compound 3aa 18
9. Computational details 21
10. References 34
11. NMR data 35

1. General information

All reactions were performed in oven-dried glassware under positive pressure of nitrogen unless otherwise mentioned. Solvents were transferred via syringe and were introduced into the reaction vessels though a rubberseptum. All of the reactions were monitored by thin-layer chromatography (TLC) carried out on 0.25 mm Merck silica gel (60-F_{254}). The TLC plates were visualized with UV light and $KMnO_4$ in water/heat. Column chromatography was carried out on columns packed with silica gel (60N spherical neutral size 63-210 nm). The ^{1}H NMR (500 MHz), ^{19}F NMR (282 MHz), ^{13}C NMR (126 MHz) spectra for solution in $CDCl_3$ were recorded on a Varian 300 and a Buruker Avance 500. Chemical shifts (δ) are expressed in ppm downfield from TMS (δ = 0.00 ppm for ^{1}H NMR), C_6F_6 (δ = −162.2 ppm for ^{19}F NMR) or $CDCl_3$ (77.16 ppm for ^{13}C NMR) as an internal standard. Mass spectra were recorded on a JEOL JMS-T2000GC (EI-MS) and SHIMAZU LCMS-2020 (ESI-MS) and JEOL JMS-700 (FAB-MS). Melting points were recorded on Buchi M-565. Infrared spectra were recorded on a JASCO FT/IR-4100 spectrometer. Chemicals were purchased and used without further purification unless otherwise noted. Solvent triglym was dried over activated MS-4A before use. Pottasium Fluoride (KF, spray dride) was purchased from FUJIFILM Wako Pure Chemical Corporation and dried overnight at 200 °C under reduced pressure and stored in Glove Box.

2. Optimization for Chloro-perfluoroalkoxylation

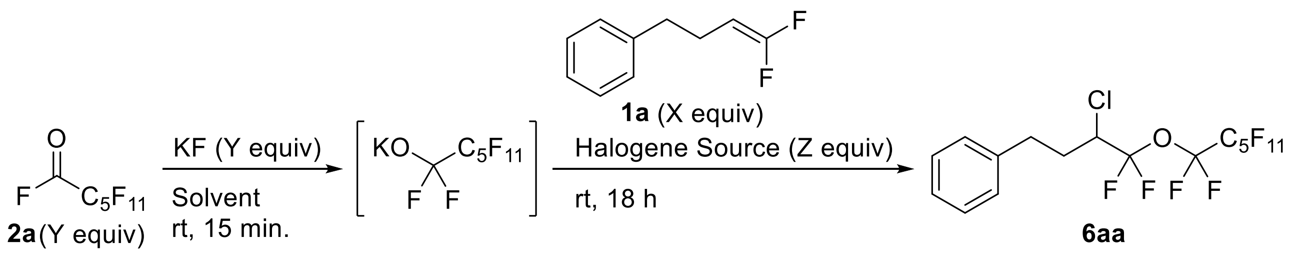

Entry	1a (X equiv)	2a (Y equiv)	Halo-source (Z equiv)	Solvent (0.1 M)	Yield (%)[a]
1	1.0	1.0	TCCA (1.0)	Triglyme	56 (57)
2	1.0	1.0	NCS (1.0)	Triglyme	0
3	1.0	1.0	DCDMH (1.0)	Triglyme	0
4	1.0	-	TCCA (1.0)	Triglyme	-
5	1.0	1.0	TCCA (1.0)	Triglyme (40 °C)	52
6	1.0	1.0	TCCA (0.33)	Triglyme	32
7	1.0	1.0	TCCA (0.67)	Triglyme	54
**8**	**1.0**	**1.5**	**TCCA (1.0)**	**Triglyme**	**72**
9	1.0	1.5	TCCA (1.5)	Triglyme	65
10	1.0	2.0	TCCA (1.0)	Triglyme	73
**11**	**2.0**	**1.0**	**TCCA (1.0)**	**Triglyme**	**73**
12	2.0	1.0	TCCA (1.5)	Triglyme	58
13	1.0	1.0	TCCA (1.0)	Triglyme/Toluene (1:1)	29

Reaction conditions: **1a**, **2a**, KF, Halogen source, solvent (1.0 mL), room temperature for 24 h. 0.1 mmol scale. [a] ^{19}F NMR yields were determined using C_6F_6 as an internal standard.

3. Preparation of fluoroalkenes

3-1. General procedure A

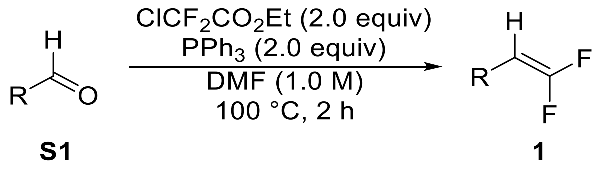

Sodium 2-chloro-2, 2-difluoroacetate (2.0 equiv) was added in the mixture of corresponding aldehyde **S1** (1.0 equiv) and Triphenyl phosphine (2.0 equiv) in DMF (1.0 M), the reaction was heated at 100 °C and kepted at this temperature until no further evolution of CO_2 was observed. Then the reaction mixture was cooled to rt, and water was added to the reaction slowly, the mixture was extracted with Et_2O 3 times. The combined organic layer was washed with brine, dried over Na_2SO_4, filtrated, and concentrated in vacuo. The residue was purified by flash column chromatography to afford the difluoroalkenes **1**[1-3].

3-2. General procedure B

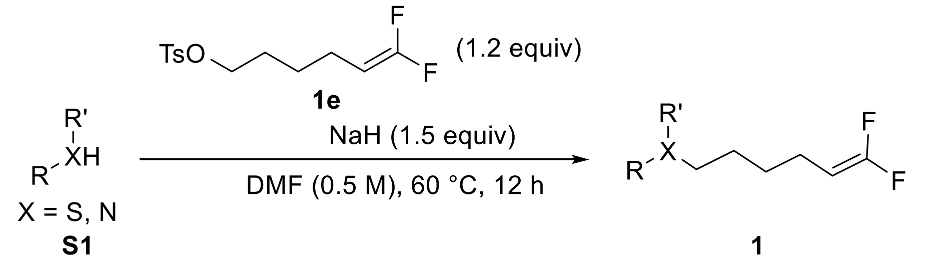

S1 was suspended in DMF (0.5 M). Then, NaH (1.0 equiv, 60% dispersion in Paraffin Liquid) was added in the solution. The reaction was stirred at room temperature for 15 min. To the above mixture, 6,6-difluorohex-5-en-1-yl 4-methylbenzenesulfonate (**1e**: 1.2 equiv) was added and the reaction mixture was stirred at 60 °C for 12 h under nitrogen gas. The solution was cooled to 0 °C and was quenched with NH_4Cl aq. The mixture was extracted with EtOAc 3 times, and the combined organic was washed with brine, dried over Na_2SO_4, filtered and concentrated. The residue was purified by flash column chromatography to afford the difluoroalkenes **1**[4-6].

4-(((5,5-difluoropent-4-en-1-yl)oxy)methyl)-1,1'-biphenyl (1b)

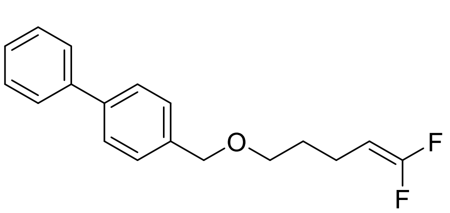

1b (599 mg, 2.1 mmol, 44% yield) was obtained by **General Procedure A** using aldehyde **S1b** (1.19 g, 4.7 mmol, 1.0 equiv), Triphenyl phosphine (2.47 g, 9.4 mmol, 2.0 equiv), Sodium 2-chloro-2, 2-difluoroacetate (1.43 g, 9.4 mmol, 2.0 equiv). The crude **1b** was purified by flash chromatography on silica gel (Hexane). Colorless oil. **HRMS** (FAB) *m/z*: $[M + H]^+$ calculated for $C_{18}H_{19}F_2O$ 289.1404; found: 289.1403. **^{1}H NMR** (500 MHz, $CDCl_3$) δ 7.59 – 7.56 (m, 4H), 7.44 – 7.39 (m, 4H), 7.35 – 7.32 (m, 1H), 4.53 (s, 2H), 4.18 – 4.10 (m, 1H), 3.51 – 3.49 (m, 2H), 2.12 – 2.08 (m, 2H), 1.73 – 1.67 (quin, *J* = 7.0 Hz, 2H). **^{13}C NMR** (126 MHz, $CDCl_3$) δ 156.5 (t, *J* = 285.6 Hz), 141.0, 140.7, 137.6, 128.9 (2C), 128.2 (2C), 127.4, 127.3 (2C), 127.2 (2C), 77.7 (t, *J* = 21.3 Hz), 72.9, 69.4, 29.6, 19.2 (d, *J* = 4.5 Hz). **^{19}F NMR** (282 MHz, $CDCl_3$) δ -89.6 (d, *J* = 47.6 Hz, 1F), -92.0 (dd, *J* = 48.1, 25.3 Hz, 1F). **IR (KBr)**: 3478, 2943, 2859, 2340, 1910, 1748, 1227, 1104, 764, 700 cm^{-1}.

1-(6,6-difluorohex-5-en-1-yl)indoline-2,3-dione (1f)

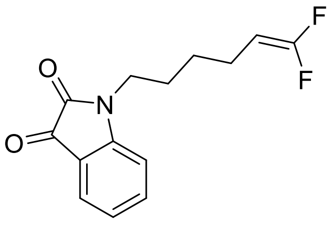

1f (448 mg, 1.2 mmol, 68% yield) was obtained by **General Procedure B** using Isatin (443 mg, 3.0 mmol, 1.2 equiv), **1e** (726 mg, 2.5 mmol, 1.0 equiv), NaH (151 mg, 3.8 mmol, 1.5 equiv, 60% dispersion in Paraffin Liquid). The crude **1f** was purified by flash chromatography on silica gel (Hexane/CH_2Cl_2/Et_2O/= 8:1:1). Orange oil. **HRMS** (ESI) *m/z*: $[M + Na]^+$ calculated for $C_{14}H_{13}F_2NNaO_2$ 288.0812; found: 288.0815. **^{1}H NMR** (500 MHz, $CDCl_3$) δ 7.62 – 7.59 (m, 2H), 7.15 – 7.11 (m, 1H), 6.92 – 6.90 (m, 1H), 4.12 (dtt, *J* = 25.5, 7.9, 2.4 Hz, 1H), 3.74 (td, *J* = 7.2, 2.3 Hz, 2H), 2.07 – 2.03 (m, 2H), 1.73 (quin, *J* = 7.5Hz, 2H), 1.48 (quin, *J* = 5.5Hz, 2H). **^{13}C NMR** (126 MHz, $CDCl_3$) δ 183.6, 158.8, 156.5 (t, *J* = 286.7 Hz), 150.9, 138.5, 125.6, 123.8, 117.6, 110.2, 77.4, 40.0, 26.7, 26.6, 21.8 (d, *J* = 3.7 Hz). **^{19}F NMR** (282 MHz, $CDCl_3$) δ -88.7 (d-like, *J* = 47.6 Hz, 1F), -91.2 (dd, *J* = 47.6, 25.8 Hz, 1F). **IR (KBr)**: 3460, 3065, 2937, 2869, 1743, 1617, 1470, 1362, 1191, 811, 754, 474 cm^{-1}.

7-(6,6-difluorohex-5-en-1-yl)-1,3-dimethyl-3,7-dihydro-1*H*-purine-2,6-dione (1n)

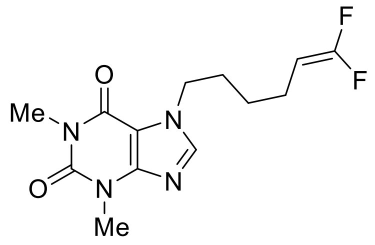

1n (334 mg, 1.1 mmol, 75% yield) was obtained by **General Procedure B** using Theophylline (269 mg, 1.5 mmol), **1e** (524 mg, 1.8 mmol, 1.2 equiv), NaH (66.0 mg, 1.7 mmol, 1.1 equiv, 60%, dispersion in Paraffin Liquid). The crude **1n** was purified by flash chromatography on silica gel (Hexane/Ethyl Acetate = 1:2). White Solid. **Mp**. = 50.7 – 51.4 °C (Chloroform). **HRMS** (EI) *m/z*: $[M]^+$ calculated for $C_{13}H_{16}F_2N_4O_2$ 298.1241; found: 298.1271. ^{1}**H NMR** (500 MHz, $CDCl_3$) δ 7.55 (s, 1H), 4.30 (t, *J* = 7.2 Hz, 2H), 4.12 (dtd, *J* = 25.3, 7.9, 2.4 Hz, 1H), 3.60 (s, 3H), 3.42 (s, 3H), 2.03 (qt, *J* = 7.5, 1.5 Hz, 2H), 1.90 (quin, *J* = 8.0 Hz, 2H), 1.41 (quin, *J* = 7.5 Hz, 2H). ^{13}**C NMR** (126 MHz, $CDCl_3$) δ 156.5 (t, *J* = 286.7 Hz), 155.2, 151.8, 149.0, 140.9, 107.0, 77.4, 47.0, 30.3, 29.9, 28.1, 26.3, 21.8 (d, *J* = 4.7 Hz). ^{19}**F NMR** (282 MHz, $CDCl_3$) δ -89.1 (d, *J* = 46.6 Hz, 1F), -91.6 (dd, *J* = 47.1, 25.3 Hz, 1F). **IR (KBr)**: 3488, 3119, 2937, 2864, 2349, 1752, 1711, 1650, 1546 cm^{-1}.

(4-chlorophenyl)(3-(3,3-difluoroallyl)-5-methoxy-2-methyl-1*H*-indol-1-yl)methanone (1o)

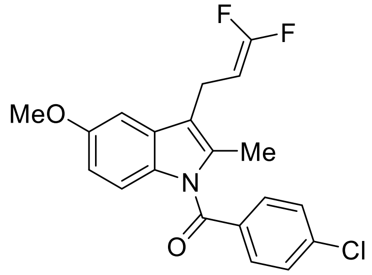

1o (780 mg, 2.1 mmol, 39% yield) was obtained by **General Procedure A** using aldehyde **S1o** (1.799 g, 5.26 mmol), Triphenylphosphine (2.772 g, 10.5 mmol, 2.0 equiv.), Sodium Chlorodifluoroacetate (3.219 g, 21.0 mmol, 4.0 equiv.). The crude **1o** was purified by flash chromatography on silica gel (Hexane/Ethyl Acetate = 98:2). White Solid. **Mp**. = 60.2 – 60.7 °C (Chloroform). **HRMS** (ESI) *m/z*: $[M + Na]^+$ calculated for $C_{20}H_{16}ClF_2NNaO_2$ 398.0735; found: 398.0733. ^{1}**H NMR** (500 MHz, $CDCl_3$) δ 7.66 – 7.64 (m, 2H), 7.48 – 7.45 (m, 2H), 6.90 (d, *J* = 2.7 Hz, 1H), 6.86 (d, *J* = 9.2 Hz, 1H), 6.67 (dd, *J* = 9.0, 2.6 Hz, 1H), 4.32 (dtd, *J* = 24.8, 7.8, 2.1 Hz, 1H), 3.84 (s, 3H), 3.34 (dt, *J* = 7.8, 1.6 Hz, 2H), 2.35 (s, 3H). ^{13}**C NMR** (126 MHz, $CDCl_3$) δ 168.4, 156.5 (t, *J* = 287.5 Hz), 156.0, 139.3, 134.5, 134.1, 131.2 (2C), 130.8, 130.6, 129.2 (2C), 117.1, 115.2, 111.4, 101.2, 77.4, 55.8, 17.4 (d, *J* = 4.5 Hz), 13.2. ^{19}**F NMR** (282 MHz, $CDCl_3$) δ -89.4 (d-like, *J* = 46.6 Hz, 1F), -91.2 (dd, *J* = 46.6, 24.8 Hz, 1F). **IR (KBr)**: 3530, 3356, 2926, 2834, 2066, 1914, 1751, 1677, 1599, 1474 cm^{-1}.

2-(1-(6,6-difluorohex-5-en-1-yl)-2,6-dioxopiperidin-3-yl)isoindoline-1,3-dione (1r)

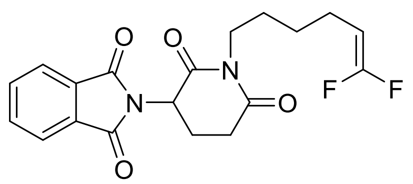

1r (289 mg, 0.77 mmol, 68% yield) was obtained by **General Procedure B** using Thalidomide (292 mg, 1.1 mmol), **1e** (394 mg, 1.4 mmol, 1.2 equiv), NaH (67.0 mg, 1.7 mmol, 1.5 equiv, 60%, dispersion in Paraffin Liquid). The crude **1r** was purified by flash chromatography on silica gel (Hexane/Et_2O/CH_2Cl_2 =2/1/2). Pale yellow oil. **HRMS** (ESI) *m/z*: $[M + H]^+$ calculated for $C_{19}H_{19}F_2N_2O_4$ 377.1313; found: 377.1310. ^{1}**H NMR** (500 MHz, $CDCl_3$) δ 7.90 – 7.87 (m, 2H), 7.78 – 7.76 (m, 2H), 5.01 – 4.97 (m, 1H), 4.12 (dtt, *J* = 25.5, 7.8, 2.5 Hz, 1H), 3.85 – 3.76 (m, 2H), 3.02 – 2.94 (m, 1H), 2.84 – 2.73 (m, 2H), 2.15 – 2.11 (m, 1H), 2.02 – 1.99 (m, 2H), 1.60 – 1.53 (m, 2H), 1.43 – 1.37 (m, 2H). ^{13}**C NMR** (126 MHz, $CDCl_3$) δ 171.0, 168.6, 167.5 (2C), 156.4 (t, *J* = 286.7 Hz), 134.5 (2C), 131.8 (2C), 123.8 (2C), 77.7 (t, *J* = 21.9 Hz), 50.2, 40.4, 32.1, 27.2, 26.7, 22.1, 21.9 (d, *J* = 3.7 Hz). ^{19}**F NMR** (282 MHz, $CDCl_3$) δ -89.7 (d-like, *J* = 48.6 Hz, 1F), -92.1 (dd, *J* = 48.1, 25.3 Hz, 1F). **IR (KBr)**: 3483 2945, 2866, 2340, 1723, 1683, 1391, 1152, 721, 530, 475 cm^{-1}.

3-3. preparation of 6,6-difluorohex-5-en-1-yl 4-methylbenzenesulfonate (1e)

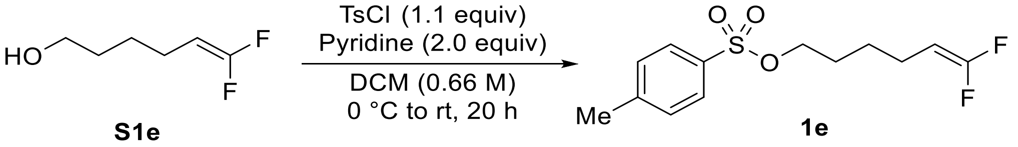

Under N_2 atmosphere, in a flame dried round bottom flask, 6,6-difluorohex-5-en-1-ol (**S1e**: 1.97 g, 14.5 mmol) and Pyridine (2.28 g, 28.9 mmol, 2.0 equiv) was dissolved in CH_2Cl_2 (0.66 M). The solution was cooled to 0 °C. To the above mixture, *p*-Toluenesulfonyl Chloride (3.03 g, 15.9 mmol, 1.1 equiv.) was added, and the reaction mixture was stirred at rt for 12 h. After completion, the solution was quenched with water, and extracted with CH_2Cl_2 3 times. Combined organic layers were finally washed with brine solution, dried over anhydrous Na_2SO_4 and then solvent was removed under reduced pressure. The crude product was purified by silica gel chromatography (Hexane/Et_2O = 97:3 to 9/1) to obtain as colourless oil (3.19 g, 11.0 mmol, 76%).

HRMS (ESI) *m/z*: $[M + Na]^+$ calculated for $C_{13}H_{16}F_2NaO_3S$ 313.0686; found: 313.0698. ^{1}**H NMR** (500 MHz, $CDCl_3$) δ 7.79 (d, *J* = 8.2 Hz, 2H), 7.35 (d, *J* = 8.5 Hz, 2H), 4.05 (dtd, *J* = 27.5, 8.0, 2.5 Hz, 1H (-CHF_2), and t, *J* = 6.5 Hz, 2H (-OCH_2 -) are overlapped), 2.45 (s, 3H), 1.94 (qt, *J* = 7.5, 1.5 Hz, 2H), 1.65 (quin, *J* = 6.5 Hz, 2H) 1.40 (quin, *J* = 7.5 Hz, 2H). ^{13}**C NMR** (126 MHz, $CDCl_3$) δ 156.5 (t, *J* = 287.7 Hz), 144.9, 133.1, 130.0 (2C), 128.0 (2C), 77.4, 70.2, 28.1, 25.3, 21.7, 21.6 (d, *J* = 4.5 Hz). ^{19}**F NMR** (282 MHz, $CDCl_3$) δ -89.3 (d-like, *J* = 46.6 Hz, 1F), -91.8 (dd, *J* = 47.6, 24.8 Hz, 1F). **IR (NaCl)**: 3713, 3628, 3409, 2947, 1747, 1359, 1176, 941, 664, 661, 488 cm^{-1}.

4. Iode-perfluoroalkoxylation of *gem*-difluoroalkenes 1

4-1. General Procedure C for Iode-perfluoroalkoxylatyion

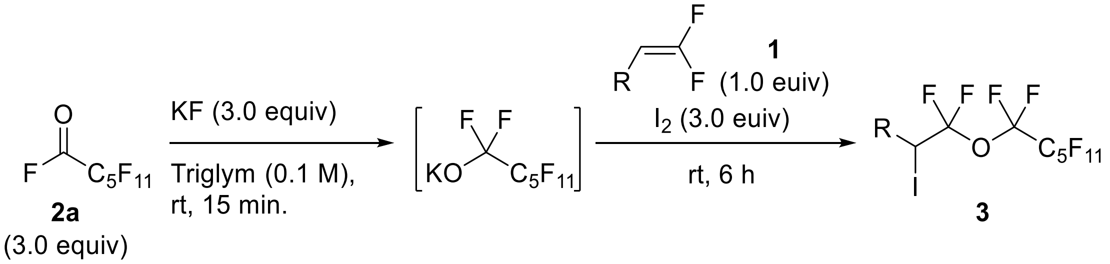

In an nitrogene gas-filled glovebox, KF (0.9 mmol, 3.0 eq), triglym (3.0 mL), and perfluoroacyl fluoride (0.9 mmol, 3.0 eq) was added to a 20 mL round-bottom flask equipped with a magnetic stir bar. After stirring for 15 min at room temptrture, fluoroalkene (0.3 mmol, 1.0 eq) was added, followed by one-portion addition of I_2 (0.9 mmol, 3.0 eq) and the mixture was stirred at room tempereture for 6 h. The reaction mixture was quenched with saturated $NaHCO_3$aq and Na_2SO_3aq. The product was extracted with Et_2O (10 mL x 3) and washed with Brine. The organic phase was dried over anhydrous Na_2SO_4 and concentrated under reduced pressure. The product was purified by column chromatography on silica gel using hexane or hexane/Ethyl Acetate as eluent.

(4,4-difluoro-3-iodo-4-((perfluorohexyl)oxy)butyl)benzene (3aa)

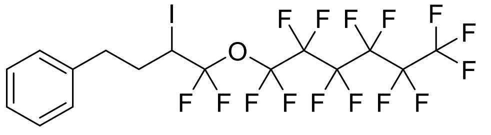

(a) **3aa** (177.9 mg, 0.282 mmol, 94% yield) was obtained by **general procedure C** using fluoroalkene **1a** (50.5 mg, 0.30 mmol), 2,2,3,3,4,4,5,5,6,6,6-undecafluorohexanoyl fluoride **2a** (0.18 mL, 0.90 mmol), KF (52.3 mg, 0.90 mmol) and I_2 (228 mg, 0.90 mmol). The crude **3aa** was purified by flash chromatography on silica gel (Hexane). (b) Gram scale reaction. **3aa** (3.47 g, 5.51 mmol, 92% yield) was obtained by **general procedure C** using fluoroalkene **1a** (1.01 g, 6.0 mmol), 2,2,3,3,4,4,5,5,6,6,6-undecafluorohexanoyl fluoride **2a** (5.7 g, 18 mmol), KF (1.05 g, 18 mmol) and I_2 (4.56 g, 18 mmol). The crude **3aa** was purified by flash chromatography on silica gel (Hexane). Colorless oil. **HRMS** (EI) *m/z*: $[M]^+$ calculated for $C_{16}H_{10}F_{15}IO$ 629.9537; found: 629.9545. **^{1}H NMR** (500 MHz, $CDCl_3$) δ 7.33 – 7.31 (m, 2H), 7.26 – 7.17 (m, 3H), 4.04 – 3.98 (m, 1H), 3.02 – 2.97 (m, 1H), 2.71 – 2.65 (m, 1H), 2.21 – 2.09 (m, 2H). **^{13}C NMR** (126 MHz, $CDCl_3$) δ 139.2, 128.9 (2C), 128.6 (2C), 126.9, 123.2 (t, *J* = 279.3 Hz), 120.7 – 105.9 (m), 34.4, 34.2, 25.6 (t, *J* = 28.2 Hz). **^{19}F NMR** (282 MHz, $CDCl_3$) δ: -71.0 to -71.2 (m, 2F), -81.2 (t-like, 3F), -84.2 (br, 2F), -122.7 (br, 2F), -123.4 (br, 2F), -126.1 (br, 2F), -126.6 to -126.7 (m, 2F). **IR (KBr)**: 3734, 3286, 3033, 2951, 2312, 1456, 1242, 1206, 700, 506 cm^{-1}.

4-(((5,5-difluoro-4-iodo-5-((perfluorohexyl)oxy)pentyl)oxy)methyl)-1,1'-biphenyl (3ba)

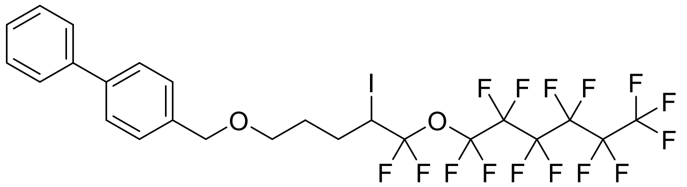

3ba (184.7 mg, 0.246 mmol, 82% yield) was obtained by **general procedure C** using fluoroalkene **1b** (86.5 mg, 0.30 mmol), 2,2,3,3,4,4,5,5,6,6,6-undecafluorohexanoyl fluoride **2a** (0.18 mL, 0.90 mmol), KF (51.8 mg, 0.89 mmol) and I_2 (225 mg, 0.89 mmol). The crude **3ba** was purified by flash chromatography on silica gel (Hexane/Ethyl Acetate = 19:1). Pale yellow oil. **HRMS** (ESI) *m/z*: $[M + Na]^+$ calculated for $C_{24}H_{18}F_{15}INaO_2$ 773.0010; found: 772.9973. **^{1}H NMR** (500 MHz, $CDCl_3$) δ 7.63 – 7.60 (m, 4H), 7.48 – 7.45 (m, 2H), 7.44 – 7.41 (m, 2H), 7.39 – 7.36 (m, 1H), 4.58 (s, 2H), 4.30 – 4.24 (m, 1H), 3.62 – 3.54 (m, 2H), 2.14 – 2.07 (m, 1H), 2.00 – 1.90 (m, 2H), 1.79 – 1.70 (m, 1H). **^{13}C NMR** (126 MHz, $CDCl_3$) δ 141.0, 140.8, 137.3, 128.9 (2C), 128.2 (2C), 127.4, 127.3 (2C), 127.2 (2C), 123.2 (t, *J* = 279.3 Hz), 120.7 – 106.2 (m), 72.9, 68.8, 30.1, 29.3, 26.3 (t, *J* = 28.2 Hz). **^{19}F NMR** (282 MHz, $CDCl_3$) δ: -70.5 to -72.1 (m, 2F), -81.2 (m, 3F), -83.9 to -84.1 (m, 2F), -122.5 (br, 2F), -123.3 (br, 2F), -126.0 (br, 2F), -126.5 to -126.6 (m, 2F). **IR (KBr)**: 3448, 3032, 2947, 2863, 2346, 1490, 1245, 1207, 1105, 761, 419 cm^{-1}.

1-(((6,6-difluoro-5-iodo-6-((perfluorohexyl)oxy)hexyl)oxy)methyl)-4-methoxybenzene (3ca)

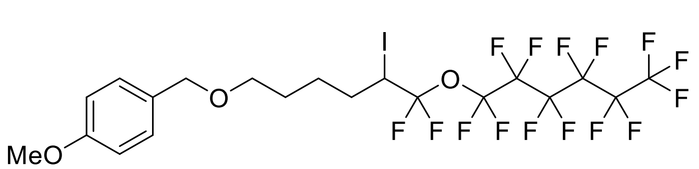

3ca (181.5 mg, 0.253 mmol, 84% yield) was obtained by **general procedure C** using fluoroalkene **1c** (76.9 mg, 0.30 mmol), 2,2,3,3,4,4,5,5,6,6,6-undecafluorohexanoyl fluoride **2a** (0.18 mL, 0.90 mmol), KF (52.5 mg, 0.90 mmol) and I_2 (222.2 mg, 0.88 mmol). The crude **3ca** was purified by flash chromatography on silica gel (Hexane/Ethyl Acetate = 99:1). Colorless oil. **HRMS** (ESI) *m/z*: $[M + Na]^+$ calculated for $C_{20}H_{18}F_{15}INaO_3$ 740.9959; found: 740.9988. **^{1}H NMR** (500 MHz, $CDCl_3$) δ 7.28 – 7.25 (m, 2H), 6.90 – 6.87 (m, 2H), 4.44 – 4.43 (m, 2H), 4.19 – 4.12 (m, 1H), 3.81 (s, 3H), 3.48 – 3.43 (m, 2H), 1.89 – 1.58 (m, 5H), 1.51 – 1.43 (m, 1H). **^{13}C NMR** (126 MHz, $CDCl_3$) δ 159.3, 130.6, 129.4 (2C), 123.2 (t, *J* = 279.5 Hz), 120.7-115.3 (m), 113.9 (2C), 113.5 to 105.9 (m), 72.8, 69.5, 55.4, 32.5, 28.7, 26.3 (t, *J* = 27.3 Hz), 26.1. **^{19}F NMR** (282 MHz, $CDCl_3$) δ -70.7 to -72.2 (m, 2F), -81.2 (t, *J* = 9.9 Hz ,3F), -84.1 to -84.3 (m, 2F), -122.7 (br, 2F), -123.4 (br, 2F), -126.1 (br, 2F), -126.6 to -126.7 (m, 2F). **IR (KBr)**: 3507, 2951, 2864, 2359, 1749, 1613, 1513, 1249, 1101, 822, 711 cm^{-1}.

6,6-difluoro-5-iodo-6-((perfluorohexyl)oxy)hexyl benzoate (3da)

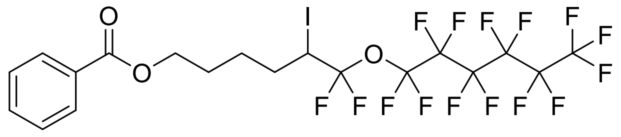

3da (180.8 mg, 0.257 mmol, 86% yield) was obtained by **general procedure C** using fluoroalkene **1d** (72.8 mg, 0.30 mmol), 2,2,3,3,4,4,5,5,6,6,6-undecafluorohexanoyl fluoride **2a** (0.18 mL, 0.90 mmol), KF (52.5 mg, 0.89 mmol) and I_2 (222 mg, 0.87 mmol). The crude **3da** was purified by flash chromatography on silica gel (Hexane/Et_2O = 98:2). Pale yellow oil. **HRMS** (ESI) *m/z*: $[M + Na]^+$ calculated for $C_{19}H_{14}F_{15}INaO_3$ 724.9646; found: 724.9621. **^{1}H NMR** (500 MHz, $CDCl_3$) δ: 8.05 – 8.04 (m, 2H), 7.58 – 7.55 (m, 1H), 7.46 – 7.43 (m, 2H), 4.39 – 4.32 (m, 2H), 4.22 – 4.16 (m, 1H), 1.95 – 1.78 (m, 5H), 1.59 – 1.53 (m, 1H). **^{13}C NMR** (126 MHz, $CDCl_3$) δ: 166.7, 133.1, 130.3, 129.7 (2C), 128.5 (2C), 123.1 (t, *J* = 279.5 Hz), 120.7-106.2 (m), 64.4, 32.5, 27.8, 25.9 (t, *J* = 30.9 Hz).**^{19}F NMR** (282 MHz, $CDCl_3$)) δ -70.6 to -72.2 (m, 2F), -81.2 (t, *J* = 9.9 Hz, 3F), -84.1 to -84.2 (m, 2F), -122.7 (br, 2F), -123.4 (br, 2F), -126.1 (br, 2F), -126.6 to -126.7 (m, 2F). **IR (KBr)**: 2954, 2362, 1720, 1700, 1604, 1455, 1247, 1205, 714, 530, 420 cm^{-1}.

6,6-difluoro-5-iodo-6-((perfluorohexyl)oxy)hexyl 4-methylbenzenesulfonate (3ea)

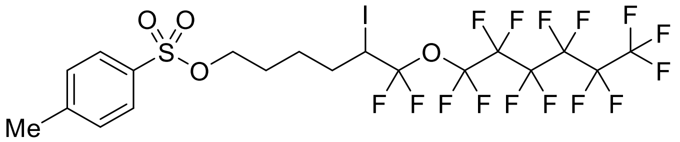

3ea (215.6 mg, 0.287 mmol, 96% yield) was obtained by **general procedure C** using fluoroalkene **1e** (87.1 mg, 0.30 mmol), 2,2,3,3,4,4,5,5,6,6,6-undecafluorohexanoyl fluoride **2a** (0.18 mL, 0.90 mmol), KF (52.3 mg, 0.90 mmol) and I_2 (222.3 mg, 0.87 mmol). The crude **3ea** was purified by flash chromatography on silica gel (Hexane/Ethyl Acetate = 19:1). Colorless oil. **HRMS** (ESI) *m/z*: $[M + Na]^+$ calculated for $C_{19}H_{16}F_{15}INaO_4S$ 774.9472; found: 774.9478. **^{1}H NMR** (500 MHz, $CDCl_3$) δ 7.81 – 7.79 (m, 2H), 7.36 (d-like, *J* = 8.2 Hz, 2H), 4.06 (m, 1H (– CHI –), and t, *J* = 6.0 Hz, 2H (– OCH_2 –) are overlapped), 2.46 (s, 3H), 1.84 – 1.62 (m, 5H), 1.44 – 1.35 (m, 1H). **^{13}C NMR** (126 MHz, $CDCl_3$) δ 145.1, 133.1, 130.0 (2C), 128.0 (2C), 123.0 (t, *J* = 279.3 Hz), 118.5-105.4 (m), 69.8, 32.1, 28.0, 25.5 (t, *J* = 27.3 Hz), 25.3, 21.8. **^{19}F NMR** (282 MHz, $CDCl_3$) δ -70.5 to -72.2 (m, 2F), -81.1 (t-like, 3F), -83.9 to -84.1 (m, 2F), -122.5 (br, 2F), -123.2 (br, 2F), -125.9 (br, 2F), -126.4 to -126.5 (m, 2F). **IR (KBr)**: 3638, 2957, 2871, 2355, 1749, 1600, 1245, 1208, 938, 665, 554 cm^{-1}.

1-(6,6-difluoro-5-iodo-6-((perfluorohexyl)oxy)hexyl)indoline-2,3-dione (3fa)

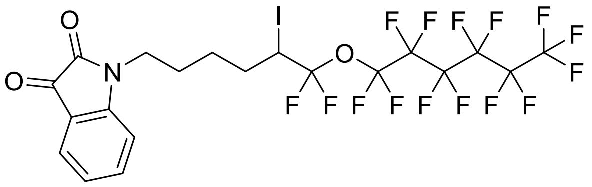

3fa (194.9 mg, 0.268 mmol, 89% yield) was obtained by **general procedure C** using fluoroalkene **1f** (78.5 mg, 0.30 mmol), 2,2,3,3,4,4,5,5,6,6,6-undecafluorohexanoyl fluoride **2a** (0.18 mL, 0.90 mmol), KF (52.1 mg, 0.89 mmol) and I_2 (226 mg, 0.90 mmol). The crude **3fa** was purified by flash chromatography on silica gel (Hexane/Et_2O = 99:1). Orange oil. **HRMS** (ESI) *m/z*: $[M + Na]^+$ calculated for $C_{20}H_{13}F_{15}INNaO_3$ 749.9598; found: 749.9625. **^{1}H NMR** (500 MHz, $CDCl_3$) δ 7.63 – 7.59 (m, 2H), 7.14 (t-like, *J* = 7.5 Hz, 1H), 6.90 (d, *J* = 7.9 Hz, 1H), 4.17 – 4.11 (m, 1H), 3.81 – 3.72 (m, 2H), 1.96 – 1.70 (m, 5H), 1.51 – 1.43 (m, 1H). **^{13}C NMR** (126 MHz, $CDCl_3$) δ 183.4, 158.3, 150.8, 138.5, 125.8, 124.0, 123.9, 123.0 (t, *J* = 279.5 Hz), 120.7-117.8 (m), 117.7, 116.3-110.2 (m), 110.1, 109.1-106.4 (m), 39.9, 32.2, 26.6, 26.4, 25.6 (t, *J* =28.2 Hz). **^{19}F NMR** (282 MHz, $CDCl_3$) δ: -70.7 to -72.4 (m, 2F), -81.2 (t, *J* = 9.9 Hz, 3F), -84.1 to -84.3 (m, 2F), -122.7 (br, 2F), -123.4 (br, 2F), -126.1 (br, 2F), -126.6 to -126.7 (m, 2F). **IR (KBr)**: 3646, 3284, 2937, 2352, 2318, 1742, 1606, 1459, 1208, 752, 472 cm^{-1}.

1,2,3,4,5-pentafluoro-6-(1,1,2,3,3-pentafluoro-2-iodo-3-((perfluorohexyl)oxy)propyl)benzene (3ga)

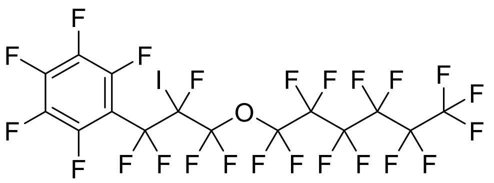

3ga (184.1mg, 0.242 mmol, 81% yield) was obtained by **general procedure C** using fluoroalkene **1g** (89.2 mg, 0.30 mmol), 2,2,3,3,4,4,5,5,6,6,6-undecafluorohexanoyl fluoride **2a** (0.18 mL, 0.90 mmol), KF (52.0 mg, 0.90 mmol) and I_2 (222 mg, 0.87 mmol). The crude **3ga** was purified by flash chromatography on silica gel (Hexane). Colorless oil. **HRMS** (FAB) *m/z*: $[M]^+$ calculated for $C_{15}F_{23}IO$ 759.8627; found: 759.8615. **^{13}C NMR** (126 MHz, $CDCl_3$) δ 146.7 – 137.0 (m), 121.6 – 104.9 (m), 83.9 – 80.7 (m). **^{19}F NMR** (282 MHz, $CDCl_3$) δ -79.4 to -80.8 (m, 2F), -86.0 (t, J = 9.9 Hz, 3F), -87.8 to -89.1 (m, 2F), -91.6 to -92.8 (m, 1F), -101.2 to -102.6 (m, 1F), -127.4 (br, 2F), -128.0 (br, 2F), -130.6 (br, 2F), -131.3 to -131.4 (m, 2F), -140.2 to -140.5 (m, 2F), -148.3 to -148.6 (m, 1F), -151.4 to -151.6 (m, 1F), -164.5 to -164.8 (m, 2F). **IR (KBr)**: 2338, 1654, 1531, 1511, 1245, 1210, 999, 714, 655, 420 cm^{-1}.

7-(6,6-difluoro-5-iodo-6-((perfluorohexyl)oxy)hexyl)-1,3-dimethyl-3,7-dihydro-1*H*-purine-2,6-dione (3na)

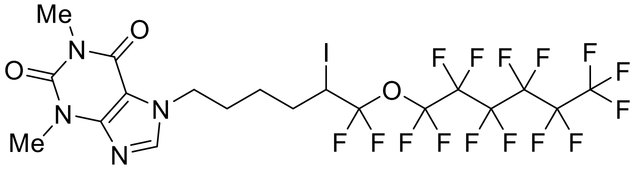

3na (171.4 mg, 0.225 mmol, 75% yield) was obtained by **general procedure C** using fluoroalkene **1n** (89.6 mg, 0.30 mmol), 2,2,3,3,4,4,5,5,6,6,6-undecafluorohexanoyl fluoride **2a** (0.18 mL, 0.90 mmol), KF (52.5 mg, 0.90 mmol) and I_2 (229 mg, 0.90 mmol). The crude **3na** was purified by flash chromatography on silica gel (Hexane/Ethyl Acetate = 1:2). White solid. **Mp**. = 68.0 – 68.6 °C (Chloroform). **HRMS** (FAB) *m/z*: $[M + H]^+$ calculated for $C_{19}H_{17}F_{15}IN_4O_3$ 761.0106; found: 761.0103. **^{1}H NMR** (500 MHz, $CDCl_3$) δ 7.57 (s, 1H), 4.37 – 4.27 (m, 2H), 4.20 – 4.14 (m, 1H), 3.60 (s, 3H), 3.42 (s, 3H), 2.03 – 1.82 (m, 4H), 1.72 – 1.63 (m, 1H), 1.46 – 1.37 (m, 1H).**^{13}C NMR** (126 MHz, $CDCl_3$) δ 155.2, 151.8, 149.1, 140.9, 123.0 (t, J = 279.5 Hz), 120.6 – 107.7 (m), 107.0, 106.9 – 105.8 (m), 46.9, 32.0, 29.9, 28.1, 26.0, 25.6 (t, J = 28.2 Hz). **^{19}F NMR** (282 MHz, $CDCl_3$) δ: -70.6 to -72.5 (m, 2F), -81.2 (t, J = 9.9 Hz, 3F), -84.1 to -84.2 (m, 2F), -122.7 (br, 2F), -123.4 (br, 2F), -126.1 (br, 2F), -126.6 to -126.7 (m, 2F). **IR (KBr)**: 3459, 3115, 2952, 2871, 1705, 1671, 1242, 1205, 746, 713, 507 cm^{-1}.

(4-chlorophenyl)(3-(3,3-difluoro-2-iodo-3-((perfluorohexyl)oxy)propyl)-5-methoxy-2-methyl-1*H*-indol-1-yl)methanone (3oa)

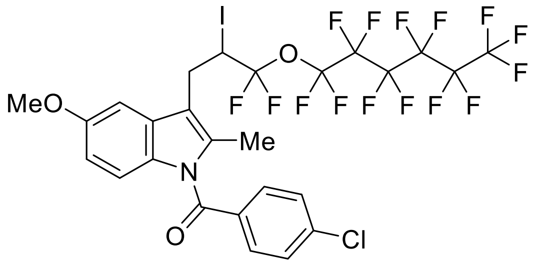

3oa (214.5 mg, 0.256 mmol, 85% yield) was obtained by **general procedure C** using fluoroalkene **1o** (112.7 mg, 0.30 mmol), 2,2,3,3,4,4,5,5,6,6,6-undecafluorohexanoyl fluoride **2a** (0.18 mL, 0.90 mmol), KF (52.4 mg, 0.90 mmol) and I_2 (222.3 mg, 0.88 mmol). The crude **3oa** was purified by flash chromatography on silica gel (Hexane/Ethyl Acetate = 8:2). Pale yellow solid. **Mp**. = 84.2 – 84.9 °C (Chloroform). **HRMS** (ESI) *m/z*: $[M + Na]^+$ calculated for $C_{26}H_{16}ClF_{15}INaO_3$ 859.9522; found: 859.9517. **^{1}H NMR** (500 MHz, $CDCl_3$) δ 7.68 – 7.65 (m, 2H), 7.50 – 7.47 (m, 2H), 6.94 (d -like, J = 9.2 Hz, 1H), 6.82 (d, J = 2.4 Hz, 1H), 6.71 (dd, J = 9.2, 2.4 Hz, 1H), 4.60 – 4.54 (m, 1H), 3.83 (s, 3H), 3.51 (dd, J = 15.4, 3.2 Hz, 1H), 3.23 (dd, J = 15.3, 11.3 Hz, 1H), 2.39 (s, 3H) . **^{13}C NMR** (126 MHz, $CDCl_3$) δ 168.5, 156.1, 139.6, 136.3, 133.8, 131.3 (2C), 131.1, 129.8, 129.3 (2C), 123.1 (t, J = 281.2 Hz), 118.4 – 115.6 (m), 115.4, 114.8, 114.4-112.2 (m), 111.6, 111.1 to 101.3 (m), 100.8, 55.7, 28.8, 25.7 (t, J = 27.3 Hz), 14.0. **^{19}F NMR** (282 MHz, $CDCl_3$) δ -70.8 to -72.3 (m, 2F), -81.2 (t-like, 3F), -84.0 to -84.1 (m, 2F), -122.6 (br, 2F), -123.3 (br, 2F), -126.1 (br, 2F), -126.6 to -126.7 (m, 2F). **IR (KBr)**: 2960, 2337, 1684, 1477, 1250, 1211, 1039, 926, 835, 711, 419 cm^{-1}.

4-((6,6-difluoro-5-iodo-6-((perfluorohexyl)oxy)hexyl)thio)quinazoline (3pa)

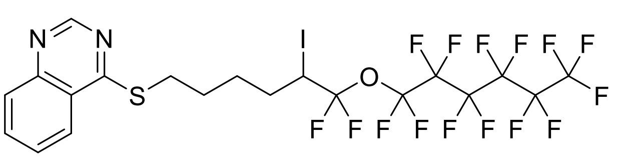

3pa (193.0 mg, 0.260 mmol, 85% yield) was obtained by **general procedure C** using fluoroalkene **1p** (84.1 mg, 0.30 mmol), 2,2,3,3,4,4,5,5,6,6,6-undecafluorohexanoyl fluoride **2a** (0.18 mL, 0.90 mmol), KF (52.7 mg, 0.90 mmol) and I_2 (228 mg, 0.90 mmol). The crude **3ra** was purified by flash chromatography on silica gel (Hexane/Et_2O = 98:2). White solid. **Mp.** = 32.9 – 33.6 °C (Chloroform). **HRMS** (ESI) *m*/*z*: $[M + H]^+$ calculated for $C_{20}H_{15}F_{15}NNaO_3$ 684.0206; found: 684.0218. ^{1}**H NMR** (500 MHz, $CDCl_3$) δ 8.98 (s, 1H), 8.09 (d, *J* = 8.5 Hz, 1H), 7.96 (d-like , *J* = 8.2 Hz, 1H), 7.87 – 7.84 (m, 1H), 7.61 – 7.57 (m, 1H), 4.27 – 4.21 (m, 1H), 3.49 – 3.34 (m, 2H), 1.97 – 1.56 (m, 7H). ^{13}**C NMR** (126 MHz, $CDCl_3$) δ 171.3, 153.6, 148.1, 133.8, 128.9, 127.5, 124.1, 124.0, 123.1 (t, *J* = 279.5 Hz), 120.6 – 106.2 (m), 32.1, 28.9, 28.3, 28.1, 25.9 (t, *J* = 28.2 Hz). ^{19}**F NMR** (282 MHz, $CDCl_3$) δ -70.6 to -72.2 (m, 2F), -81.3 (t, *J* = 9.9 Hz, 3F), -84.1 to -84.2 (m, 2F), -122.7 (br, 2F), -123.4 (br, 2F), -126.1 (br, 2F), -126.6 to -126.7 (m, 2F). **IR (KBr)**: 3470, 2944, 2860, 2334, 1747, 1485, 1243, 1207, 841, 711, 461 cm^{-1}.

(3*R*,5*R*,8*R*,9*S*,10*S*,13*R*,14*S*,17*R*)-17-((2*R*)-6,6-difluoro-5-iodo-6-((perfluorohexyl)oxy)hexan-2-yl)-3-methoxy-10,13-dimethylhexadecahydro-1*H*-cyclopenta[*a*]phenanthrene (3qa)

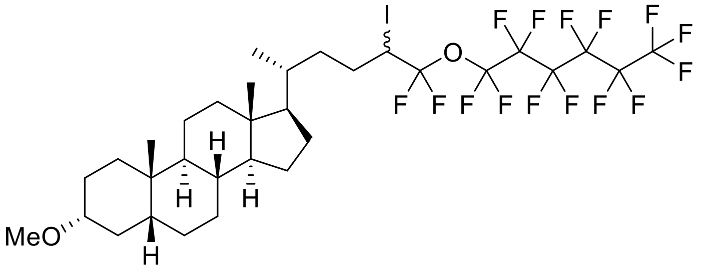

3qa (245.4 mg, 0.282 mmol, 94% yield, 1:1.2 *dr*) was obtained by **general procedure C** using fluoroalkene **1q** (122.8 mg, 0.30 mmol), 2,2,3,3,4,4,5,5,6,6,6-undecafluorohexanoyl fluoride **2a** (0.18 mL, 0.90 mmol), KF (52.6 mg, 0.91 mmol) and I_2 (230 mg, 0.91 mmol). The crude **3qa** was purified by flash chromatography on silica gel (Hexane/Ethyl Acetate = 95:5). Yellow oil. **HRMS** (ESI) *m*/*z*: $[M + H]^+$ calculated for $C_{32}H_{43}F_{15}IO_2$ 871.2068; found: 871.2042. ^{1}**H NMR** (500 MHz, $CDCl_3$) δ 4.14 – 4.08 (m, 1H), 3.35 (s, 3H), 3.19 – 3.13 (m, 1H), 2.00 – 0.96 (m, 29H), 0.91 (t, *J* = 9.5 Hz, 6H), 0.65 (d-like, *J* = 2.4 Hz, 3H). ^{13}**C NMR** (126 MHz, $CDCl_3$) δ 129.2, 128.4, 123.2 (t, *J* = 279.5 Hz), 123.2 (t, *J* = 279.5 Hz, rotamer), 120.7 – 104.6 (m), 80.5, 56.6, 56.6, 56.1, 56.0, 55.7, 42.9, 42.2, 40.4, 40.3, 40.2, 36.0, 35.7, 35.5, 35.4, 35.0, 34.9, 34.7, 32.9, 30.1, 29.9, 29.2, 28.5, 28.3, 27.6, 27.5, 27.3, 27.3, 27.1, 27.1, 26.9, 26.9, 26.5, 24.3, 23.6, 20.9, 19.0, 18.2, 12.2. ^{19}**F NMR** (282 MHz, $CDCl_3$) δ: -70.9- to -71.0 (m, 2F-rotamer), -71.3 to -71.4 (m, 2F), -81.2 (t-like, 3F), -84.1 to -84.2 (m, 2F), -122.7 (br, 2F), -123.4 (br, 2F), -126.1 (br, 2F), -126.6 to -126.7 (m, 2F). **IR (KBr)**: 3286, 2935, 2866, 2527, 1452, 1242, 1211, 1045, 712, 420 cm^{-1}.

2-(1-(6,6-difluoro-5-iodo-6-((perfluorohexyl)oxy)hexyl)-2,6-dioxopiperidin-3-yl)isoindoline-1,3-dione (3ra)

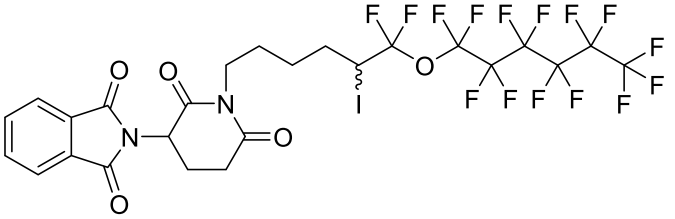

3ra (228 mg, 0.272 mmol, 91% yield) was obtained by **general procedure C** using fluoroalkene **1r** (112 mg, 0.30 mmol), 2,2,3,3,4,4,5,5,6,6,6-undecafluorohexanoyl fluoride **2a** (0.18 mL, 0.89 mmol), KF (51.6 mg, 0.89 mmol) and I_2 (226 mg, 0.89 mmol). The crude **3ra** was purified by flash chromatography on silica gel (Hexane/Ethyl Acetate = 2:1). Pale yellow oil. **HRMS** (ESI) *m*/*z*: $[M + Na]^+$ calculated for $C_{25}H_{18}F_{15}IN_2NaO_5$ 860.1919; found: 860.9896. ^{1}**H NMR** (500 MHz, $CDCl_3$) δ 7.90 – 7.88 (m, 2H), 7.79 – 7.76 (m, 2H), 5.03 – 4.95 (m, 1H), 4.21 – 4.13 (m, 1H), 3.88 – 3.78 (m, 2H), 3.03 – 2.93 (m, 1H), 2.64 – 2.88 (2H), 2.17 – 2.09 (m, 1H), 1.93 – 1.77 (m, 2H), 1.69 – 1.54 (m, 2H), 1.44 – 1.35 (m, 1H). ^{13}**C NMR** (126 MHz, $CDCl_3$) δ 171.0, 168.6, 167.5 (2C), 134.6 (2C), 131.9 (2C), 123.9 (2C), 123.1 (t, *J* = 280.4 Hz), 120.7 – 106.1 (m), 72.0, 70.8, 68.3, 67.5, 59.2, 50.2, 40.2, 40.1, 32.2, 32.1, 26.7, 26.6, 26.5, 26.3, 26.2, 26.2, 26.0, 26.0, 25.8, 22.2, 22.2. ^{19}**F NMR** (282 MHz, $CDCl_3$) δ: -70.6 to -72.5 (m, 2F), -81.2 (t-like, 3F), -84.1 (t-like, 2F), -122.7 (br, 2F), -123.4 (br, 2F), -126.1 (br, 2F), -126.6 to -126.7 (m, 2F). **IR (KBr)**:3850, 3646, 3286, 3032, 2926, 2358, 1715, 1680, 1390, 722, 506, 411 cm^{-1}.

5. Bromo-perfluoroalkoxylation of *gem*-difluoroalkenes 1

5-1. General Procedure D for Bromo-perfluoroalkoxylatyion

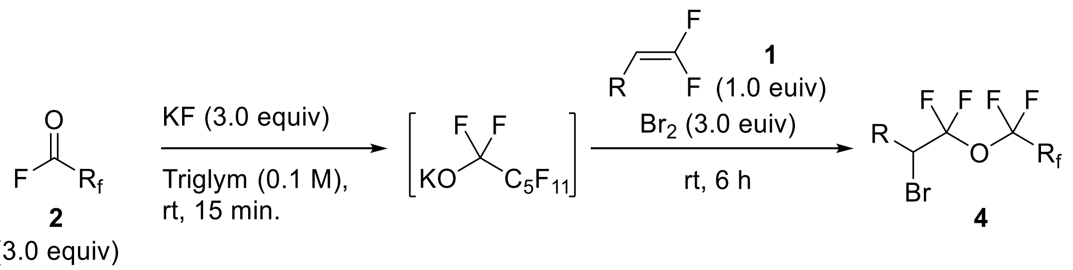

In an nitrogene gas-filled glovebox, KF (0.9 mmol, 3.0 eq), triglym (2.7 mL), and perfluoroacyl fluoride (0.9 mmol, 3.0 eq) was added to a 20 mL round-bottom flask equipped with a magnetic stir bar. After stirring for 15 min at room temptrture, fluoroalkene (0.3 mmol, 1.0 eq) was added, followed by one-portion addition of Br_2 (0.9 mmol, 3.0 eq) in 0.3 mL triglym and the mixture was stirred at room tempereture for 6 h. The reaction mixture was quenched with saturated $NaHCO_3$aq and Na_2SO_3 aq. The product was extracted with Et_2O (10 mL x 3) and washed with Brine. The organic phase was dried over anhydrous Na_2SO_4 and concentrated under reduced pressure. The product was purified by column chromatography on silica gel using hexane or hexane/Ethyl Acetate as eluent.

5-2. General Procedure E for Bromo-perfluoroalkoxylatyion

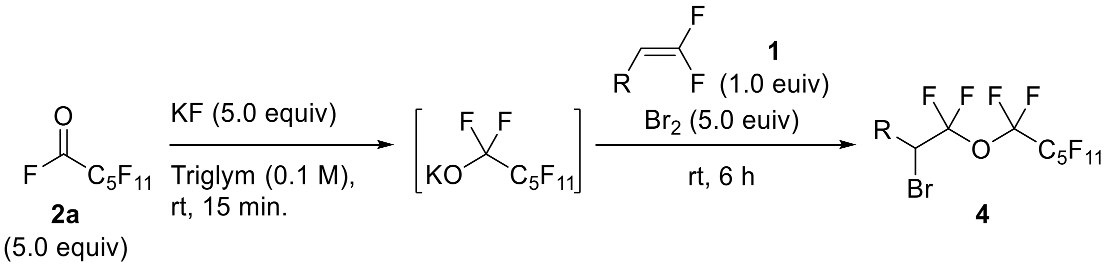

In an nitrogene gas-filled glovebox, KF (1.5 mmol, 5.0 eq), triglym (5.5 mL), and perfluoroacyl fluoride (1.5 mmol, 5.0 eq) was added to a 20 mL round-bottom flask equipped with a magnetic stir bar. After stirring for 15 min at room temptrture, fluoroalkene (0.3 mmol, 1.0 eq) was added, followed by one-portion addition of Br_2 (1.5 mmol, 5.0 eq) in 0.5 mL triglym and the mixture was stirred at room tempereture for 6 h. The reaction mixture was quenched with saturated $NaHCO_3$ aq and Na_2SO_3 aq. The product was extracted with Et_2O (10 mL x 3) and washed with Brine. The organic phase was dried over anhydrous Na_2SO_4 and concentrated under reduced pressure. The product was purified by column chromatography on silica gel using hexane or hexane/Ethyl Acetate as eluent.

(3-bromo-4,4-difluoro-4-((perfluorohexyl)oxy)butyl)benzene (4aa)

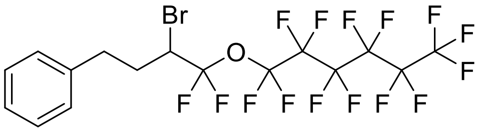

4aa (154.5 mg, 0.265 mmol, 88% yield) was obtained by **general procedure D** using fluoroalkene **1a** (50.5 mg, 0.30 mmol), 2,2,3,3,4,4,5,5,6,6,6-undecafluorohexanoyl fluoride **2a** (0.18 mL, 0.90 mmol), KF (53.0 mg, 0.91 mmol) and Br_2 (0.90 mmol). The crude **4aa** was purified by flash chromatography on silica gel (Hexane). Colorless oil. **HRMS** (EI) *m/z*: $[M]^+$ calculated for $C_{16}H_{10}BrF_{15}O$ 581.9676; found: 581.9677. **^{1}H NMR** (500 MHz, $CDCl_3$) δ 7.34 – 7.31 (m, 2H), 7.26 – 7.23 (m, 1H), 7.21 – 7.20 (m, 2H), 3.98 – 3.92 (m, 1H), 3.04 – 2.98 (m, 1H), 2.78 – 2.72 (m, 1H), 2.37 – 2.30 (m, 1H), 2.19 – 2.11 (m, 1H). **^{13}C NMR** (126 MHz, $CDCl_3$) δ 139.2, 128.9 (2C), 128.6 (2C), 126.9, 125.0 (t, *J* = 280.2 Hz), 120.7 – 107.9 (m), 48.4 (t, *J* =30.0 Hz), 32.9, 32.6. **^{19}F NMR** (282 MHz, $CDCl_3$) δ: -74.7 to -74.8 (m, 2F), -81.2 to -81.3 (m, 3F), -84.1 (br, 2F), -122.8 (br, 2F), -123.4 (br, 2F), -126.2 (br, 2F), -126.6 to -126.7 (m, 2F). **IR (KBr)**: 3800, 3285, 3037, 2959, 2339, 1605, 1499, 1241, 1208, 746, 646 cm^{-1}.

1-(((5-bromo-6,6-difluoro-6-((perfluorohexyl)oxy)hexyl)oxy)methyl)-4-methoxybenzene (4ca)

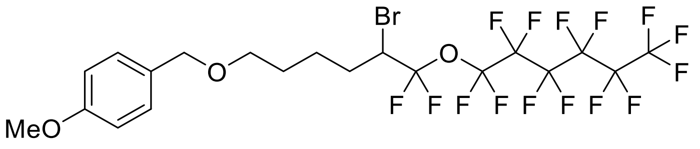

4ca (186.5 mg, 0.278 mmol, 93% yield) was obtained by **general procedure D** using fluoroalkene **1c** (76.9 mg, 0.30 mmol), 2,2,3,3,4,4,5,5,6,6,6-undecafluorohexanoyl fluoride **2a** (0.18 mL, 0.90 mmol), KF (52.3 mg, 0.90 mmol) and Br_2 (0.90 mmol). The crude **4ca** was purified by flash chromatography on silica gel (Hexane). Colorless oil. **HRMS** (FAB) *m*/*z*: $[M]^+$ calculated for $C_{20}H_{18}BrF_{15}O_3$ 670.0200; found: 670.0197. ^{1}**H NMR** (500 MHz, $CDCl_3$) δ 7.29 – 7.28 (m, 2H), 6.92 – 6.90 (m, 2H), 4.46 (s, 2H), 4.12 – 4.07 (m, 1H), 3.83 (s, 3H), 3.49 (t, *J* = 6.3 Hz, 2H), 2.09 – 2.03 (m, 1H), 1.90 – 1.49 (m, 6H). ^{13}**C NMR** (126 MHz, $CDCl_3$) δ 159.3, 130.5, 129.4 (2C), 122.7 (t, *J* = 280.4 Hz), 118.6 – 115.3 (m), 113.9 (2C), 113.3 – 106.2 (m), 72.8, 69.4, 55.4, 49.3 (t, *J* = 30.0 Hz), 31.1, 28.9, 24.0. ^{19}**F NMR** (282 MHz, $CDCl_3$) δ -74.2 to -75.5 (m, 2F), -81.2 (t-like, 3F), -83.9 to -84.0 (m, 2F), -122.7 (br, 2F), -123.3 (br, 2F), -126.0 (br, 2F), -126.5 to -126.6 (m, 2F). **IR (KBr)**: 3431, 2939, 2863, 2337, 1744, 1500, 1244, 1208, 810, 710, 417 cm^{-1}.

5-bromo-6,6-difluoro-6-((perfluorohexyl)oxy)hexyl 4-methylbenzenesulfonate (4ea)

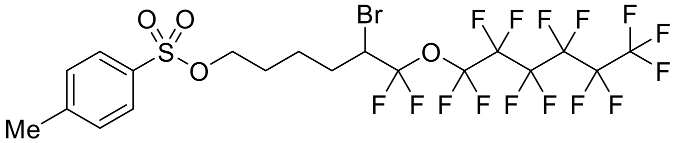

4ea (190.9 mg, 0.271 mmol, 90% yield) was obtained by **general procedure D** using fluoroalkene **1e** (87.1 mg, 0.30 mmol), 2,2,3,3,4,4,5,5,6,6,6-undecafluorohexanoyl fluoride **2a** (0.18 mL, 0.90 mmol), KF (52.3 mg, 0.90 mmol) and Br_2 (0.9 mmol). The crude **4ea** was purified by flash chromatography on silica gel (Hexane/Ethyl Acetate = 19:1). Colorless oil. **HRMS** (ESI) *m*/*z*: $[M + Na]^+$ calculated for $C_{19}H_{16}BrF_{15}NaO_4S$ 726.9611; found: 726.9612. ^{1}**H NMR** (500 MHz, $CDCl_3$) δ 7.80 (d-like, *J* = 8.2 Hz, 2H), 7.36 (d-like, J = 8.2 Hz, 2H), 4.05 (t, *J* = 5.5 Hz, 2H (-OCH_2-) and, m, 1H (-CHBr-) are overlapped), 2.45 (s, 3H), 2.01 – 1.95 (m, 1H), 1.81 – 1.64 (m, 4H), 1.51 – 1.41 (m, 1H). ^{13}**C NMR** (126 MHz, $CDCl_3$) δ 145.1, 133.0, 130.0 (2C), 128.0 (2C), 122.5 (t, *J* = 280.4 Hz), 118.6 – 105.5 (m), 69.8, 48.8 (t, *J* =30.0 Hz), 30.7, 27.9, 23.2, 21.7. ^{19}**F NMR** (282 MHz, $CDCl_3$) δ -74.3 to -75.7 (m, 2F), -81.2 (t, *J* = 9.9 Hz, 3F), -84.0 to -84.2 (m, 2F), -122.8 (br, 2F), -123.4 (br, 2F), -126.1 (br, 2F), -126.6 to -126.7 (m, 2F). **IR (KBr)**: 3541, 3286, 2962, 2351, 1732, 1599, 1362, 1245, 1208, 813, 436 cm^{-1}.

(1-bromo-2,2-difluoro-2-((perfluorohexyl)oxy)ethyl)benzene (4ha)

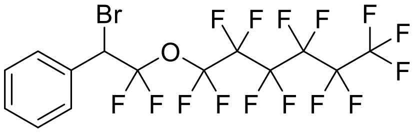

4ha (66.7 mg, 0.120 mmol, 40% yield) was obtained by **general procedure D** using fluoroalkene **1h** (42.0 mg, 0.30 mmol), 2,2,3,3,4,4,5,5,6,6,6-undecafluorohexanoyl fluoride **2a** (0.18 mL, 0.90 mmol), KF (52.4 mg, 0.90 mmol) and Br_2 (0.90 mmol). The crude **4ha** was purified by flash chromatography on silica gel (Hexane). Colorless oil. **HRMS** (FAB) *m*/*z*: $[M]^+$ calculated for $C_{14}H_6BrF_{15}O$ 553.9363; found: 553.9374. ^{1}**H NMR** (500 MHz, $CDCl_3$) δ 7.48 – 7.47 (m, 2H), 7.42 – 7.35 (m, 3H), 5.12 (t, *J* = 8.4 Hz, 1H). ^{13}**C NMR** (126 MHz, $CDCl_3$) δ 132.6, 130.2, 129.4 (2C), 128.9 (2C), 122.1 (t, *J* = 281.2 Hz), 118.6 – 106.2 (m), 48.9 (t, *J* = 30.0 Hz). ^{19}**F NMR** (282 MHz, $CDCl_3$) δ -73.0 to -74.4 (m, 2F), -81.3 to -81.2 (t, *J* = 9.0 Hz, 3F), -83.4 to -84.7 (m, 2F), -122.8 (br, 2F), -123.5 (br, 2F), -126.0 to -126.1 (m, 2F), -126.6 to -126.7 (m. 2F). **IR (KBr)**: 3733, 3565, 2928, 2351, 1460, 1244, 1208, 849, 697, 649 cm^{-1}.

1-(1-bromo-2,2-difluoro-2-((perfluorohexyl)oxy)ethyl)-4-methylbenzene (4ia)

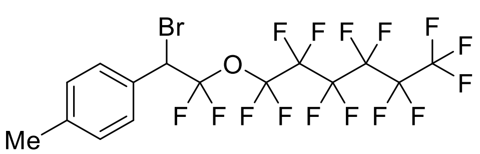

4ia (91.3 mg, 0.160 mmol, 53% yield) was obtained by **general procedure D** using fluoroalkene **1i** (46.4 mg, 0.30 mmol), 2,2,3,3,4,4,5,5,6,6,6-undecafluorohexanoyl fluoride **2a** (0.18 mL, 0.90 mmol), KF (52.4 mg, 0.90 mmol) and Br_2 (0.90 mmol). The crude **4ia** was purified by flash chromatography on silica gel (Hexane).

Colorless oil. **HRMS** (FAB) *m/z*: $[M]^+$ calculated for $C_{15}H_8BrF_{15}O$ 567.9519; found: 567.9508. **^{1}H NMR** (500 MHz, $CDCl_3$) δ 7.36 (d-like, *J* = 7.9 Hz, 2H), 7.18 (d-like, *J* = 7.9 Hz, 2H), 5.10 (t, *J* = 8.5 Hz, 1H), 2.36 (s, 3H). **^{13}C NMR** (126 MHz, $CDCl_3$) δ 140.4, 129.7, 129.6 (2C), 129.2 (2C), 128.1 (m), 121.8 (t, *J* = 246,6 Hz), 121.0 – 120.5 (m), 119.8-106.0 (m), 48.9 (t, *J* = 30.1 Hz), 21.3. **^{19}F NMR** (282 MHz, $CDCl_3$) δ -73.1 to -74.3 (m, 2F), -81.4 to -81.1 (t, *J* = 9.0 Hz, 3F), -83.4 to -84.6 (m, 2F), -122.8 (br, 2F), -123.4 (br, 2F), -126.0 to -126.1 (m, 2F), -126.6 to -126.7 (m. 2F). **IR (KBr)**: 3748, 3044, 2355, 1517, 1247, 1209, 857, 713, 648, 505 cm^{-1}.

1-(1-bromo-2,2-difluoro-2-((perfluorohexyl)oxy)ethyl)-4-(trifluoromethyl)benzene (4ja)

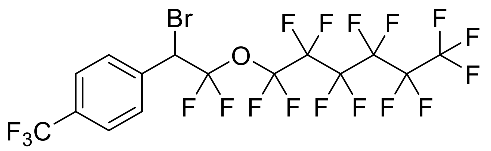

4ja (124.3 mg, 0.199 mmol, 66% yield) was obtained by **general procedure D** using fluoroalkene **1j** (61.9 mg, 0.30 mmol), 2,2,3,3,4,4,5,5,6,6,6-undecafluorohexanoyl fluoride **2a** (0.18 mL, 0.90 mmol), KF (52.4 mg, 0.90 mmol) and Br_2 (0.90 mmol). The crude **4ja** was purified by flash chromatography on silica gel (Hexane). Colorless oil. **HRMS** (FAB) *m/z*: $[M + H]^+$ calculated for $C_{15}H_6BrF_{18}O$ 622.9315; found: 622.9320. **^{1}H NMR** (500 MHz, $CDCl_3$) δ 7.67 – 7.65 (m, 2H), 7.63 – 7.61 (m, 2H), 5.19 – 5.15 (m, 1H). **^{13}C NMR** (126 MHz, $CDCl_3$) δ 136.4, 132.5 (q, *J* = 32.8 Hz), 129.9 (2C), 125.9 (d-like, *J* = 3.7 Hz, 2C), 124.7 (q, *J* = 273.0 Hz), 121.8 (t, *J* = 281,2 Hz), 118.6 – 105.8 (m), 47.7 (t, *J* =31.0 Hz). **^{19}F NMR** (282 MHz, $CDCl_3$) δ -63.6 (s, 3F), -72.8 to -74.4 (m, 2F), -81.2 to -81.3 (t-like, 3F), -83.3 to -84.7 (m, 2F), -122.8 (br, 2F), -123.5 (br, 2F), -126.0 (br, 2F), -126.6 to -126.7 (m. 2F). **IR (KBr)**: 3540, 3035, 2778, 2122, 1625, 1423, 1245, 1142, 1020, 644, 505 cm^{-1}.

5-(3-bromo-4,4-difluoro-2-methyl-4-((perfluorohexyl)oxy)butyl)benzo[*d*][1,3]dioxole (4ka)

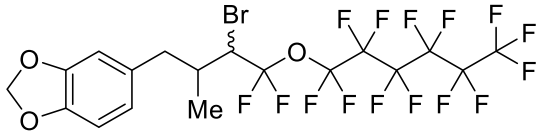

4ka (168.5 mg, 0.263 mmol, 88% yield, 1.2:1 *dr*) was obtained by **general procedure E** using fluoroalkene **1k** (67.9 mg, 0.30 mmol), 2,2,3,3,4,4,5,5,6,6,6-undecafluorohexanoyl fluoride **2a** (0.30 mL, 1.5 mmol), KF (87.2 mg, 1.5 mmol) and Br_2 (1.5 mmol). The crude **4ka** was purified by flash chromatography on silica gel (Hexane/Et_2O = 99:1). Colorless oil. **HRMS** (FAB) *m/z*: $[M]^+$ calculated for $C_{18}H_{12}BrF_{15}O_3$ 639.9730; found: 639.9726. **^{1}H NMR** (500 MHz, $CDCl_3$) δ 7.02 (s, 1H), 6.74 (s, 1H), 5.99 – 5.98 (m, 2H), 4.09 – 4.05 (m, 1H), 2.75 to 2.70 (m, 1H), 2.65 – 2.61 (m, 1H), 2.51 – 2.45 (m, 1H), 1.07 (d, *J* = 6.7 Hz, 3H). **^{13}C NMR** (126 MHz, $CDCl_3$) δ 147.6, 147.5, 130.7, 122.7 (t, *J* = 264.9 Hz), 118.6 – 115.6 (m), 115.1, 113.3, 113.0 – 112.1 (m), 111.0, 110.8 – 106.7 (m), 54.7 (t, *J* = 27.2 Hz), 41.9, 33.3, 15.0. **^{19}F NMR** (282 MHz, $CDCl_3$) δ -69.8 to -72.4 (m, 2F), -81.2 (t-like, 3F), -83.8 to -84.0 (m, 2F), -122.6 (br, 2F), -123.3 (br, 2F), -126.0 (br, 2F), -126.5 to -126.7 (m, 2F). **IR (KBr)**: 2978, 2903, 1747, 1507, 1481, 1335, 1235, 1208, 938, 647 cm^{-1}.

tert-butyl 4-(1-bromo-2,2-difluoro-2-((perfluorohexyl)oxy)ethyl)piperidine-1-carboxylate (4la)

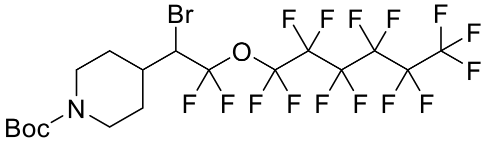

4la (152.9 mg, 0.231 mmol, 82% yield) was obtained by **general procedure E** using fluoroalkene **1l** (70.0 mg, 0.28 mmol), 2,2,3,3,4,4,5,5,6,6,6-undecafluorohexanoyl fluoride **2a** (0.30 mL, 1.5 mmol), KF (87.2 mg, 1.5 mmol) and Br_2 (1.5 mmol).The crude **4la** was purified by flash chromatography on silica gel (Hexane/Ethyl Acetate = 9:1). Yellow solid. **Mp.** = 51.6 – 52.3 °C (Chloroform). **HRMS** (ESI) *m/z*: $[M + Na]^+$ calculated for $C_{18}H_{19}BrF_{15}NNaO_3$ 684.0206; found: 684.0218. **^{1}H NMR** (500 MHz, $CDCl_3$) δ 4.22 – 4.08 (br, 2H, (-CH_2-) and, td, *J* = 9.0, 4.0 Hz, 1H (-CH-) are overlapped), 2.71 (br, 2H), 2.02 – 1.96 (m, 1H), 1.77 – 1.76 (m, 1H), 1.59 – 1.49 (m, 3H), 1.46 (s, 9H). **^{13}C NMR** (126 MHz, $CDCl_3$) δ 154.7, 122.5 (t, *J* =282.1 Hz), 118.6 – 106.2 (m), 79.9, 54.7 (t, *J* =28.2 Hz), 43.4, 42.9, 37.2, 30.4, 28.5 (3C), 27.6. **^{19}F NMR** (282 MHz, $CDCl_3$) δ -70.3 (br, 2F), -81.2 (t, *J* = 8.7 Hz, 3F), -83.2 to -84.5 (m, 2F), -122.7 (br, 2F), -123.3 (br, 2F),

-126.0 (br, 2F), -126.5 to -126.6 (m, 2F). **IR (KBr)**: 3592, 3411, 2988, 2939, 2337, 1683, 1413, 1243, 1210, 775, 647 cm^{-1}.

2-(3-bromo-3,4,4-trifluoro-4-((perfluorohexyl)oxy)butyl)isoindoline-1,3-dione (4ma)

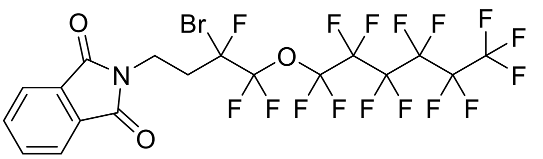

(a) **4ma** (184.7 mg, 0.276 mmol, 92% yield) was obtained by **general procedure E** using fluoroalkene **1m** (76.6 mg, 0.30 mmol), 2,2,3,3,4,4,5,5,6,6,6-undecafluorohexanoyl fluoride **2a** (0.30 mL, 1.5 mmol), KF (88.0 mg, 1.51 mmol) and Br_2 (1.5 mmol). The crude **4ma** was purified by flash chromatography on silica gel (Hexane/Ethyl Acetate = 19:1). (b) **4ma** (154.5 mg, 0.23 mmol, 85% yield) was obtained by **general procedure D** using fluoroalkene **1v** (70.0 mg, 0.27 mmol), 2,2,3,3,4,4,5,5,6,6,6-undecafluorohexanoyl fluoride **2a** (0.18 mL, 0.9 mmol), KF (52.2 mg, 0.9 mmol) and Br_2 (0.9 mmol). The crude **4ma** was purified by flash chromatography on silica gel (Hexane/Ethyl Acetate = 19:1).

White solid. **Mp**. = 75.4 – 76.0 °C (Chloroform). **HRMS** (FAB) *m/z*: $[M + H]^+$ calculated for $C_{18}H_9BrF_{16}NO_3$ 669.9510; found: 669.9517. **^{1}H NMR** (500 MHz, $CDCl_3$) δ 7.90 – 7.86 (m, 2H), 7.77 – 7.74 (m, 2H), 4.13 (t, *J* = 7.3 Hz, 2H), 2.77 – 2.70 (m, 1H), 2.60 – 2.48 (m, 1H). **^{13}C NMR** (126 MHz, $CDCl_3$) δ 167.9 (2C), 134.4 (2C), 132.0 (2C), 123.6 (2C), 121.6-106.1 (m), 100.6 (dt, *J* = 264.9, 34.7 Hz), 35.4 (d, *J* = 20.0 Hz), 33.3. **^{19}F NMR** (282 MHz, $CDCl_3$) δ -81.1 (t, *J* = 9.9 Hz, 3F), -82.7 to -83.4 (m, 2F), -83.7 to -83.8 (m, 2F), -122.5 (br, 2F), -123.1 (br, 2F), -125.8 (br, 2F), -126.4 to -126.5 (m, 2F), -127.4 to -127.6 (m, 1F). **IR (KBr)**: 3732, 3287, 2958, 2312, 1778, 1715, 1212, 1148, 649, 530, 409 cm^{-1}.

2-(3-bromo-4,4-difluoro-4-((perfluorohexyl)oxy)butyl)isoindoline-1,3-dione (4sa)

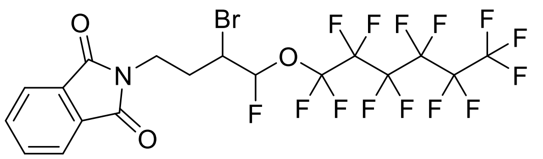

4sa (157.4 mg, 0.248 mmol, 51% yield) was obtained by **general procedure D** using fluoroalkene **1s** (107 mg, 0.49 mmol), 2,2,3,3,4,4,5,5,6,6,6-undecafluorohexanoyl fluoride **2a** (0.30 mL, 1.5 mmol), KF (87.2 mg, 1.5 mmol) and Br_2 (1.5 mmol). The crude **4sa** was purified by flash chromatography on silica gel (Hexane/Ethyl Acetate = 19:1). White solid. **Mp**. = 46.4 – 46.9 °C (Chloroform). **HRMS** (FAB) *m/z*: $[M + H]^+$ calculated for $C_{18}H_{11}BrF_{14}NO_3$633.9699; found: 633.9708. **^{1}H NMR** (500 MHz, $CDCl_3$) δ 7.87 – 7.86 (m, 2H), 7.75 – 7.74 (m, 2H), 6.04 (dd, *J* = 56.3, 4.4 Hz, 1H), 4.08 – 4.04 (m, 1H), 4.00 – 3.89 (m, 2H), 2.48 – 2.42 (m, 1H), 2.23 – 2.15 (m, 1H). **^{13}C NMR** (126 MHz, $CDCl_3$) δ 168.3 (2C), 134.3 (2C), 132.0 (2C), 123.6 (2C), 120.9 – 103.3 (m), 105.2 (dt, *J* = 240.3, 3.7 Hz), 46.9 (d, *J* = 24.5 MHz), 35.7, 30.9. **^{19}F NMR** (282 MHz, $CDCl_3$) δ -81.2 (t, *J* = 9.7 Hz, 3F), -83.3 to -83.8 (m, 1F), -85.5 to -86.0 (m, 1F), -122.6 (br, 2F), -123.2 (br, 2F), -125.8 (br, 2F), -126.5 to -126.6 (m, 2F), -128.0 to -128.3 (m, 1F) **IR (KBr)**: 3475, 2952, 1945, 1778, 1617, 1356, 1281, 1081, 964, 855, 776 cm^{-1}.

2-(3-bromo-4,4-difluoro-4-((perfluorohexyl)oxy)butyl)isoindoline-1,3-dione (4ta)

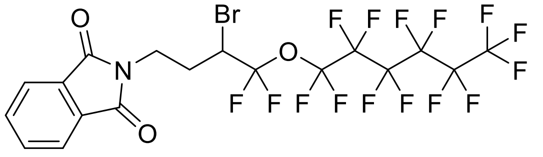

4ta (182.8 mg, 0.280 mmol, 93% yield) was obtained by **general procedure D** using fluoroalkene **1t** (71.0 mg, 0.30 mmol), 2,2,3,3,4,4,5,5,6,6,6-undecafluorohexanoyl fluoride **2a** (0.18 mL, 0.9 mmol), KF (52.4 mg, 0.9 mmol) and Br_2 (0.9 mmol). The crude **4ta** was purified by flash chromatography on silica gel (Hexane/Ethyl Acetate = 19:1). White solid. **Mp**. = 47.3 – 47.6 °C (Chloroform). **HRMS** (FAB) *m/z*: $[M + H]^+$ calculated for $C_{18}H_{10}BrF_{15}NO_3$ 651.9605; found: 651.9597. **^{1}H NMR** (500 MHz, $CDCl_3$) δ 7.89 – 7.87 (m, 2H), 7.76 – 7.75 (m, 2H), 4.20 – 4.14 (m, 1H), 4.01 – 3.89 (m, 2H), 2.54 – 2.47 (m, 1H), 2.25 – 2.18 (m, 1H). **^{13}C NMR** (126 MHz, $CDCl_3$) δ 168.2 (2C), 134.4 (2C), 132.0 (2C), 123.6 (2C), 122.4 (t, *J* = 281.2 Hz) 120.9 – 105.9 (m), 46.1 (t, J = 30.0 MHz), 30.9. **^{19}F NMR** (282 MHz, $CDCl_3$) δ -74.4 to -75.8 (m, 2F), -81.2 (t, *J* = 9.7 Hz, 3F), -83.9 to -84.0 (m, 2F), -122.6 (br, 2F), -123.3 (br, 2F), -126.0 (br, 2F), -126.5 to -126.6 (m, 2F). **IR (KBr)**: 3856, 3615, 2967, 1617, 1470, 1442, 1006, 989, 850, 776 cm^{-1}.

6. Bromo-perfluoroalkoxylation (C3 to C5) of *gem*-difluoroalkenes 1

6-1. General Procedure F for Bromo-perfluoroalkoxylatyion

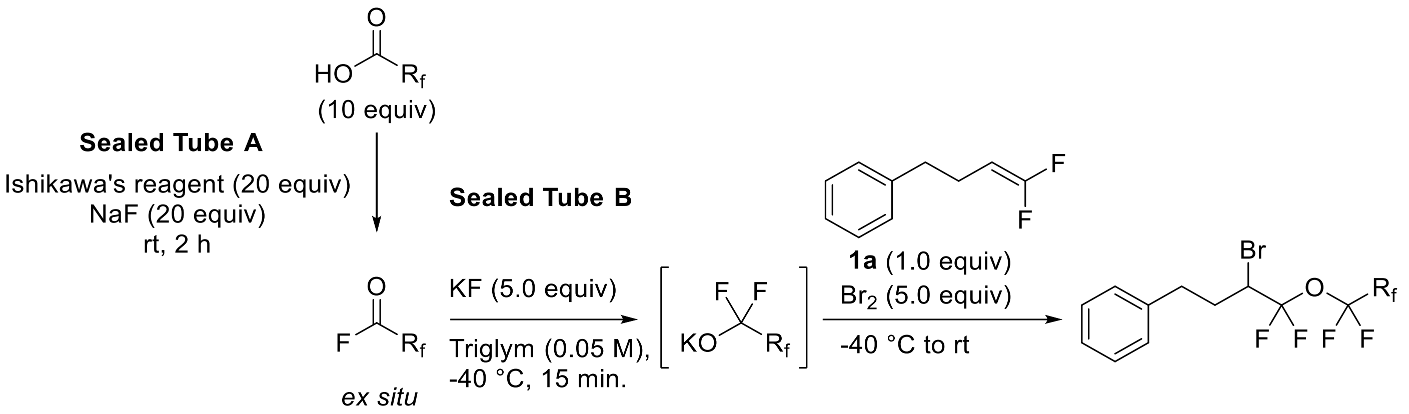

To a 15-mL oven-dried sealed tube (A) equipped with a magnetic stir bar was added NaF (252 mg, 6.0 mmol, 20 equiv) in the glove box. Tube (A) was removed out of glove box and Ishikawa's Reagent (1.1 mL, 6.0 mmol, 20 equiv) was added under N_2. To another 15-mL oven-dried sealed tube (B) equipped with a magnetic stir bar were added KF (5.0 equiv) and 5.0 mL dry triglym in the glove box and Tube (B) was removed out of glove box. Tube (A) was placed into liquid N_2, and perfluorocarboxylic acid (3.0 mmol, 10 equiv) was added until the solution was frozen. Tube (A) was sealed. The mixture was warmed to room temperature and stirred for 2 h. Tube (B) was evacuated for 1-2 seconds before it was connected with tube (A) through a gas-tube and tube (A) was stirred at rt while tube (B) was cooled to -78 °C. The R_fCOF was transferred from tube (A) to tube (B) for 15 min. Then the Tube (B) was sealed again, warmed up to -40 °C. After stirring for 15 min at -40 °C, fluoroalkene **1a** (0.3 mmol, 1.0 eq) was added, followed by one-portion addition of Br_2 (1.5 mmol, 5.0 eq) in 1.0 mL triglym and the mixture was warmed up to rt and stirred at room tempereture for 6 h. The reaction mixture was quenched with saturated $NaHCO_3$ aq and Na_2SO_3 aq. The product was extracted with Et_2O (10 mL x 3) and washed with Brine. The organic phase was dried over anhydrous Na_2SO_4 and concentrated under reduced pressure. The product was purified by column chromatography on silica gel using hexane as eluent.

(3-bromo-4,4-difluoro-4-(perfluoropropoxy)butyl)benzene (4ab)

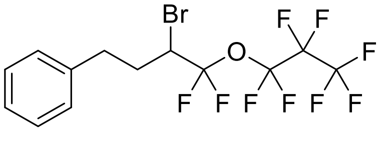

4ab (98.9 mg, 0.228 mmol, 76% yield) was obtained by **general procedure F** using fluoroalkene **1a** (51.2 mg, 0.30 mmol), 2,2,3,3,3-pentafluoropropanoyl fluoride (exess), KF (87.2 mg, 1.5 mmol) and Br_2 (1.5 mmol). The crude **4ab** was purified by flash chromatography on silica gel (Hexane). Colorless oil. **HRMS** (FAB) *m/z*: $[M]^+$ calculated for $C_{13}H_{10}BrF_9O$ 431.9771; found: 431.9781. ^{1}**H NMR** (500 MHz, $CDCl_3$) δ 7.34 – 7.20 (m, 5H), 3.98 – 3.92 (m, 1H), 3.03 – 2.98 (m, 1H), 2.78 – 2.72 (m, 1H), 2.36 – 2.29 (m, 1H), 2.18 – 2.11 (m, 1H). ^{13}**C NMR** (126 MHz, $CDCl_3$) δ 139.2, 128.9 (2C), 128.6 (2C), 126.9, 122.7 (t, J = 280.4 Hz), 121.0 – 120.7 (m), 118.7 – 104.1 (m), 48.5 (t, J = 30.0 Hz), 32.9, 32.6. ^{19}**F NMR** (282 MHz, $CDCl_3$) δ -74.6 to -74.7 (m, 2F), -81.5 (t, J = 7.4 Hz, 3F), -85.2 to -85.3 (m, 2F), -130.4 (s, 2F). **IR (KBr)**: 3441, 3033, 2938, 2354, 1456, 1341, 1232, 1071, 755, 504 cm^{-1}.

(3-bromo-4,4-difluoro-4-(perfluorobutoxy)butyl)benzene (4ac)

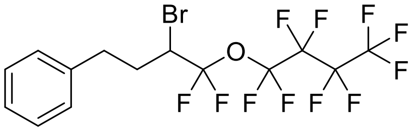

4ac (94.6 mg, 0.196 mmol, 65% yield) was obtained by **general procedure F** using fluoroalkene **1a** (50.5 mg, 0.30 mmol), 2,2,3,3,4,4,4-heptafluorobutanoyl fluoride (exess), KF (87.2 mg, 1.5 mmol) and Br_2 (1.5 mmol). The crude **4ac** was purified by flash chromatography on silica gel (Hexane). Colorless oil. **HRMS** (FAB) *m/z*: $[M]^+$ calculated for $C_{14}H_{10}BrF_{11}O$ 481.9739; found: 481.9745. ^{1}**H NMR** (500 MHz, $CDCl_3$) δ 7.35 – 7.32 (m, 2H), 7.27 – 7.21 (m, 3H), 3.99 – 3.93 (m, 1H), 3.04 – 2.99 (m, 1H), 2.79 – 2.73 (m, 1H), 2.37 – 2.30 (m, 1H), 2.19 – 2.11 (m, 1H). ^{13}**C NMR** (126 MHz, $CDCl_3$) δ 139.2, 128.9 (2C), 128.6 (2C), 126.9,

122.7 (t, J = 281.2 Hz), 118.7 – 105.3 (m), 48.4 (t, J = 29.1 Hz), 32.9, 32.6. **^{19}F NMR** (282 MHz, $CDCl_3$) δ -74.6 to -74.7 (m, 2F), -81.4 (t, J = 8.9 Hz, 3F), -84.3 (m, 2F), -126.8 (m, 2F), -127.0 (m, 2F). **IR (KBr)**: 3854, 3032, 2960, 2368, 1604, 1239, 1214, 903, 748, 698 cm^{-1}.

(3-bromo-4,4-difluoro-4-((perfluoropentyl)oxy)butyl)benzene (4ad)

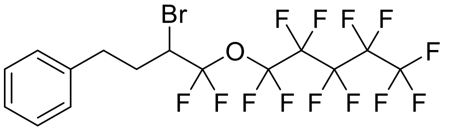

4ad (179.9 mg, 0.337 mmol, 99% yield) was obtained by **general procedure F** using fluoroalkene **1a** (57.1 mg, 0.34 mmol), 2,2,3,3,4,4,5,5,5-nonafluoropentanoyl fluoride (exess), KF (87.15 mg, 1.5 mmol) and Br_2 (1.5 mmol). The crude **4ad** was purified by flash chromatography on silica gel (Hexane). Colorless oil. **HRMS** (FAB) m/z: $[M]^+$ calculated for $C_{15}H_{10}BrF_{13}O$ 531.9707; found: 531.9704. **^{1}H NMR** (500 MHz, $CDCl_3$) δ 7.32 (t-like, J = 7.3 Hz, 2H), 7.26 – 7.20 (m, 3H), 3.98 – 3.92 (m, 1H), 3.03 – 2.98 (m, 1H), 2.78 – 2.72 (m, 1H), 2.36 – 2.30 (m, 1H), 2.19 – 2.11 (m, 1H). **^{13}C NMR** (126 MHz, $CDCl_3$) δ 139.2, 128.9 (2C), 128.6 (2C), 126.9, 122.7 (t, J = 267.5 Hz), 121.0 – 120.6 (m), 118.7 – 105.8 (m), 48.4 (t, J = 29.1 Hz), 32.9, 32.6. **^{19}F NMR** (282 MHz, $CDCl_3$) δ -74.7 to -74.8 (m, 2F), -81.2 (t, J = 9.9 Hz, 3F), -84.1(m, 2F), -123.5 to -123.6 (m, 2F), -126.3 (m, 2F), -126.7 to -126.8 (m, 2F). **IR (KBr)**: 3747, 3647, 3033, 2357, 1498, 1242, 1213, 735, 702, 413 cm^{-1}.

5-2. Procedure for G for Bromo-heptafluoroisopropoxylation

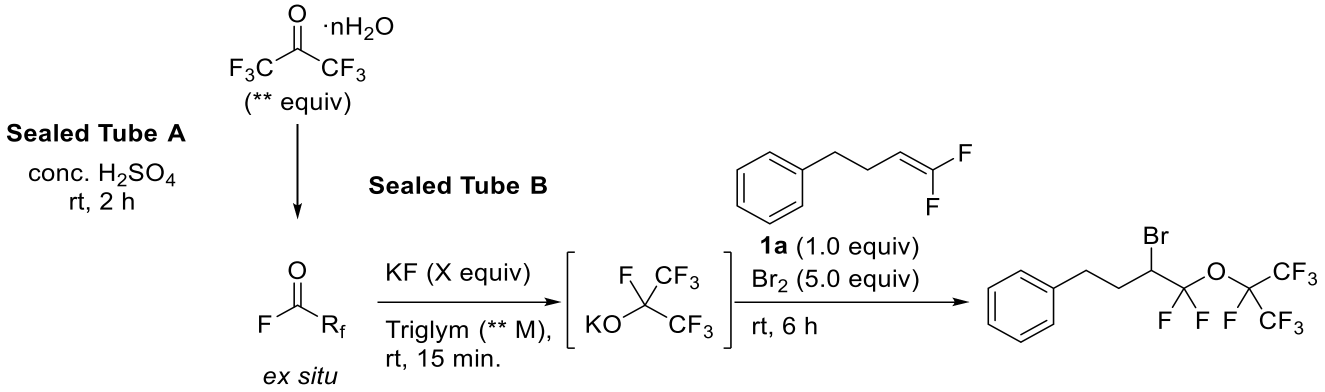

To a 15-mL oven-dried sealed tube (B) equipped with a magnetic stir bar were added KF (87.2 mg, 1.5 mmol, 5.0 equiv) and 5.0 mL dry triglym in the glove box and Tube (B) was removed out of glove box. To another 15-mL oven-dried sealed tube (A) equipped with a magnetic stir bar were added conc. H_2SO_4 (8.0 mL). Tube (A) was placed into liquid N_2, and Hexafluoroacetone Hydrate (0.4 mL) was added until the solution was frozen. Tube (A) was sealed. The mixture was warmed to 50 °C and stirred for 1 h. Tube (B) was evacuated for 1-2 seconds before it was connected with tube (A) through a gas-tube and tube (A) was stirred at 50 °C while tube (B) was cooled to -78 °C. The hexafluoroacetone was transferred from tube (A) to tube (B) for 15 min. Then the Tube (B) was sealed again, warmed up to -40 °C. After stirring for 15 min at -40 °C, fluoroalkene **1a** (50.5 mg, 0.30 mmol, 1.0 equiv) was added, followed by one-portion addition of Br_2 (1.5 mmol, 5.0 eq) in 1.0 mL triglym and the mixture was warmed up to rt and stirred at room tempereture for 6 h. The reaction mixture was quenched with saturated $NaHCO_3$ aq and Na_2SO_3 aq. The product was extracted with Et_2O (10 mL x 3) and washed with Brine. The organic phase was dried over anhydrous Na_2SO_4 and concentrated under reduced pressure. The crude product was purified by silica gel chromatography (Hexane) to obtain as colourless oil.

(3-bromo-4,4-difluoro-4-((perfluoropropan-2-yl)oxy)butyl)benzene (4ae)

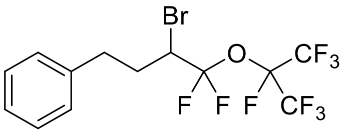

4ae (107.8 mg, 0.249 mmol, 83% yield). **HRMS** (FAB) m/z: $[M]^+$ calculated for $C_{13}H_{10}BrF_9O$ 431.9771; found: 431.9763. **^{1}H NMR** (500 MHz, $CDCl_3$) δ 7.34 – 7.31 (m, 2H), 7.26 – 7.20 (m, 3H), 4.01 – 3.95 (m, 1H), 3.03 – 2.97 (m, 1H), 2.78 – 2.72 (m, 1H), 2.37 – 2.31 (m, 1H), 2.20 – 2.12 (m, 1H). **^{13}C NMR** (126 MHz, $CDCl_3$) δ 139.3, 128.9 (2C), 128.7 (2C), 126.8, 122.6 (td, J = 279.3, 3.7 Hz), 119.0 (qd, J =

290.3, 31.8 Hz, 2C), 102.6 (dquin, J = 266.6, 38.3 Hz), 48.9 (t, J = 38.2 Hz), 33.1, 32.7. **^{19}F NMR** (282 MHz, $CDCl_3$) δ -72.1 to -73.5 (m, 2F), -80.7 (s, 6F), -146.3 (t, J = 22.8 Hz, 1F). **IR (KBr)**: 3853, 3465, 3028, 2933, 2350, 1249, 1139, 987, 701, 444 cm^{-1}.

7. Chloro-perfluoroalkoxylation of *gem*-difluoroalkenes 1

7-1. General Procedure H for Chloro-perfluoroalkoxylatyion

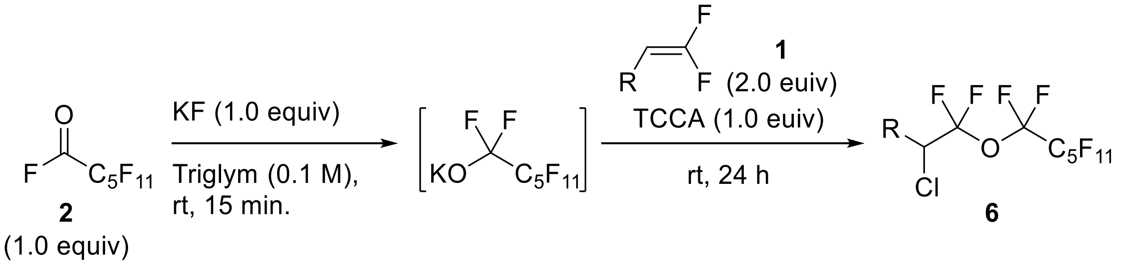

In an nitrogene gas-filled glovebox, KF (0.3 mmol, 1.0 eq), triglym (3.0 mL), and perfluoroacyl fluoride (0.3 mmol, 1.0 eq) was added to a 20 mL round-bottom flask equipped with a magnetic stir bar. After stirring for 15 min at room temptrture, fluoroalkene (0.6 mmol, 2.0 eq) was added, followed by one-portion addition of TCCA (0.3 mmol, 1.0 eq) and the mixture was stirred at room tempereture for 24 h. The reaction mixture was quenched with saturated $NaHCO_3$aq and Na_2SO_3 aq. The product was extracted with Et_2O (10 mL x 3) and washed with Brine. The organic phase was dried over anhydrous Na_2SO_4 and concentrated under reduced pressure. The product was purified by column chromatography on silica gel using hexane or hexane/Ethyl Acetate as eluent.

(3-chloro-4,4-difluoro-4-((perfluorohexyl)oxy)butyl)benzene (6aa)

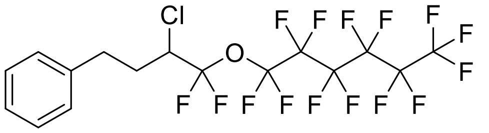

6aa (87.1 mg, 0.162 mmol, 53% yield) was obtained by **general procedure H** using fluoroalkene **1a** (100.9 mg, 0.60 mmol), 2,2,3,3,4,4,5,5,6,6,6-undecafluorohexanoyl fluoride **2a** (94.8 mg, 0.30 mmol), KF (17.4 mg, 0.30 mmol) and TCCA (69.7 mg, 0.30 mmol). The crude **6aa** was purified by flash chromatography on silica gel (Hexane). Colorless oil. **HRMS** (ESI) *m*/*z*: [M + H]$^+$ calculated for $C_{16}H_{11}OF_{15}Cl$ 539.0259; found: 539.0234. **^{1}H NMR** (500 MHz, $CDCl_3$) δ 7.34-7.31 (m, 2H), 7.26-7.23 (m, 1H), 7.21-7.19 (m, 2H), 3.98-3.93 (m, 1H), 3.02-2.97 (m, 1H), 2.79-2.73 (m, 1H), 2.32-2.25 (m, 1H), 2.12-2.05 (m, 1H). **^{13}C NMR** (126 MHz, $CDCl_3$) δ 139.3, 128.9 (2C), 128.6 (2C), 126.8, 122.9 (t, J = 280.6 Hz), 121.0-105.9 (m, 6C), 58.1 (t, J = 30.0 Hz), 32.5, 31.5. **^{19}F NMR** (282 MHz, $CDCl_3$) δ -77.2 to -77.3 (m, 2F), -81.2 (t, J = 9.9 Hz, 3F), -83.8 (br, 2F), -122.7 (br, 2F), -123.2 to -123.3 (m, 2F), -126.0 (br, 2F), -126.5 to -126.6 (m, 2F). **IR (KBr)**: 3567, 3032, 2363, 1605, 1498, 1339, 1201, 954, 777, 556 cm^{-1}.

4-(((4-chloro-5,5-difluoro-5-((perfluorohexyl)oxy)pentyl)oxy)methyl)-1,1'-biphenyl (6ba)

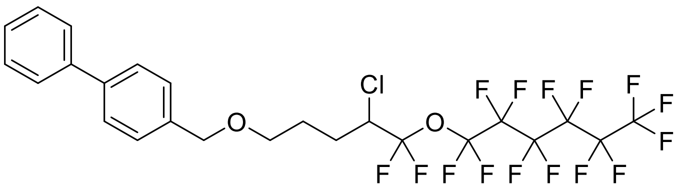

6ba (82.5 mg, 0.120 mmol, 40% yield) was obtained by **general procedure H** using fluoroalkene **1b** (173 mg, 0.60 mmol), 2,2,3,3,4,4,5,5,6,6,6-undecafluorohexanoyl fluoride **2a** (94.8 mg, 0.30 mmol), KF (17.4 mg, 0.3 mmol) and TCCA (69.7 mg, 0.3 mmol). The crude **6ba** was purified by flash chromatography on silica gel (Hexane). Colorless oil. **HRMS** (ESI) *m*/*z*: [M + Na]$^+$ calculated for $C_{24}H_{18}O_2F_{15}NaCl$ 681.0654; found: 681.0630. **^{1}H NMR** (500 MHz, $CDCl_3$) δ 7.59-7.57 (m, 4H), 7.45-7.33 (m, 5H), 4.55 (s, 2H), 4.19-4.13 (m, 1H), 3.59-3.51 (m, 2H), 2.23-2.17 (m, 1H), 2.00-1.93 (m, 1H), 1.90-1.75 (m, 2H). **^{13}C NMR** (126 MHz, $CDCl_3$) δ 141.0, 140.8, 137.3, 128.9 (2C), 128.2 (2C), 127.4, 127.3 (2C), 127.2 (2C), 122.9 (t, J = 280.6 Hz),δ 118.7-105.9 (m, 6C),δ 72.9, 69.0, 59.1 (t, J = 30.4 Hz), 28.2, 26.0. **^{19}F NMR** (282 MHz, $CDCl_3$) δ -76.8 to -78.1 (m, 2F), -81.2 (t, J = 9.9 Hz, 3F), -83.8 to -84.0 (m, 2F), -122.7 (br, 2F), -123.3 (br, 2F), -126.0 (br, 2F), -126.5 to -126.6 (m, 2F). **IR (KBr)**: 3430, 3060, 2933, 2862, 2345, 1782, 1488, 1238, 844, 698 cm^{-1}.

5-chloro-6,6-difluoro-6-((perfluorohexyl)oxy)hexyl benzoate (6da)

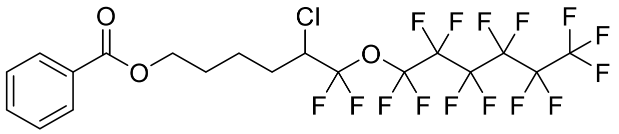

6da (97.7 mg, 0.160 mmol, 53% yield) was obtained by **general procedure H** using fluoroalkene **1d** (144.2 mg, 0.60 mmol), 2,2,3,3,4,4,5,5,6,6,6-undecafluorohexanoyl fluoride **2a** (94.8 mg, 0.30 mmol), KF (17.4 mg, 0.3 mmol) and TCCA (69.7 mg, 0.3 mmol). The crude **6da** was purified by flash chromatography on silica gel (Hexane/EtOAc = 98:2). Colorless oil. **HRMS** (ESI) *m/z*: $[M + Na]^+$ calculated for $C_{19}H_{14}O_3F_{15}NaCl$ 633.0290; found: 633.0290. **^{1}H NMR** (500 MHz, $CDCl_3$) δ 8.05-8.03 (m, 2H), 7.58-7.55 (m, 1H), 7.46-7.43 (m, 2H), 4.39-4.32 (m, 2H), 4.12-4.07 (m, 1H), 2.10-2.04 (m, 1H), 1.90-1.77 (m, 4H), 1.67-1.56 (m, 1H). **^{13}C NMR** (126 MHz, $CDCl_3$) δ 166.7, 133.1, 130.3, 129.7 (2C), 128.5 (2C), 122.8 (t, J = 281.1 Hz), δ 120.7-106.2 (m, 6C), 64.4, 59.0 (t, J = 30.4 Hz), 30.7, 28.1, 22.7. **^{19}F NMR** (282 MHz, $CDCl_3$) δ -76.7 to -78.0 (m, 2F), -81.2 (t, J = 9.9 Hz, 3F), -83.8 to -83.9 (m, 2F), -122.7 (br, 2F), -123.2 (br, 2F), -126.0 (br, 2F), -126.4 to -126.6 (m, 2F). **IR (KBr)**: 3446, 3073, 2962, 2365, 1968, 1722, 1610, 1463, 1252, 777 cm^{-1}.

5-chloro-6,6-difluoro-6-((perfluorohexyl)oxy)hexyl 4-methylbenzenesulfonate (6ea)

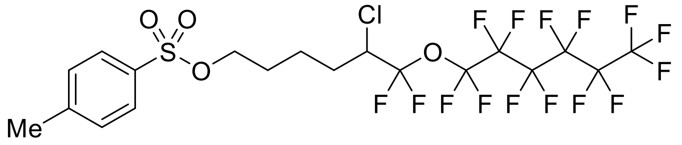

6ea (91.0 mg, 0.140 mmol, 46% yield) was obtained by **general procedure H** using fluoroalkene **1e** (174.2 mg, 0.60 mmol), 2,2,3,3,4,4,5,5,6,6,6-undecafluorohexanoyl fluoride **2a** (94.8 mg, 0.30 mmol), KF (17.4 mg, 0.3 mmol) and TCCA (69.7 mg, 0.3 mmol). The crude **6ea** was purified by flash chromatography on silica gel (Hexane:EtOAc = 95:5). Colorless oil. **HRMS** (ESI) *m/z*: $[M + Na]^+$ calculated for $C_{19}H_{16}O_4F_{15}NaSCl$ 683.0116; found: 683.0129. **^{1}H NMR** (500 MHz, $CDCl_3$) δ 7.80-7.79 (m, 2H), 7.36-7.35 (m, 2H), 4.09-3.97 (m, 3H), 2.45 (s, 3H), 1.96-1.89 (m, 1H), 1.75-1.65 (m, 4H), 1.51-1.42 (m, 1H). **^{13}C NMR** (126 MHz, $CDCl_3$) δ 145.1, 133.1, 130.0 (2C), 128.0 (2C), 122.7 (t, J = 281.1 Hz), δ 120.4-105.9 (m, 6C), 69.8, 58.8 (t, J = 30.4 Hz), 30.3, 28.2, 22.0, 21.7. **^{19}F NMR** (282 MHz, $CDCl_3$) δ -76.8 to -78.2 (m, 2F), -81.2 (t, J = 10.0 Hz, 3F), -83.8 to -83.9 (m, 2F), -122.7 (br, 2F), -123.2 (br, 2F), -126.0 (br, 2F), -126.5 to -126.5 (m, 2F). **IR (KBr)**: 3467, 2962, 2588, 2365, 1924, 1665, 1599, 1460, 967, 531 cm^{-1}..

2-(3-chloro-4,4-difluoro-4-((perfluorohexyl)oxy)butyl)isoindoline-1,3-dione (6ta)

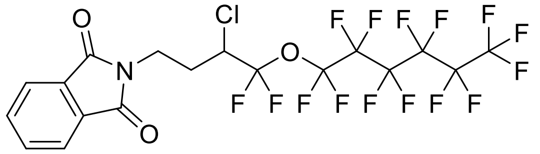

6ta (104 mg, 0.171 mmol, 57% yield) was obtained by **general procedure H** using fluoroalkene **1t** (142.3 mg, 0.60 mmol), 2,2,3,3,4,4,5,5,6,6,6-undecafluorohexanoyl fluoride **2a** (94.8 mg, 0.30 mmol), KF (17.4 mg, 0.3 mmol) and TCCA (69.7 mg, 0.3 mmol). The crude **6ta** was purified by flash chromatography on silica gel (Hexane/Et_2O = 9:1). Colorless oil. **HRMS** (ESI) *m/z*: $[M + Na]^+$ calculated for $C_{18}H_9NO_3F_{15}NaCl$ 629.9929; found: 629.9918. **^{1}H NMR** (500 MHz, $CDCl_3$) δ 7.89-7.84 (m, 2H), 7.78-7.70 (m, 2H), 4.22-4.16 (m, 1H), 4.01-3.90 (m, 2H), 2.47-2.41 (m, 1H), 2.18-2.11 (m, 1H) δ 7.89-7.84 (m, 2H), 7.78-7.70 (m, 2H), 4.22-4.16 (m, 1H), 4.01-3.90 (m, 2H), 2.47-2.41 (m, 1H), 2.18-2.11 (m, 1H). **^{13}C NMR** (126 MHz, $CDCl_3$) δ 168.2 (2C), 134.4 (2C), 132.0 (2C), 123.6 (2C), δ 123.6 (t, J = 140.3 Hz), 120.9-107.7 (m, 6C), 56.8 (t, J = 30.9 Hz), 34.6, 30.4. **^{19}F NMR** (282 MHz, $CDCl_3$) δ -77.6 (ddtd, J = 219.8, 140.7, 13.5, 6.5 Hz, 2F), -81.2 (t, J = 9.9 Hz, 3F), -83.8 to -83.9 (m, 2F), -122.7 (br, 2F), -123.3 (br, 2F), -126.0 (br, 2F), -126.5 to -126.6 (m, 2F). **IR (KBr)**: 3464, 3227, 3066, 2926, 2696, 2403, 1738, 1228, 851, 571 cm^{-1}.

8. Synthetic application of compound 3aa

8-1. Procedure for preparation of 1-((1,1-difluoro-1-((perfluorohexyl)oxy)-4-phenylbutan-2-yl)oxy)-2,2,6,6-tetramethylpiperidine (7)

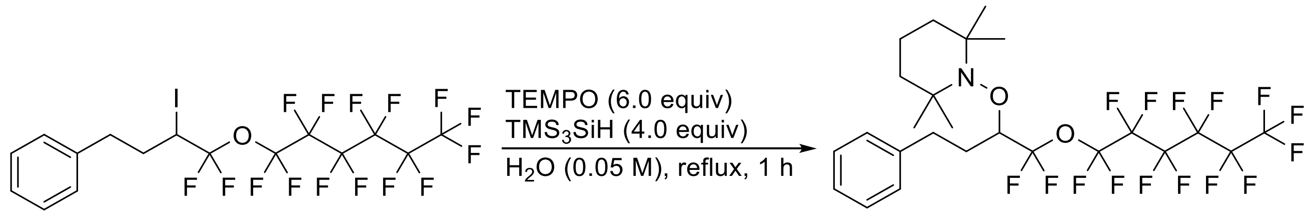

In a round bottom flask, (4,4-difluoro-3-iodo-4-((perfluorohexyl)oxy)butyl)benzene (**3aa**: 1.0 equiv, 504.1 mg, 0.80 mmol) and TEMPO (750.0 mg, 4.80 mmol, 6.0 equiv) was dissolved in degassed H_2O (0.05 M). Then, the solution was heated under reflux. Tris(trimethylsilyl)silane (TMS_3SiH: 795.7 mg, 3.20 mmol, 4.0 equiv.) was added in three portions every 60 min. After the last addition of TMS_3SiH, the mixture was heated under reflux for another 1 h. After completion, the solution was diluted with CH_2Cl_2. The mixture was extracted with CH_2Cl_2 3 times and washed with Brine. The organic phase was dried over anhydrous Na_2SO_4 and concentrated under reduced pressure. The crude product was purified by silica gel chromatography (Hexane) to obtain the compounds **7** as colourless oil (381.9 mg, 0.567 mmol, 71% yield).

HRMS (FAB) *m/z*: $[M + H]^+$ calculated for $C_{25}H_{29}F_{15}NO_2$ 660.1959; found: 660.1972. **^{1}H NMR** (500 MHz, $CDCl_3$) δ 7.31 – 7.28 (m, 2H), 7.22 – 7.19 (m, 3H), 4.29 – 4.24 (m, 1H), 2.85 – 2.79 (m, 1H), 2.75 – 2.69 (m, 1H), 2.60 – 2.53 (m, 1H), 2.00 – 1.92 (m, 1H), 1.59 – 1.26 (m, 6H), 1.13 (d, J = 17.1 Hz, 6H), 1.06 (d, J = 18.6 Hz, 6H). **^{13}C NMR** (126 MHz, $CDCl_3$) δ 141.4, 128.6 (2C), 128.5 (2C), 126.2, 124.2 (t, J = 284.9 Hz), 120.7 – 106.0 (m), 80.7 (t, J = 25.5 Hz), 61.4, 60.1, 40.7, 40.5, 33.7, 32.2, 29.4 (2C), 20.3 (2C), 17.2. **^{19}F NMR** (282 MHz, $CDCl_3$) δ -75.8 to -77.6 (m, 2F), -81.3 (t, J = 9.9 Hz, 3F), -83.4 to -83.6 (m, 2F), -122.6 to -122.7 (m, 2F), -123.4 (br, 2F), −126.1 (m, 2F), −126.6 to −126.7 (m, 2F). **IR (KBr)**: 3650, 3423, 2941, 2311, 1456, 1244, 1209, 741, 711, 407 cm^{-1}.

8-2. Procedure for preparation of 1,1-difluoro-1-((perfluorohexyl)oxy)-4-phenylbutan-2-ol (8)

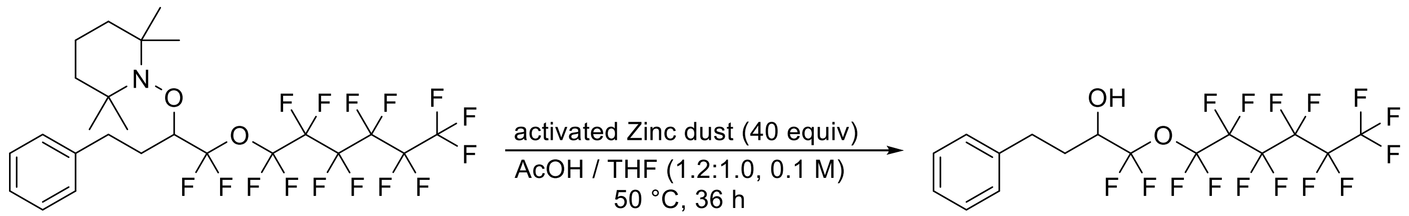

In a round bottom flask, 1-((1,1-difluoro-1-((perfluorohexyl)oxy)-4-phenylbutan-2-yl)oxy)-2,2,6,6-tetramethylpiperidine (**7**: 1.0 equiv, 134.7 mg, 0.200 mmol) was dissolved in acetic acid/THF (1.2:1, 0.1 M). Activated Zinc dust (520 mg, 7.95 mmol, 39.8 equiv) was added, and the reaction mixture was stirred at 50 ˚C for 36 h. After completion, the mixture was cooled to rt, and diluted with Et_2O. The solution was filtered through a plug of silica, which was subsequently washed with Ethyl Acetate. The solvent was removed under reduced pressure. The crude product was purified by silica gel chromatography (Hexane/Ethyl Acetate = 96:4) to obtain the compounds **8**. The characterization data of the compound **7** as yellow oil (103.5 mg, 0.198 mmol, 99% yield).

HRMS (FAB) *m/z*: $[M]^+$ calculated for $C_{16}H_{11}F_{15}O_2$ 520.0520; found: 520.0510. **^{1}H NMR** (500 MHz, $CDCl_3$) δ 7.33 – 7.30 (m, 2H), 7.24 – 7.20 (m, 3H), 3.90 – 3.84 (m, 1H), 2.96 – 2.91 (m, 1H), 2.77 – 2.71 (m, 1H), 2.15 (d, J = 6.1 Hz, 1H), 2.04 – 1.98 (m, 1H), 1.95 – 1.88 (m, 1H). **^{13}C NMR** (126 MHz, $CDCl_3$) δ 140.4, 128.8 (2C), 128.6 (2C), 126.5, 123.8 (t, J = 283.1 Hz), 116.1 – 108.3 (m), 70.9 (t, J = 28.2 Hz), 31.0, 30.8. **^{19}F NMR** (282 MHz, $CDCl_3$) δ -81.2 (t, J = 9.9 Hz, 3F), -83.1 to -83.3 (m, 2F), -83.6 to -83.7 (m, 2F), -122.8 (br, 2F), -123.3 to -123.4 (m, 2F), -126.1 to -126.1 (m, 2F), -126.5 to -126.7 (m, 2F). **IR (KBr)**: 3868, 3444, 2966, 2317, 1714, 1249, 1207, 804, 700, 531 cm^{-1}.

8-3. Procedure for preparation of (4,4-difluoro-4-((perfluorohexyl)oxy)butyl)benzene (9)

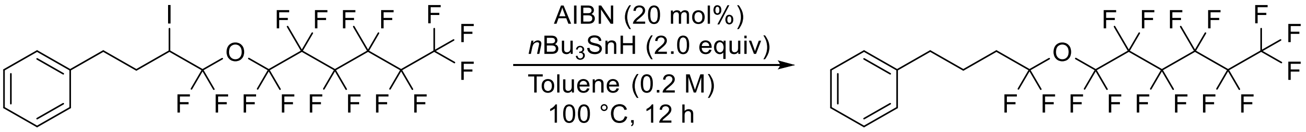

3aa **9**

In a round bottom flask, (4,4-difluoro-3-iodo-4-((perfluorohexyl)oxy)butyl)benzene (**3aa**: 189.0 mg, 0.3 mmol, 1.0 equiv) was dissolved in Toluene (0.2 M). AIBN (9.9 g, 0.06 mmol, 20 mol%), and *n*Bu_3SnH (0.161 mL, 0.6 mmol, 2.0 equiv.) were added to the solution. Then, the reaction mixture was warmed up to 100 °C and stirred at 100 °C for 12 h. After completion, the solution was quenched with KF (5%) aq., and extracted with Et_2O. The organic layer was washed with brine, and dried over Na_2SO_4. The solvent was removed under reduced pressure. The crude product was purified by silica gel chromatography (Hexane) to obtain the compound **9** as colourless oil (141.2 mg, 0.28 mmol, 93% yield).

HRMS (FAB) *m/z*: $[M]^+$ calculated for $C_{16}H_{11}F_{15}O$ 504.0570; found: 504.0562. **^{1}H NMR** (500 MHz, $CDCl_3$) δ: 7.32 – 7.29 (m, 2H), 7.25 – 7.21 (m, 1H), 7.18 – 7.16 (m, 2H), 2.69 (t, J = 7.6 Hz, 2H), 2.16 – 2.08 (m, 2H), 1.92 – 1.86 (m, 2H). **^{13}C NMR** (126 MHz, $CDCl_3$) δ: 140.7, 128.8 (2C), 128.5 (2C), 126.5, 125.8 (t, J = 278.5 Hz), 121.0 – 106.0 (m), 35.3 (t, J = 25.5 Hz), 34.7, 23.6. **^{19}F NMR** (282 MHz, $CDCl_3$) δ -69.7 to -69.8 (m, 2F), -81.3 (t, J = 9.4 Hz, 3F), -83.8 to -83.9 (m, 2F), -122.9 (br, 2F), -123.4 (br, 2F), -126.3 (br, 2F), -126.6 to -126.7 (m, 2F). **IR (KBr)**: 3459, 2955, 2873, 2339, 1457, 1246, 1006, 745, 530, 425 cm^{-1}.

8-4. Procedure for preparation of (3-(difluoro((perfluorohexyl)oxy)methyl)hex-5-en-1-yl)benzene (10)

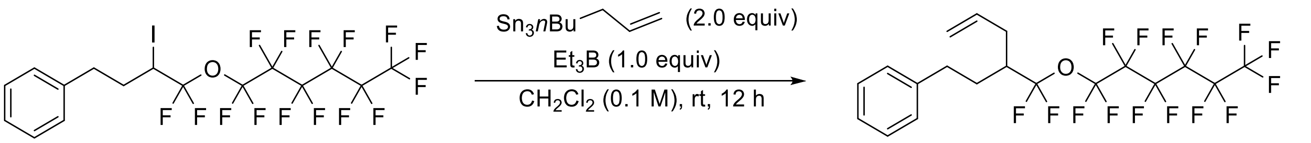

3aa **10**

In a round bottom flask, (4,4-difluoro-3-iodo-4-((perfluorohexyl)oxy)butyl)benzene (**3aa**: 189.0 mg, 0.30 mmol, 1.0 equiv) was dissolved in CH_2Cl_2 (0.1 M). Allyltributyltin (198.7 mg, 0.60 mmol, 2.0 equiv), and Triethylborane (29.4 mg, 0.30 mmol, 1.0 equiv.) were added to the solution. Then, the reaction mixture was stirred at rt for 12 h. After completion, the solution was quenched with KF (5%) aq., and extracted with Et_2O. The organic layer was washed with brine, and dried over Na_2SO_4. The solvent was removed under reduced pressure. The crude product was purified by silica gel chromatography (Hexane) to obtain the compound **10** as colourless oil (126.5 mg, 0.232 mmol, 77% yield).

HRMS (FAB) *m/z*: $[M]^+$ calculated for $C_{19}H_{15}F_{15}O$ 544.0883; found: 544.0894. **^{1}H NMR** (500 MHz, $CDCl_3$) δ 7.31 – 7.28 (m, 2H), 7.22 – 7.19 (m, 1H), 7.18 – 7.15 (m, 2H), 5.77 – 5.69 (m, 1H), 5.15 – 5.10 (m, 2H), 2.75 – 2.65 (m, 2H), 2.46 – 2.40 (m, 1H), 2.27 – 2.13 (m, 2H), 1.96 – 1.88 (m, 1H), 1.82 – 1.74 (m, 1H). **^{13}C NMR** (126 MHz, $CDCl_3$) δ 141.1, 134.2, 128.7(2C), 128.5 (2C), 127.0 (t, J = 282.1 Hz), 126.3, 118.1, 116.1-108.2 (m), 43.5 (t, J = 21.8 Hz), 32.9, 32.3, 29.1. **^{19}F NMR** (282 MHz, $CDCl_3$) δ -72.2 to -72.4 (m, 2F), -80.8 (br, 3F), -83.3 (br, 2F), -122.3 (br, 2F), -122.9 (br, 2F), -125.7 (br, 2F), -126.2 (br, 2F). **IR (KBr)**: 3929, 3450, 3032, 2937, 2324, 1645, 1244, 1206, 746, 701 cm^{-1}.

8-5. Procedure for preparation of (*E*)-1-(3-(difluoro((perfluorohexyl)oxy)methyl)-5-phenylpent-1-en-1-yl)pyrrolidin-2-one (11)

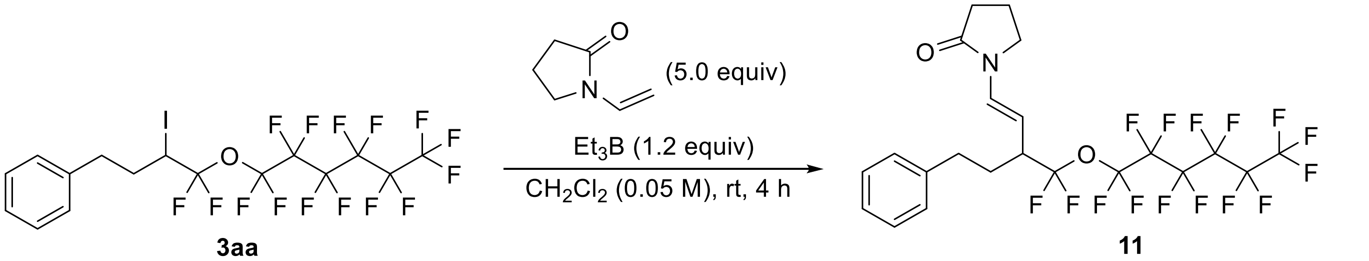

To a solution of (4,4-difluoro-3-iodo-4-((perfluorohexyl)oxy)butyl)benzene (**3aa**: 189.0 mg, 0.30 mmol) and 1-vinylpyrrolidin-2-one (166.7 mg, 1.50 mmol, 5.0 equiv) in CH_2Cl_2 (0.05 M) was added Triethylborane (1.0 M in Hexane, 0.36 ml, 0.36 mmol, 1.2 equiv.) while the needle was immersed in the solution. The resulting solution was stirred open to air under $CaCl_2$ guard tube for 4 h at rt. After completion, the solvent was removed under reduced pressure. The crude product was purified by silica gel chromatography (Hexane/Ethyl Acetate = 4.5:1) to obtain the compound **11** as white solid (105.7 mg, 0.173 mmol, 58% yield).

Mp. = 69.7 – 70.3 °C (Chloroform). **HRMS** (FAB) *m/z*: $[M + H]^+$ calculated for $C_{22}H_{19}F_{15}NO_2$ 614.1176; found: 614.1186. **^{1}H NMR** (500 MHz, $CDCl_3$) δ: 7.31 – 7.28 (m, 2H), 7.22 – 7.20 (m, 1H), 7.15 – 7.14 (m, 2H), 7.05 (d, *J* = 14.3 Hz, 1H), 4.61 (dd, *J* = 14.3, 9.5 Hz, 1H), 3.55 – 3.46 (m, 2H), 2.76 – 2.66 (m, 2H), 2.57 – 2.51 (m, 3H), 2.18 – 2.08 (m, 3H), 1.79 – 1.71 (m, 1H). **^{13}C NMR** (126 MHz, $CDCl_3$) δ: 173.5, 140.7, 129.3, 128.7 (2C), 128.5 (2C), 126.4, 125.7 (t, *J* = 282.1 Hz), 121.0 – 106.2 (m), 103.9, 47.0 (t, *J* = 24.6 Hz), 45.1, 32.6, 31.2, 29.7, 17.5. **^{19}F NMR** (282 MHz, $CDCl_3$) δ -74.3 to -74.5 (m, 2F), -81.2 (t-like, *J* = 9.9 Hz, 3F), -83.8 (m, 2F), -122.8 (br, 2F), -123.3 to -123.4 (m, 2F), -126.2 (m, 2F), -126.5 to -126.6 (m, 2F). **IR (KBr)**: 3901, 3384, 2928, 2319, 1689, 1199, 1105, 954, 714, 422 cm^{-1}.

8-6. Procedure for preparation of (*E*)-(4,4-difluoro-4-((perfluorohexyl)oxy)but-2-en-1-yl)benzene (12)

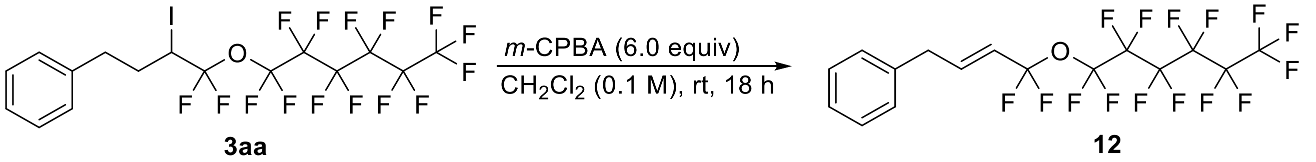

In a round bottom flask, (4,4-difluoro-3-iodo-4-((perfluorohexyl)oxy)butyl)benzene (**3aa**: 189.0 mg, 0.30 mmol, 1.0 equiv) was dissolved in CH_2Cl_2 (0.1 M). The solution was cooled to 0 °C, then *m*-CPBA (3-Chloroperoxybenzoic Acid, 30% in water, 477.7 mg, 1.8 mmol, 6.0 equiv) was added to the reaction mixture. The reaction mixture was stirred at rt for 18 h. After completion, the solution was quenched with $NaHCO_3$ aq., and extracted with CH_2Cl_2 3 times. The organic layer was washed with brine, and dried over Na_2SO_4. The solvent was removed under reduced pressure. The crude product was purified by silica gel chromatography (Hexane/Ethyl Acetate = 98:2) to obtain the compound **12** as colourless oil (101.2 mg, 0.202 mmol, 67% yield).

HRMS (FAB) *m/z*: $[M]^+$ calculated for $C_{16}H_9F_{15}O$ 502.0414; found: 502.0409. **^{1}H NMR** (500 MHz, $CDCl_3$) δ 7.37 – 7.32 (m, 2H), 7.28 – 7.25 (m, 1H), 7.18 (s, 2H), 6.59 – 6.53 (m, 1H), 5.66 – 5.59 (m, 1H), 3.51 – 3.49 (m, 2H). **^{13}C NMR** (126 MHz, $CDCl_3$) δ 139.8 (t, *J* = 7.3 Hz), 137.2, 128.9 (2C), 128.9 (2C), 127.6, 127.0, 121.4 (t, *J* = 271.2 Hz), 120.7 (t, *J* =30.0 Hz), 119.2 – 110.3 (m), 37.8. **^{19}F NMR** (282 MHz, $CDCl_3$) δ -66.7 (m, 2F), -81.2 (t, *J* = 9.7 Hz, 3F), -83.2 to -83.3 (m, 2F), -122.8 (br, 2F), -123.4 (br, 2F), -126.1 (br, 2F), -126.6 to -126.7 (m, 2F). **IR (KBr)**: 3733, 3033, 2310, 1683, 1245, 1204, 747, 701, 421 cm^{-1}.

9. Computational details

Density functional theory (DFT) calculations were performed with the Gaussian 16 suite of computational programs. The geometries of all stationary points were optimized using the B3LYP-D3 functional at the basis set level of 6-311+G(d,p) and in diethtylether as solvent using the polarizable continuum model (PCM). All geometry optimizations were performed without symmetry constrains. The geometries of optimized structures were represented using CYLview20, Build 0001.

Cartesian coordination and Gibbs free energies for all the calculated species.

[K][OCF_3]

Zero-point correction= 0.015774 (Hartree/Particle)
Thermal correction to Energy= 0.022190
Thermal correction to Enthalpy= 0.023134
Thermal correction to Gibbs Free Energy= -0.016561
Sum of electronic and zero-point Energies= -441.418168
Sum of electronic and thermal Energies= -441.411752
Sum of electronic and thermal Enthalpies= -441.410808
Sum of electronic and thermal Free Energies= -441.450503

6	2.837494000	4.312690000	-5.455830000
9	3.531724000	5.264349000	-4.701742000
9	3.928188000	3.348353000	-5.768006000
8	2.232286000	4.700087000	-6.449274000
19	3.380272000	3.501010000	-8.456676000
9	2.120043000	3.598676000	-4.490516000

[K][OC_6F_{13}]

Zero-point correction= 0.075621 (Hartree/Particle)
Thermal correction to Energy= 0.096234
Thermal correction to Enthalpy= 0.097178
Thermal correction to Gibbs Free Energy= 0.024312
Sum of electronic and zero-point Energies= -1630.661650
Sum of electronic and thermal Energies= -1630.641038
Sum of electronic and thermal Enthalpies= -1630.640094
Sum of electronic and thermal Free Energies= -1630.712959

6	2.470658000	0.883309000	-1.397616000
9	1.637527000	1.611399000	-0.625731000
9	3.746254000	1.193561000	-1.077350000
6	2.199929000	1.231383000	-2.903060000
9	2.730980000	0.253682000	-3.675406000
9	0.861393000	1.240673000	-3.090254000

6	2.813755000	2.609659000	-3.347191000
9	2.612553000	3.505069000	-2.349725000
9	4.142133000	2.426395000	-3.495292000
6	2.187118000	3.185700000	-4.658691000
9	2.026355000	2.147531000	-5.548783000
9	0.946278000	3.635825000	-4.346039000
6	2.250968000	-0.624953000	-1.040039000
9	1.056104000	-1.043667000	-1.474098000
9	3.203176000	-1.389102000	-1.582236000
9	2.298544000	-0.766591000	0.289544000
6	2.945345000	4.331021000	-5.423925000
9	3.457670000	5.202899000	-4.440051000
9	4.220051000	3.616756000	-5.864356000
8	2.346963000	4.834795000	-6.374987000
19	3.122132000	3.199223000	-8.308977000

G3

Zero-point correction= 0.262855 (Hartree/Particle)
Thermal correction to Energy= 0.278625
Thermal correction to Enthalpy= 0.279569
Thermal correction to Gibbs Free Energy= 0.216905
Sum of electronic and zero-point Energies= -616.481189
Sum of electronic and thermal Energies= -616.465420
Sum of electronic and thermal Enthalpies= -616.464475
Sum of electronic and thermal Free Energies= -616.527139

1	2.900530000	-1.574236000	-3.127722000
6	3.276964000	-0.688020000	-2.613814000
1	3.927412000	-1.004867000	-1.786192000
1	3.872306000	-0.091992000	-3.320070000
8	2.151470000	0.036638000	-2.142010000
6	2.513259000	1.220713000	-1.448221000
1	3.137355000	0.985498000	-0.573638000
1	3.085171000	1.896443000	-2.100893000
6	1.232248000	1.899809000	-0.999329000
1	0.660709000	1.225456000	-0.345922000
1	0.608768000	2.136644000	-1.873148000
8	1.595292000	3.084560000	-0.304859000
6	0.475586000	3.817804000	0.169674000
1	-0.173078000	4.121193000	-0.664332000
1	-0.122964000	3.209694000	0.862733000
6	1.004334000	5.047040000	0.886078000

1	1.653041000	4.743608000	1.720033000
1	1.602839000	5.655160000	0.192992000
8	-0.115334000	5.780278000	1.360699000
6	0.247785000	6.965051000	2.055087000
1	0.871342000	6.728256000	2.928862000
1	0.819274000	7.639373000	1.401601000
6	-1.033172000	7.644188000	2.504072000
1	-1.605038000	6.968497000	3.156825000
1	-1.657345000	7.879384000	1.629538000
8	-0.671286000	8.828279000	3.197777000
6	-1.796708000	9.552856000	3.669881000
1	-2.391873000	8.956750000	4.376218000
1	-2.447342000	9.869751000	2.842423000
1	-1.420189000	10.439042000	4.183777000

[KF][2G3]

Zero-point correction= 0.532057 (Hartree/Particle)
Thermal correction to Energy= 0.567023
Thermal correction to Enthalpy= 0.567967
Thermal correction to Gibbs Free Energy= 0.463822
Sum of electronic and zero-point Energies= -1361.325746
Sum of electronic and thermal Energies= -1361.290780
Sum of electronic and thermal Enthalpies= -1361.289836
Sum of electronic and thermal Free Energies= -1361.393980

1	1.703145000	0.160931000	-1.908164000
6	1.980939000	-0.660843000	-1.250994000
1	2.974874000	-1.022377000	-1.541906000
1	2.017600000	-0.314123000	-0.207974000
8	0.999103000	-1.677650000	-1.417470000
6	1.286918000	-2.852706000	-0.676280000
1	2.311335000	-3.191531000	-0.882322000
1	1.203912000	-2.658700000	0.404290000
6	0.308964000	-3.938930000	-1.058087000
1	-0.722527000	-3.597048000	-0.897910000
1	0.481834000	-4.813352000	-0.413105000
8	0.510863000	-4.278056000	-2.423297000
6	-0.294848000	-5.361213000	-2.873517000
1	-0.060928000	-6.274129000	-2.304491000
1	-1.360785000	-5.132901000	-2.729907000
6	0.008061000	-5.588288000	-4.341801000
1	1.090886000	-5.665205000	-4.476339000

1	-0.441524000	-6.534658000	-4.668343000
8	-0.436330000	-4.505464000	-5.152979000
6	-1.724019000	-4.701204000	-5.738868000
1	-1.663085000	-5.480367000	-6.512004000
1	-2.451298000	-5.023323000	-4.980978000
6	-2.188818000	-3.409650000	-6.367082000
1	-3.044985000	-3.623311000	-7.025813000
1	-1.382291000	-2.966141000	-6.965653000
8	-2.578460000	-2.507386000	-5.335241000
6	-2.971044000	-1.234863000	-5.850094000
1	-3.842761000	-1.334423000	-6.513888000
1	-2.142550000	-0.771060000	-6.397726000
1	-3.234110000	-0.615057000	-4.993528000
19	0.208697000	-1.782003000	-4.269982000
1	2.873494000	-4.009686000	-3.197831000
6	3.668403000	-3.363810000	-3.570047000
1	4.279612000	-3.018100000	-2.723616000
1	4.314773000	-3.935992000	-4.251607000
8	3.047372000	-2.279385000	-4.238271000
6	3.976050000	-1.323158000	-4.737229000
1	4.562836000	-0.900253000	-3.907967000
1	4.673769000	-1.802556000	-5.440271000
6	3.229209000	-0.228821000	-5.461347000
1	2.556045000	-0.664313000	-6.211086000
1	3.956774000	0.419094000	-5.974784000
8	2.478129000	0.524930000	-4.511892000
6	1.561141000	1.429192000	-5.125762000
1	2.102958000	2.233479000	-5.648176000
1	0.940371000	0.887861000	-5.851077000
6	0.693298000	2.044844000	-4.054110000
1	1.304048000	2.584335000	-3.314694000
1	0.014222000	2.767001000	-4.529645000
8	-0.047265000	1.014770000	-3.406740000
6	-1.108823000	1.496816000	-2.597931000
1	-0.742228000	2.244731000	-1.878291000
1	-1.874722000	1.973370000	-3.226859000
6	-1.712036000	0.344216000	-1.828006000
1	-2.520333000	0.734285000	-1.190583000
1	-0.953407000	-0.124607000	-1.187942000
8	-2.218898000	-0.610997000	-2.750849000
6	-2.951381000	-1.656301000	-2.132175000
1	-3.871737000	-1.276061000	-1.665275000

1	-2.356326000	-2.163393000	-1.360923000
1	-3.209794000	-2.368289000	-2.916581000
9	0.229134000	-1.056195000	-6.767429000

[K][2G3]

Zero-point correction= 0.532035 (Hartree/Particle)

Thermal correction to Energy= 0.564718

Thermal correction to Enthalpy= 0.565662

Thermal correction to Gibbs Free Energy= 0.467889

Sum of electronic and zero-point Energies= -1261.294931

Sum of electronic and thermal Energies= -1261.262248

Sum of electronic and thermal Enthalpies= -1261.261303

Sum of electronic and thermal Free Energies= -1261.359077

1	1.440925000	0.285101000	-1.478250000
6	1.796299000	-0.573810000	-0.909235000
1	2.833397000	-0.786511000	-1.198042000
1	1.765891000	-0.341846000	0.163456000
8	0.938552000	-1.668181000	-1.221481000
6	1.325464000	-2.879872000	-0.580172000
1	2.358292000	-3.137150000	-0.851735000
1	1.274578000	-2.767779000	0.512154000
6	0.393523000	-3.991579000	-1.002391000
1	-0.647389000	-3.727184000	-0.770085000
1	0.651242000	-4.898959000	-0.438722000
8	0.544520000	-4.216170000	-2.402538000
6	-0.168090000	-5.353407000	-2.884637000
1	0.176960000	-6.263848000	-2.373648000
1	-1.242802000	-5.242611000	-2.685407000
6	0.104879000	-5.494141000	-4.371442000
1	1.184104000	-5.501400000	-4.540789000
1	-0.303373000	-6.449503000	-4.725695000
8	-0.410509000	-4.409987000	-5.145738000
6	-1.785692000	-4.550894000	-5.502829000
1	-1.923120000	-5.443265000	-6.129018000
1	-2.413970000	-4.656699000	-4.609078000
6	-2.219783000	-3.330055000	-6.277421000
1	-3.245864000	-3.488077000	-6.638972000
1	-1.568616000	-3.179505000	-7.150218000
8	-2.165679000	-2.199266000	-5.414869000
6	-2.712236000	-1.017873000	-5.991855000
1	-3.763472000	-1.166294000	-6.272557000

1	-2.150244000	-0.711221000	-6.884824000
1	-2.653457000	-0.241417000	-5.230211000
19	0.226679000	-1.797976000	-3.935832000
1	2.836864000	-4.131162000	-3.447133000
6	3.609639000	-3.457910000	-3.815591000
1	4.188012000	-3.077774000	-2.962853000
1	4.288263000	-4.004166000	-4.484399000
8	2.954635000	-2.397636000	-4.504007000
6	3.857191000	-1.385380000	-4.939647000
1	4.413990000	-0.983626000	-4.081634000
1	4.581069000	-1.798758000	-5.656314000
6	3.082718000	-0.274285000	-5.606960000
1	2.480742000	-0.674190000	-6.436354000
1	3.791528000	0.455149000	-6.023446000
8	2.244219000	0.341380000	-4.634125000
6	1.451425000	1.404244000	-5.149827000
1	2.085835000	2.187269000	-5.589297000
1	0.778183000	1.032075000	-5.936691000
6	0.646343000	2.002055000	-4.018903000
1	1.314061000	2.427620000	-3.257479000
1	0.025135000	2.812790000	-4.423657000
8	-0.165555000	0.985865000	-3.438623000
6	-1.205032000	1.489842000	-2.603143000
1	-0.806199000	2.219873000	-1.885111000
1	-1.963721000	1.992290000	-3.218554000
6	-1.824431000	0.345028000	-1.833538000
1	-2.647291000	0.740426000	-1.220123000
1	-1.084669000	-0.116048000	-1.166167000
8	-2.305866000	-0.620185000	-2.761851000
6	-3.005576000	-1.692779000	-2.147026000
1	-3.905112000	-1.336699000	-1.626459000
1	-2.371559000	-2.225428000	-1.424541000
1	-3.298073000	-2.375049000	-2.945640000

$FCOC_5F_{11}$

Zero-point correction= 0.073470 (Hartree/Particle)
Thermal correction to Energy= 0.090907
Thermal correction to Enthalpy= 0.091851
Thermal correction to Gibbs Free Energy= 0.026954
Sum of electronic and zero-point Energies= -1502.347983
Sum of electronic and thermal Energies= -1502.330546
Sum of electronic and thermal Enthalpies= -1502.329602

Sum of electronic and thermal Free Energies= -1502.394499

6	2.527371000	0.869273000	-1.425501000
9	1.782457000	1.618518000	-0.587940000
9	3.833616000	1.146028000	-1.219226000
6	2.148400000	1.237423000	-2.901557000
9	2.621744000	0.282954000	-3.729927000
9	0.801833000	1.268302000	-2.999660000
6	2.726887000	2.623826000	-3.359820000
9	2.599226000	3.519737000	-2.358292000
9	4.042038000	2.475008000	-3.641319000
6	2.034668000	3.210735000	-4.636371000
9	1.923534000	2.239135000	-5.562937000
9	0.802174000	3.661390000	-4.317228000
6	2.293307000	-0.634390000	-1.056551000
9	1.046816000	-1.004277000	-1.370737000
9	3.158279000	-1.423867000	-1.696949000
9	2.469437000	-0.785106000	0.260222000
6	2.864875000	4.372196000	-5.245653000
9	2.746870000	5.469172000	-4.484876000
8	3.510171000	4.322531000	-6.226610000

[Na(G3)$_2$][OC$_6$F$_{13}$]

Zero-point correction= 0.608191 (Hartree/Particle)
Thermal correction to Energy= 0.661357
Thermal correction to Enthalpy= 0.662301
Thermal correction to Gibbs Free Energy= 0.515588
Sum of electronic and zero-point Energies= -2997.671153
Sum of electronic and thermal Energies= -2997.617987
Sum of electronic and thermal Enthalpies= -2997.617043
Sum of electronic and thermal Free Energies= -2997.763757

1	1.376720000	0.499310000	-2.074288000
6	1.577214000	-0.277492000	-1.342692000
1	2.641001000	-0.538088000	-1.397091000
1	1.347758000	0.084607000	-0.329648000
8	0.759618000	-1.390419000	-1.681528000
6	0.992947000	-2.502007000	-0.833147000
1	2.056127000	-2.776904000	-0.854186000
1	0.725692000	-2.258716000	0.207049000
6	0.156502000	-3.672549000	-1.289484000
1	-0.911099000	-3.421674000	-1.259357000

1	0.328414000	-4.513023000	-0.600772000
8	0.545767000	-4.023549000	-2.609672000
6	-0.008665000	-5.249342000	-3.068843000
1	0.226670000	-6.064316000	-2.367621000
1	-1.102572000	-5.172002000	-3.143574000
6	0.612110000	-5.557457000	-4.417128000
1	1.701036000	-5.541385000	-4.325304000
1	0.313645000	-6.564267000	-4.738989000
8	0.271351000	-4.585655000	-5.397457000
6	-0.926570000	-4.891530000	-6.108974000
1	-0.759455000	-5.760519000	-6.761765000
1	-1.743950000	-5.133897000	-5.416405000
6	-1.310087000	-3.701092000	-6.948332000
1	-2.107771000	-3.993969000	-7.648444000
1	-0.443070000	-3.369273000	-7.523782000
8	-1.760875000	-2.666848000	-6.085882000
6	-2.176914000	-1.498507000	-6.783977000
1	-2.972105000	-1.733560000	-7.506639000
1	-1.341015000	-1.028557000	-7.309357000
1	-2.559824000	-0.808447000	-6.033536000
11	0.640168000	-1.785049000	-4.512543000
1	2.876847000	-4.070939000	-2.913412000
6	3.748393000	-3.446622000	-3.093902000
1	4.195100000	-3.169045000	-2.128127000
1	4.496708000	-4.007668000	-3.672106000
8	3.306055000	-2.301011000	-3.806002000
6	4.343079000	-1.337127000	-3.959349000
1	4.619565000	-0.927092000	-2.976604000
1	5.237066000	-1.806821000	-4.395877000
6	3.883919000	-0.237879000	-4.881857000
1	3.559640000	-0.666857000	-5.833778000
1	4.728063000	0.441737000	-5.076781000
8	2.809345000	0.464249000	-4.266126000
6	2.187835000	1.403049000	-5.136076000
1	2.867031000	2.239868000	-5.360888000
1	1.912254000	0.922014000	-6.076087000
6	0.949852000	1.939928000	-4.460856000
1	1.209729000	2.505531000	-3.553866000
1	0.441221000	2.621858000	-5.156332000
8	0.103732000	0.847453000	-4.114191000
6	-1.202639000	1.249677000	-3.722854000
1	-1.161302000	2.154200000	-3.099277000

1	-1.802282000	1.475959000	-4.615269000
6	-1.844408000	0.145317000	-2.914699000
1	-2.876668000	0.443290000	-2.678158000
1	-1.298010000	-0.013761000	-1.977086000
8	-1.831123000	-1.052399000	-3.679218000
6	-2.696933000	-2.049200000	-3.161238000
1	-3.749010000	-1.744431000	-3.257690000
1	-2.490446000	-2.250120000	-2.102412000
1	-2.534430000	-2.948909000	-3.751626000
8	1.213355000	-1.444771000	-6.806199000
6	1.460973000	-0.919517000	-7.889356000
6	1.296700000	-1.810462000	-9.180181000
6	1.959629000	-1.316808000	-10.508971000
6	1.401652000	-2.030514000	-11.795076000
6	2.334345000	-1.885384000	-13.047773000
6	1.644051000	-2.235923000	-14.408336000
9	2.576303000	-2.271474000	-15.369317000
9	1.050963000	-3.434213000	-14.343662000
9	0.733338000	-1.316283000	-14.737649000
9	2.783644000	-0.613959000	-13.140751000
9	3.392095000	-2.712593000	-12.908319000
9	1.242144000	-3.352518000	-11.555447000
9	0.193540000	-1.502541000	-12.100826000
9	3.294513000	-1.549913000	-10.455883000
9	1.760870000	0.009329000	-10.672162000
9	1.812420000	-3.042112000	-8.915010000
9	-0.039221000	-1.976786000	-9.428770000
9	0.679443000	0.273625000	-8.190907000
9	2.820810000	-0.382718000	-8.030454000

$[K(G3)_2][OC_6F_{13}]$

Zero-point correction= 0.606854 (Hartree/Particle)
Thermal correction to Energy= 0.660589
Thermal correction to Enthalpy= 0.661533
Thermal correction to Gibbs Free Energy= 0.512199
Sum of electronic and zero-point Energies= -2863.713291
Sum of electronic and thermal Energies= -2863.659556
Sum of electronic and thermal Enthalpies= -2863.658612
Sum of electronic and thermal Free Energies= -2863.807946

1	1.372334000	0.353894000	-1.969699000
6	1.525117000	-0.405213000	-1.205471000

1	2.579393000	-0.707529000	-1.219014000
1	1.281892000	0.002741000	-0.213974000
8	0.675418000	-1.500987000	-1.529147000
6	0.838729000	-2.601996000	-0.647776000
1	1.898801000	-2.882870000	-0.584269000
1	0.494605000	-2.340254000	0.364477000
6	0.033793000	-3.776333000	-1.152201000
1	-1.024358000	-3.500006000	-1.255519000
1	0.102275000	-4.591436000	-0.416799000
8	0.562124000	-4.190621000	-2.405778000
6	-0.028488000	-5.381228000	-2.915345000
1	0.132379000	-6.216358000	-2.216925000
1	-1.112280000	-5.247771000	-3.041947000
6	0.633654000	-5.701098000	-4.241930000
1	1.719364000	-5.688428000	-4.113250000
1	0.341769000	-6.709104000	-4.564481000
8	0.328896000	-4.738491000	-5.246807000
6	-0.829554000	-5.055443000	-6.019224000
1	-0.629590000	-5.939593000	-6.641114000
1	-1.684660000	-5.278790000	-5.366950000
6	-1.166531000	-3.887077000	-6.912236000
1	-1.921539000	-4.206623000	-7.646302000
1	-0.271888000	-3.558516000	-7.452317000
8	-1.676928000	-2.826125000	-6.111222000
6	-2.076573000	-1.694028000	-6.882602000
1	-2.869269000	-1.966108000	-7.594182000
1	-1.231402000	-1.269851000	-7.432654000
1	-2.455948000	-0.953827000	-6.178980000
19	0.654365000	-1.897930000	-4.438720000
1	3.014120000	-3.961125000	-2.648596000
6	3.865669000	-3.305937000	-2.828454000
1	4.220364000	-2.905975000	-1.867698000
1	4.682686000	-3.881497000	-3.286810000
8	3.422686000	-2.268316000	-3.688600000
6	4.422055000	-1.282875000	-3.927896000
1	4.709456000	-0.802793000	-2.980785000
1	5.319471000	-1.747982000	-4.362168000
6	3.892932000	-0.252847000	-4.895718000
1	3.539501000	-0.741208000	-5.810419000
1	4.708503000	0.434911000	-5.166175000
8	2.827648000	0.459678000	-4.271078000
6	2.167217000	1.363718000	-5.151459000

1	2.852188000	2.160817000	-5.478029000
1	1.809455000	0.835966000	-6.040836000
6	1.001557000	1.987344000	-4.422037000
1	1.348734000	2.555992000	-3.546837000
1	0.495789000	2.683402000	-5.105714000
8	0.110406000	0.957827000	-4.003051000
6	-1.146207000	1.445516000	-3.552483000
1	-1.008557000	2.272962000	-2.840547000
1	-1.729705000	1.820488000	-4.405140000
6	-1.894296000	0.335009000	-2.851291000
1	-2.858401000	0.732034000	-2.499071000
1	-1.322792000	-0.021178000	-1.984223000
8	-2.098419000	-0.735989000	-3.764123000
6	-2.931991000	-1.761310000	-3.245917000
1	-3.953008000	-1.393186000	-3.070245000
1	-2.538788000	-2.158950000	-2.300847000
1	-2.956126000	-2.555607000	-3.992167000
8	1.401772000	-1.550729000	-7.028798000
6	1.601275000	-0.979844000	-8.097351000
6	1.439894000	-1.845335000	-9.403211000
6	1.972714000	-1.262786000	-10.754491000
6	1.389466000	-1.980962000	-12.026407000
6	2.225311000	-1.727863000	-13.329262000
6	1.479172000	-2.094908000	-14.655226000
9	2.345966000	-2.020888000	-15.673720000
9	0.994734000	-3.341212000	-14.596601000
9	0.474717000	-1.247420000	-14.893639000
9	2.568467000	-0.423036000	-13.408506000
9	3.350413000	-2.472597000	-13.282096000
9	1.347640000	-3.317696000	-11.821939000
9	0.126758000	-1.541071000	-12.237073000
9	3.322800000	-1.390744000	-10.790629000
9	1.665487000	0.048068000	-10.862047000
9	2.078430000	-3.032865000	-9.209296000
9	0.110239000	-2.126994000	-9.568073000
9	0.755431000	0.184760000	-8.342979000
9	2.929789000	-0.375227000	-8.248052000

$[Cs(G3)_2][OC_6F_{13}]$

Zero-point correction= 0.605586 (Hartree/Particle)

Thermal correction to Energy= 0.659953

Thermal correction to Enthalpy= 0.660897

Thermal correction to Gibbs Free Energy= 0.506100

Sum of electronic and zero-point Energies= -2855.548950

Sum of electronic and thermal Energies= -2855.494583

Sum of electronic and thermal Enthalpies= -2855.493639

Sum of electronic and thermal Free Energies= -2855.648436

1	1.753920000	-0.159386000	-1.992585000
6	2.119002000	-1.118938000	-1.629497000
1	3.083315000	-1.325774000	-2.108479000
1	2.257289000	-1.079823000	-0.539630000
8	1.149970000	-2.100655000	-1.986054000
6	1.540526000	-3.418421000	-1.628304000
1	2.536825000	-3.644749000	-2.031794000
1	1.586422000	-3.518237000	-0.533067000
6	0.539730000	-4.414932000	-2.164265000
1	-0.477292000	-4.127704000	-1.861195000
1	0.759520000	-5.399438000	-1.725668000
8	0.633274000	-4.478395000	-3.583992000
6	-0.193169000	-5.492062000	-4.149238000
1	0.116321000	-6.480700000	-3.777362000
1	-1.241144000	-5.336117000	-3.856326000
6	-0.049185000	-5.469569000	-5.660164000
1	1.012541000	-5.433234000	-5.918582000
1	-0.467122000	-6.396842000	-6.074741000
8	-0.653208000	-4.333260000	-6.272916000
6	-2.038248000	-4.497400000	-6.572546000
1	-2.166530000	-5.301698000	-7.311166000
1	-2.607695000	-4.764339000	-5.672176000
6	-2.584590000	-3.212782000	-7.149309000
1	-3.585913000	-3.408406000	-7.561525000
1	-1.940309000	-2.865219000	-7.967131000
8	-2.659518000	-2.231076000	-6.121957000
6	-3.136532000	-0.970647000	-6.588593000
1	-4.152488000	-1.060148000	-6.997562000
1	-2.475940000	-0.551810000	-7.357394000
1	-3.153314000	-0.298374000	-5.729487000
55	0.329100000	-1.516450000	-5.027933000
1	3.338907000	-3.788939000	-4.446262000
6	4.117672000	-3.029044000	-4.525532000
1	4.640923000	-2.950079000	-3.562024000
1	4.843545000	-3.337680000	-5.291246000
8	3.484353000	-1.810242000	-4.876101000

6	4.387722000	-0.714685000	-4.965512000
1	4.866351000	-0.539898000	-3.990593000
1	5.177411000	-0.931339000	-5.700179000
6	3.645182000	0.524768000	-5.407336000
1	3.114456000	0.329264000	-6.347050000
1	4.376817000	1.325532000	-5.594235000
8	2.732569000	0.914166000	-4.386589000
6	1.901801000	2.007668000	-4.770171000
1	2.512004000	2.898182000	-4.985614000
1	1.333596000	1.756258000	-5.674190000
6	0.962515000	2.335244000	-3.632966000
1	1.531038000	2.604790000	-2.730626000
1	0.354087000	3.202673000	-3.926357000
8	0.131344000	1.211524000	-3.360235000
6	-0.928412000	1.497265000	-2.457615000
1	-0.542316000	1.999360000	-1.557645000
1	-1.660965000	2.164606000	-2.933424000
6	-1.595181000	0.210426000	-2.029251000
1	-2.347365000	0.447739000	-1.261446000
1	-0.853309000	-0.469699000	-1.588378000
8	-2.214138000	-0.407993000	-3.152289000
6	-2.919385000	-1.592147000	-2.805960000
1	-3.759197000	-1.372836000	-2.131233000
1	-2.260798000	-2.322233000	-2.314408000
1	-3.298669000	-2.024035000	-3.732240000
8	0.228521000	0.497056000	-7.432685000
6	0.665245000	0.327682000	-8.568508000
6	1.253393000	-1.101588000	-8.876765000
6	2.050673000	-1.338031000	-10.199437000
6	2.217981000	-2.857893000	-10.569569000
6	3.362962000	-3.129938000	-11.607377000
6	3.285968000	-4.535150000	-12.294008000
9	4.410819000	-4.741190000	-12.990662000
9	3.168581000	-5.504336000	-11.377095000
9	2.250370000	-4.603895000	-13.134506000
9	3.329504000	-2.199900000	-12.586901000
9	4.554313000	-3.053883000	-10.975326000
9	2.498511000	-3.576302000	-9.457425000
9	1.052867000	-3.312642000	-11.087583000
9	3.292242000	-0.805272000	-10.069359000
9	1.436481000	-0.738627000	-11.241288000
9	2.098403000	-1.435464000	-7.854575000

9	0.221245000	-1.999289000	-8.845212000
9	-0.286549000	0.556547000	-9.643938000
9	1.748876000	1.232620000	-8.970604000

10. References

1. Jiang, X.; Wang, G.; Zheng, Z.; Xiaohui, Y.; Hong, Y.; Xia, H.; Yu, C. Autocatalytic Synthesis of Thioesters via Thiocarbonylation of *gem*-Difluoroalkenes. *Org. Lett.* **2020,** *22*, 9762-9766.

2. Hu, J.; Han, X.; Yuan, Y.; Shi, Z. Stereoselective Synthesis of Z Fluoroalkenes through Copper-Catalyzed Hydrodefluorination of *gem*-Difluoroalkenes with Water. *Angew. Chem. Int. Ed.* **2017,** *56*, 13342-13346.

3. Ito, H.; Seo, T.; Kojima, R.; Kubota, K. Copper(I)-catalyzed Stereoselective Defluoroborylation of Aliphatic *Gem*-Difluoroalkenes. *Chem. Lett.* **2018,** *47*, 1330-1332.

4. Ross, S. P.; Rahman, A. A.; Sigman, M. S. Development and Mechanistic Interrogation of Interrupted Chain-Walking in the Enantioselective Relay Heck Reaction. *J. Am. Chem. Soc.* **2020,** *142*, 10516−10525.

5. Wu, Z.; Xu, X.; Wang, J.; Dong, G. Carbonyl 1,2-transposition through triflate-mediated α-amination. *Science* **2021,** *374*, 734-740.

6. Kampmann, S. S.; Skelton, B. W.; Yeoh, G. C.; Abrahama, L. J.; Lengkeek, N. A.; Stubbs, K. A.; Heath, C. H.; Stewart, S. G. The synthesis and fluorescence profile of novel thalidomide analogues. *Tetrahedron.* **2015,** *71*, 8140-8149.

11. NMR data

¹H NMR (500 MHz, $CDCl_3$): **1b**

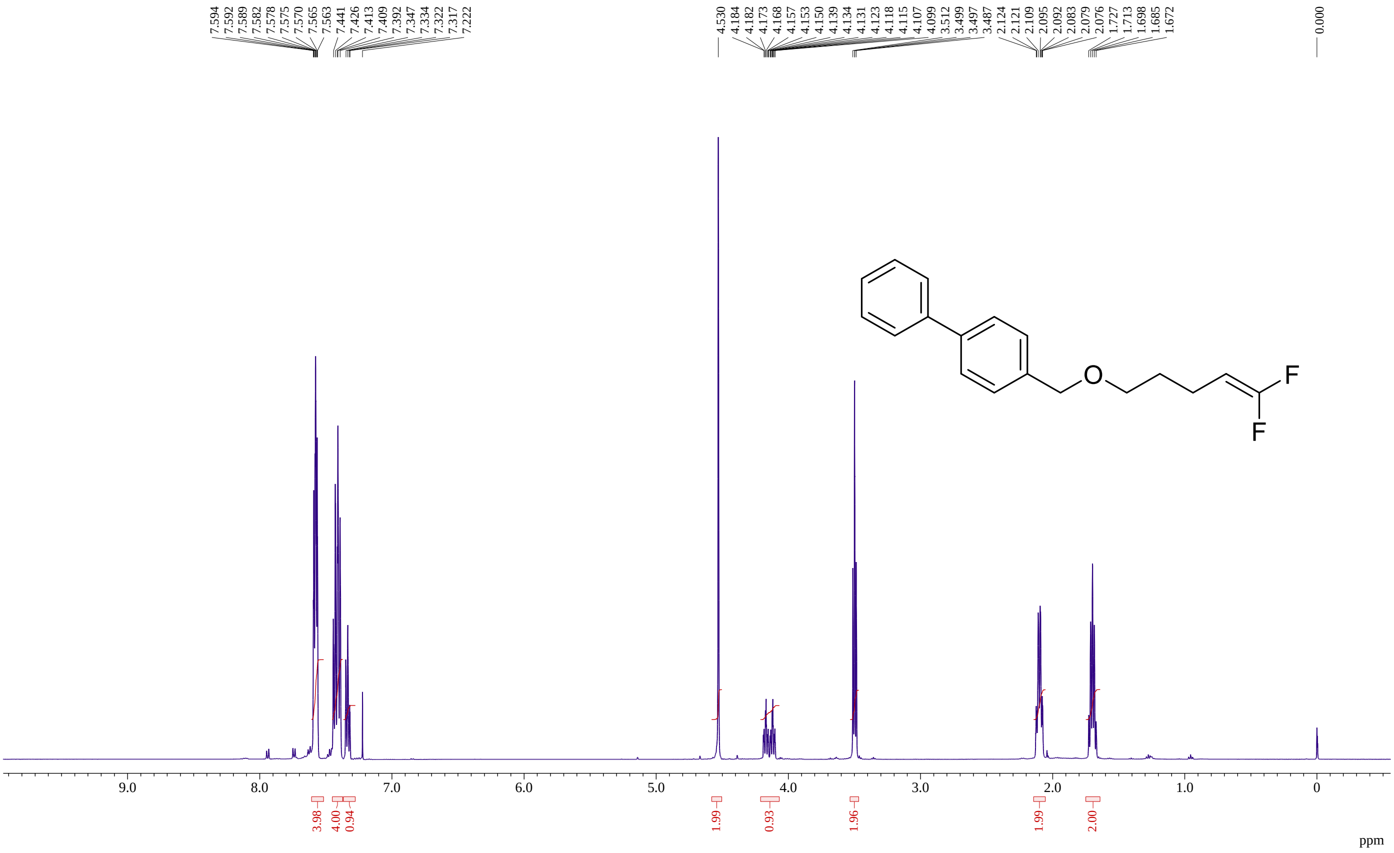

¹³C NMR (126 MHz, $CDCl_3$): **1b**

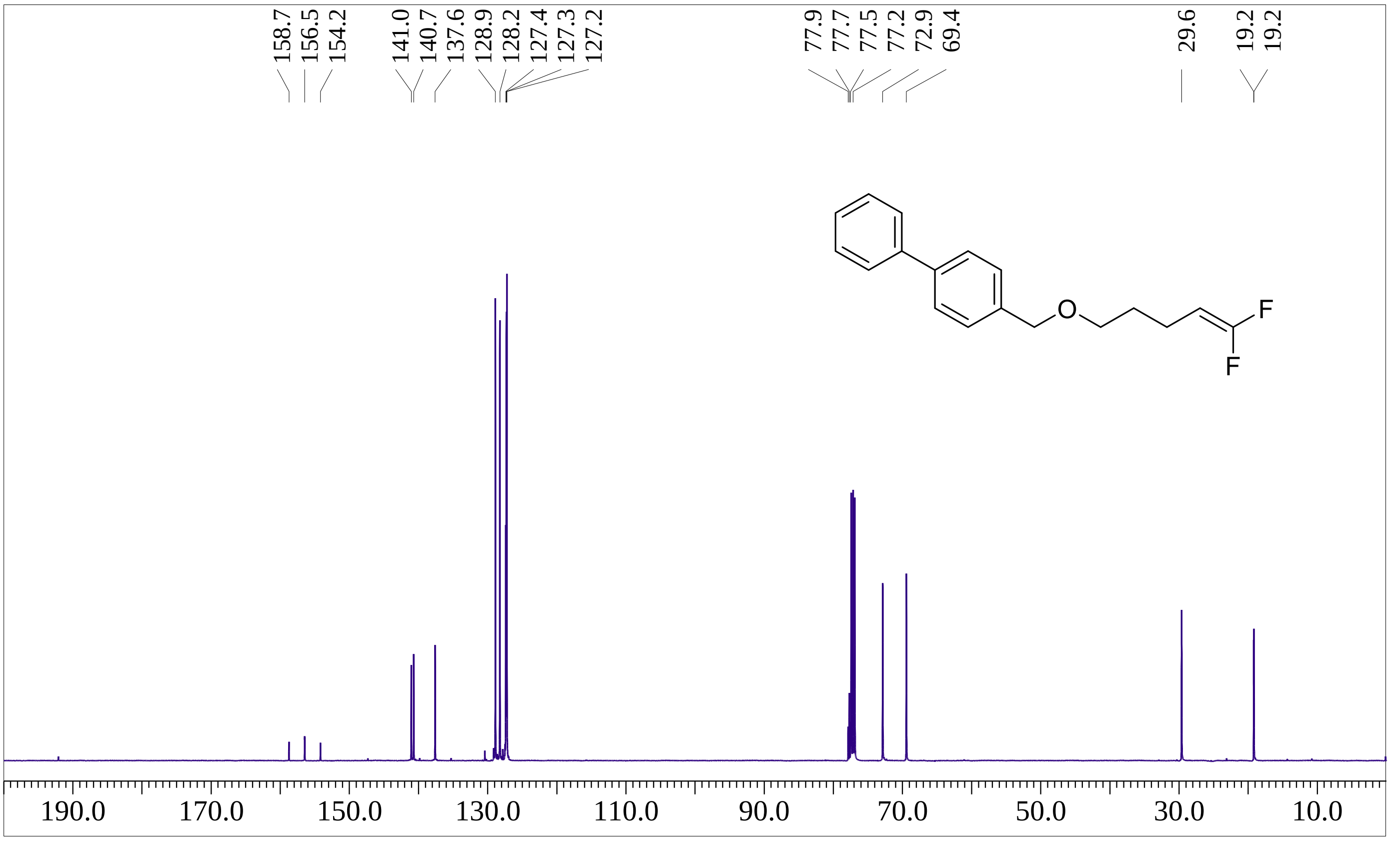

^{19}**F NMR** (282 MHz, $CDCl_3$): **1b**

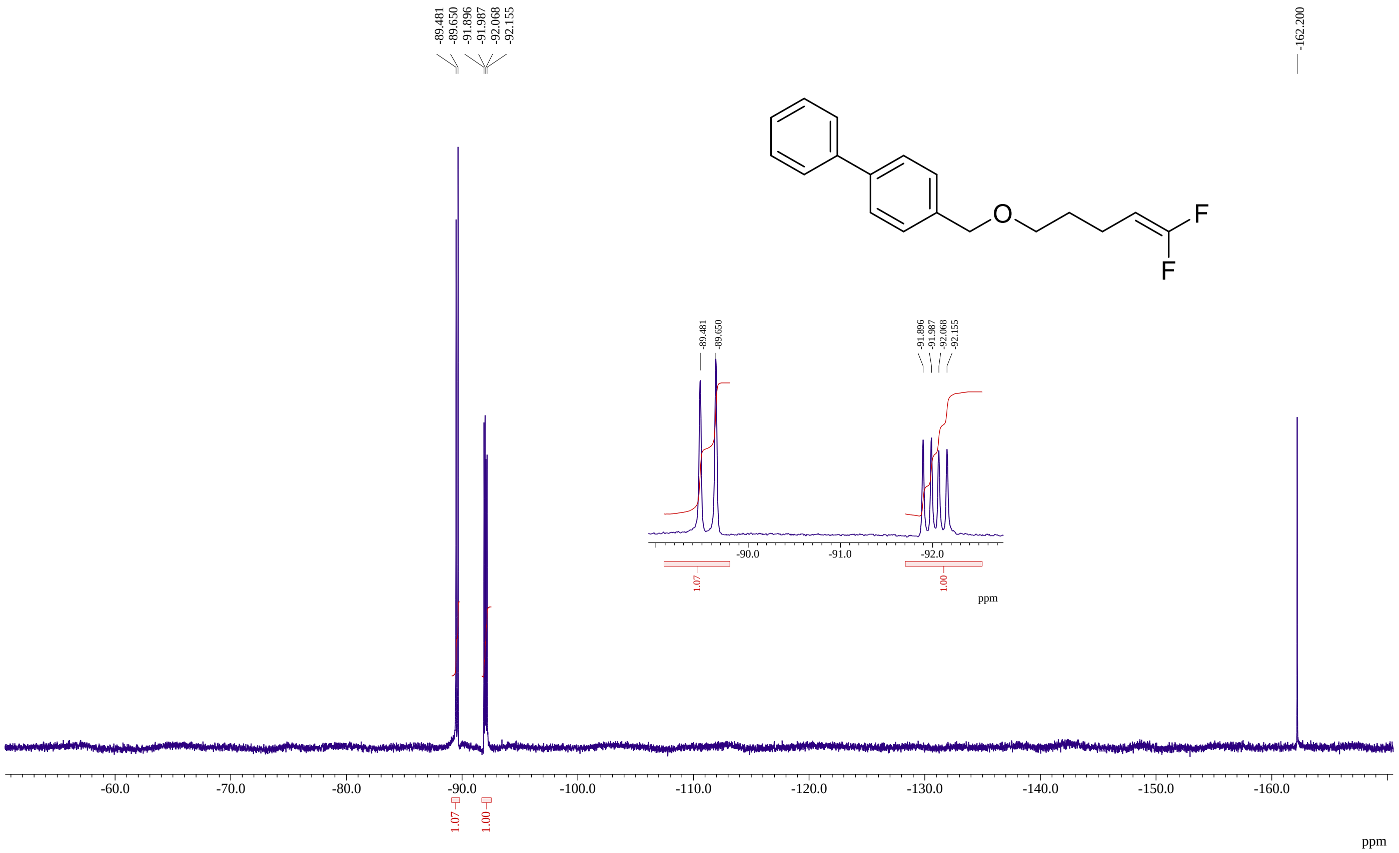

^{1}**H NMR** (500 MHz, $CDCl_3$): **1e**

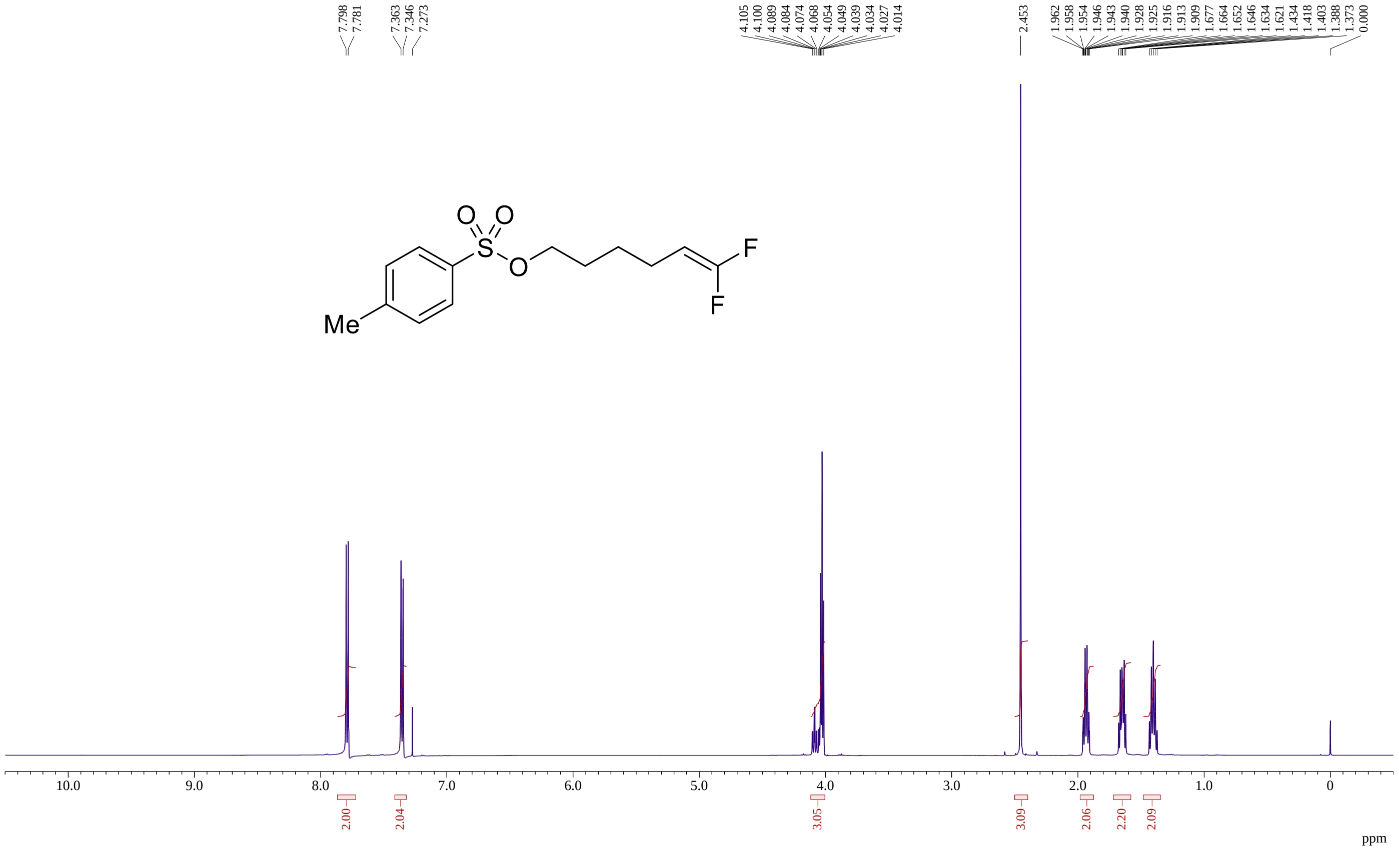

^{13}C NMR (126 MHz, $CDCl_3$): **1e**

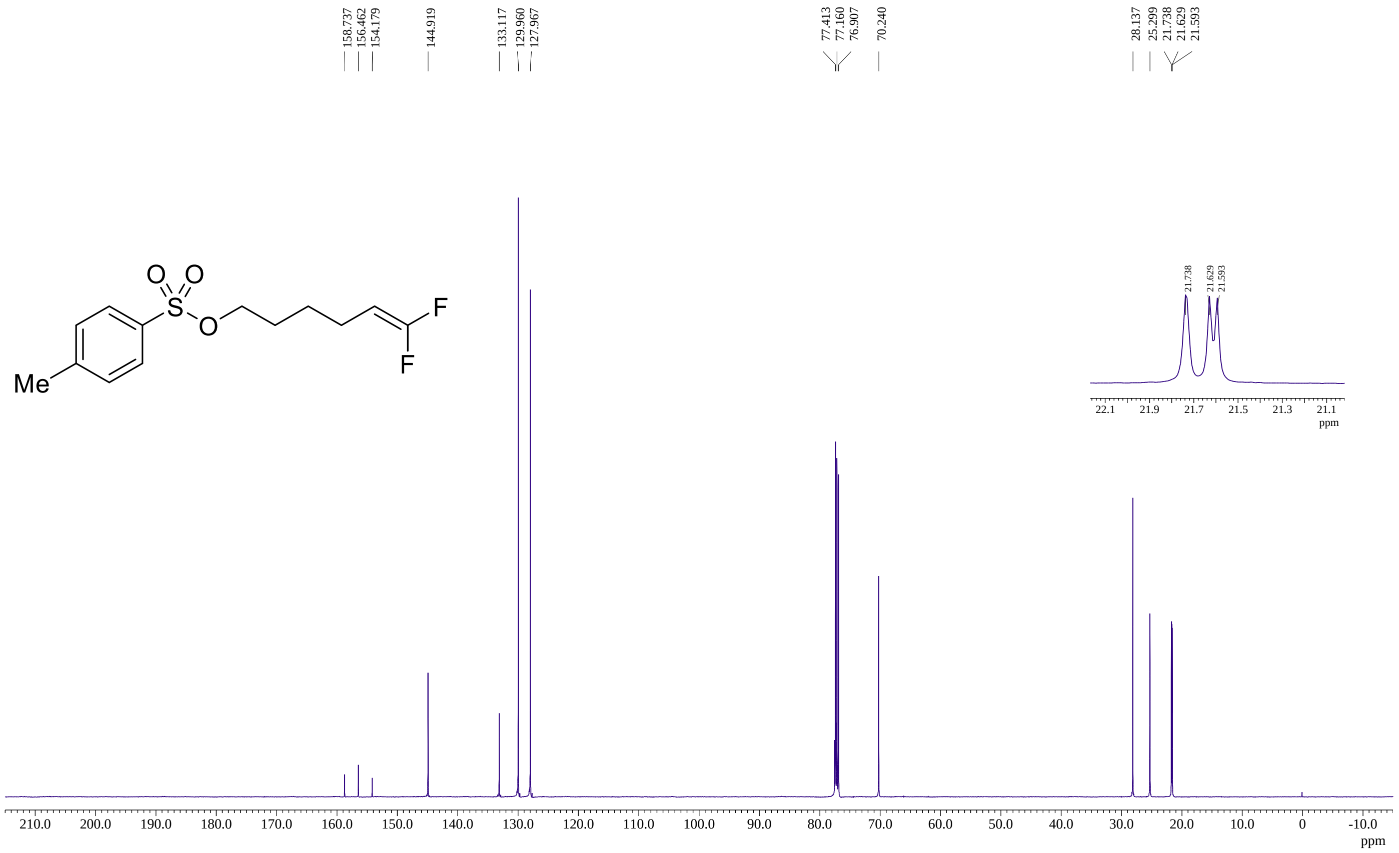

^{19}F NMR (282 MHz, $CDCl_3$): **1e**

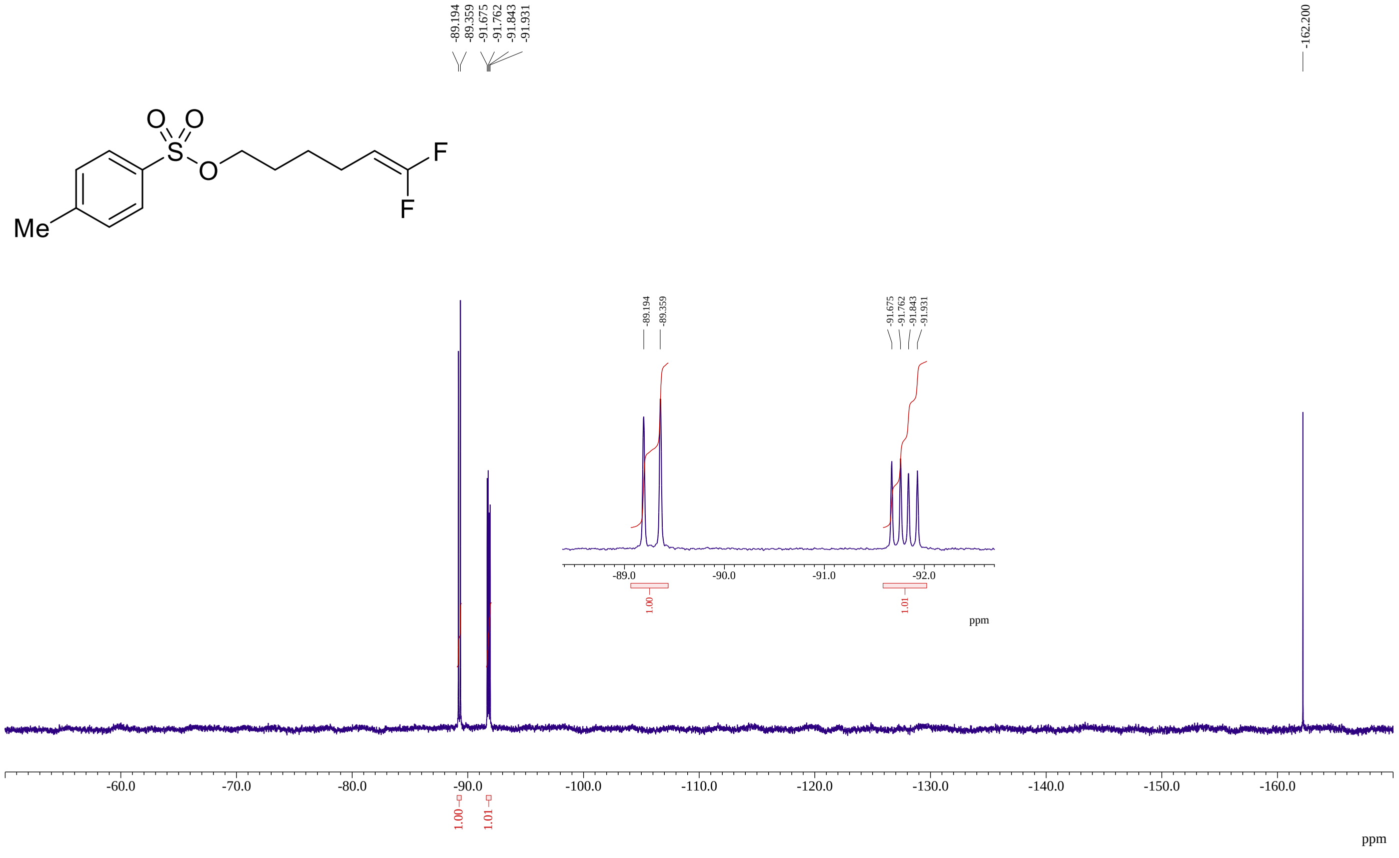

[1]**H NMR** (500 MHz, $CDCl_3$): **1f**

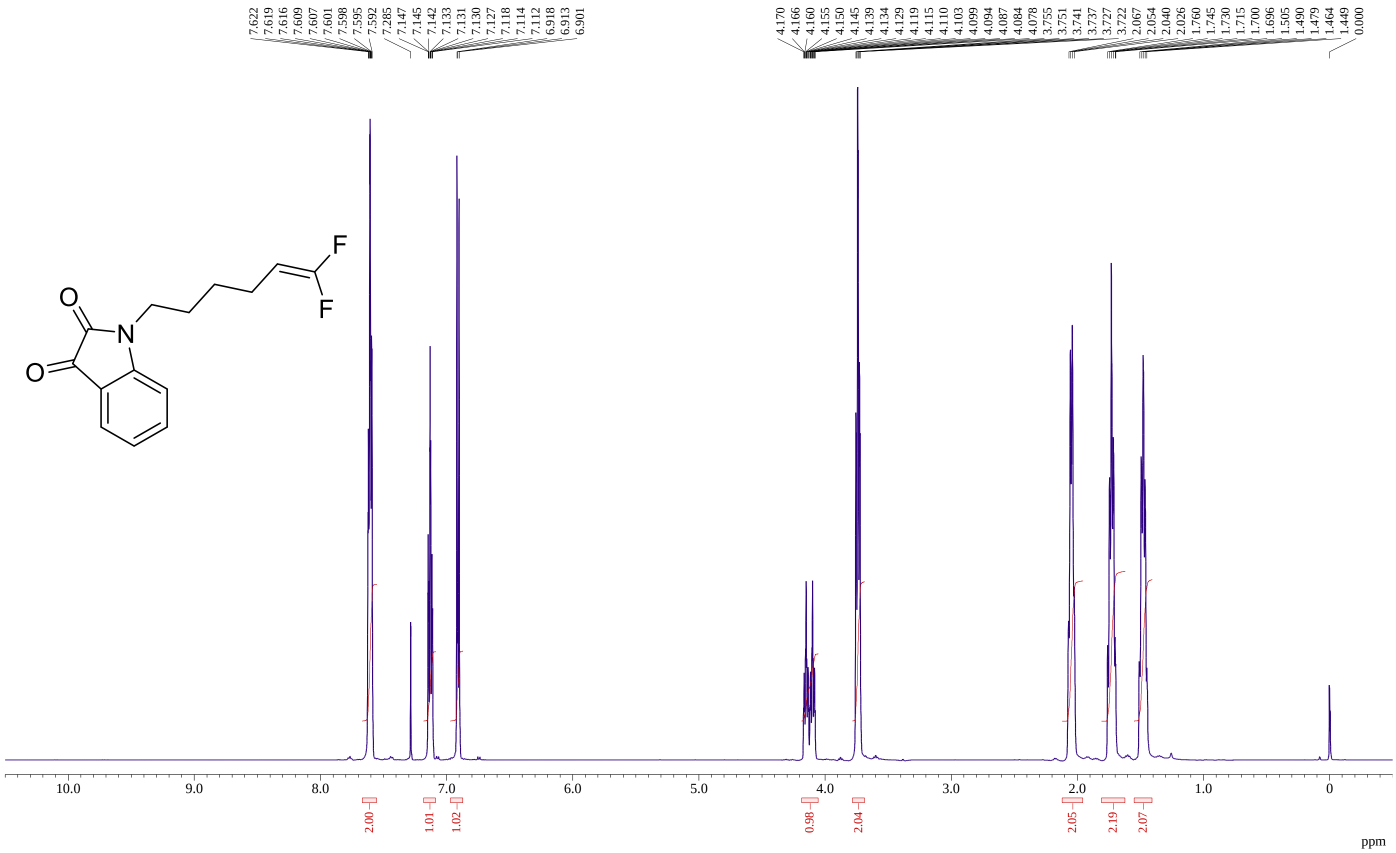

[13]**C NMR** (126 MHz, $CDCl_3$): **1f**

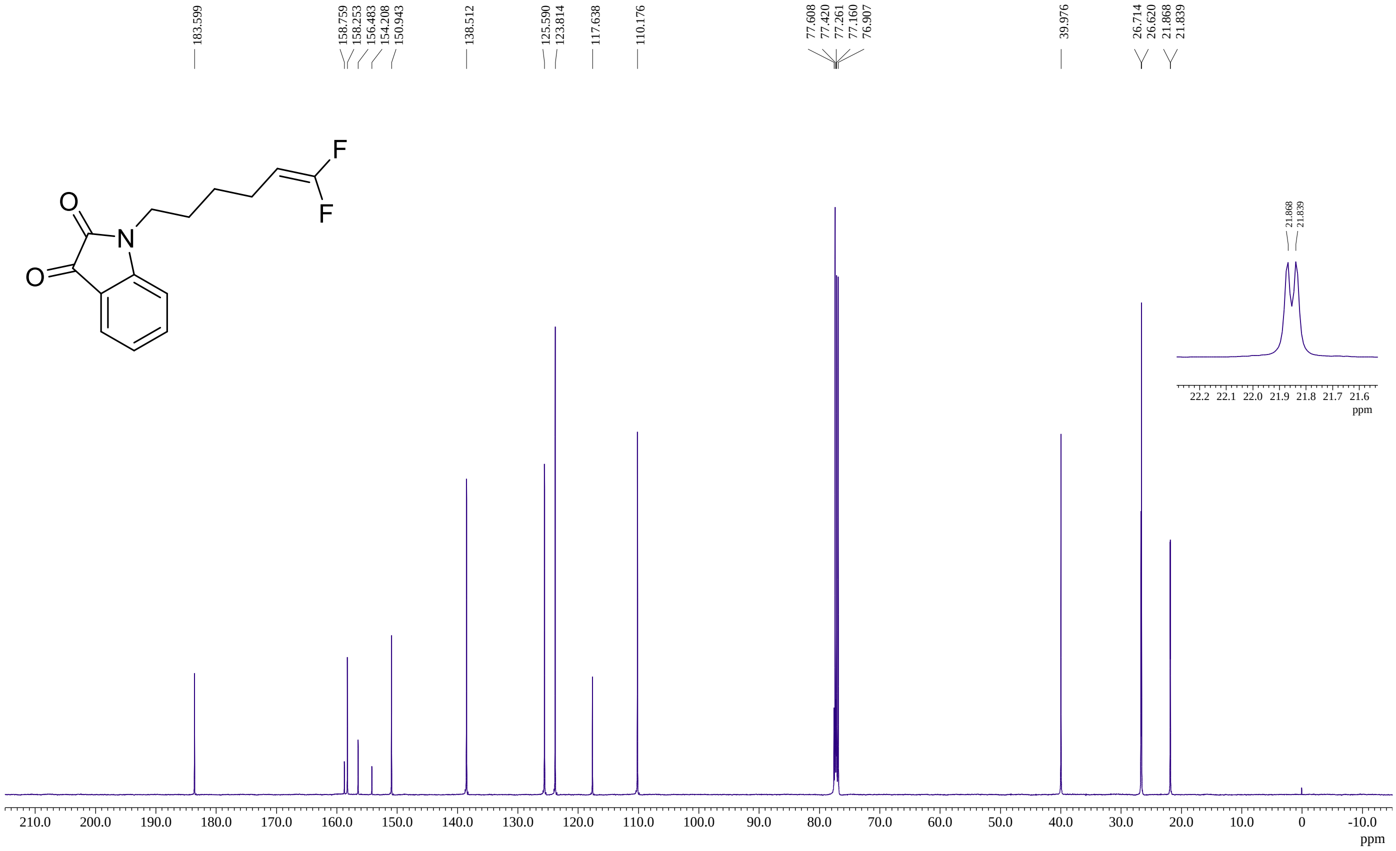

^{19}F NMR (282 MHz, $CDCl_3$): **1f**

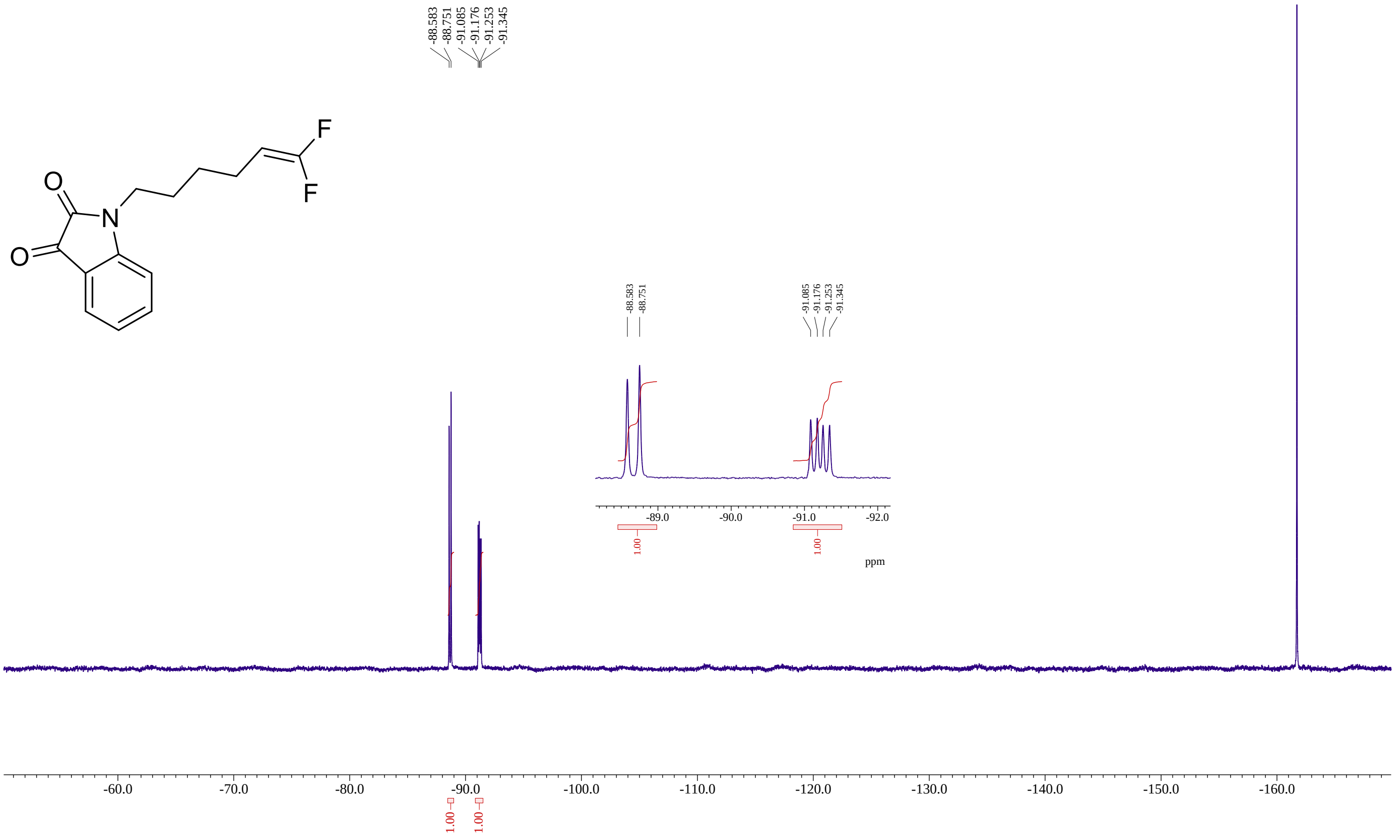

^{1}H NMR (500 MHz, $CDCl_3$): **1n**

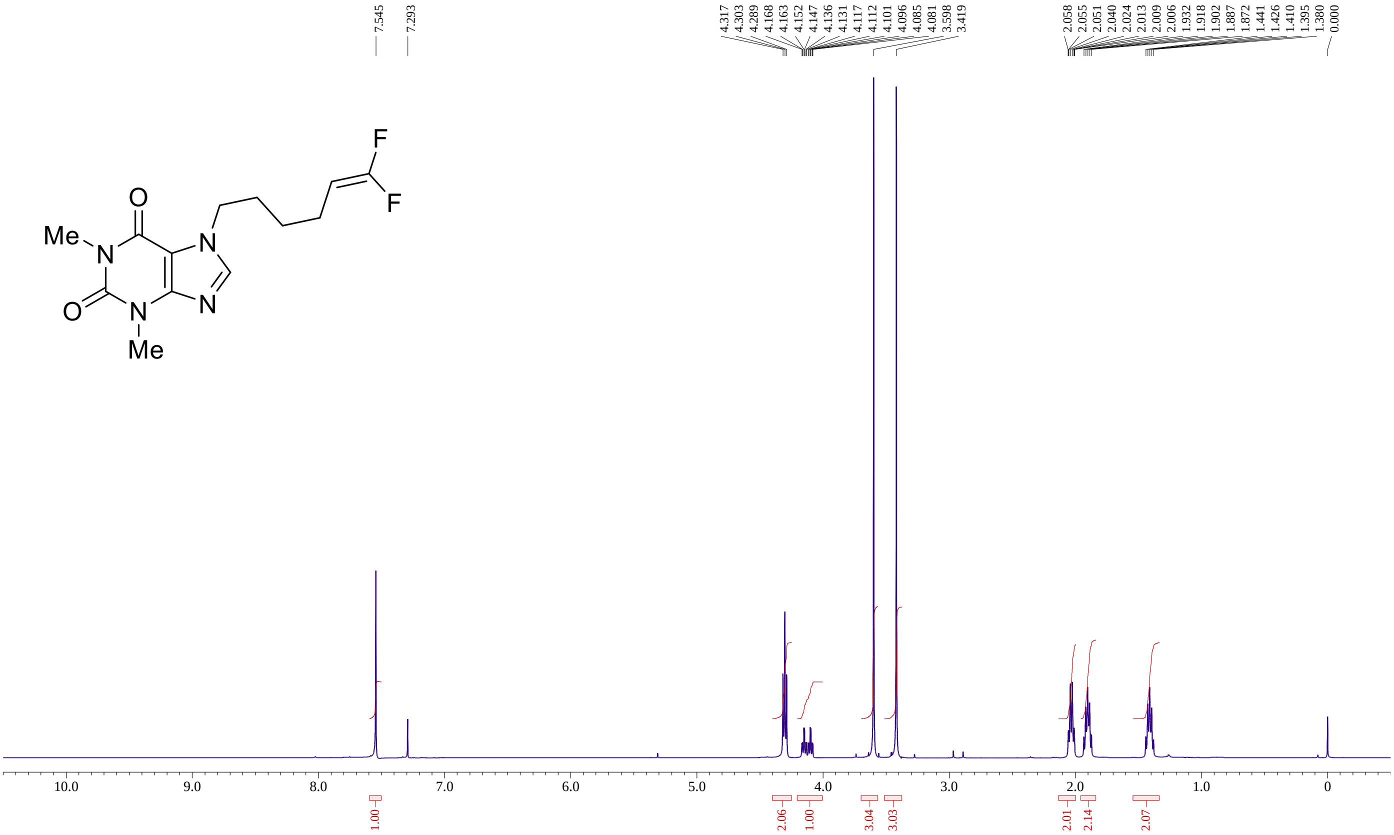

^{13}**C NMR** (126 MHz, $CDCl_3$): **1n**

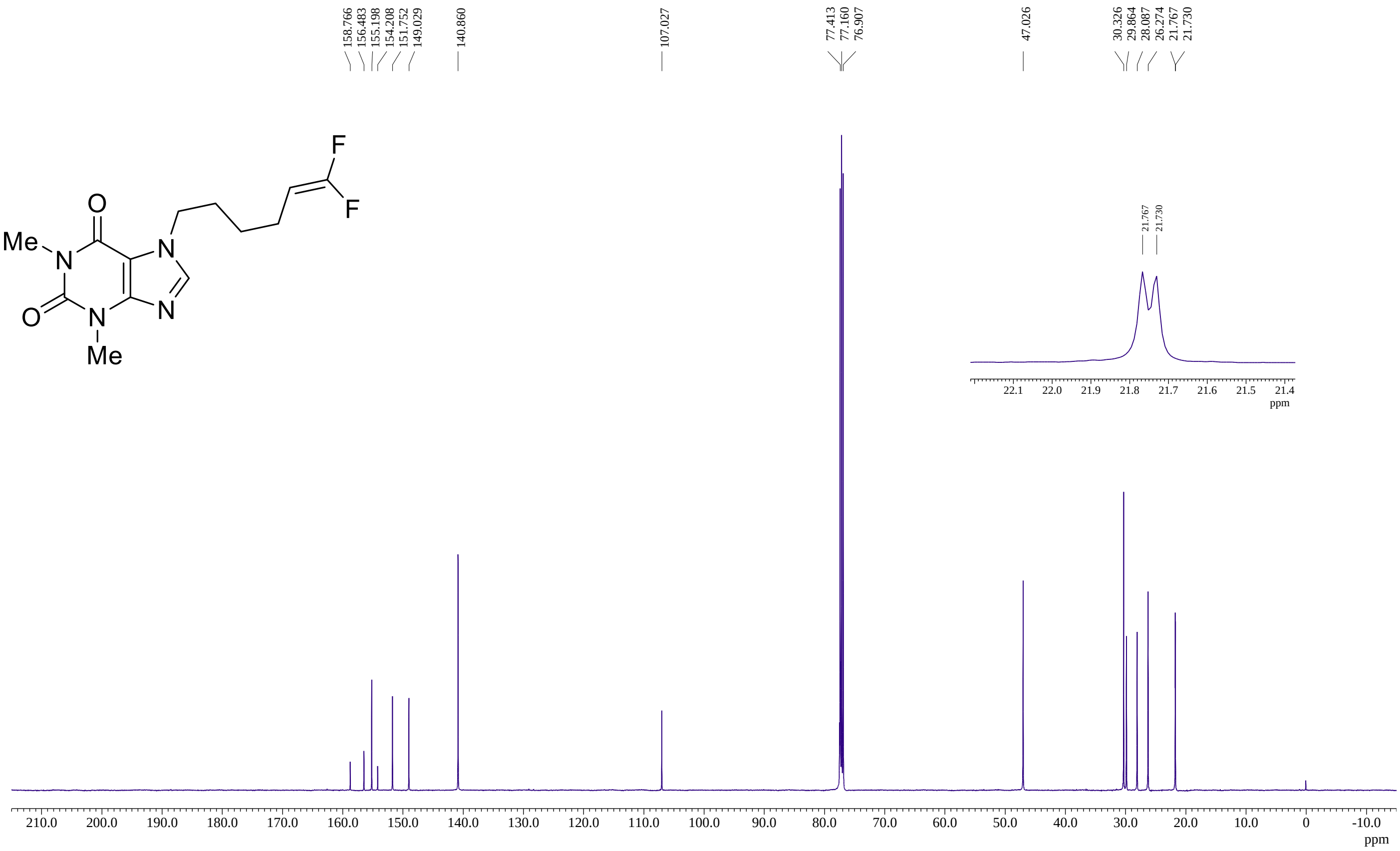

^{19}**F NMR** (282 MHz, $CDCl_3$): **1n**

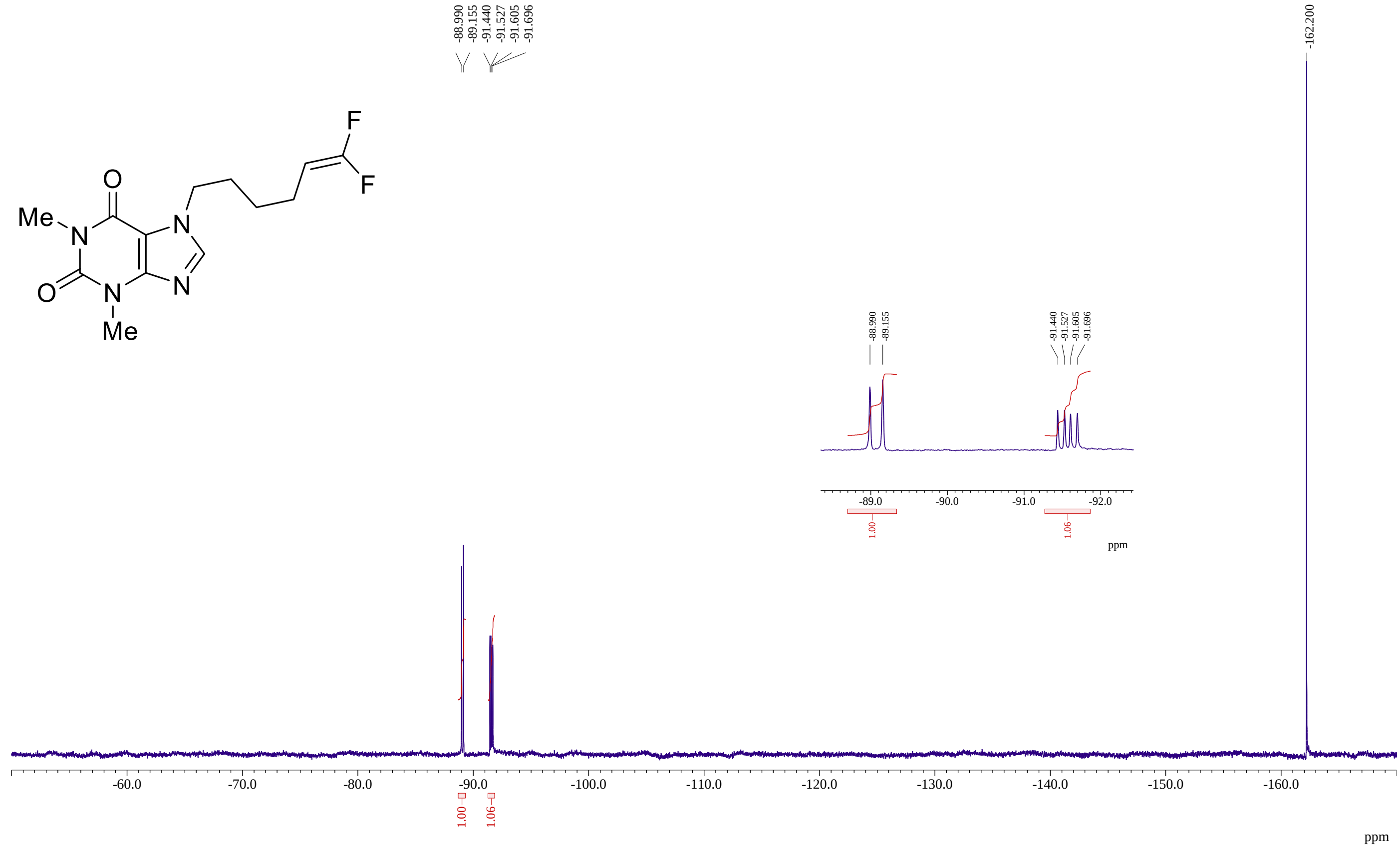

^{1}**H NMR** (500 MHz, $CDCl_3$): **1o**

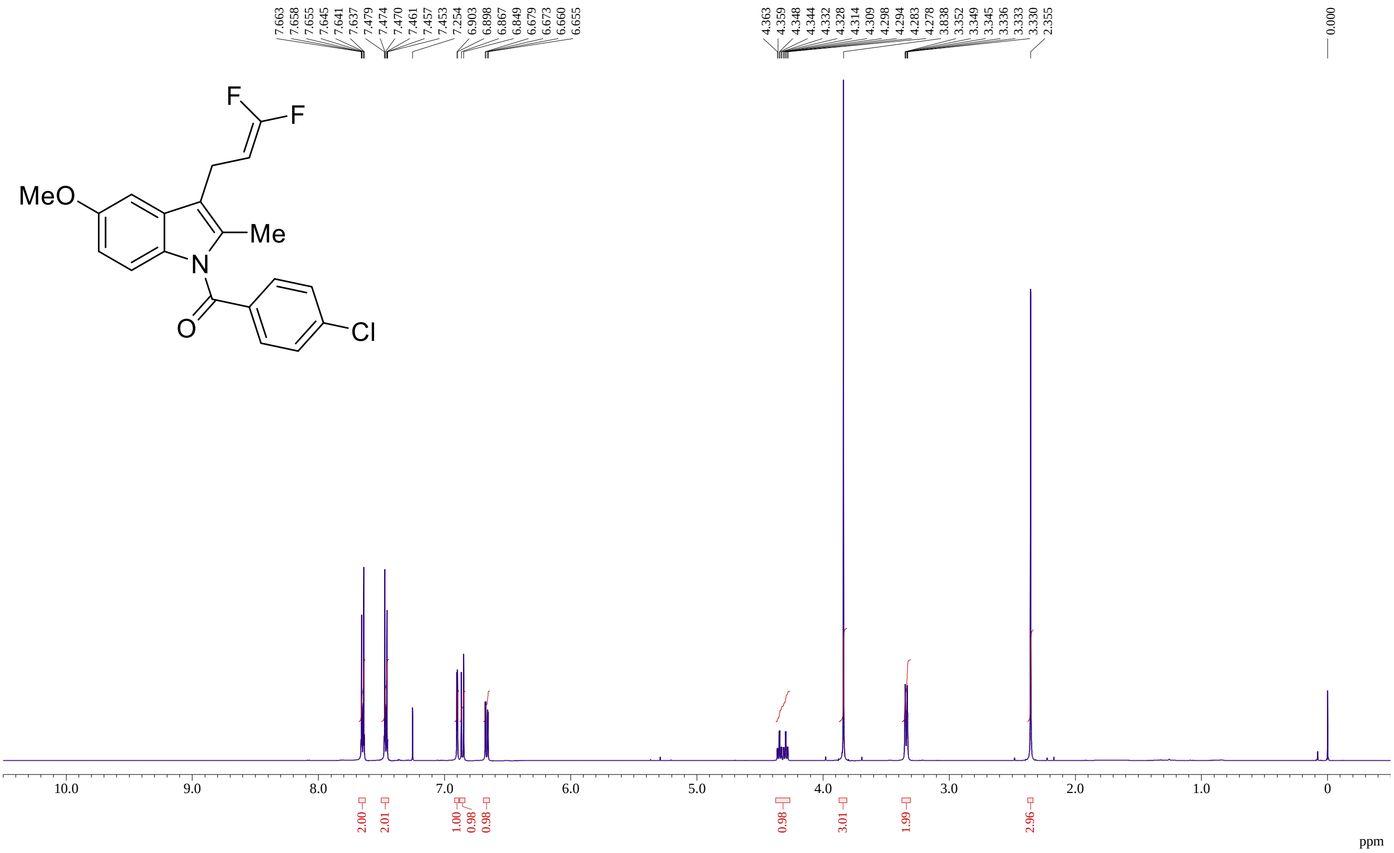

^{13}**C NMR** (126 MHz, $CDCl_3$): **1o**

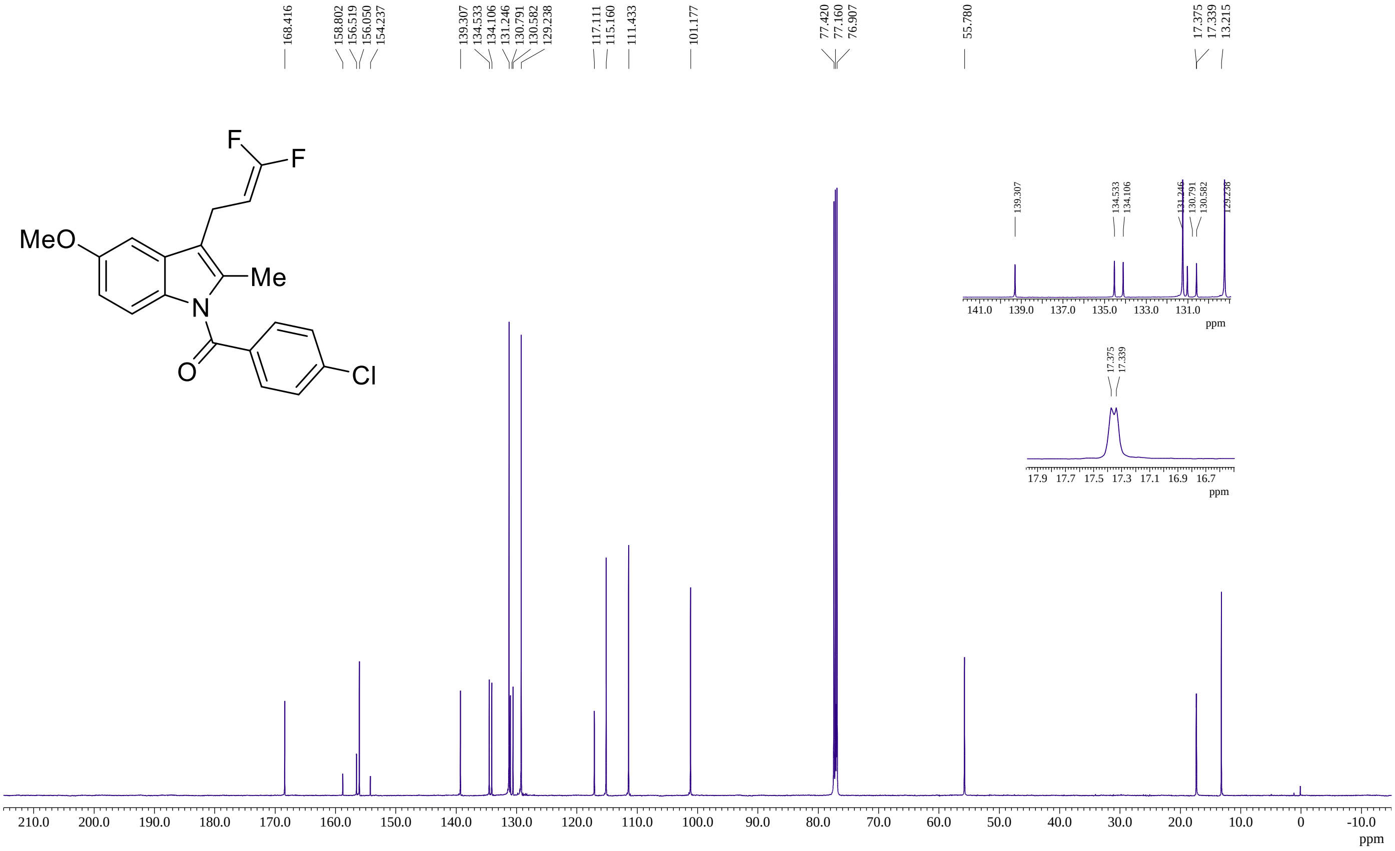

^{19}F NMR (282 MHz, $CDCl_3$): **1o**

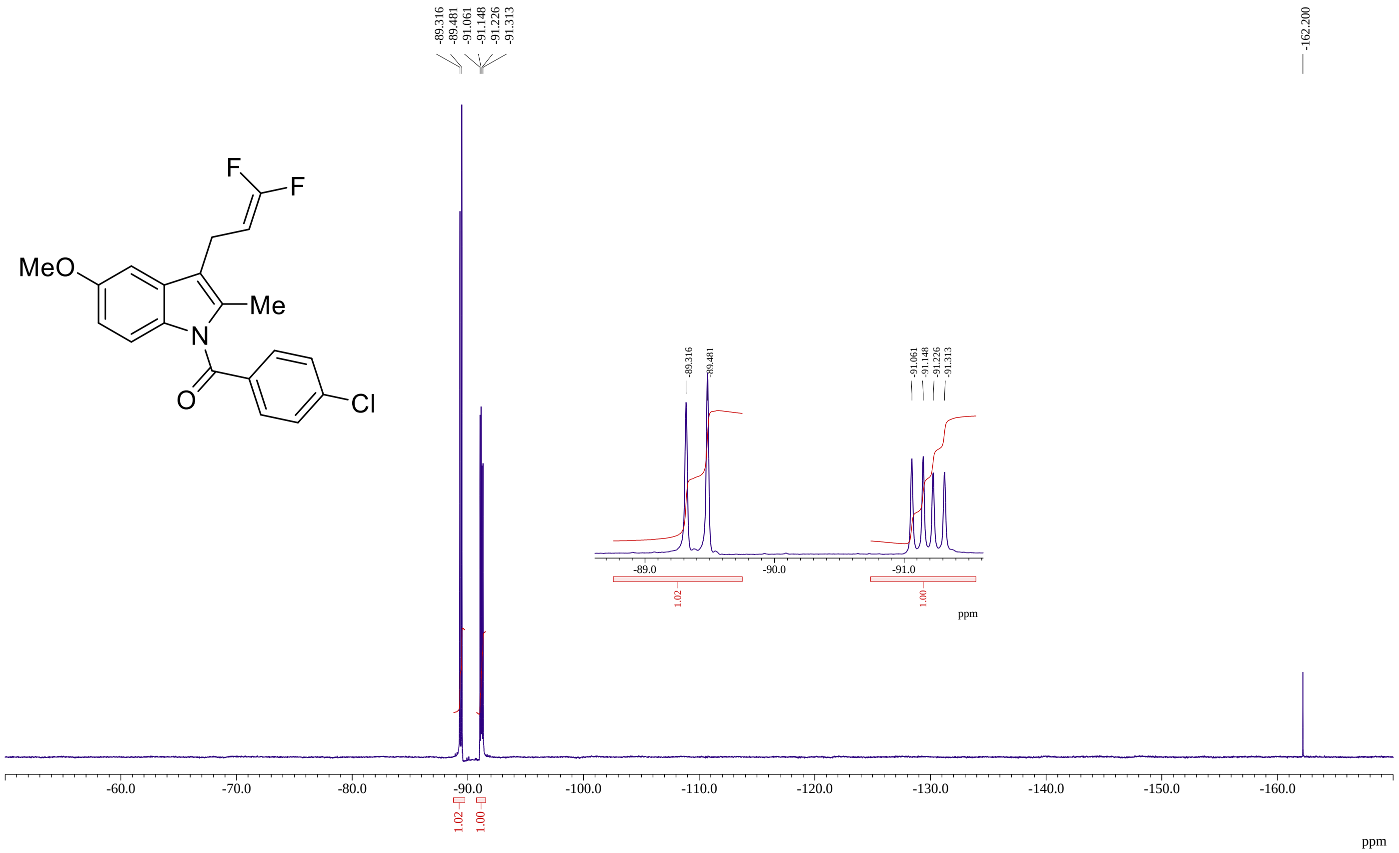

^{1}H NMR (500 MHz, $CDCl_3$): **1r**

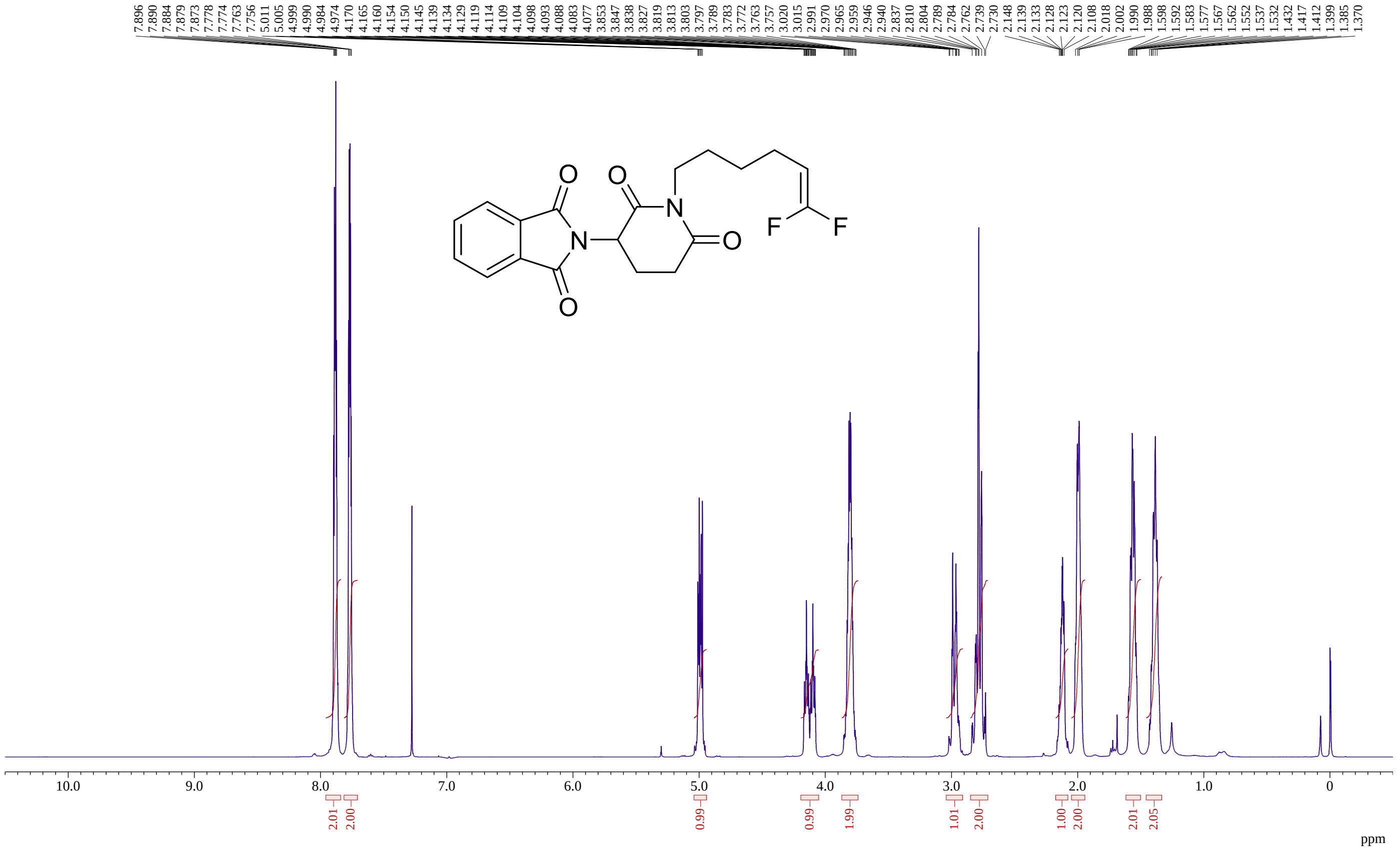

^{13}C NMR (126 MHz, $CDCl_3$): **1r**

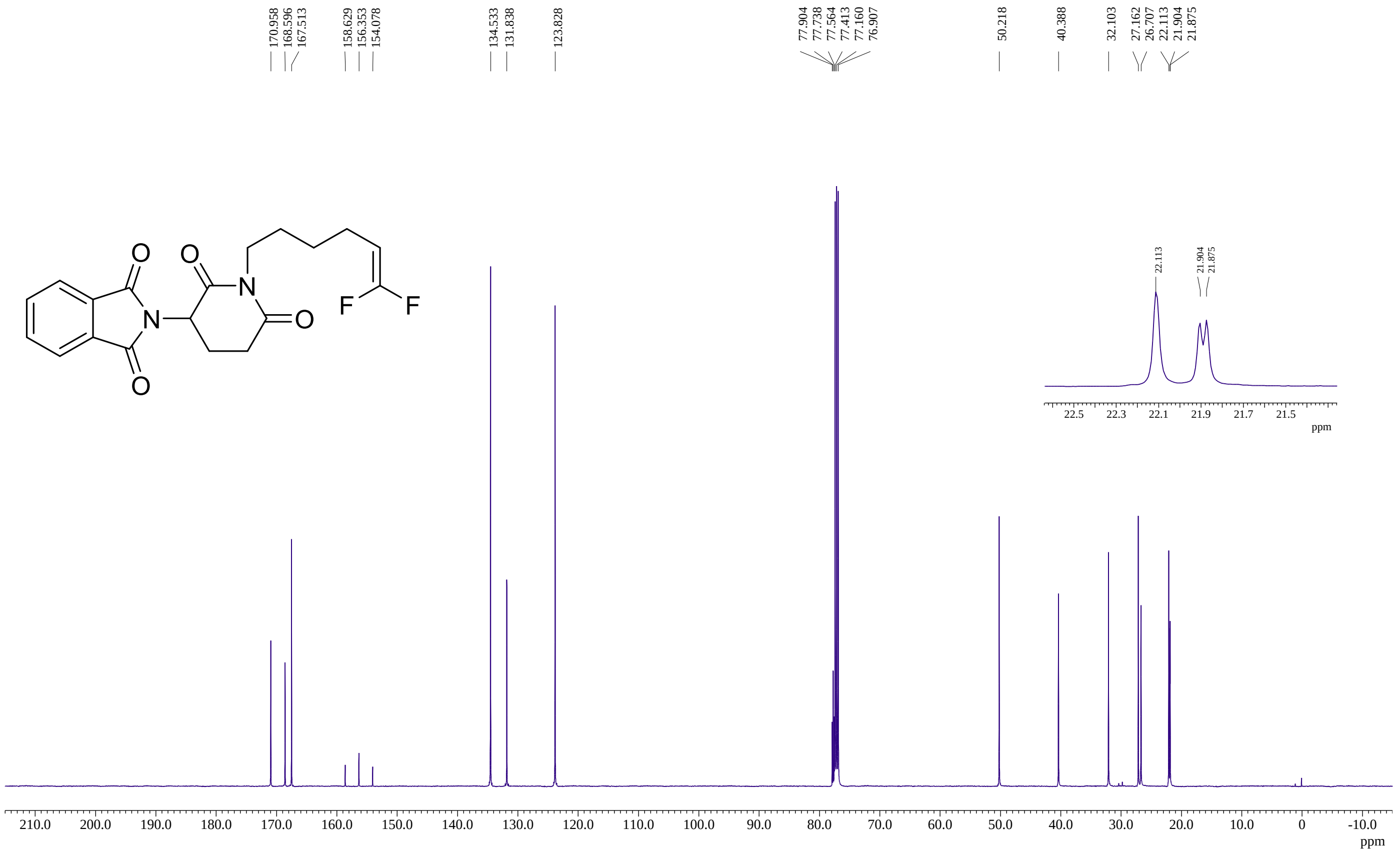

^{19}F NMR (282 MHz, $CDCl_3$): **1r**

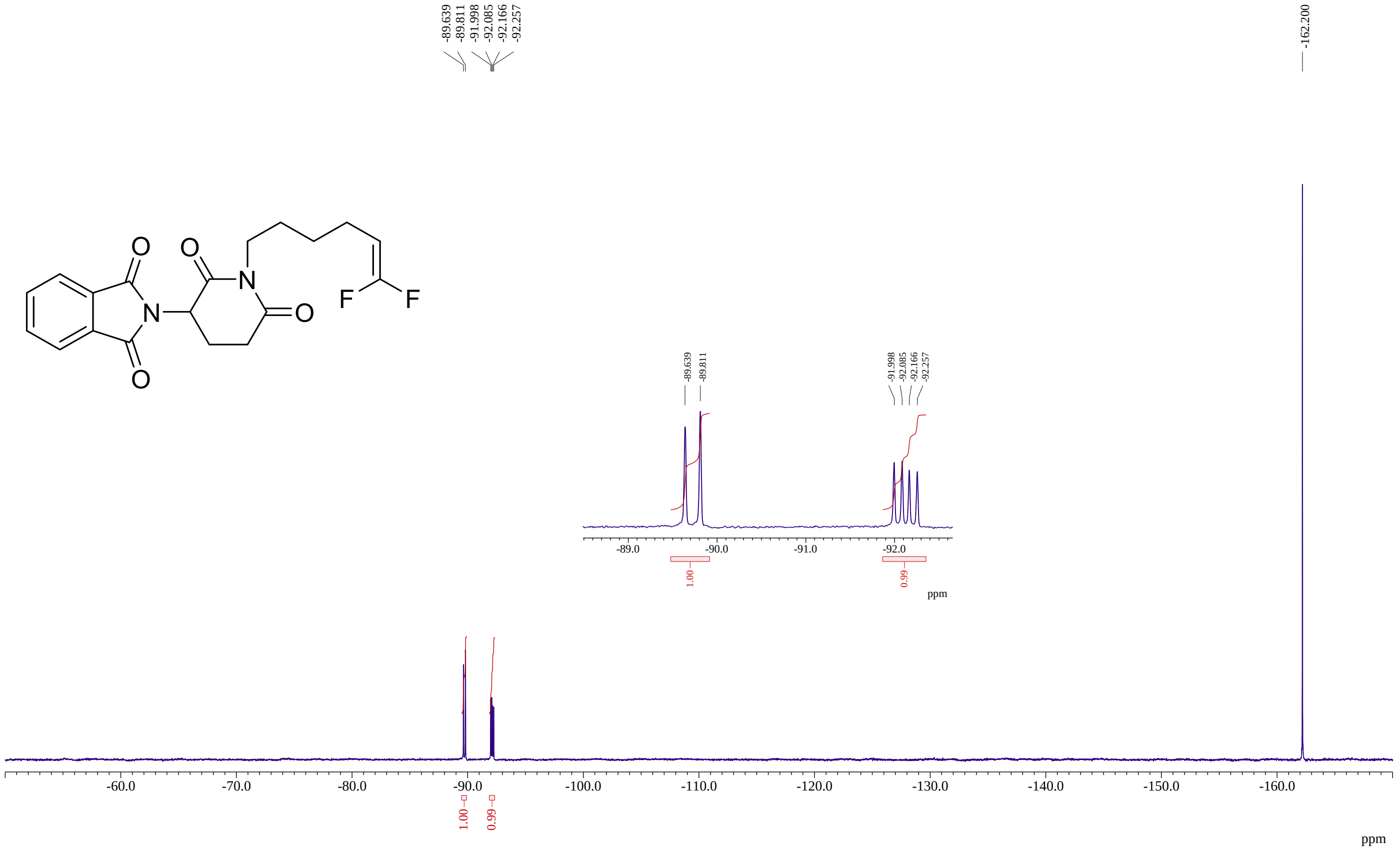

^{1}H NMR (500 MHz, $CDCl_3$): **3aa**

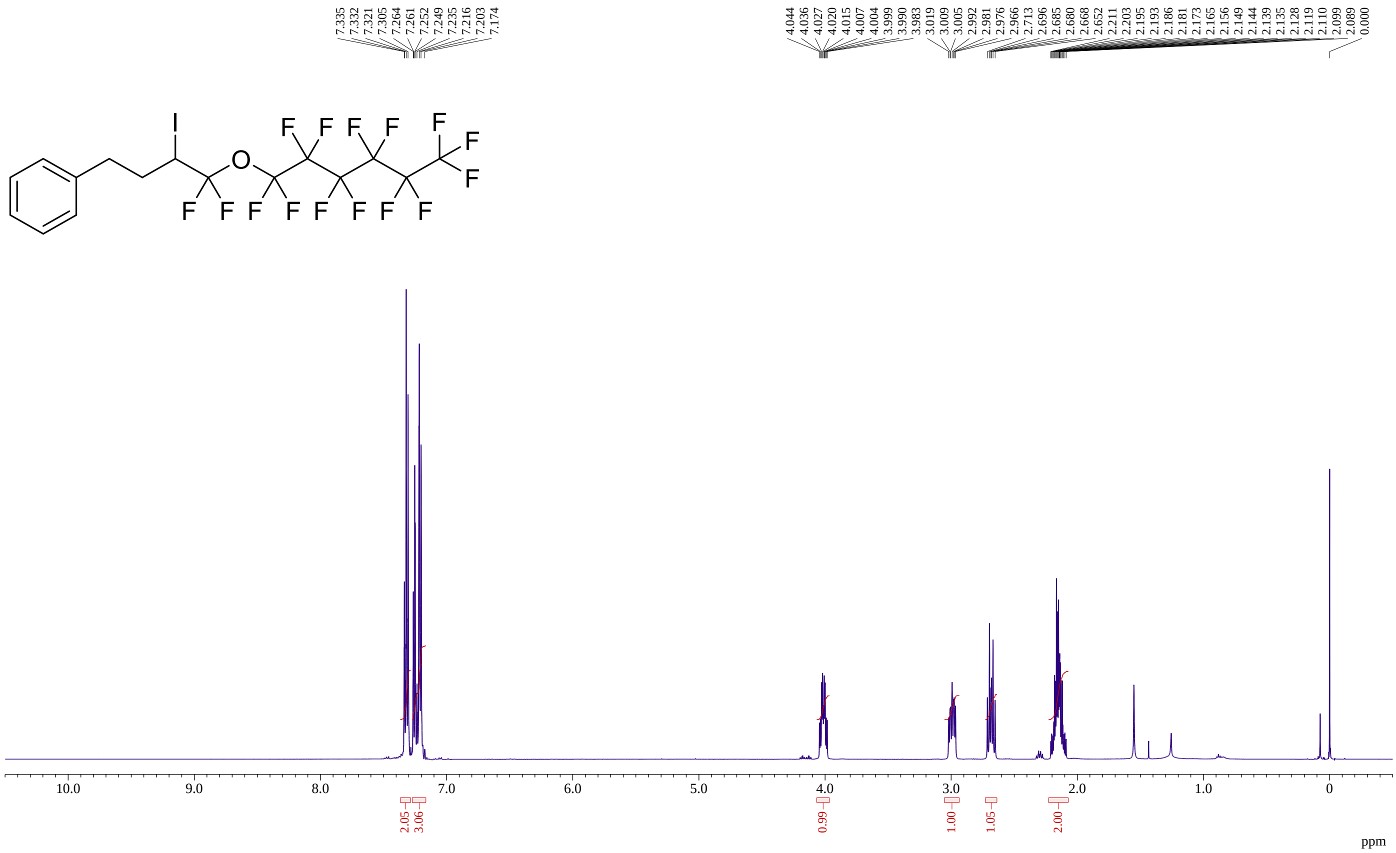

^{13}C NMR (126 MHz, $CDCl_3$): **3aa**

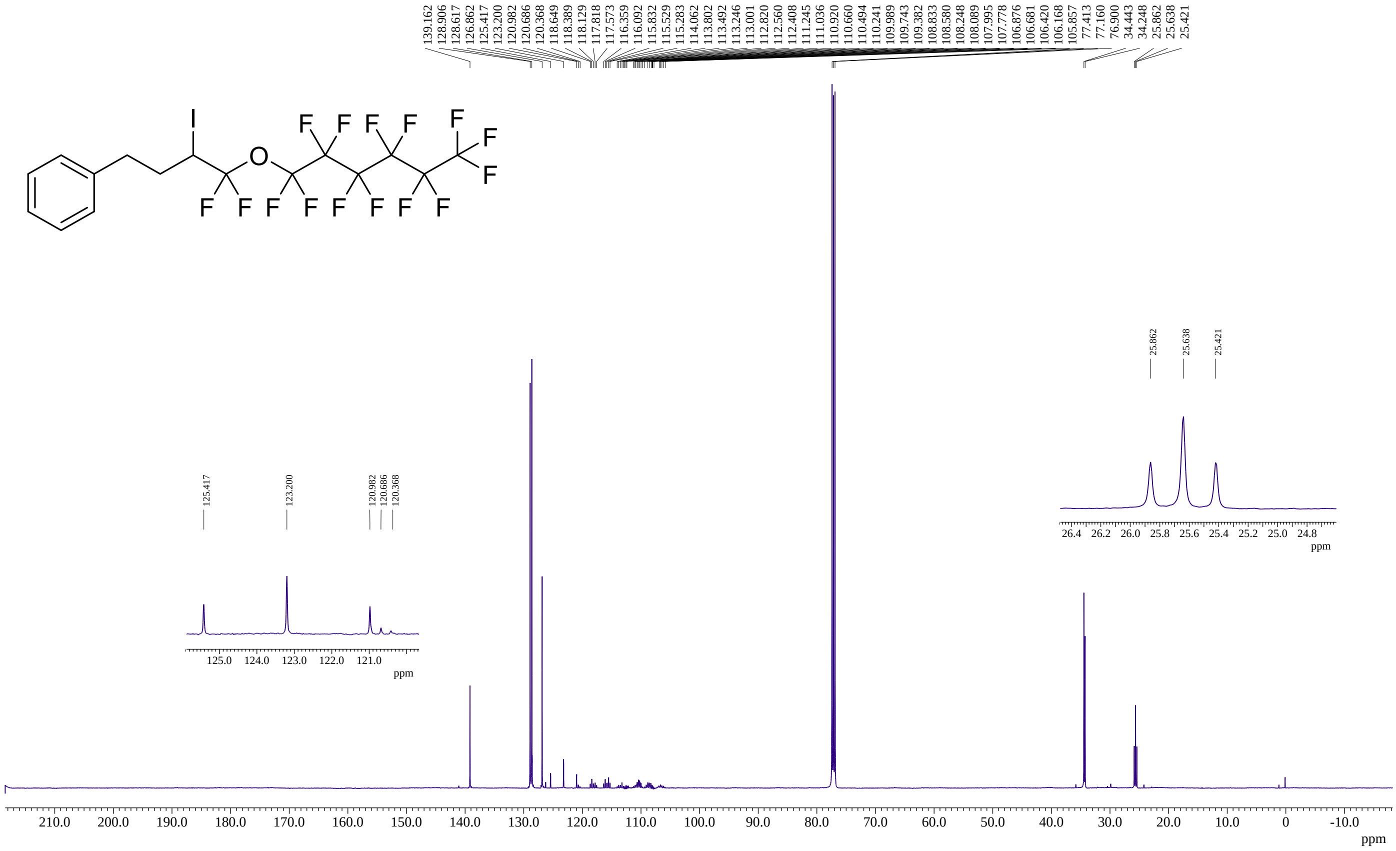

^{19}F NMR (282 MHz, $CDCl_3$): **3aa**

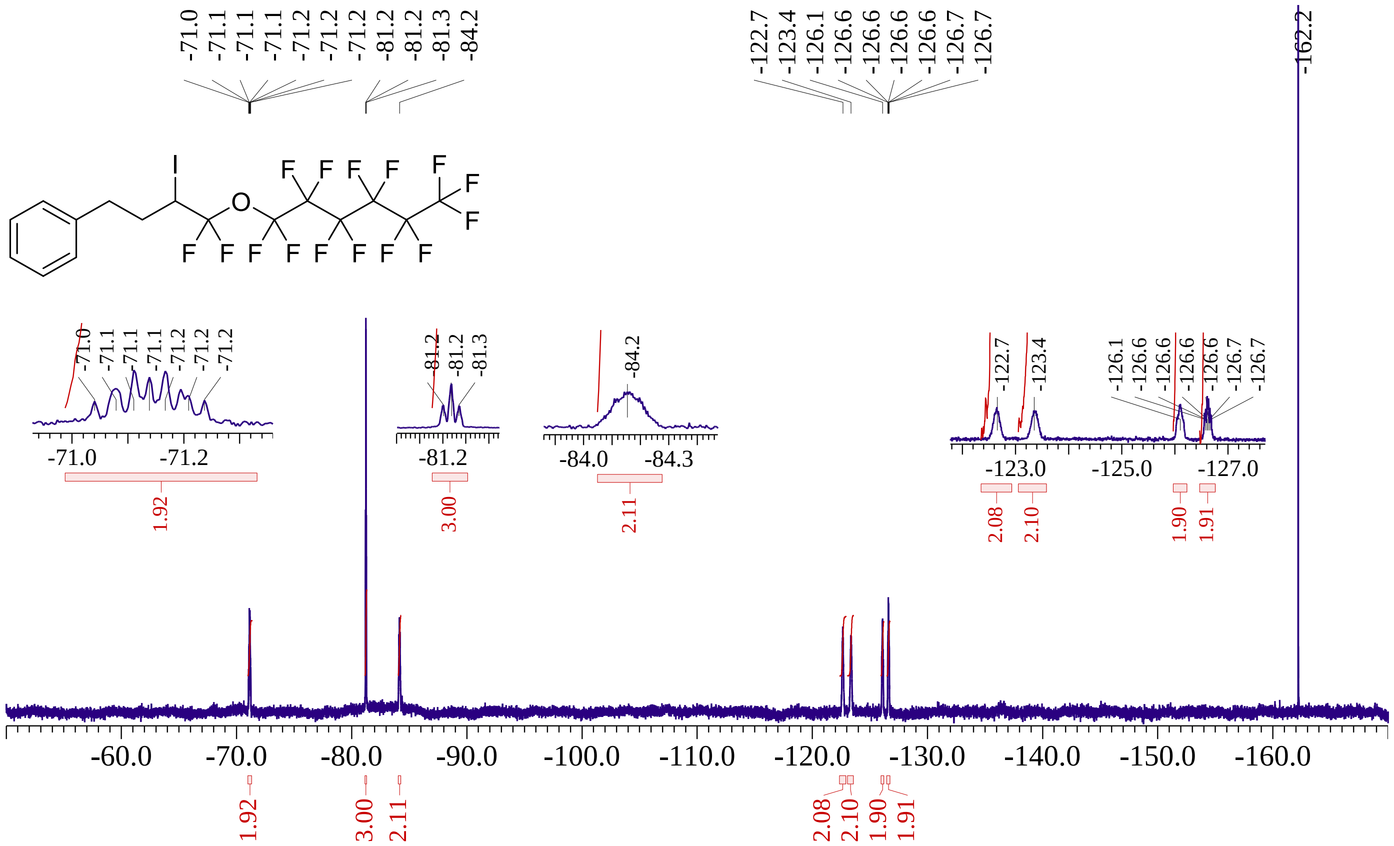

^{1}H NMR (500 MHz, $CDCl_3$): **3ba**

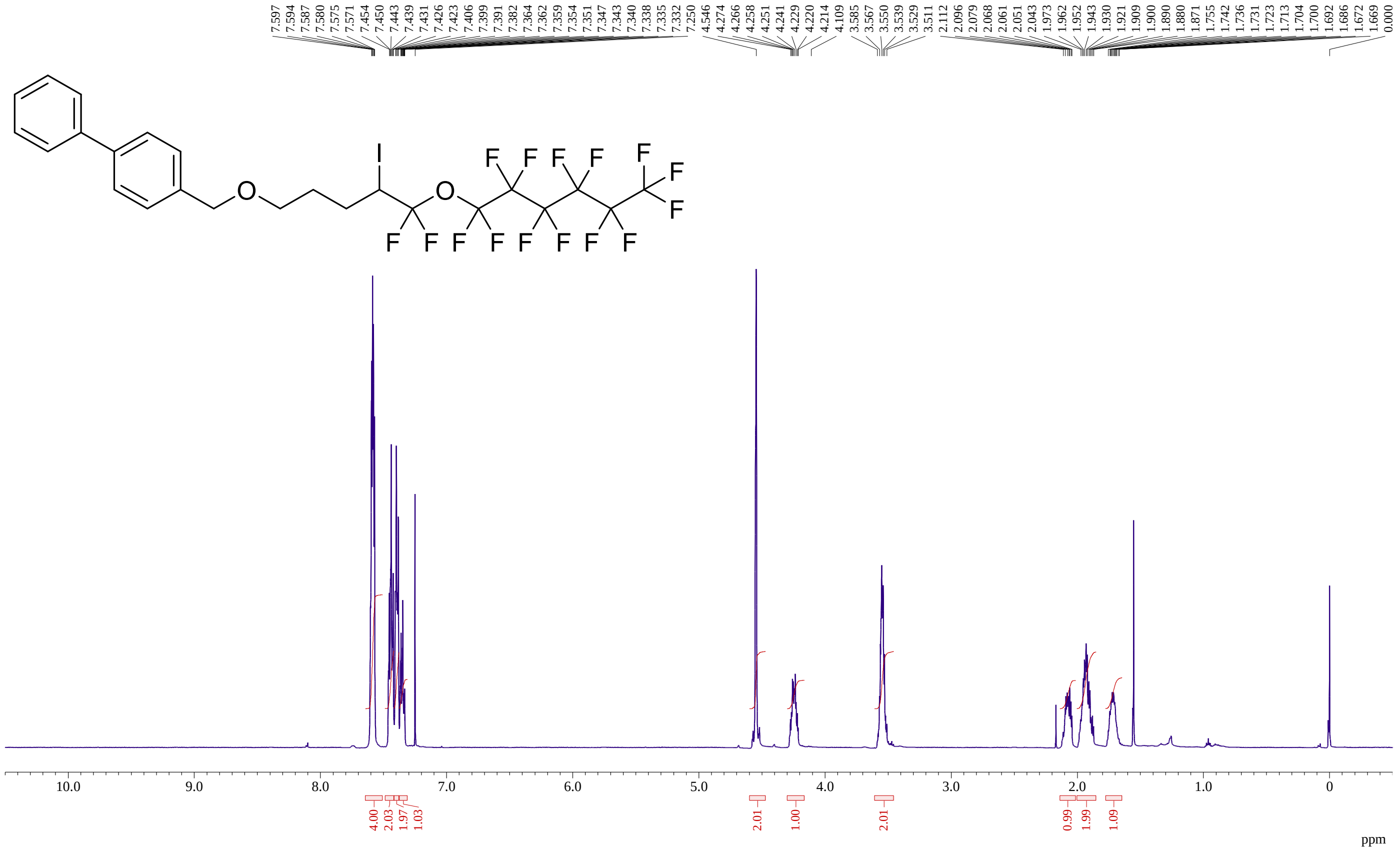

^{13}**C NMR** (126 MHz, $CDCl_3$): **3ba**

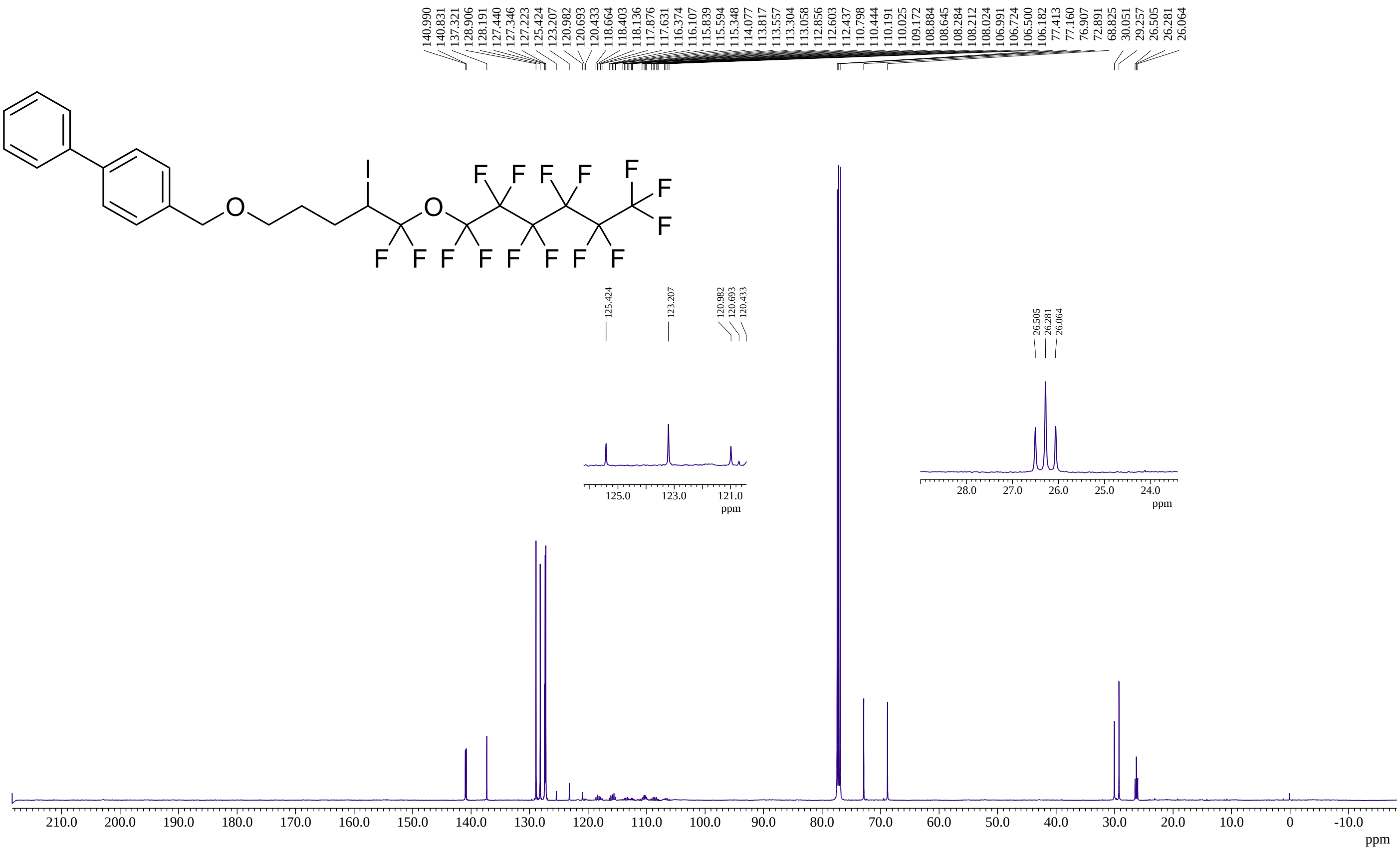

^{19}**F NMR** (282 MHz, $CDCl_3$): **3ba**

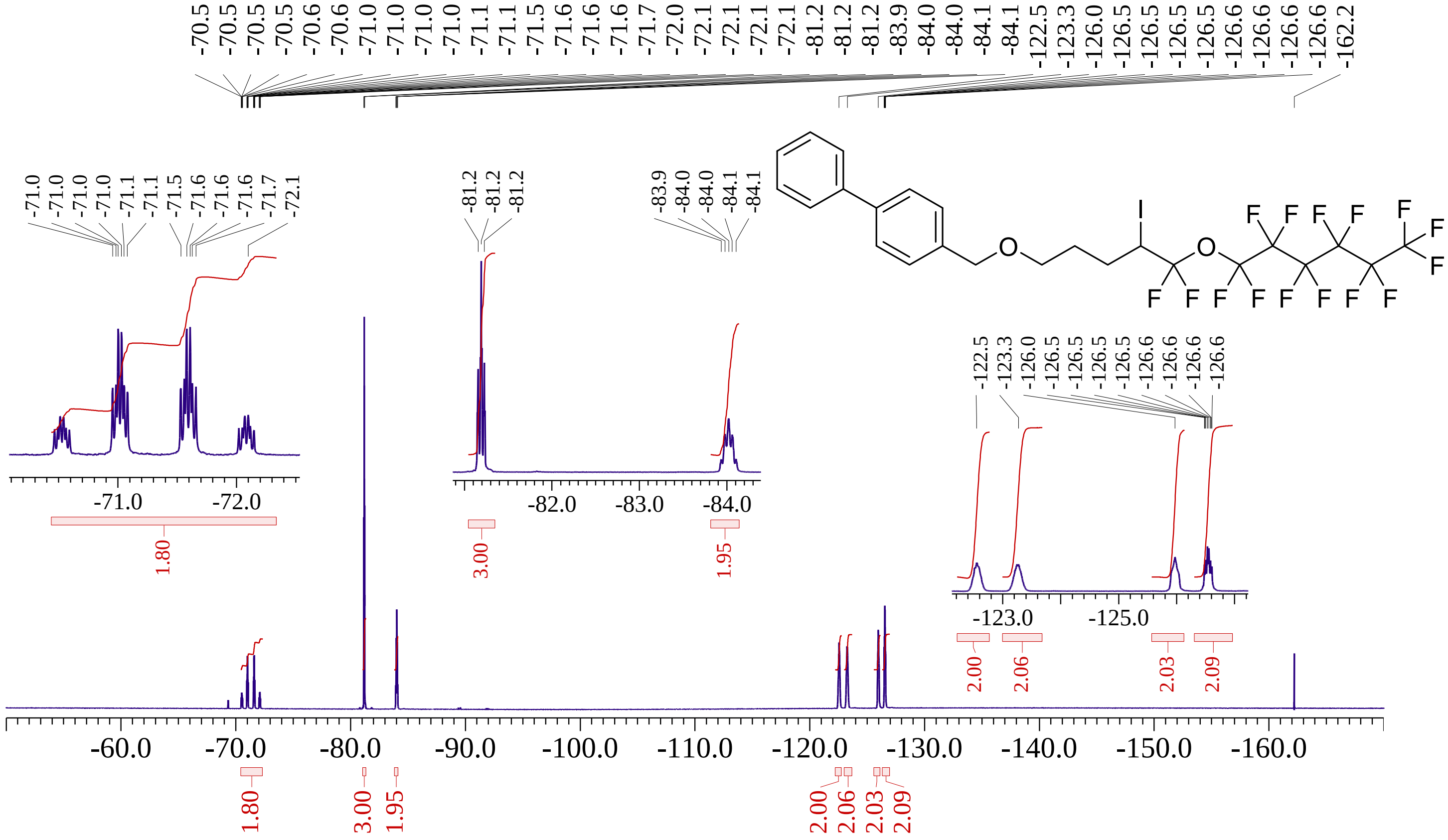

^{1}H NMR (500 MHz, $CDCl_3$): **3ca**

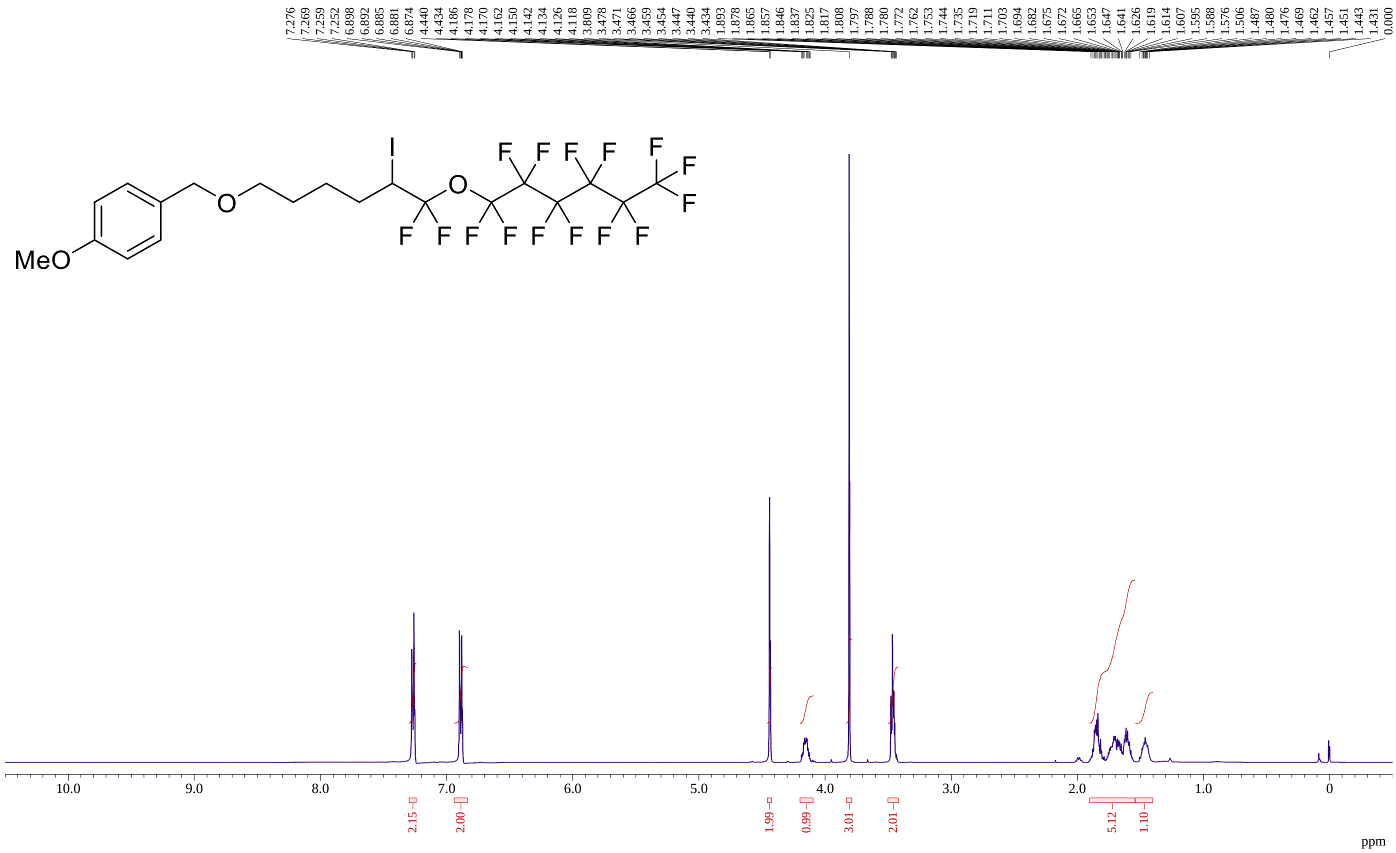

^{13}C NMR (126 MHz, $CDCl_3$): **3ca**

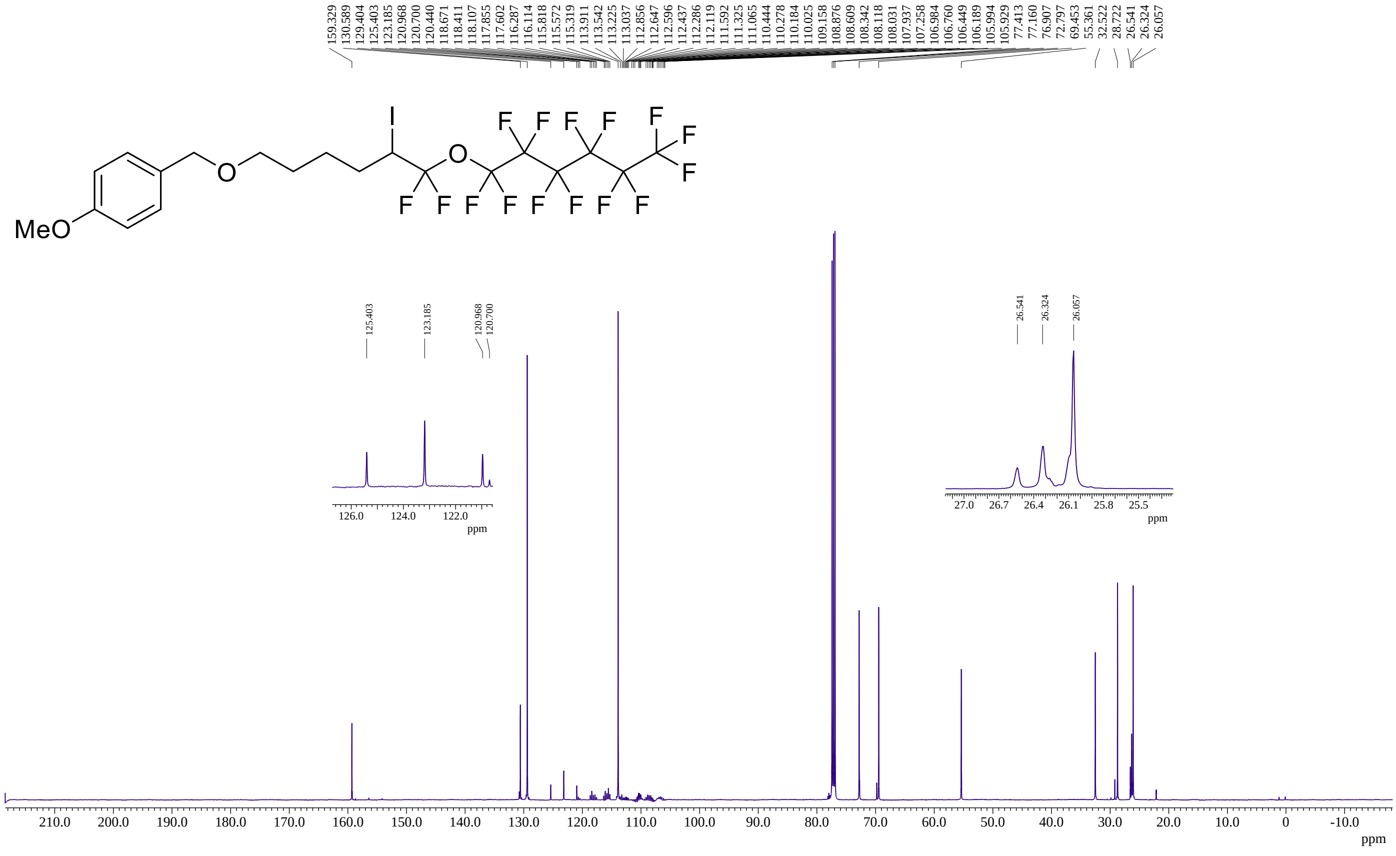

^{19}F NMR (282 MHz, CDCl$_3$): **3ca**

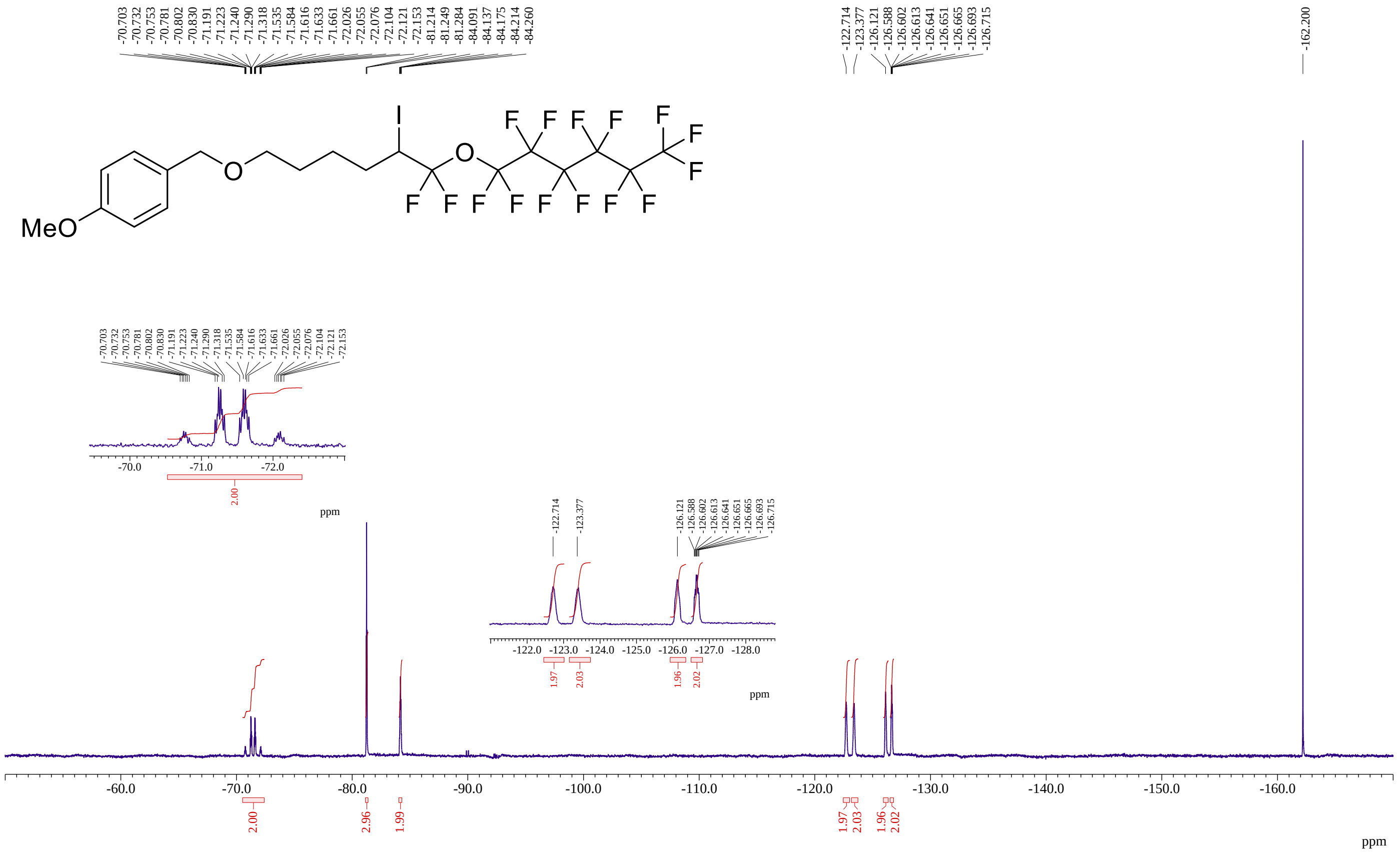

^{1}H NMR (500 MHz, CDCl$_3$): **3da**

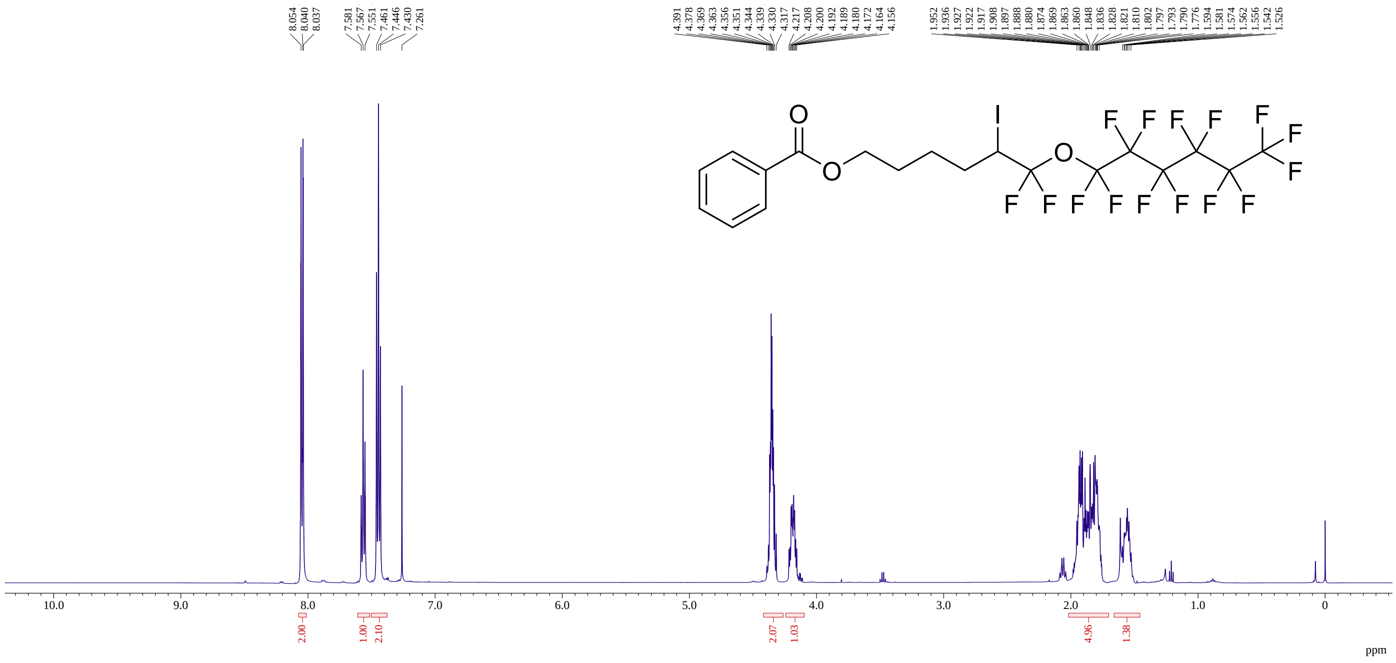

^{13}C NMR (126 MHz, $CDCl_3$):**3da**

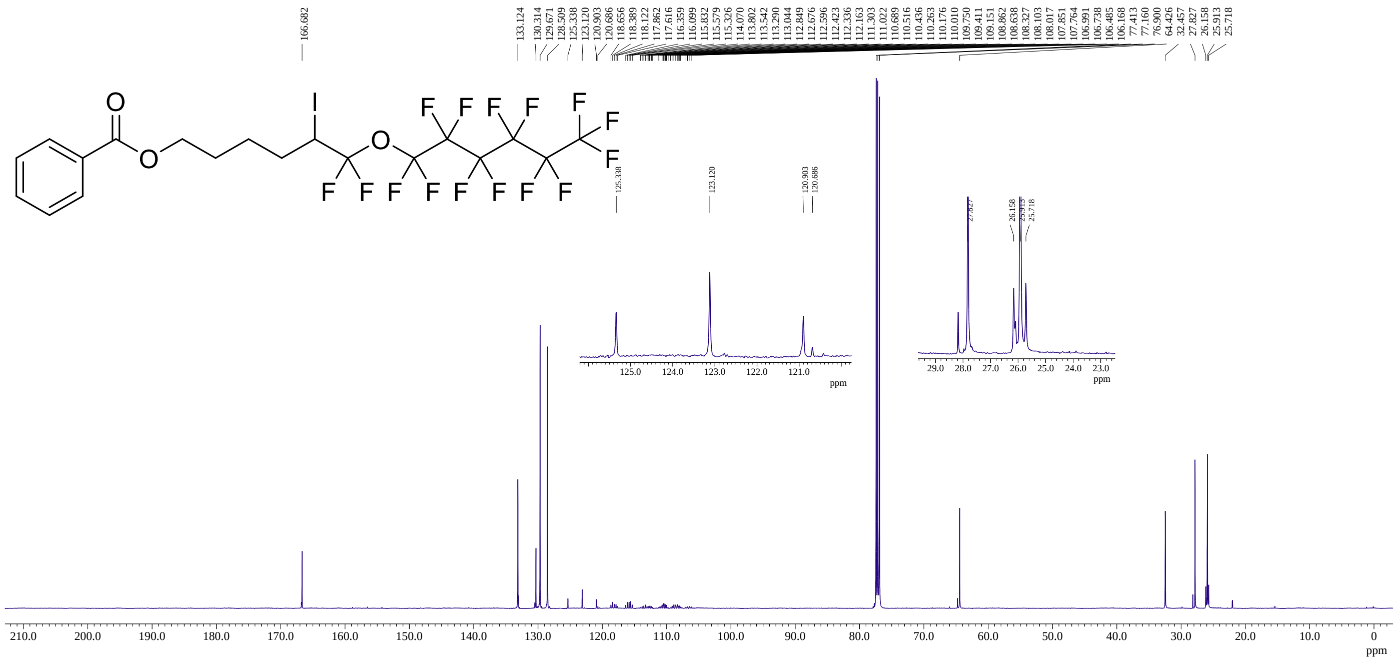

^{19}F NMR (282 MHz, $CDCl_3$): **3da**

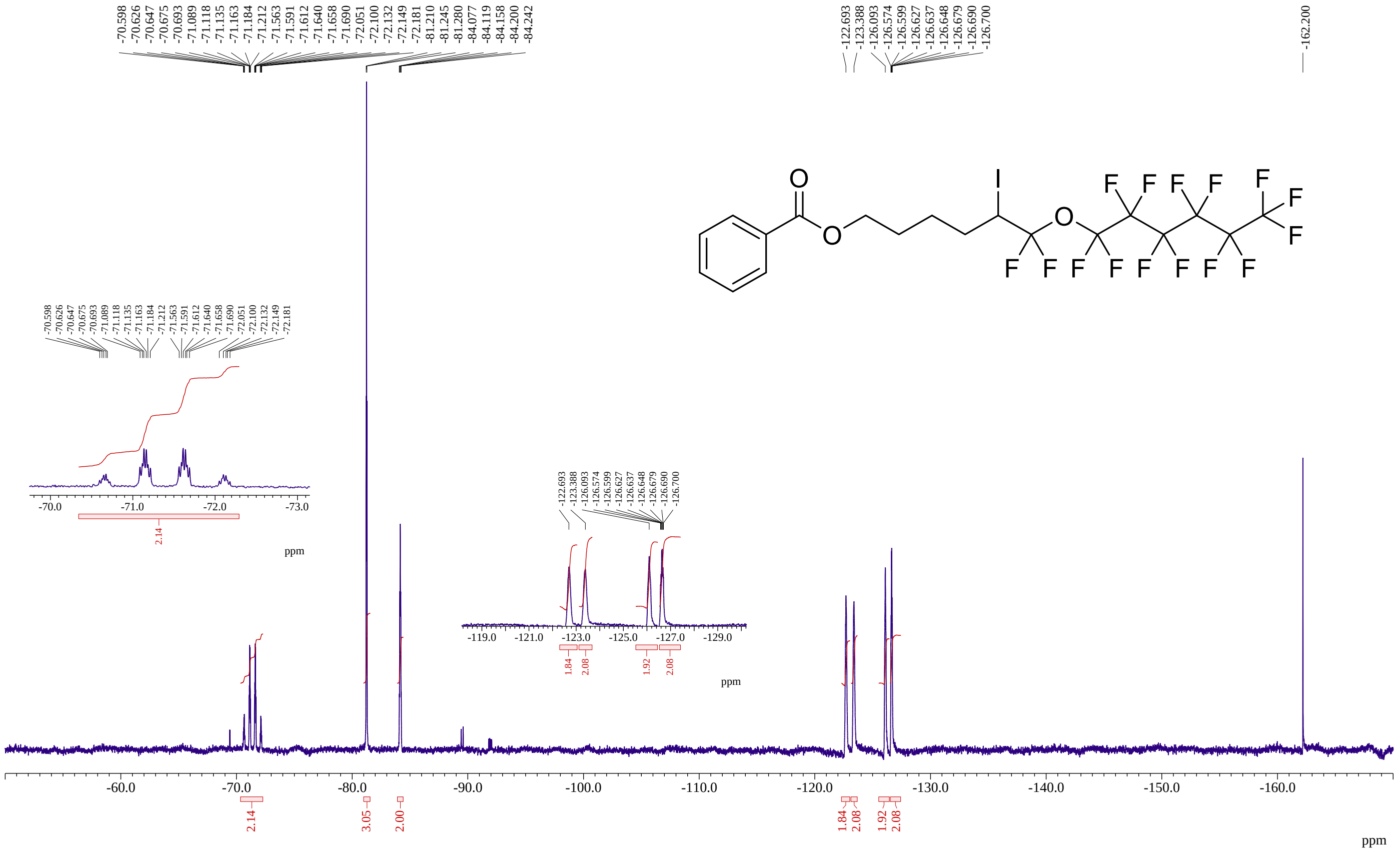

^{1}H NMR (500 MHz, $CDCl_3$): **3ea**

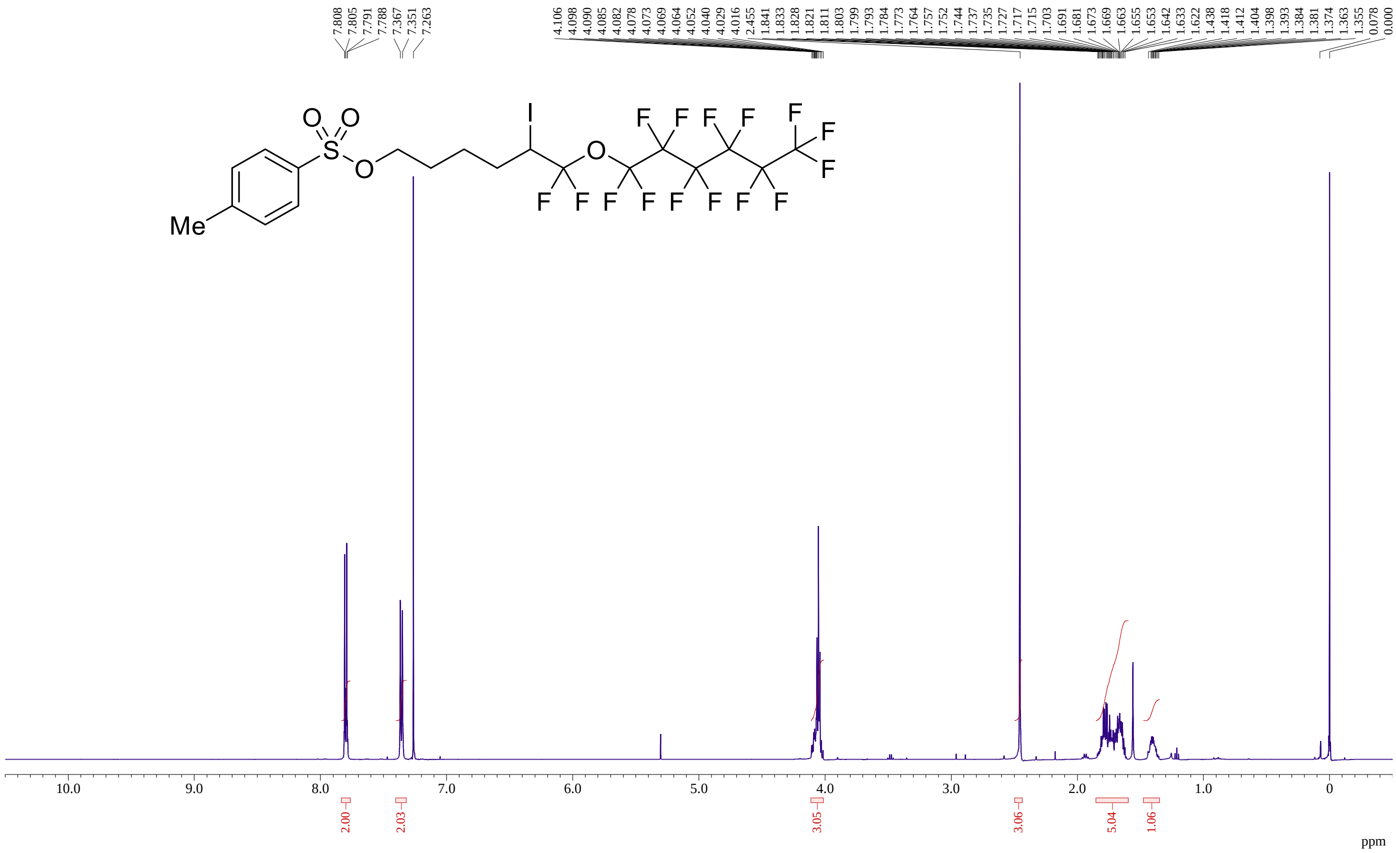

^{13}C NMR (126 MHz, $CDCl_3$): **3ea**

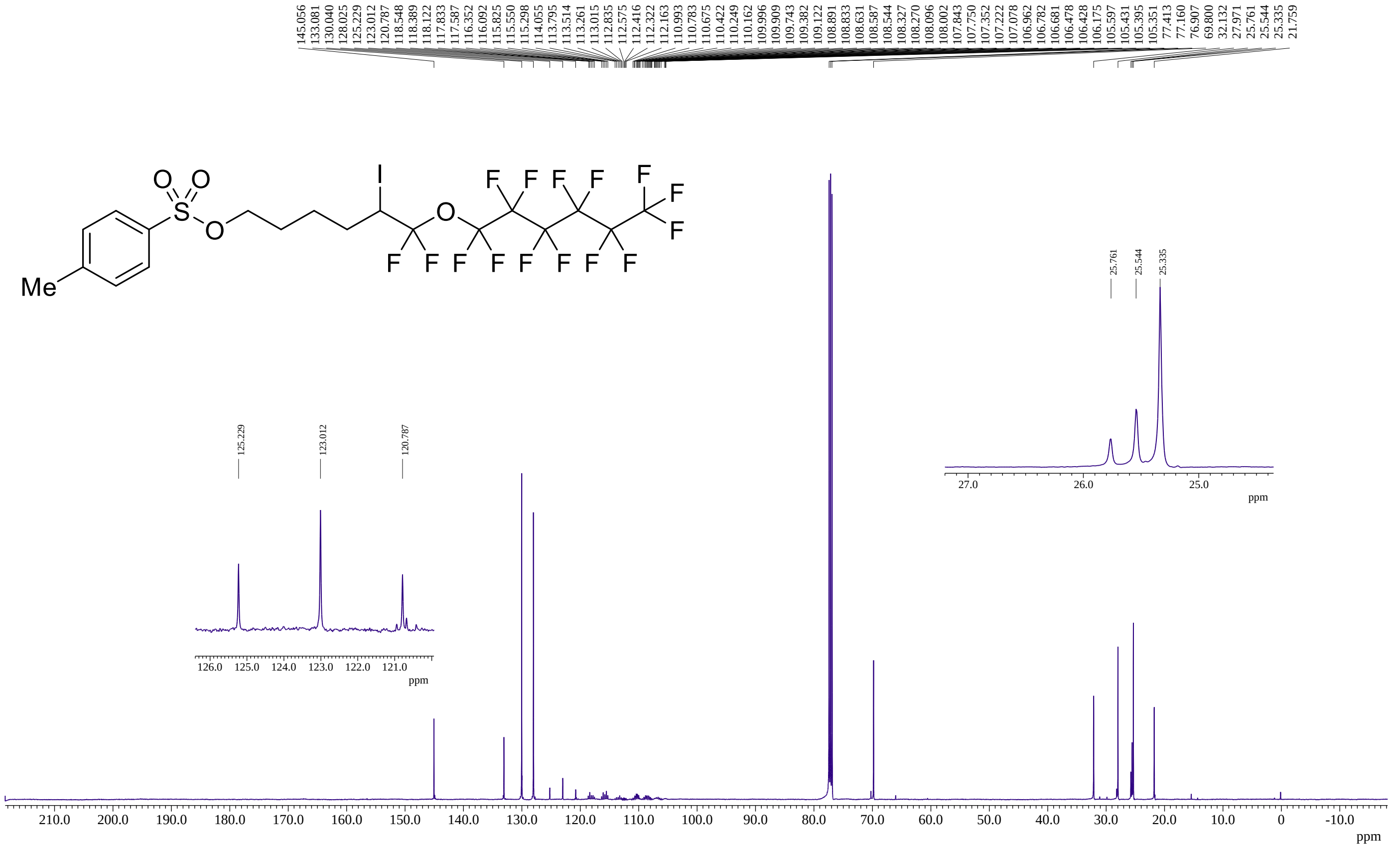

^{19}F NMR (282 MHz, CDCl$_3$): **3ea**

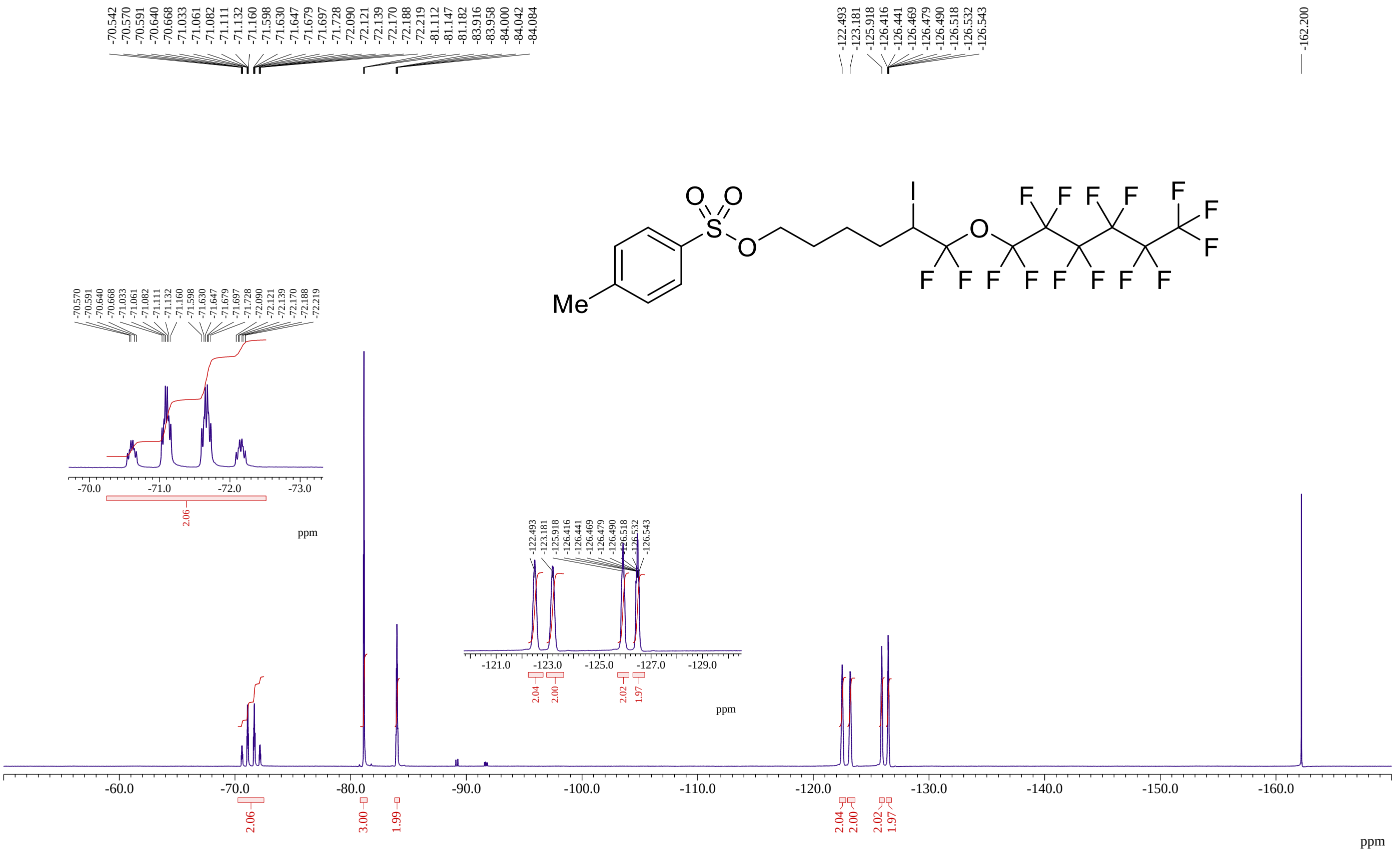

^{1}H NMR (500 MHz, CDCl$_3$): **3fa**

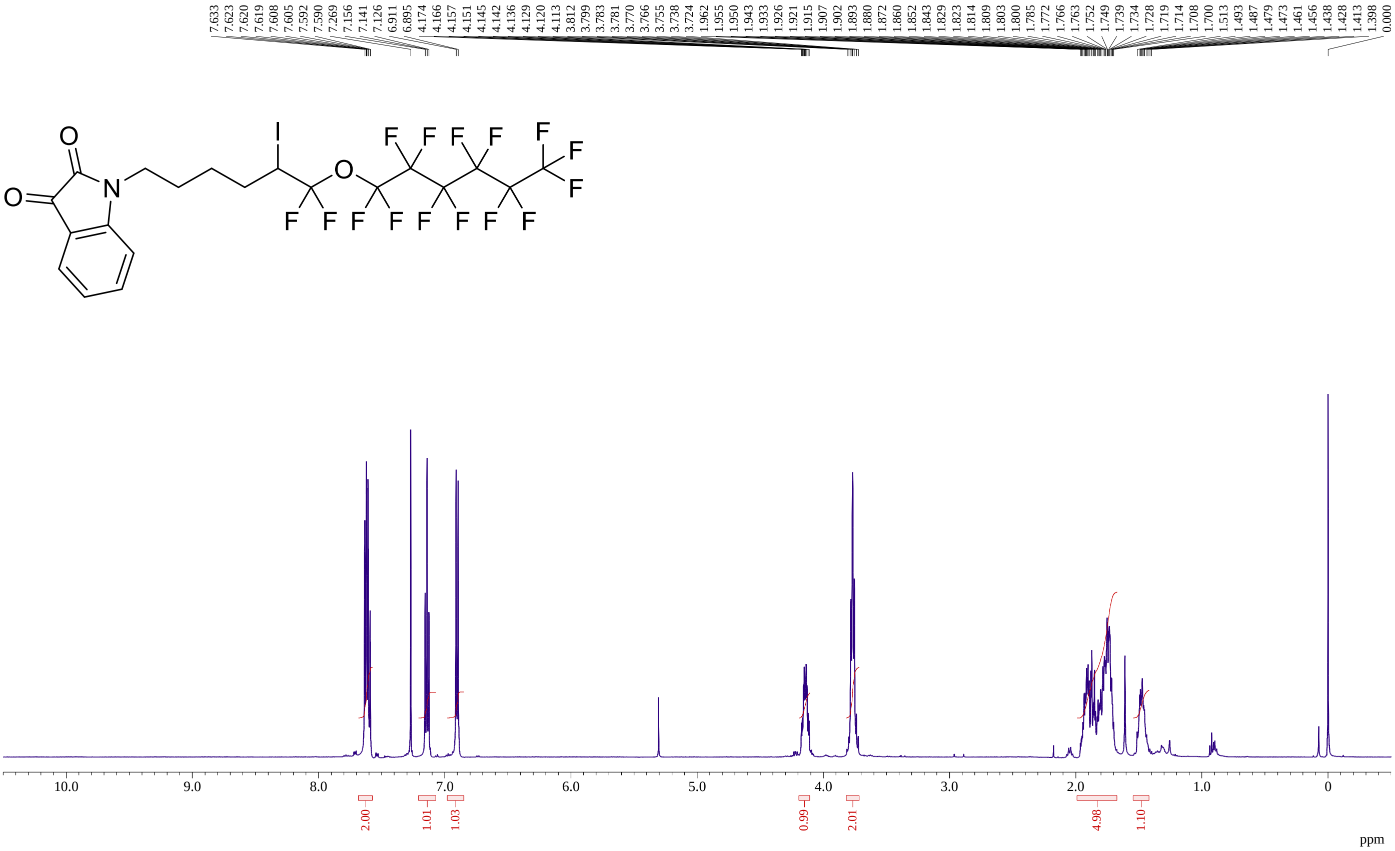

^{13}C NMR (126 MHz, $CDCl_3$): **3fa**

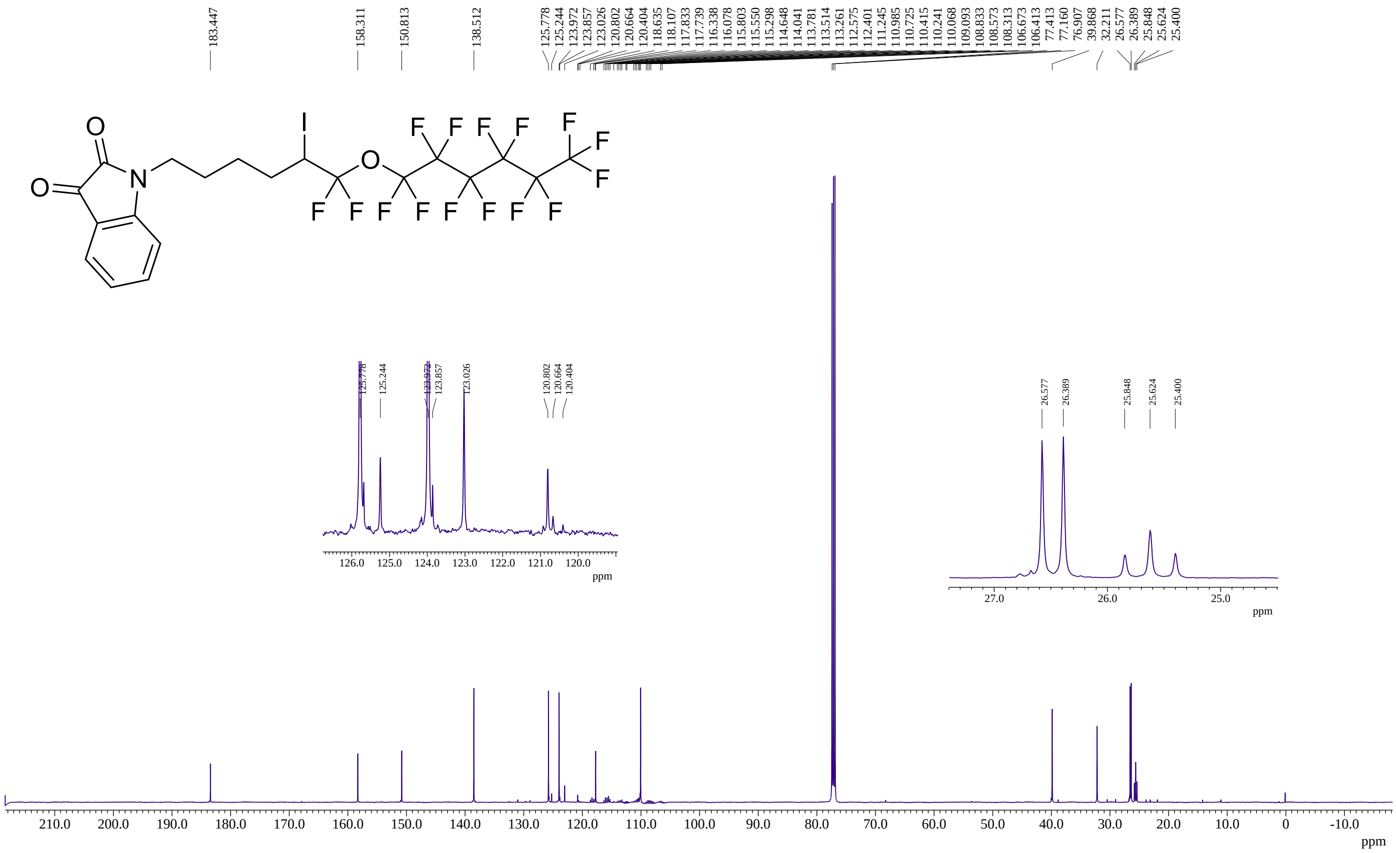

^{19}F NMR (282 MHz, $CDCl_3$): **3fa**

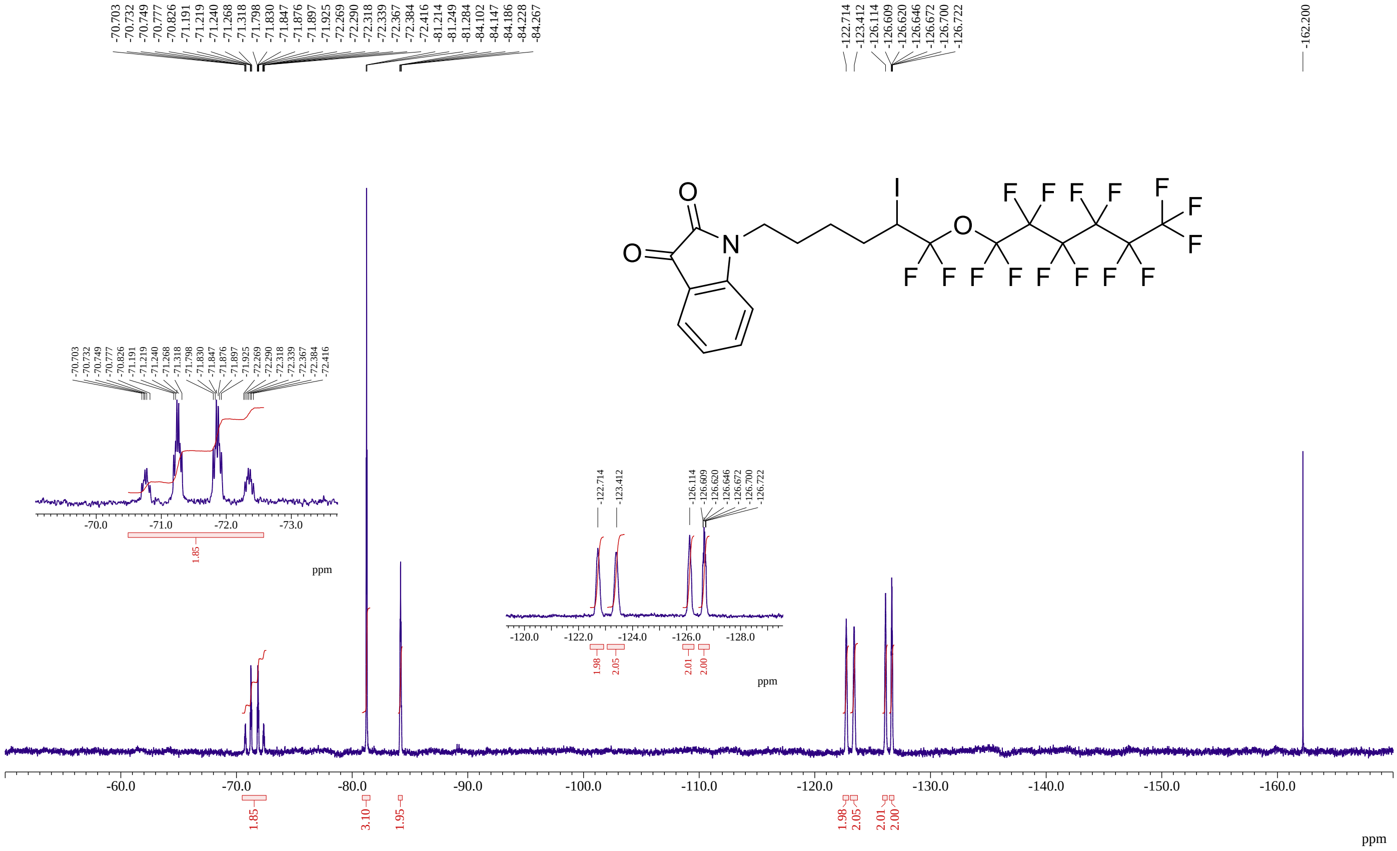

^{13}C NMR (126 MHz, $CDCl_3$): **3ga**

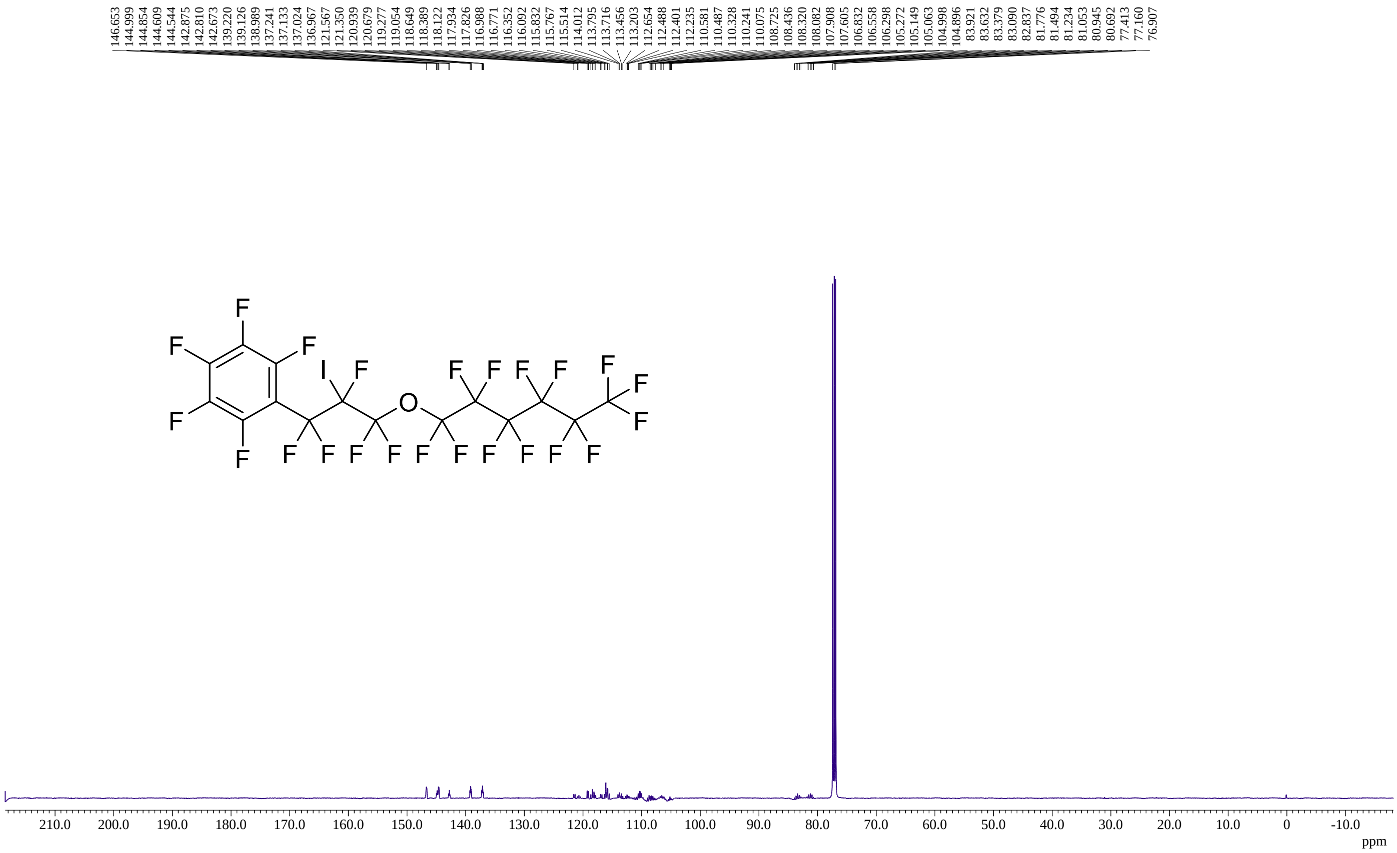

^{19}F NMR (282 MHz, $CDCl_3$): **3ga**

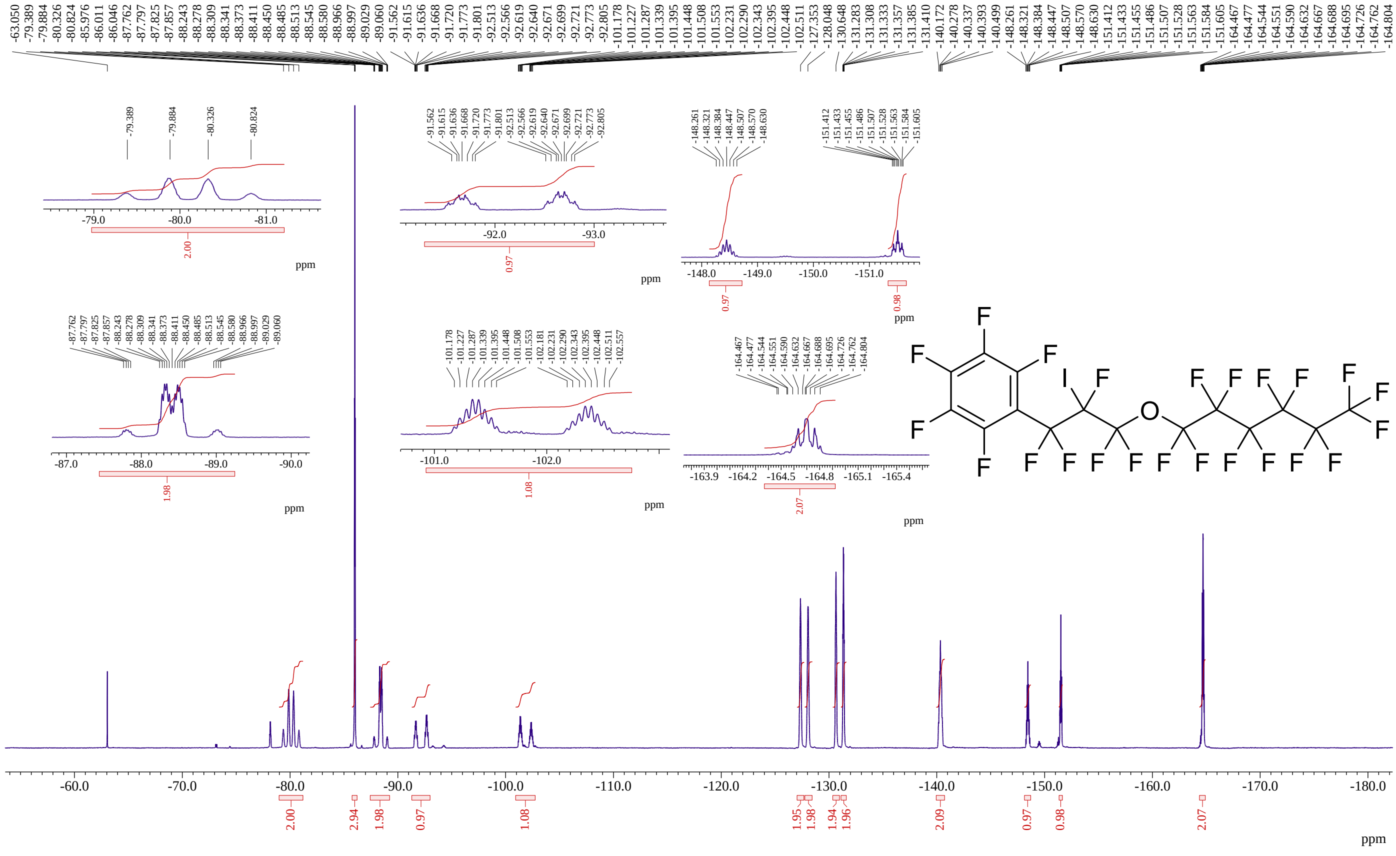

^{1}H NMR (500 MHz, $CDCl_3$): **3na**

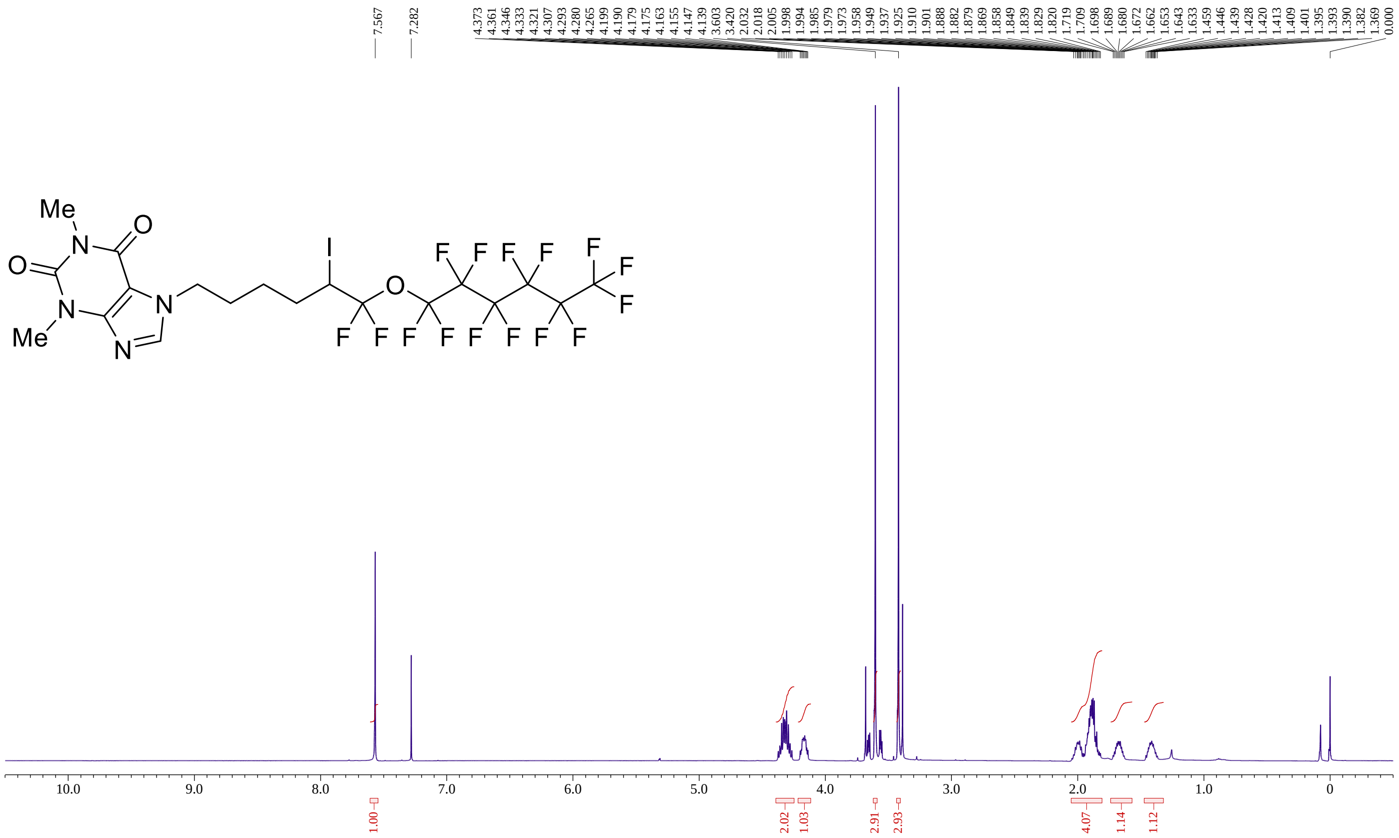

^{13}C NMR (126 MHz, $CDCl_3$): **3na**

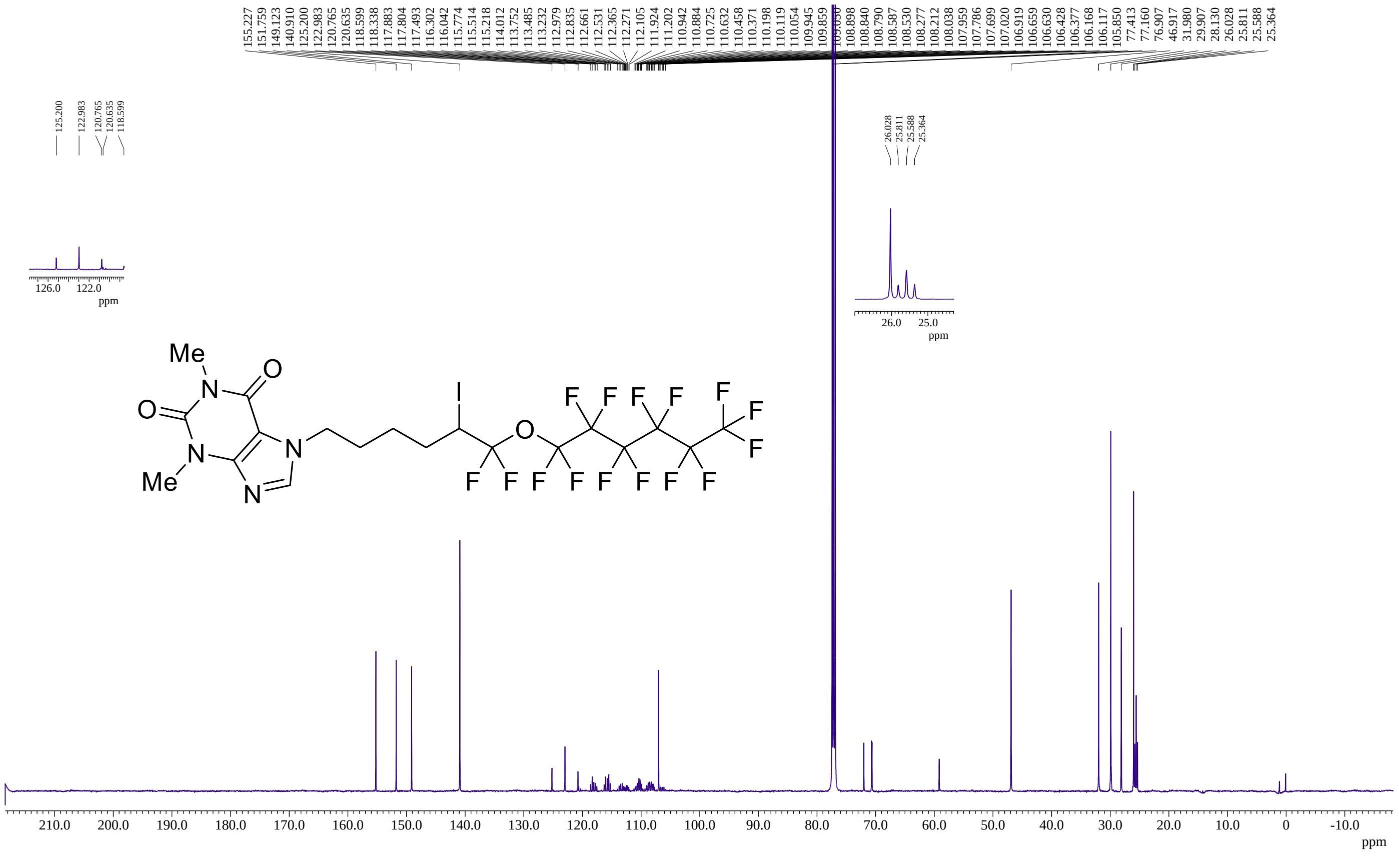

^{19}F NMR (282 MHz, $CDCl_3$): **3na**

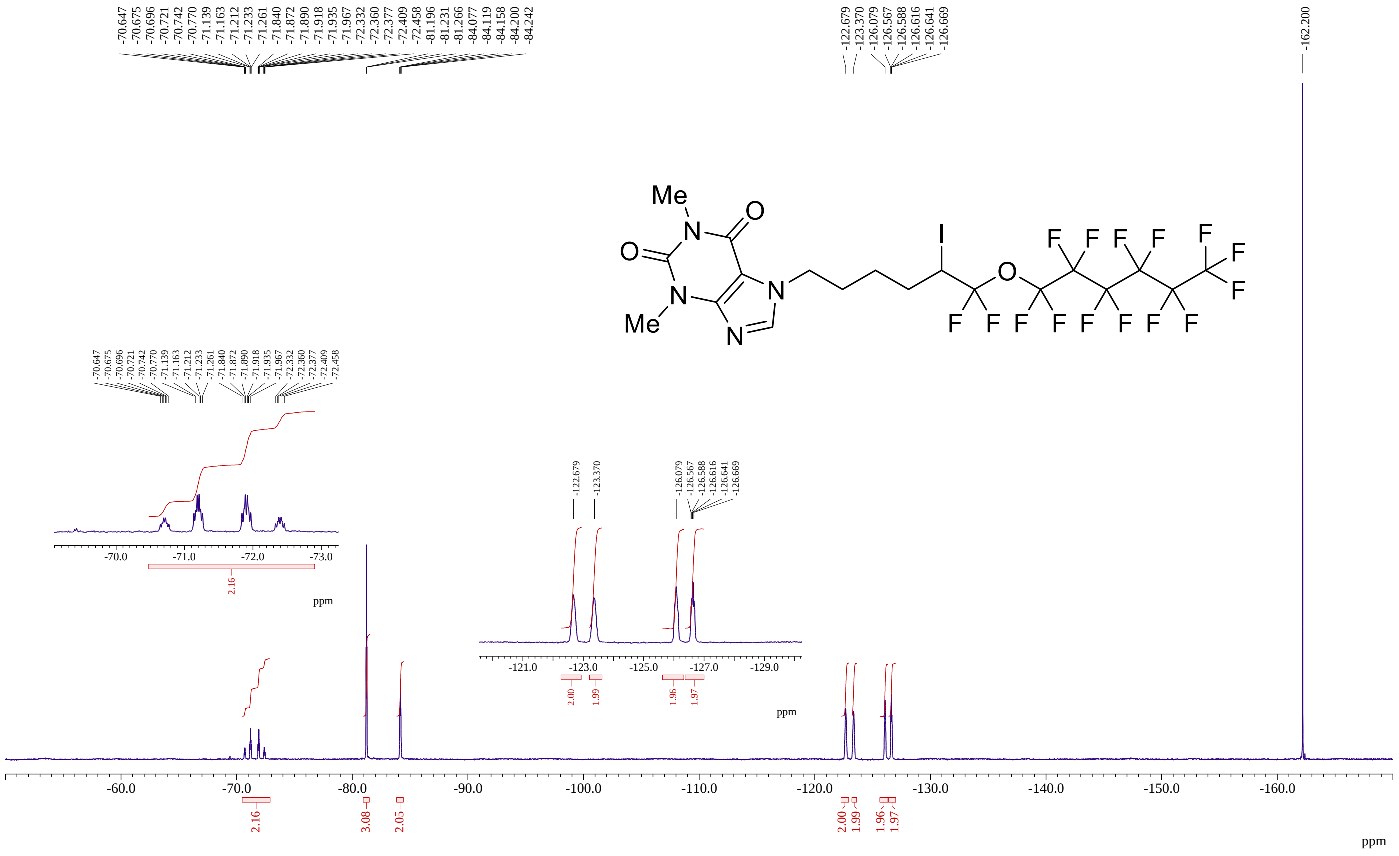

^{1}H NMR (500 MHz, $CDCl_3$): **3oa**

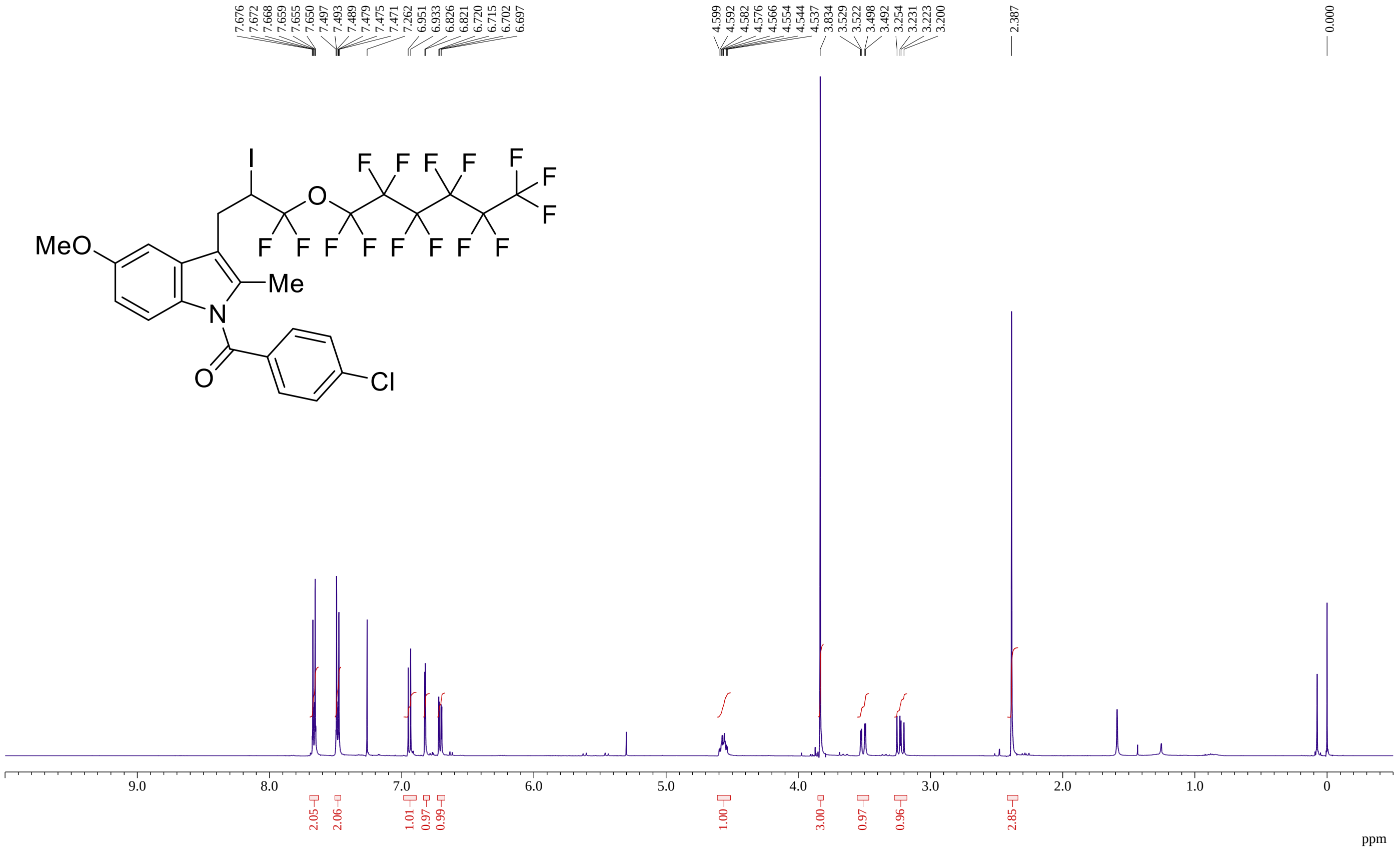

^{13}C NMR (126 MHz, CDCl$_3$): **3oa**

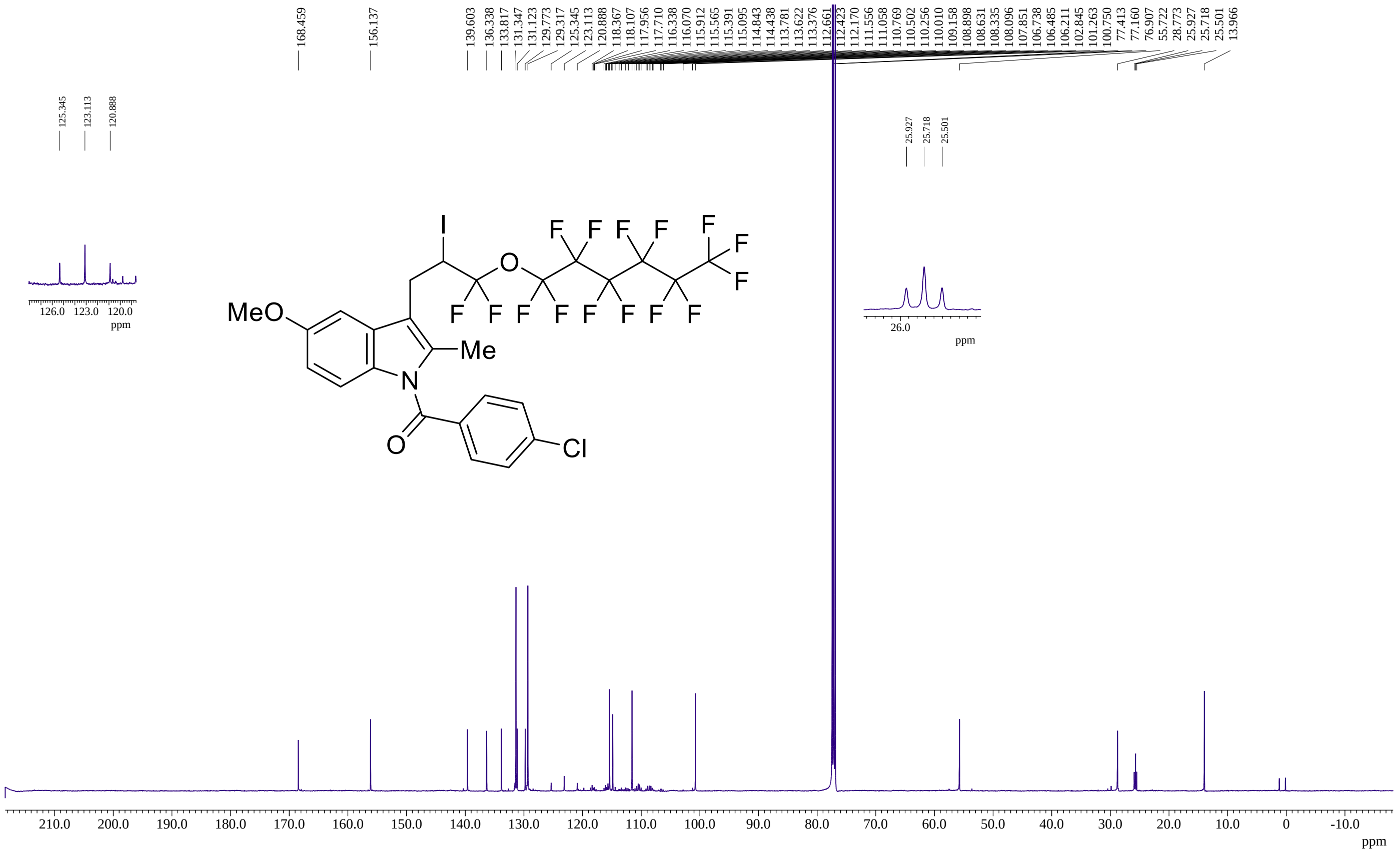

^{19}F NMR (282 MHz, CDCl$_3$): **3oa**

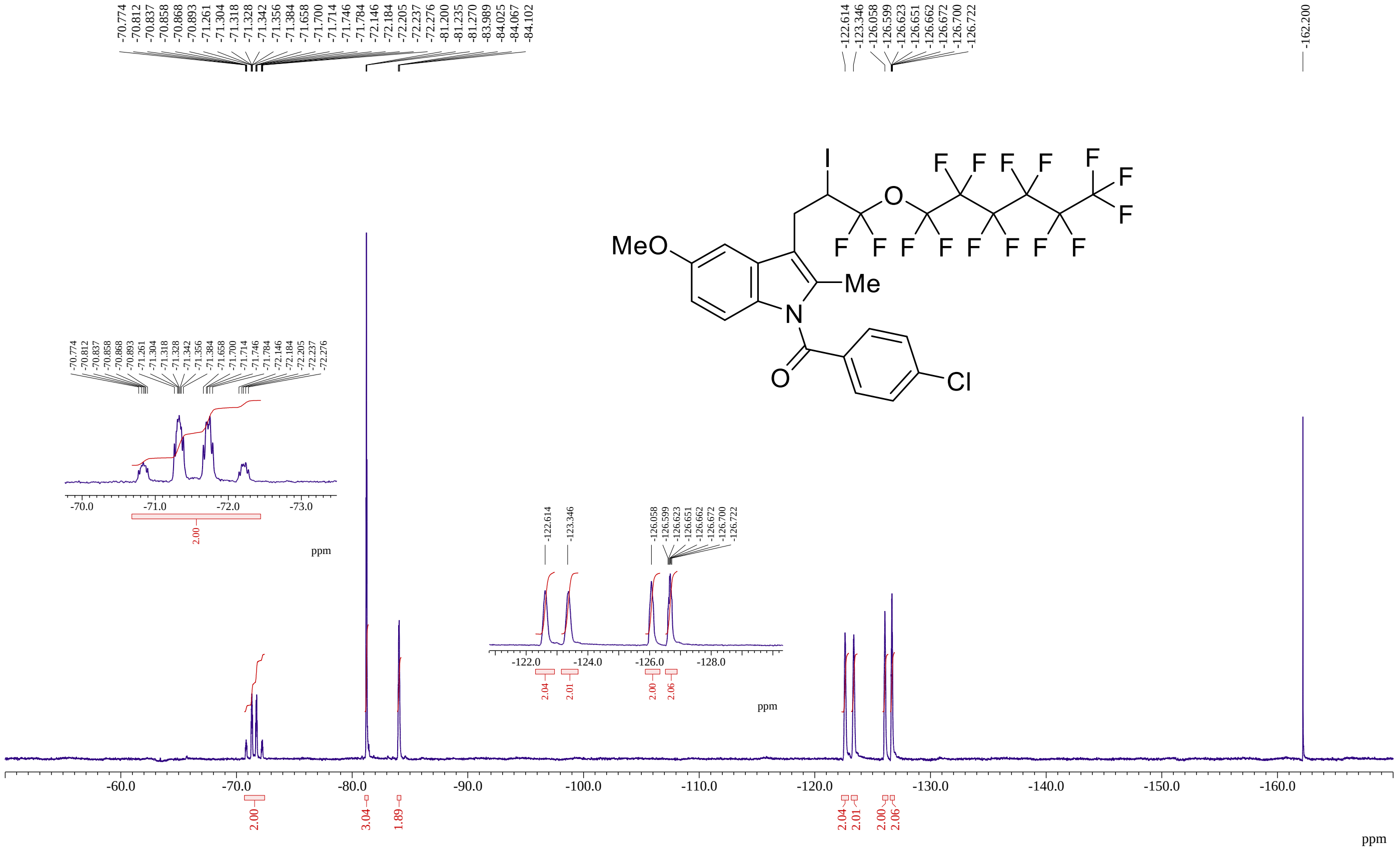

^{1}H NMR (500 MHz, CDCl$_3$): **3pa**

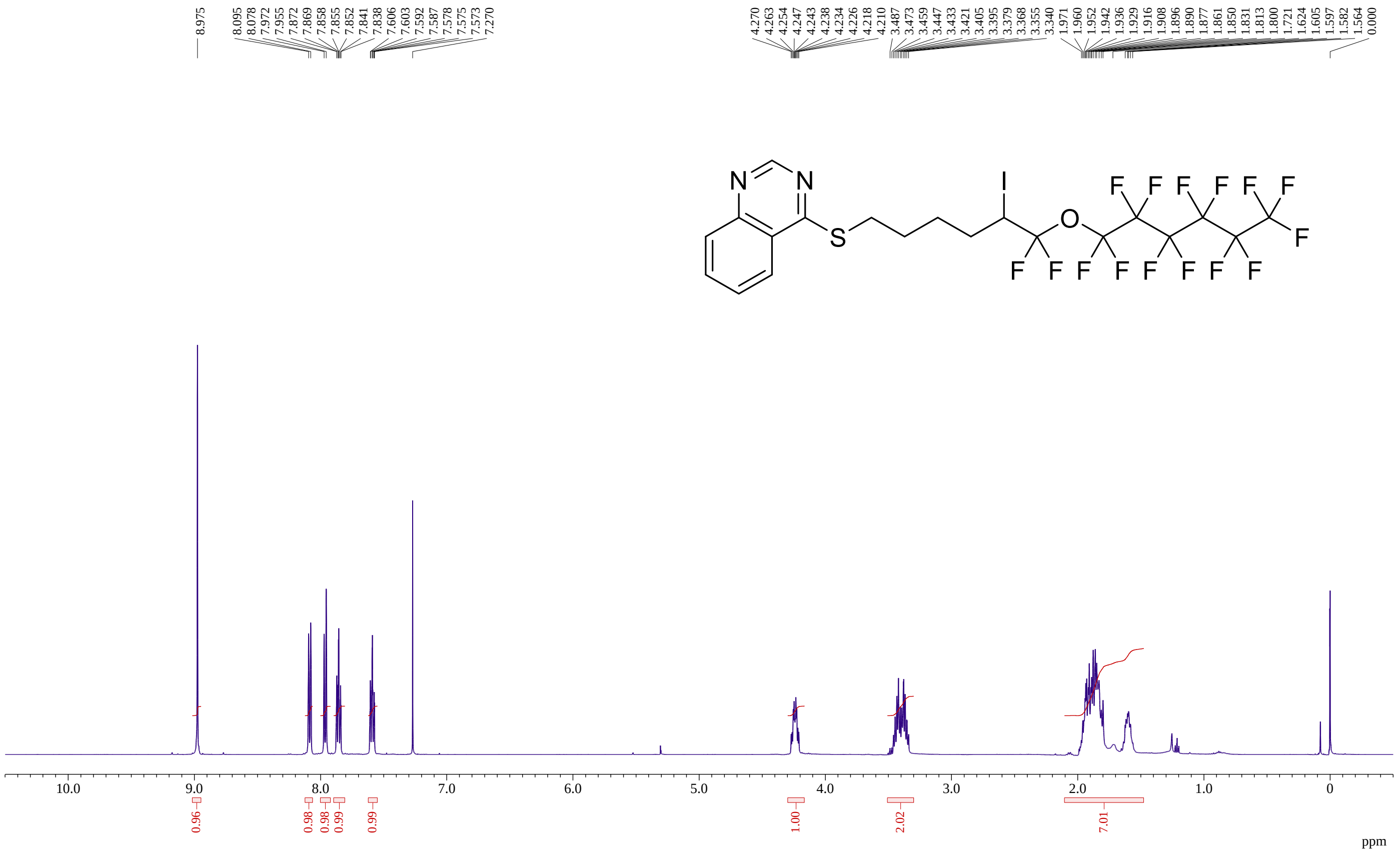

^{13}C NMR (126 MHz, CDCl$_3$): **3pa**

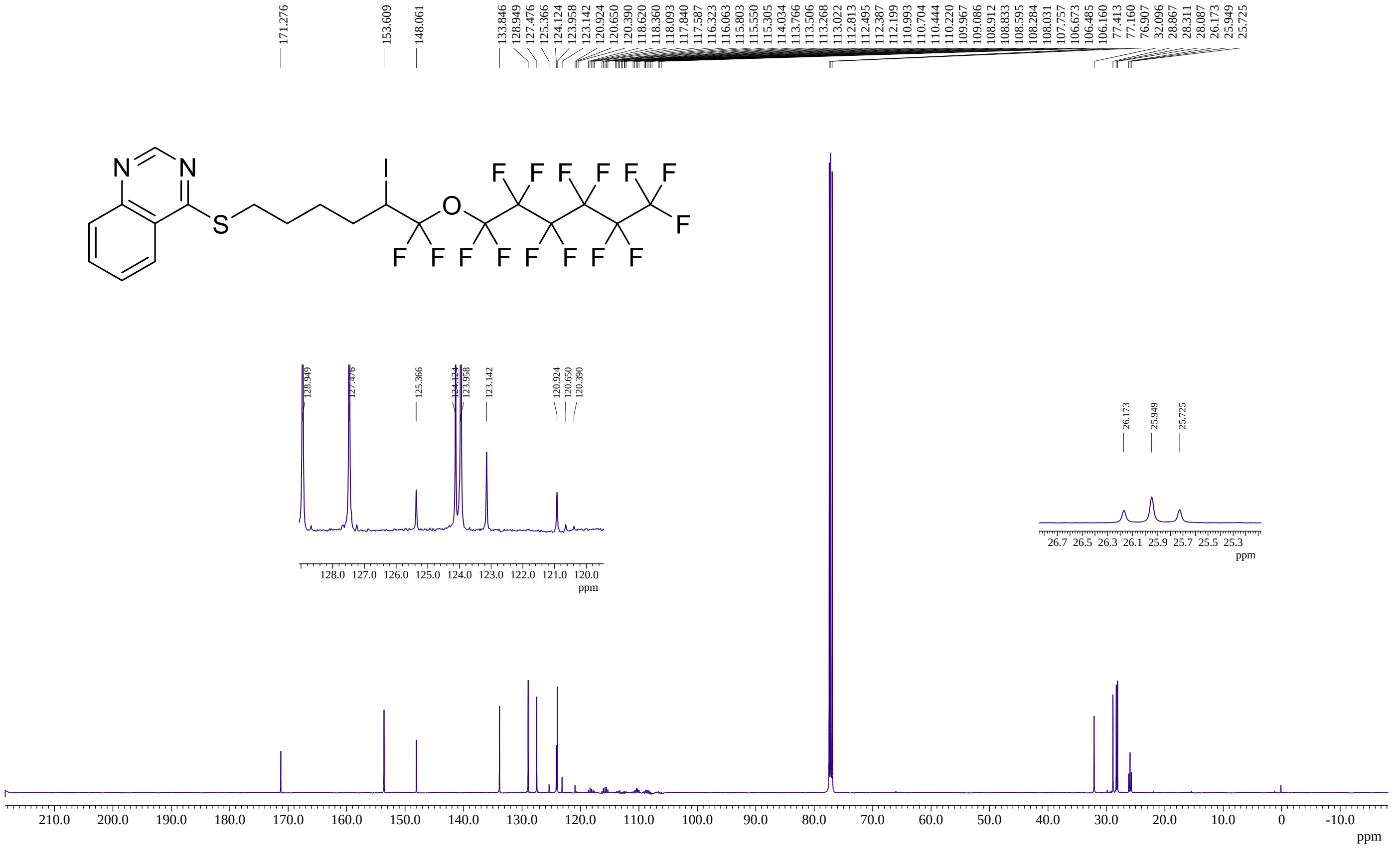

^{19}F NMR (282 MHz, CDCl$_3$): **3pa**

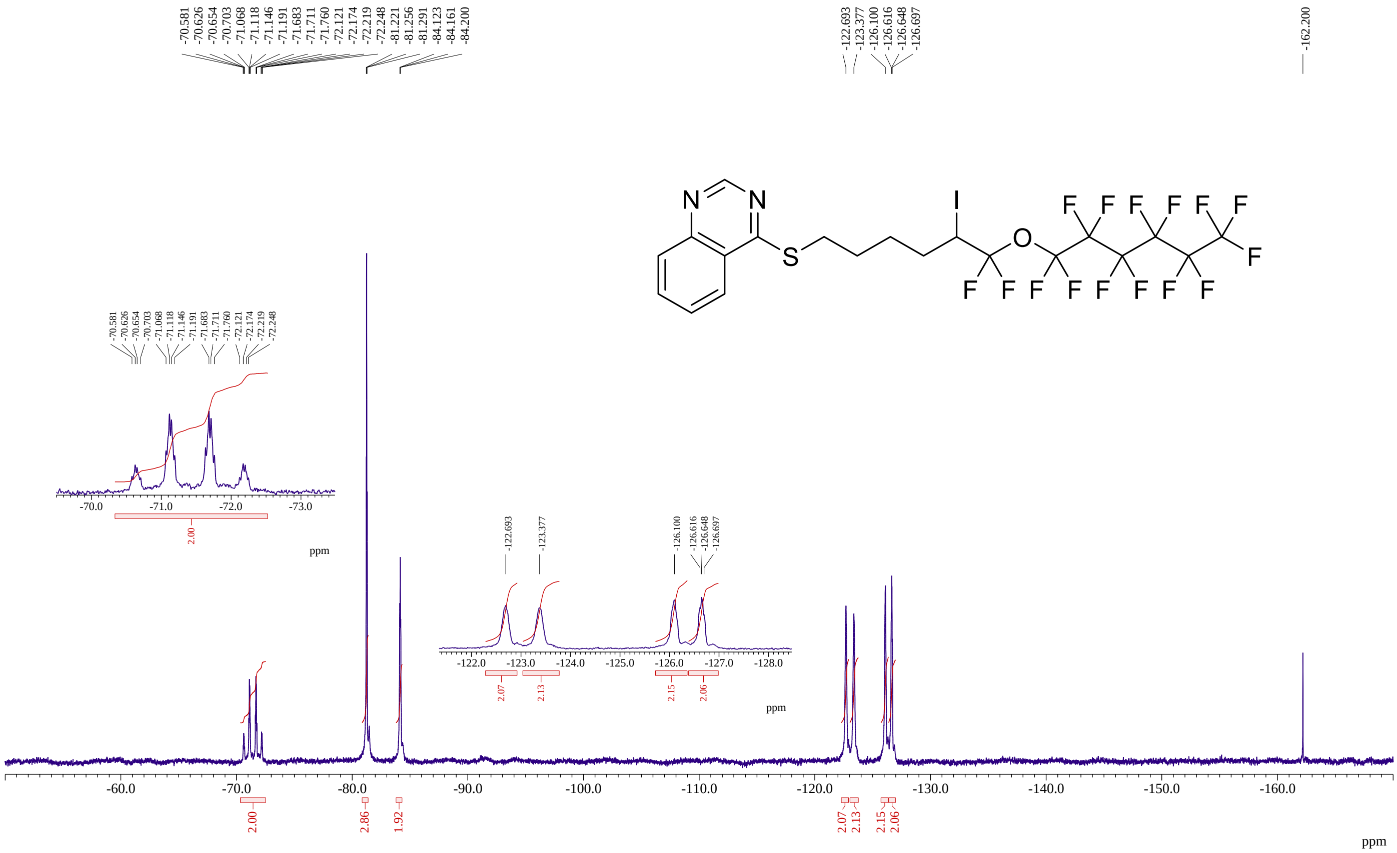

^{1}H NMR (500 MHz, CDCl$_3$): **3qa**

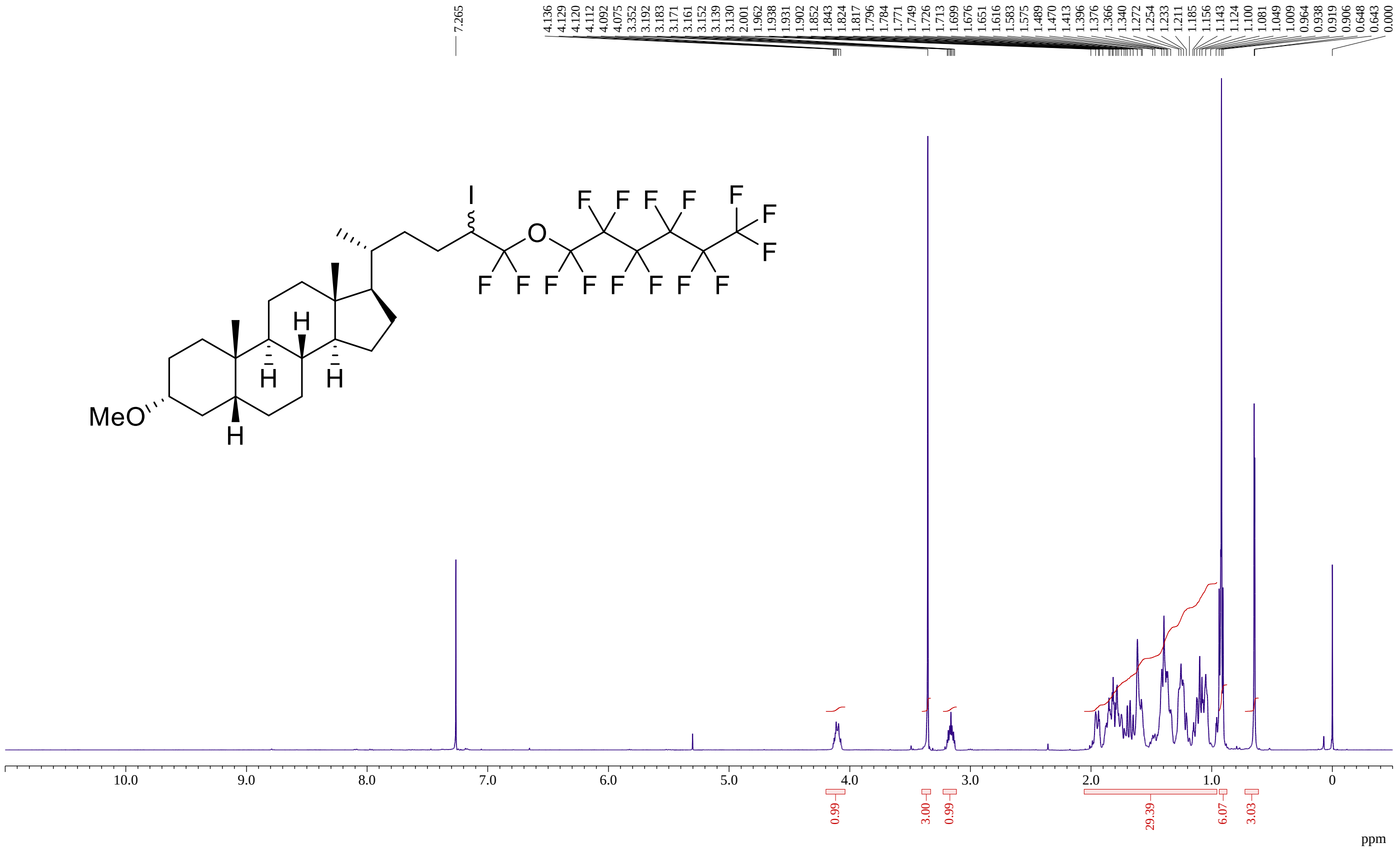

^{13}C NMR (126 MHz, CDCl$_3$): 3qa

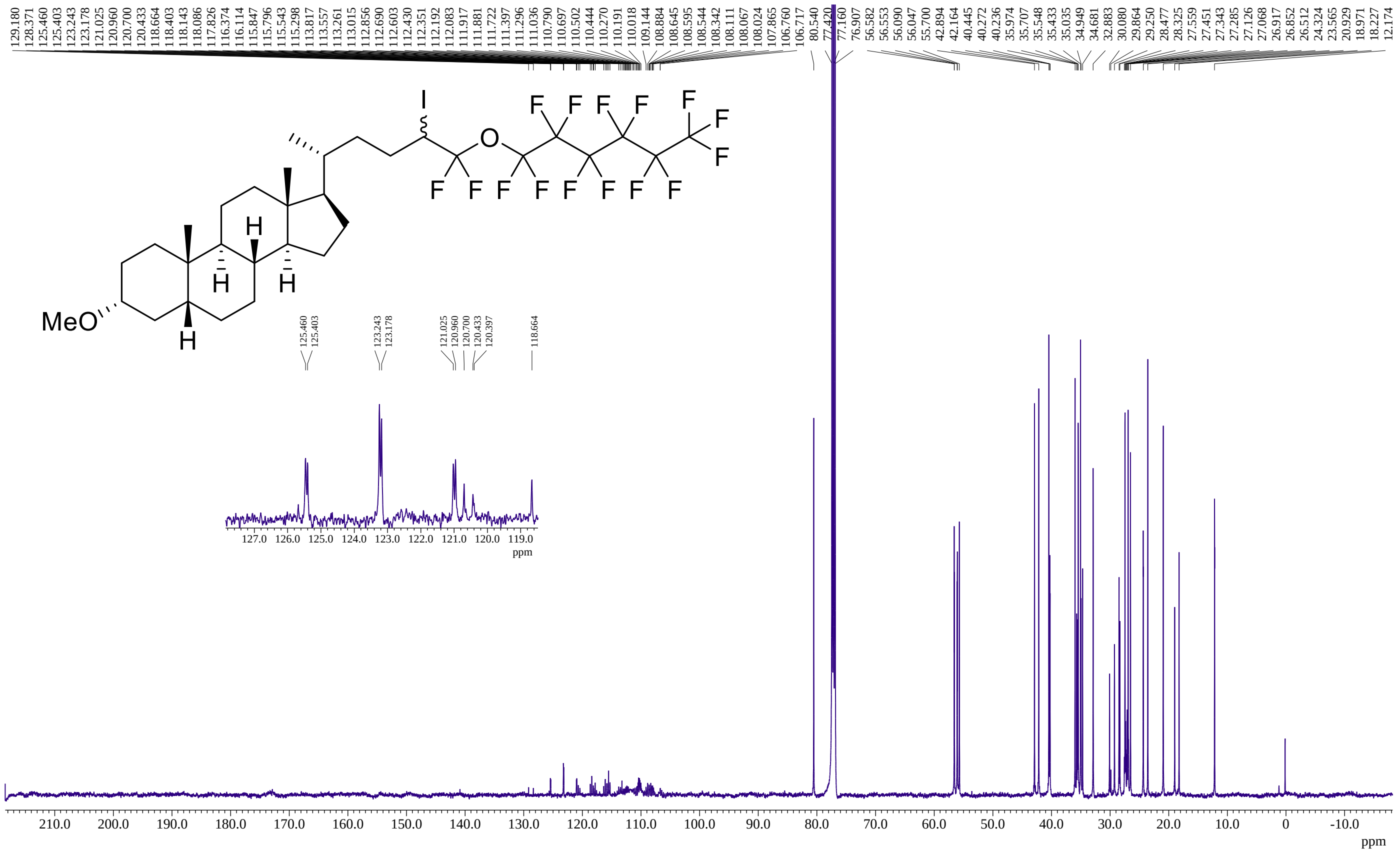

^{19}F NMR (282 MHz, CDCl$_3$): 3qa

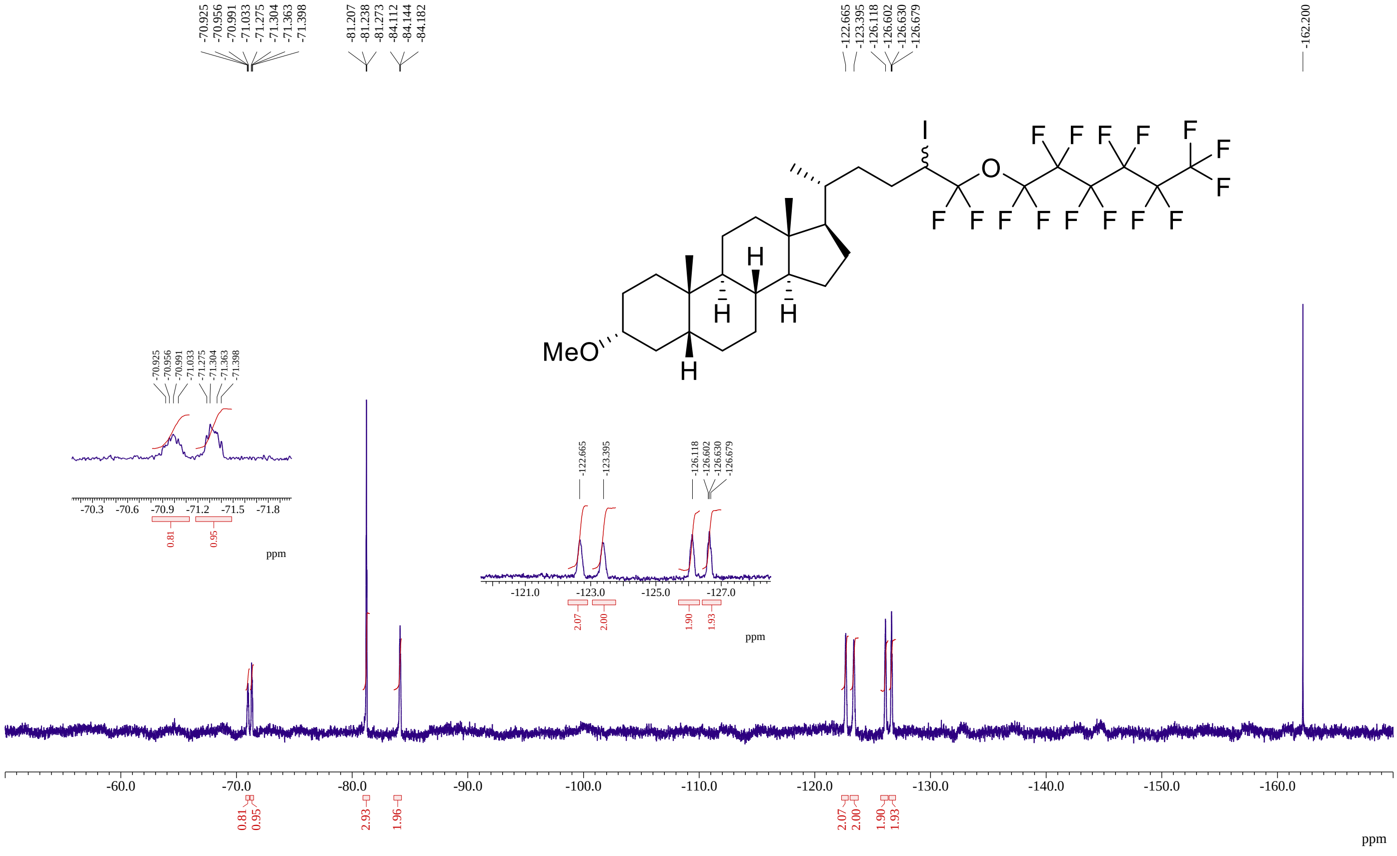

^{1}H NMR (500 MHz, $CDCl_3$): **3ra**

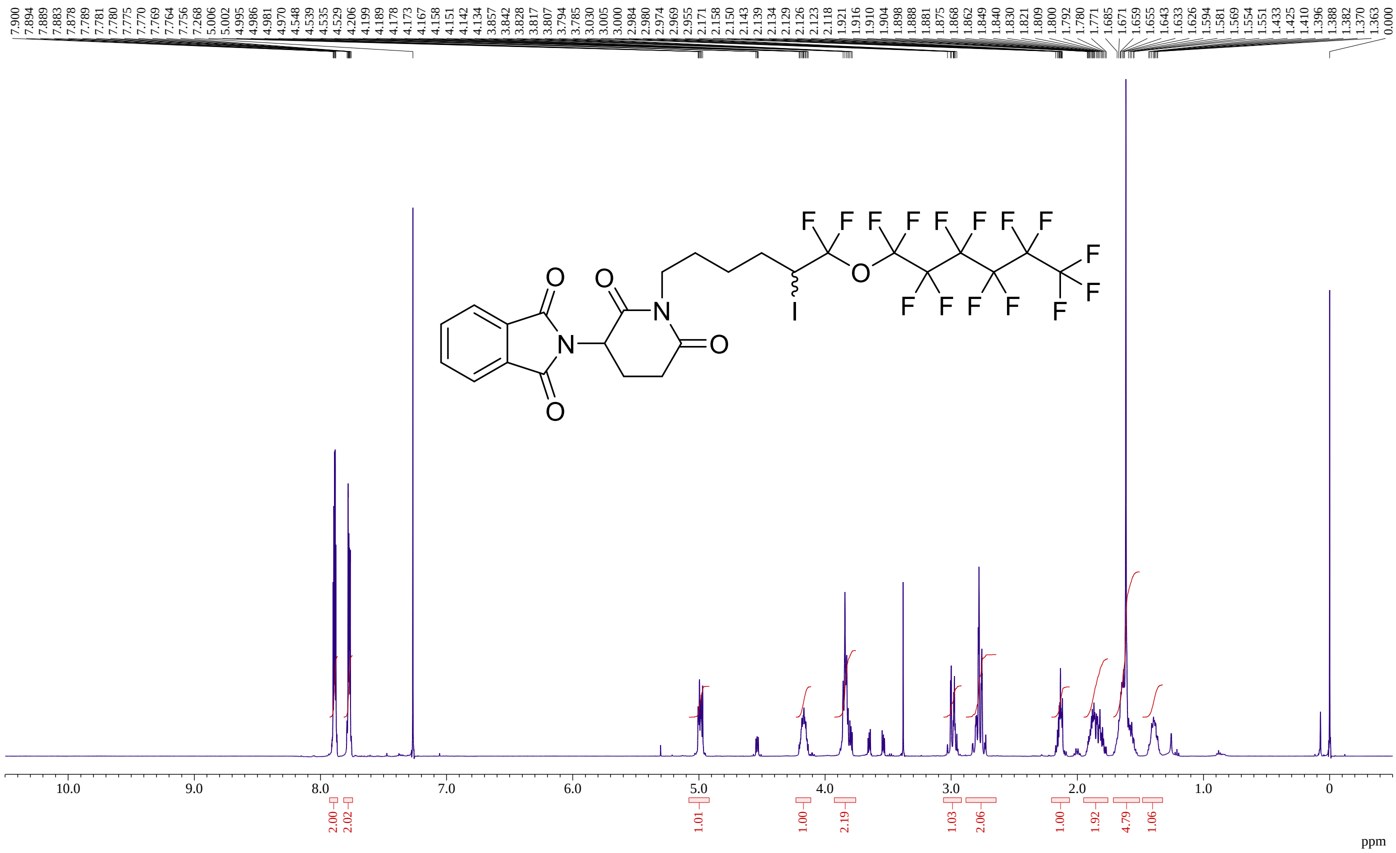

^{13}C NMR (126 MHz, $CDCl_3$): **3ra**

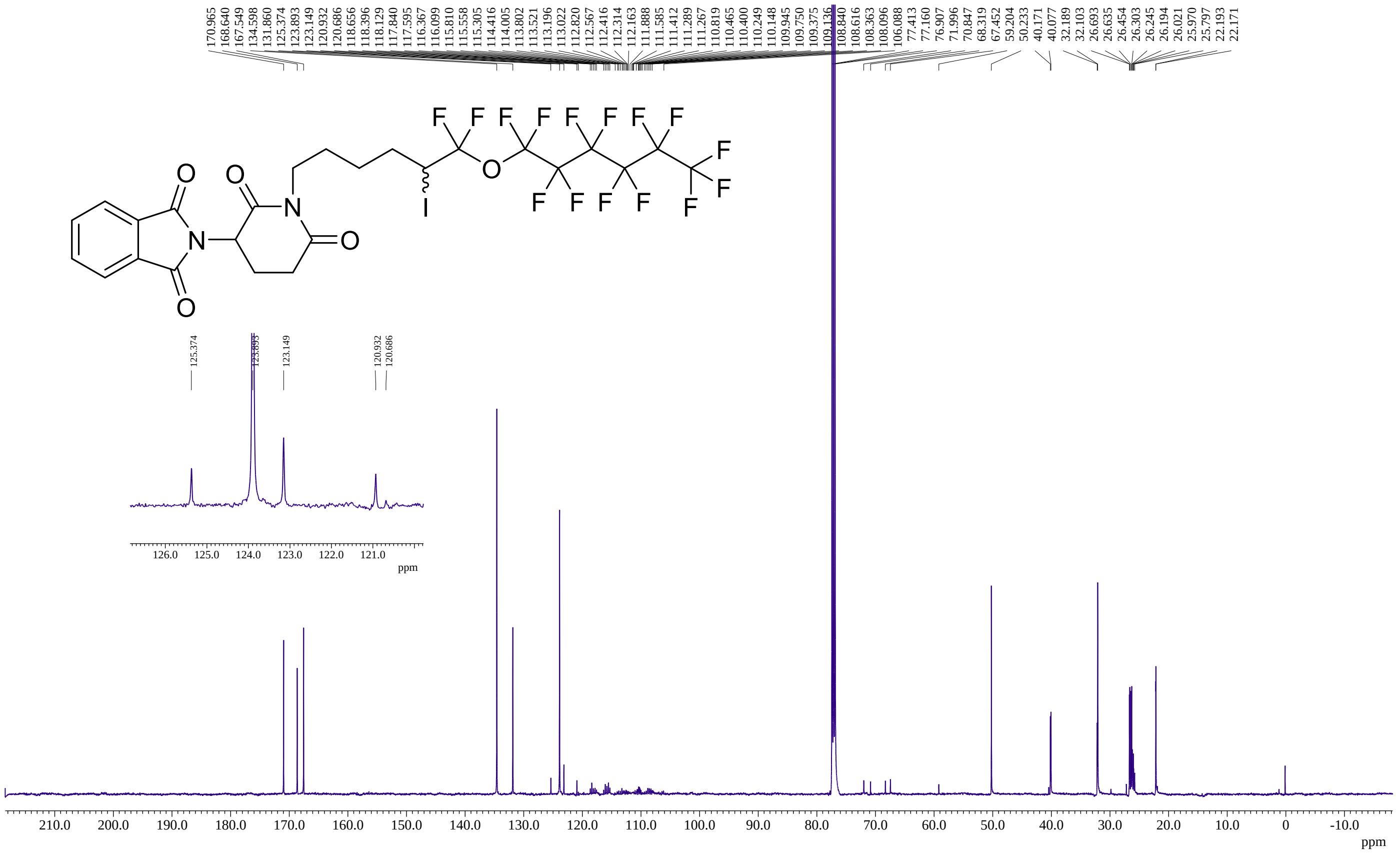

^{19}F NMR (282 MHz, $CDCl_3$): **3ra**

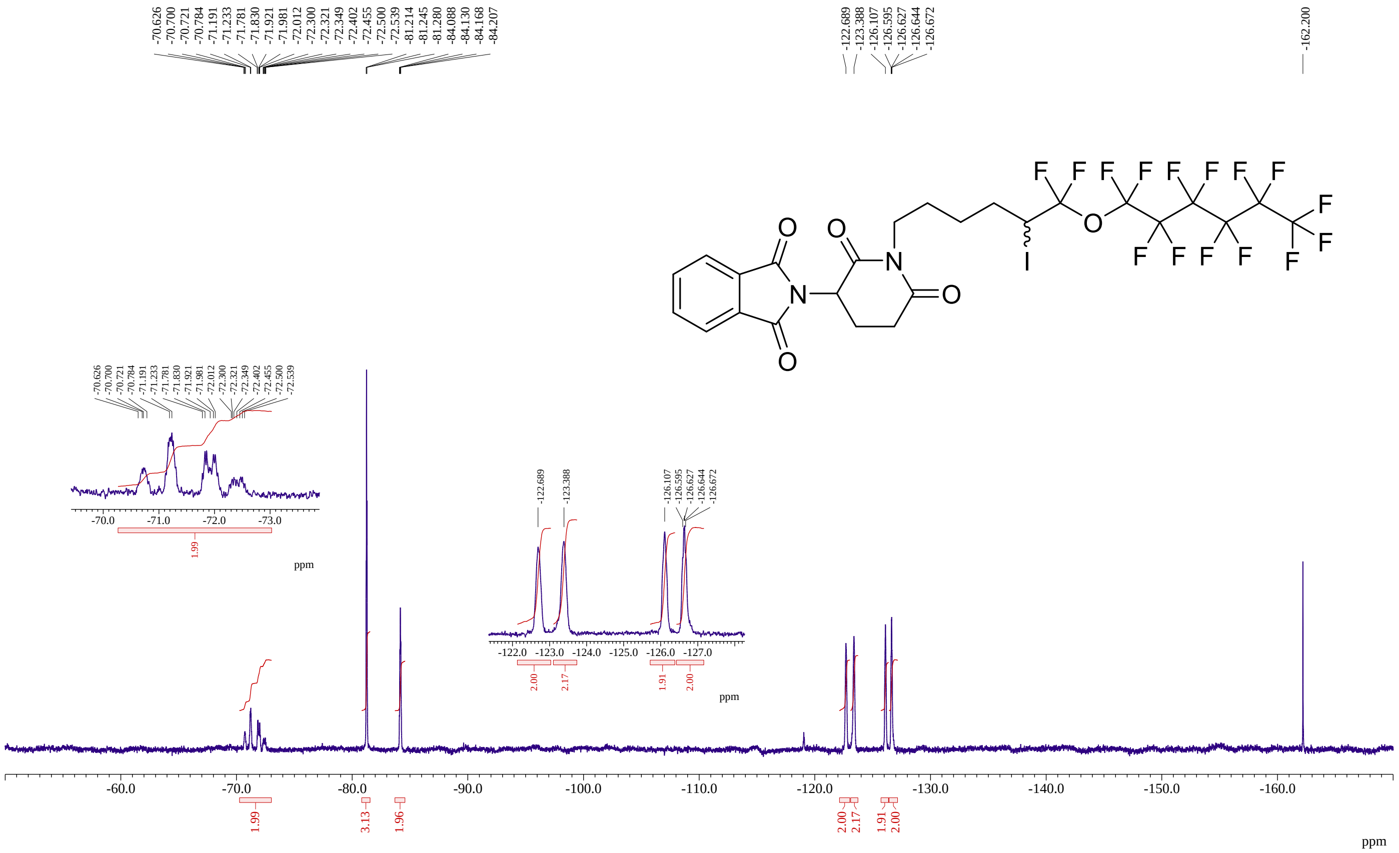

^{1}H NMR (500 MHz, $CDCl_3$): **4aa**

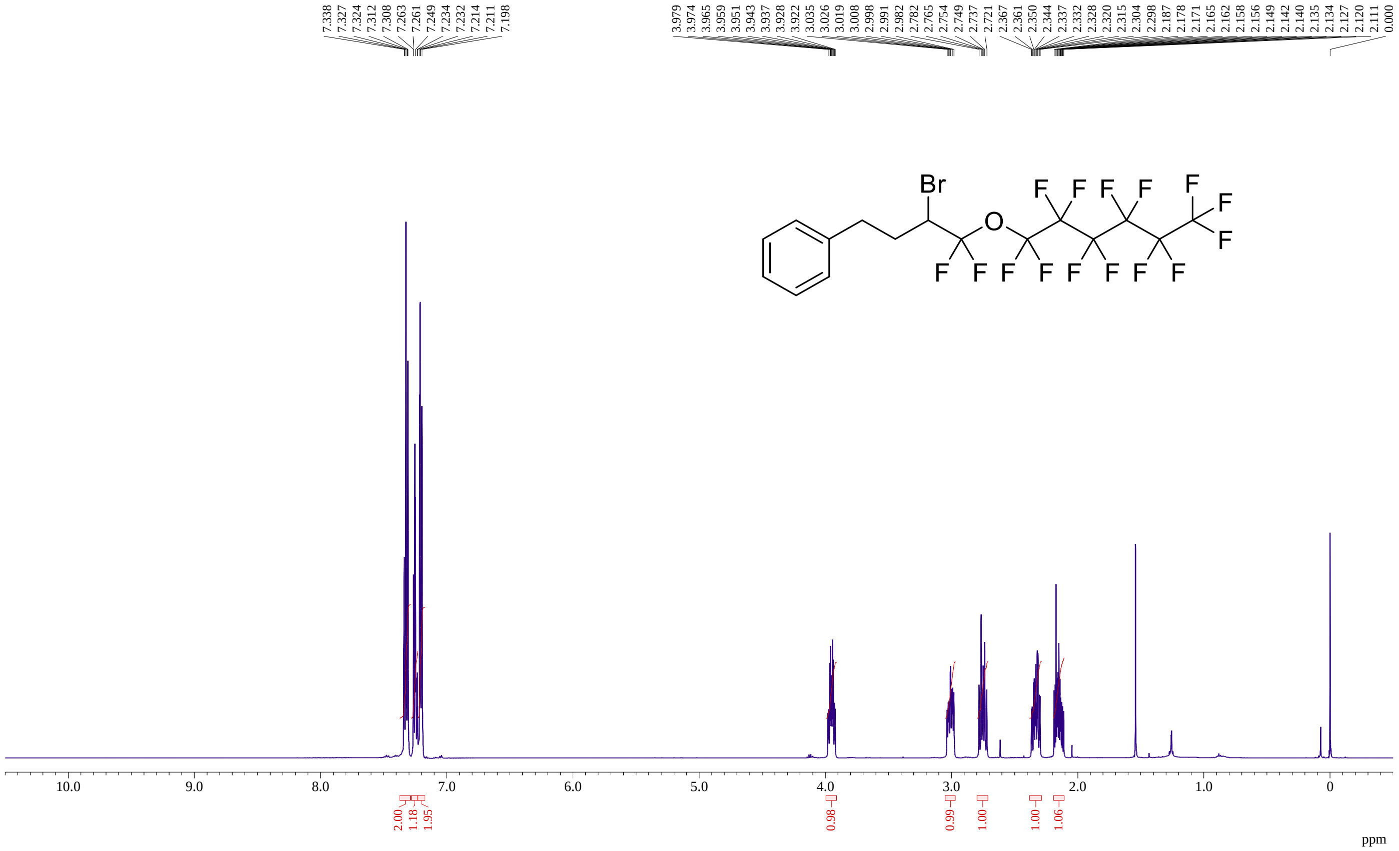

^{13}C NMR (126 MHz, $CDCl_3$): **4aa**

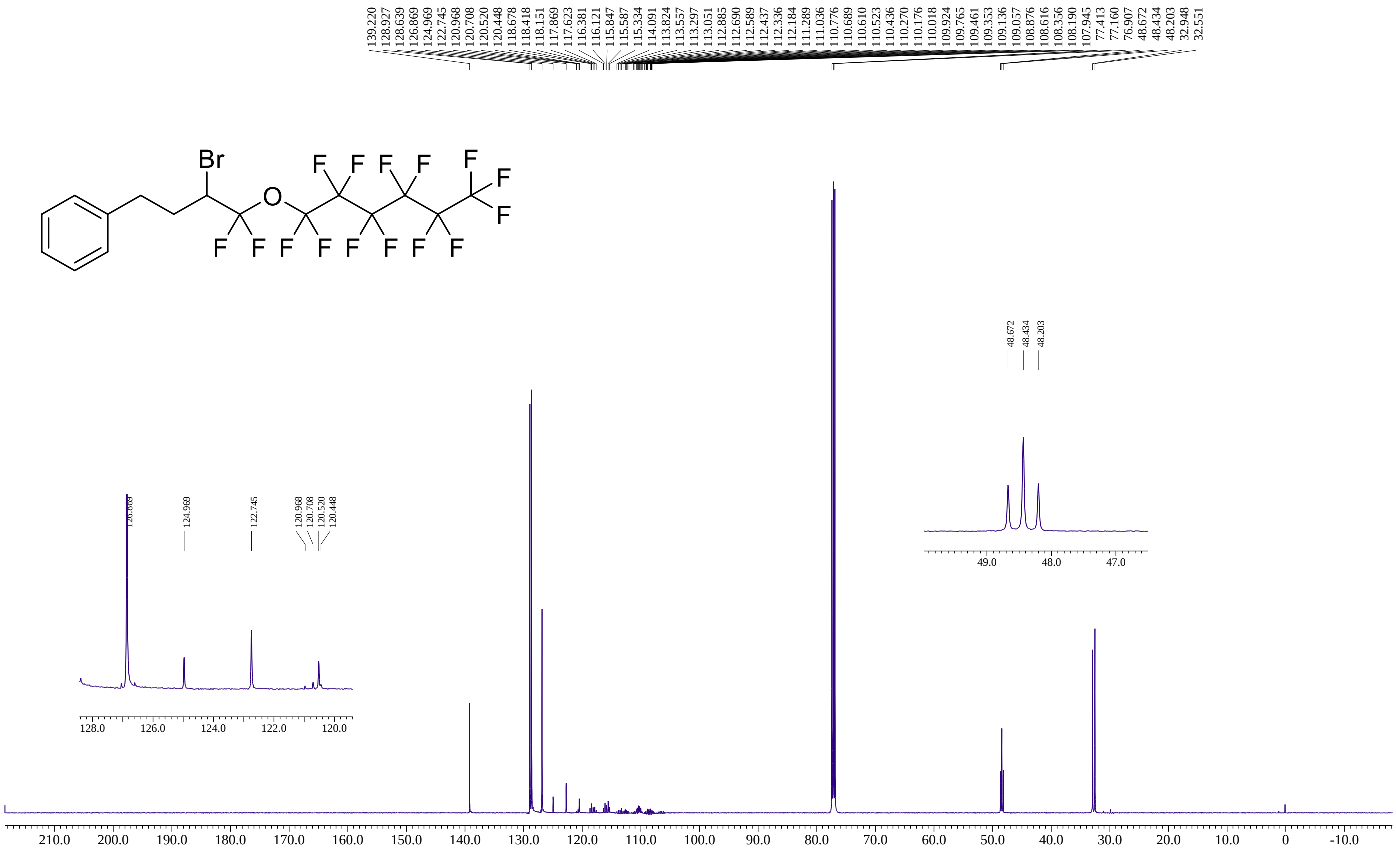

^{19}F NMR (282 MHz, $CDCl_3$): **4aa**

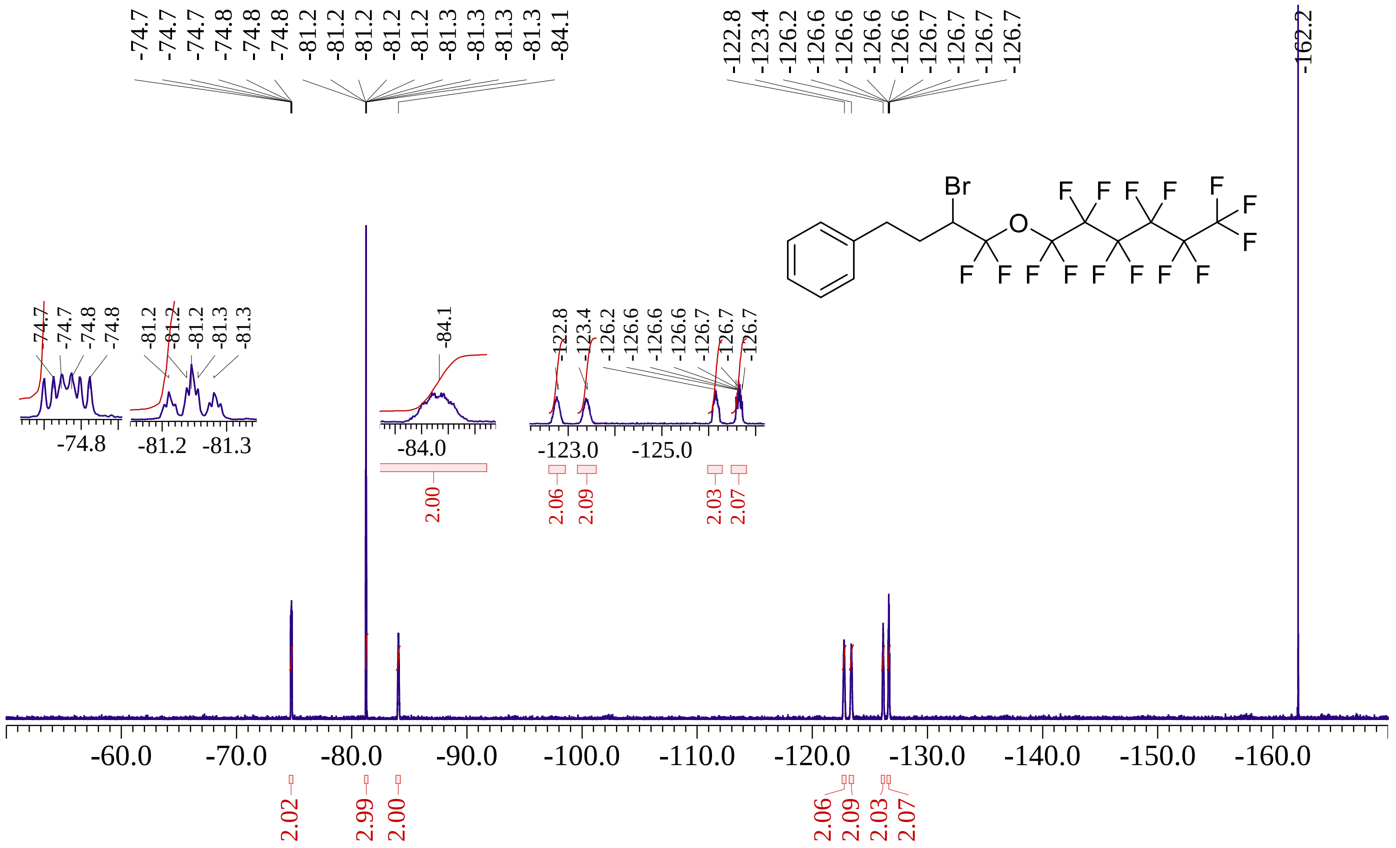

^{1}H NMR (500 MHz, $CDCl_3$): **4ca**

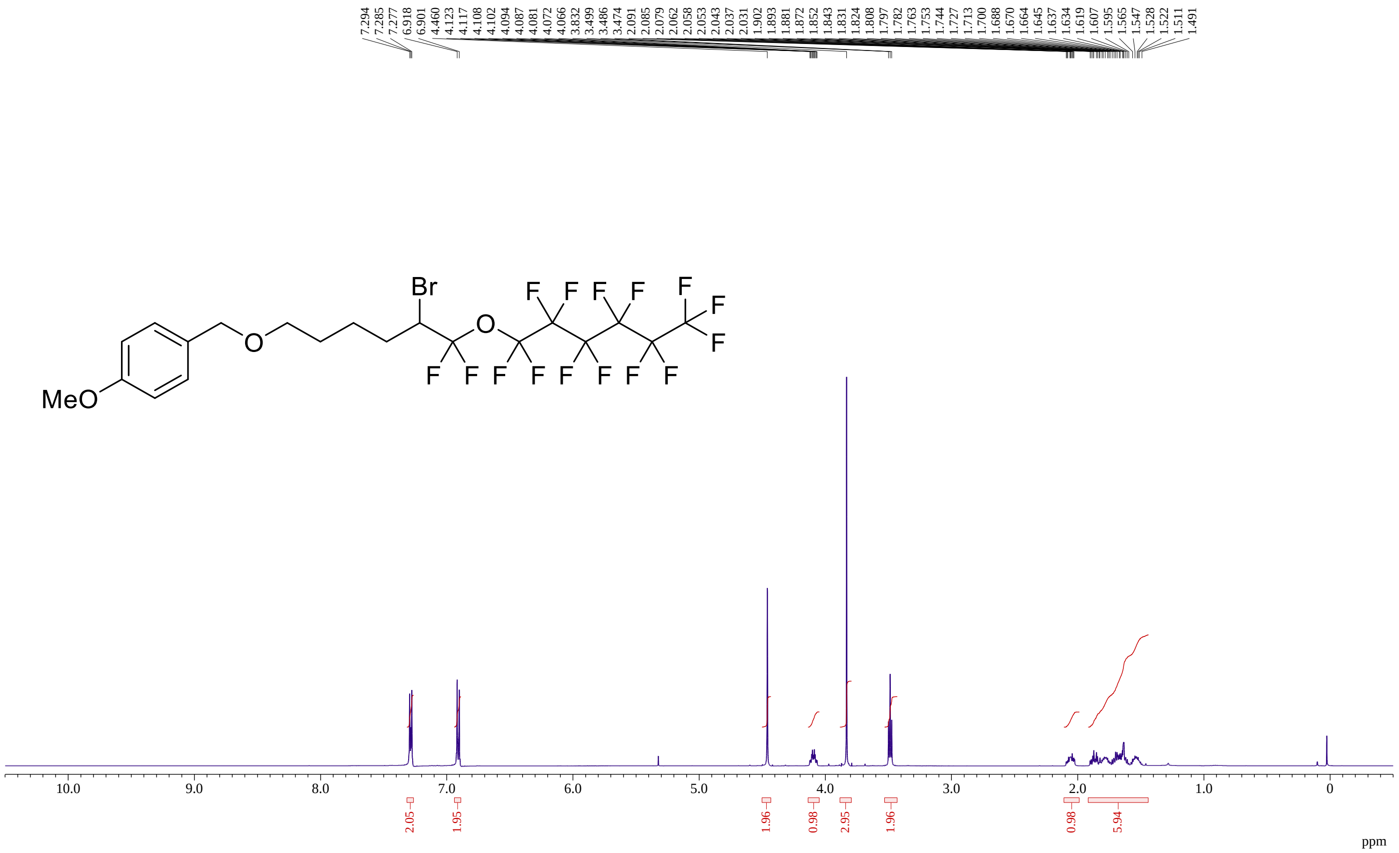

^{13}C NMR (126 MHz, $CDCl_3$): **4ca**

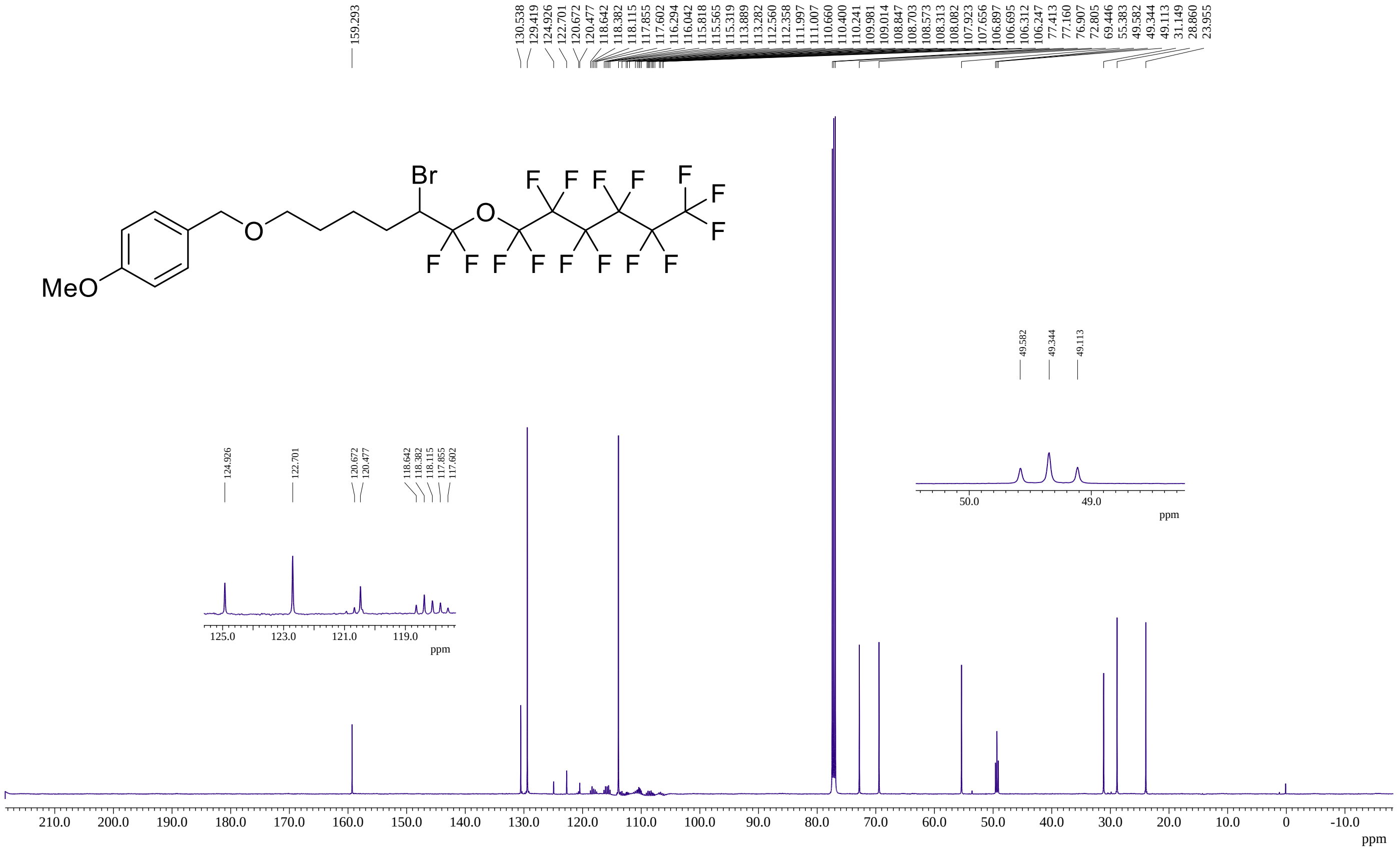

^{19}F NMR (282 MHz, $CDCl_3$): **4ca**

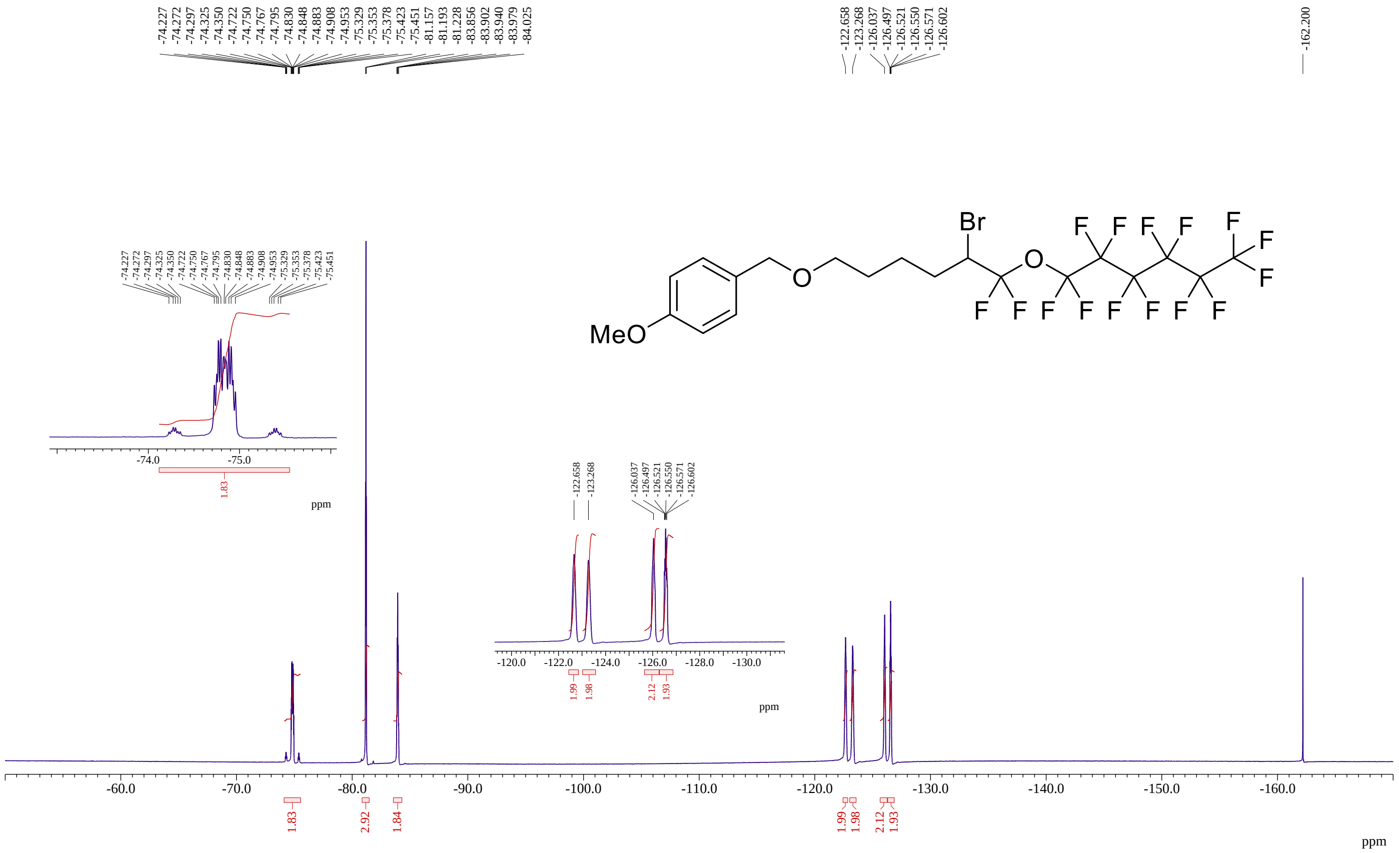

^{1}H NMR (500 MHz, $CDCl_3$): **4ea**

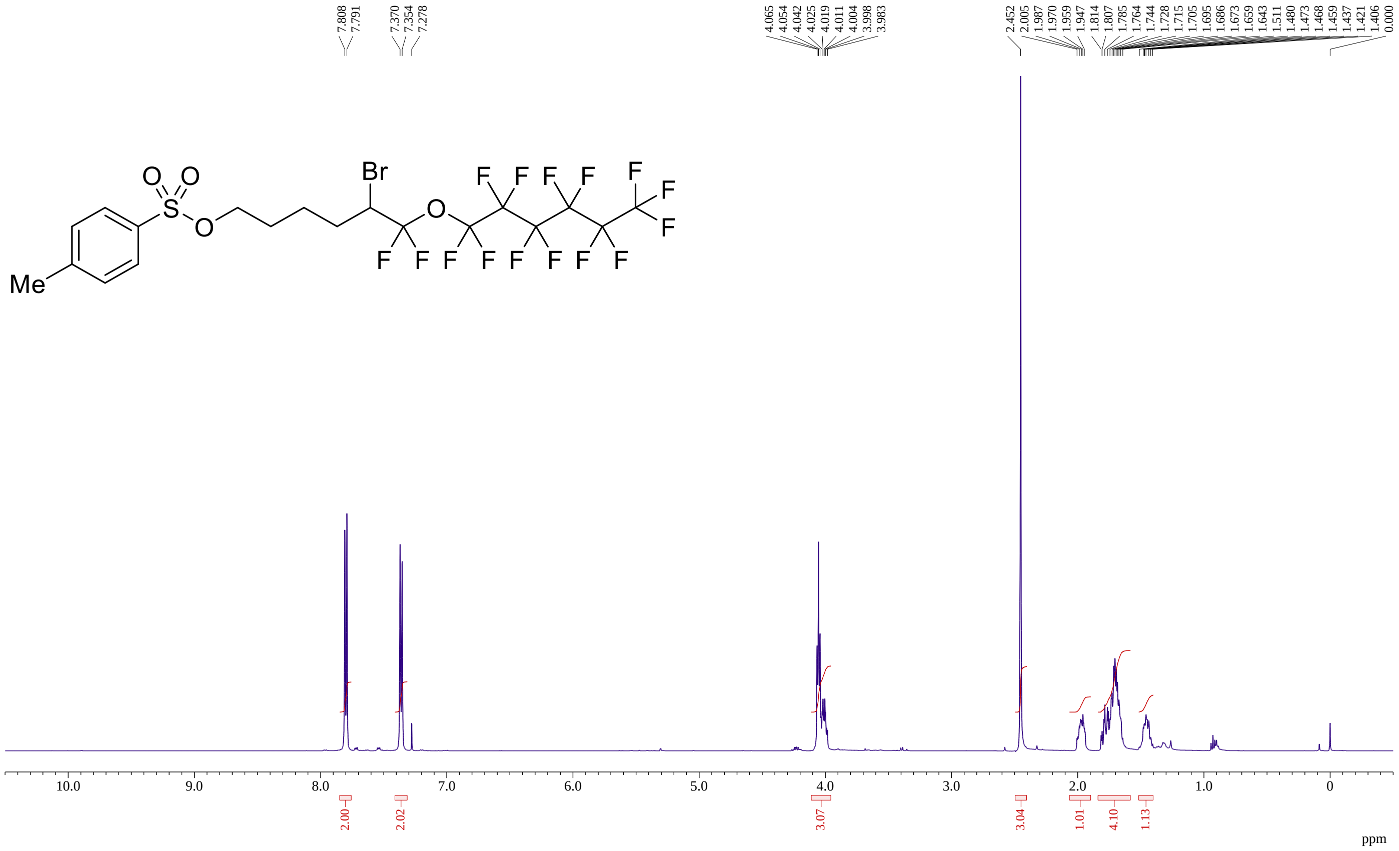

^{13}C NMR (126 MHz, $CDCl_3$): **4ea**

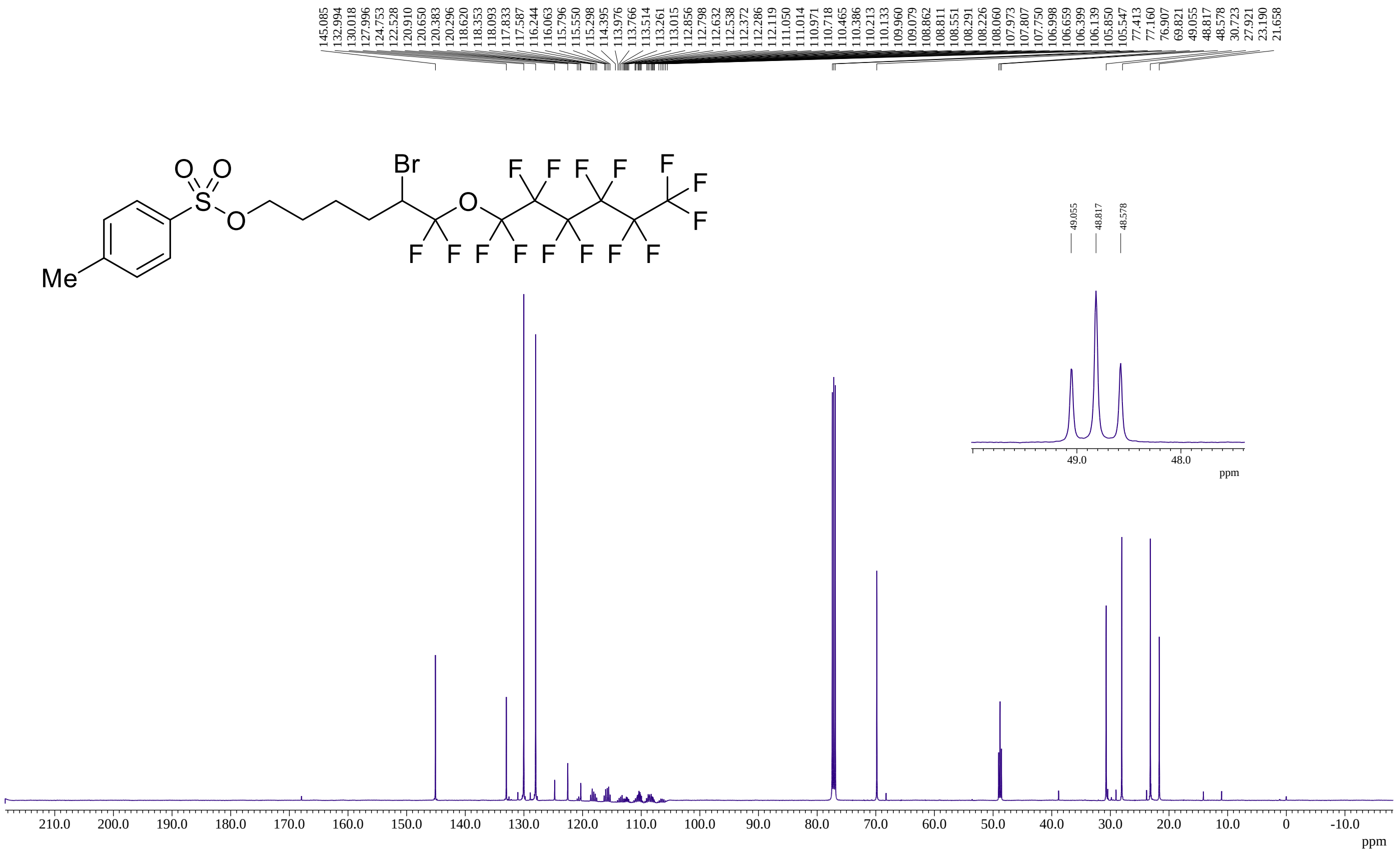

^{19}F NMR (282 MHz, $CDCl_3$): **4ea**

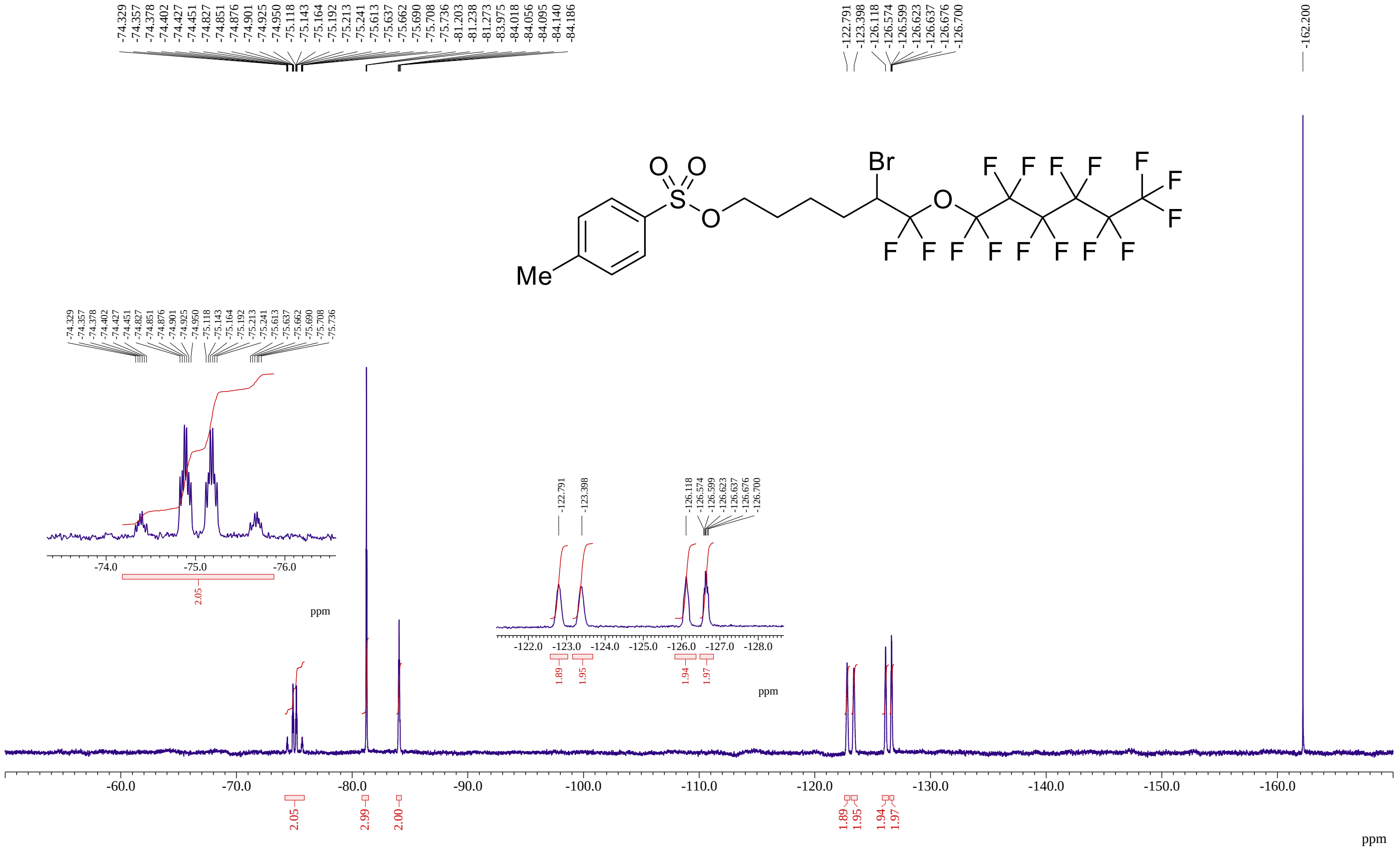

^{1}H NMR (500 MHz, CDCl$_3$): **4ha**

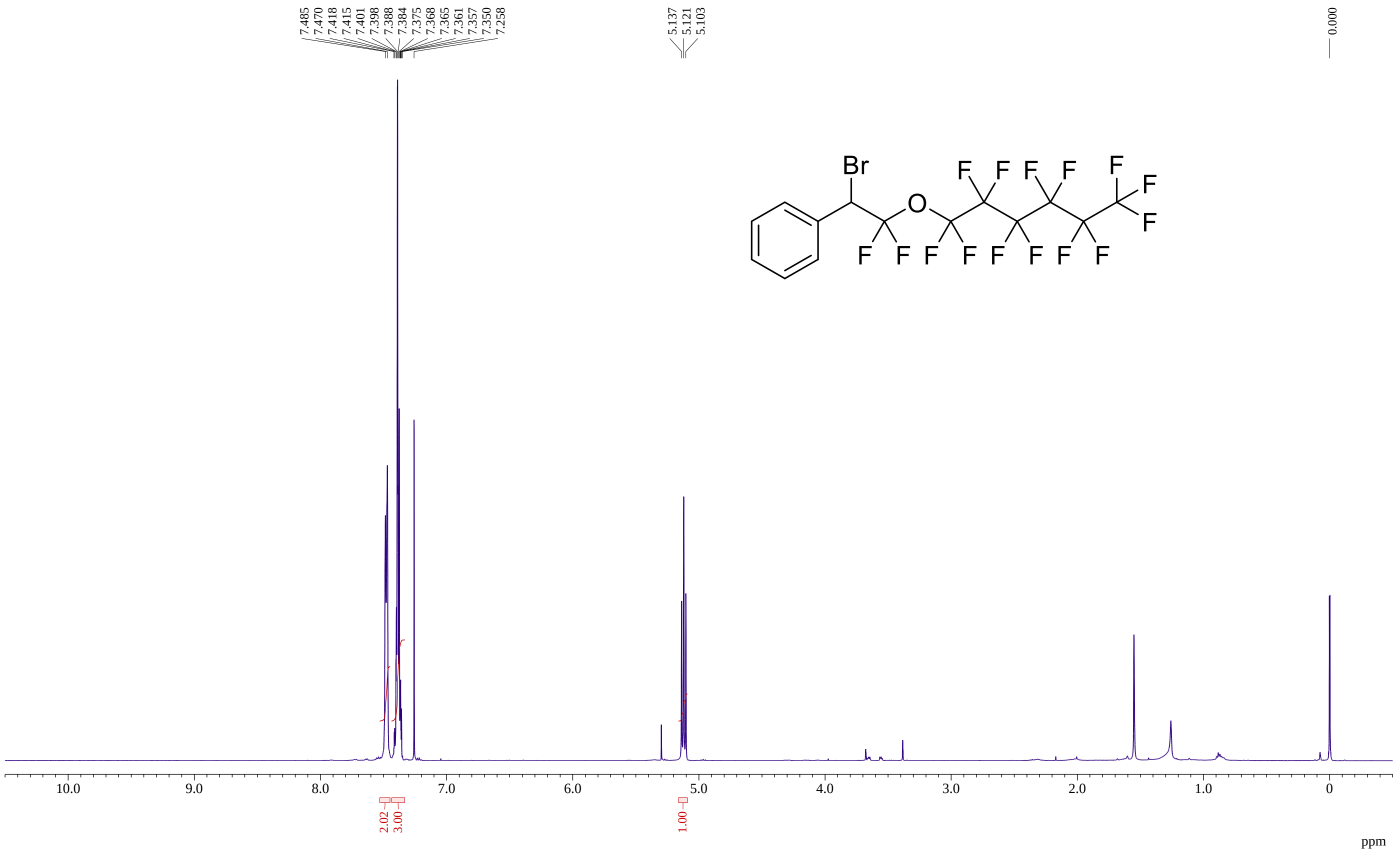

^{13}C NMR (126 MHz, CDCl$_3$): **4ha**

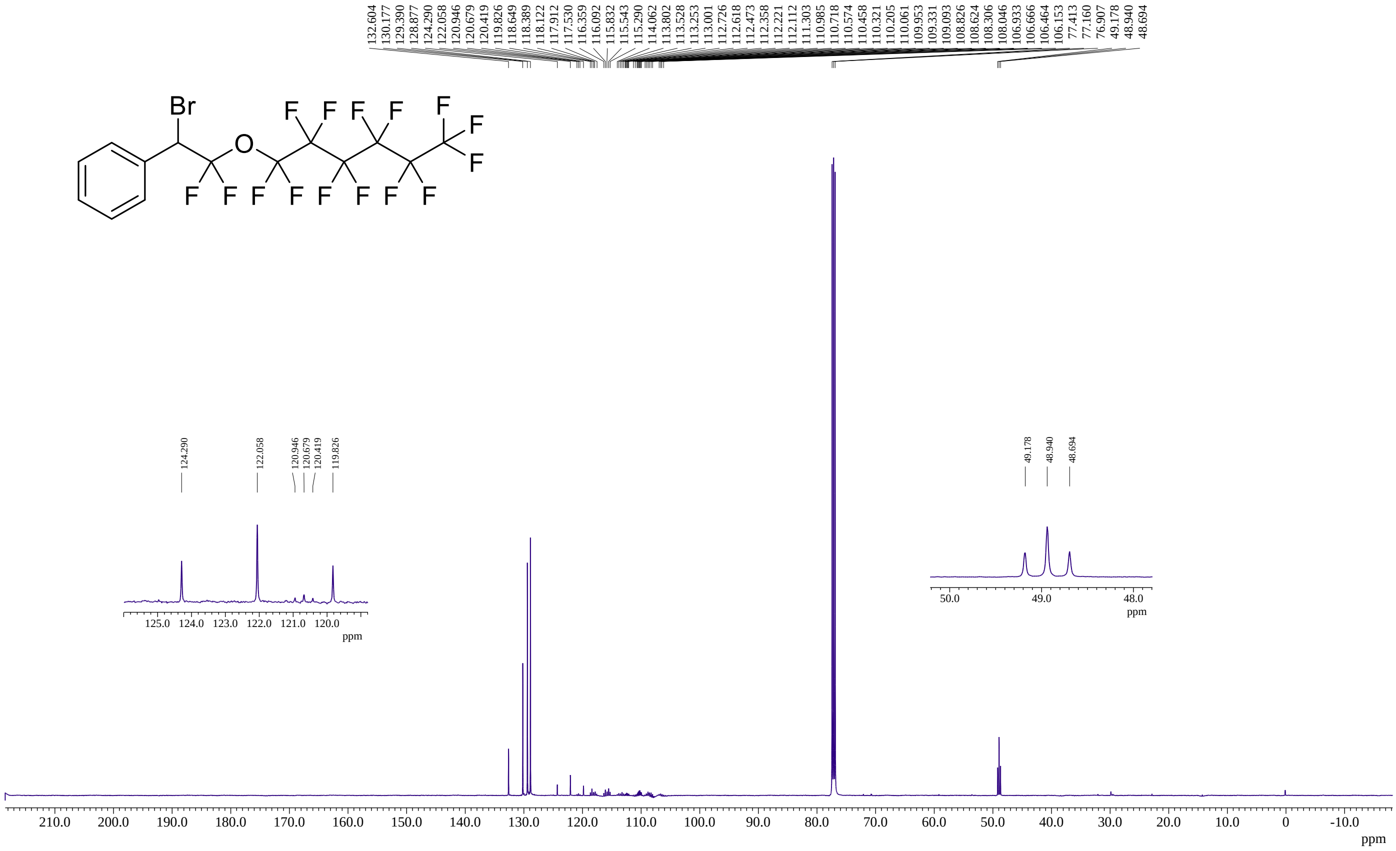

^{19}F NMR (282 MHz, $CDCl_3$): **4ha**

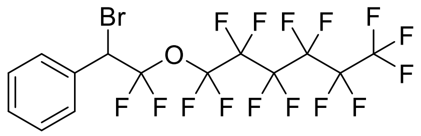

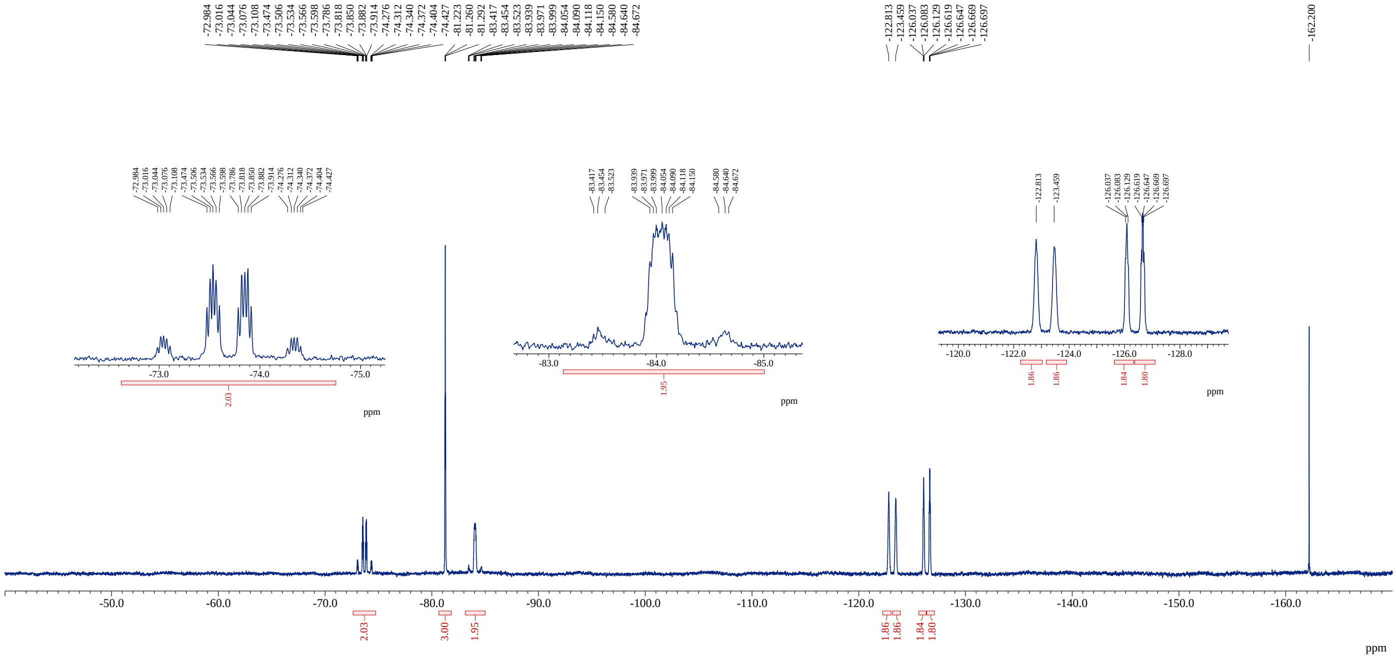

^{1}H NMR (500 MHz, $CDCl_3$): **4ia**

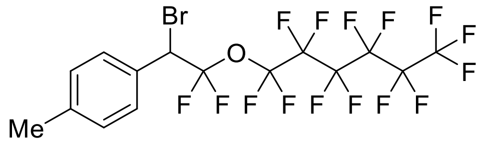

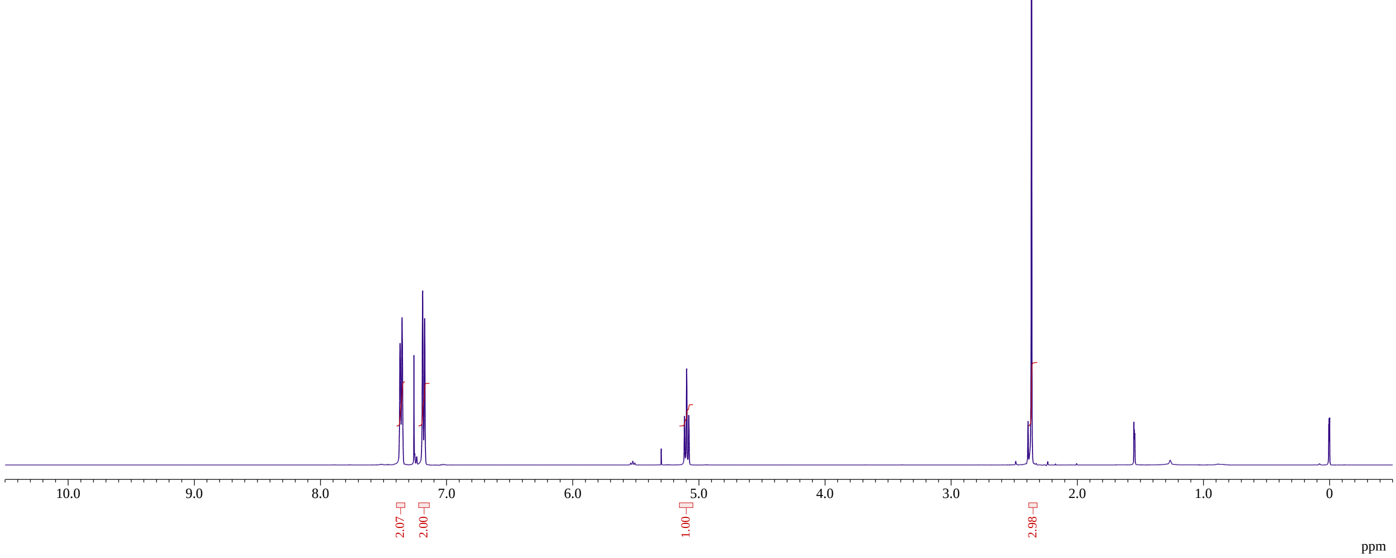

^{13}C NMR (126 MHz, $CDCl_3$): **4ia**

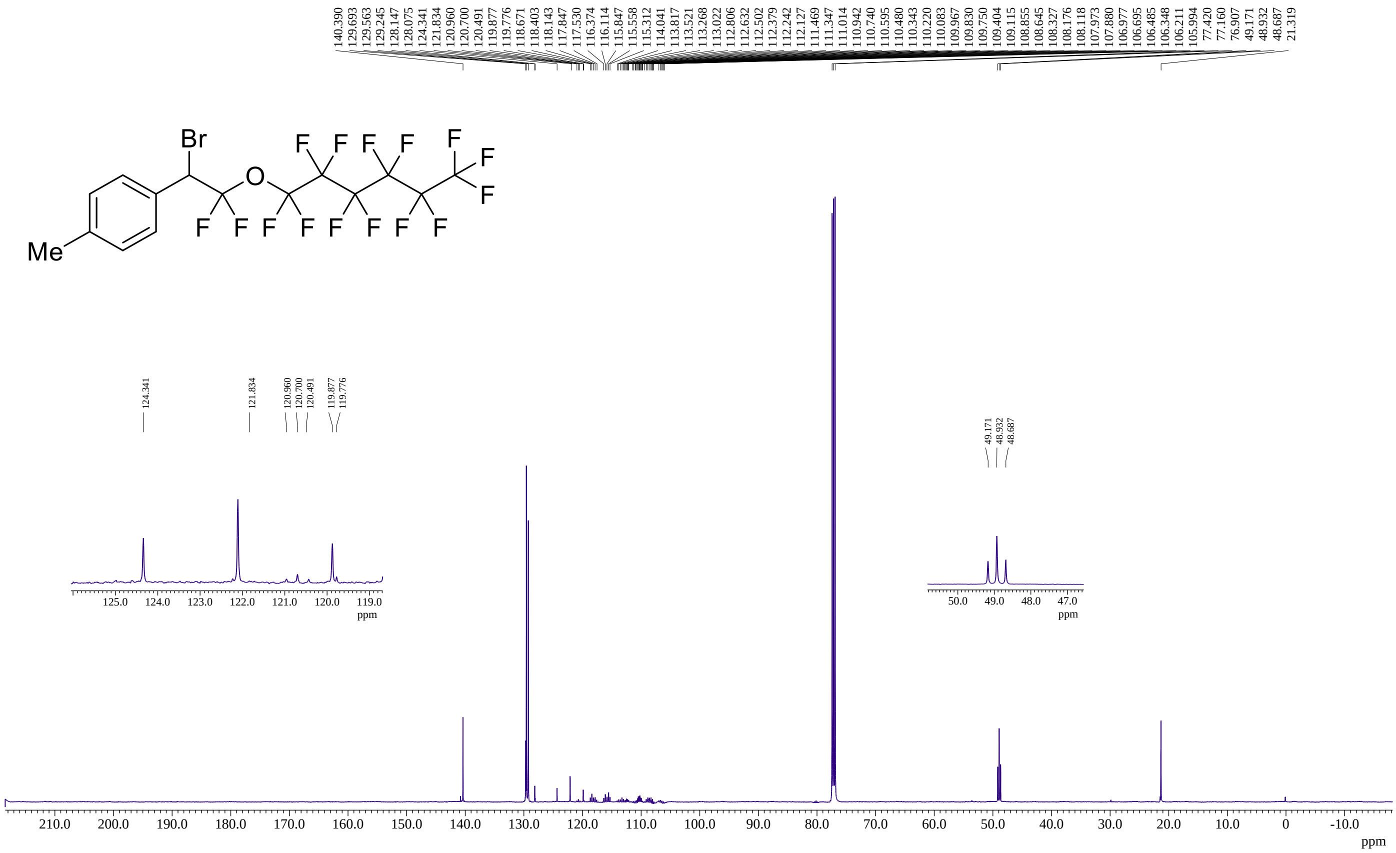

^{19}F NMR (282 MHz, $CDCl_3$): **4ia**

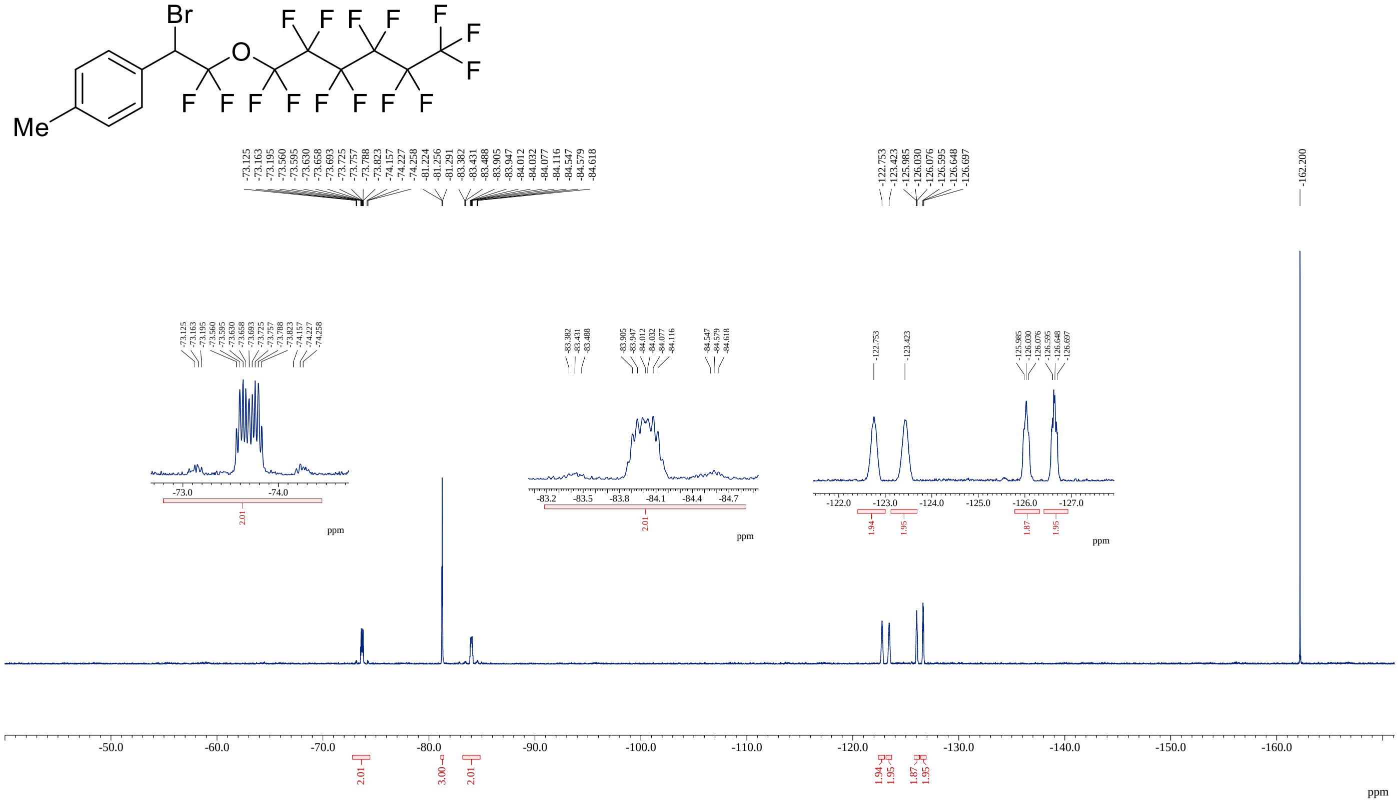

^{1}H NMR (500 MHz, $CDCl_3$): **4ja**

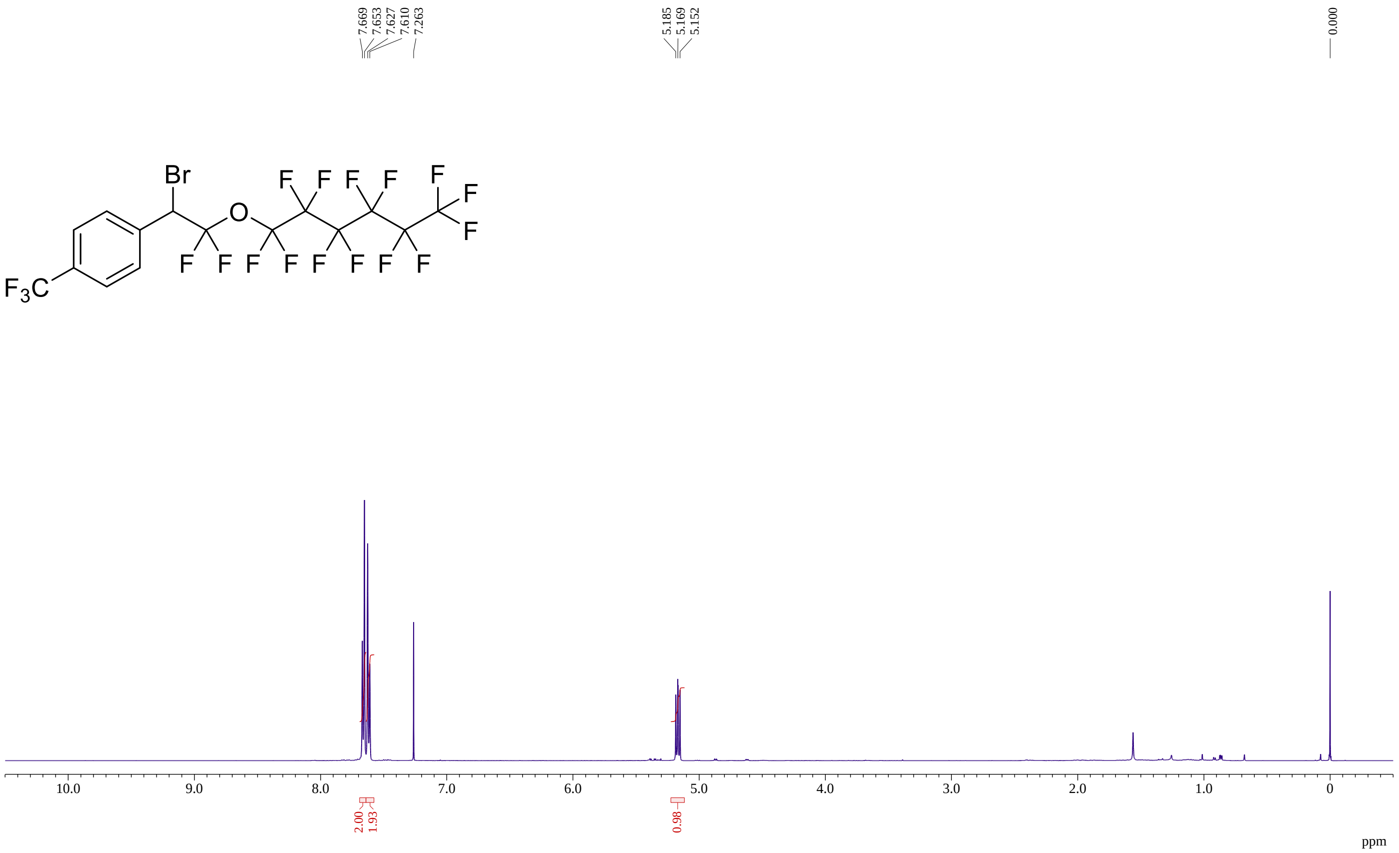

^{13}C NMR (126 MHz, $CDCl_3$): **4ja**

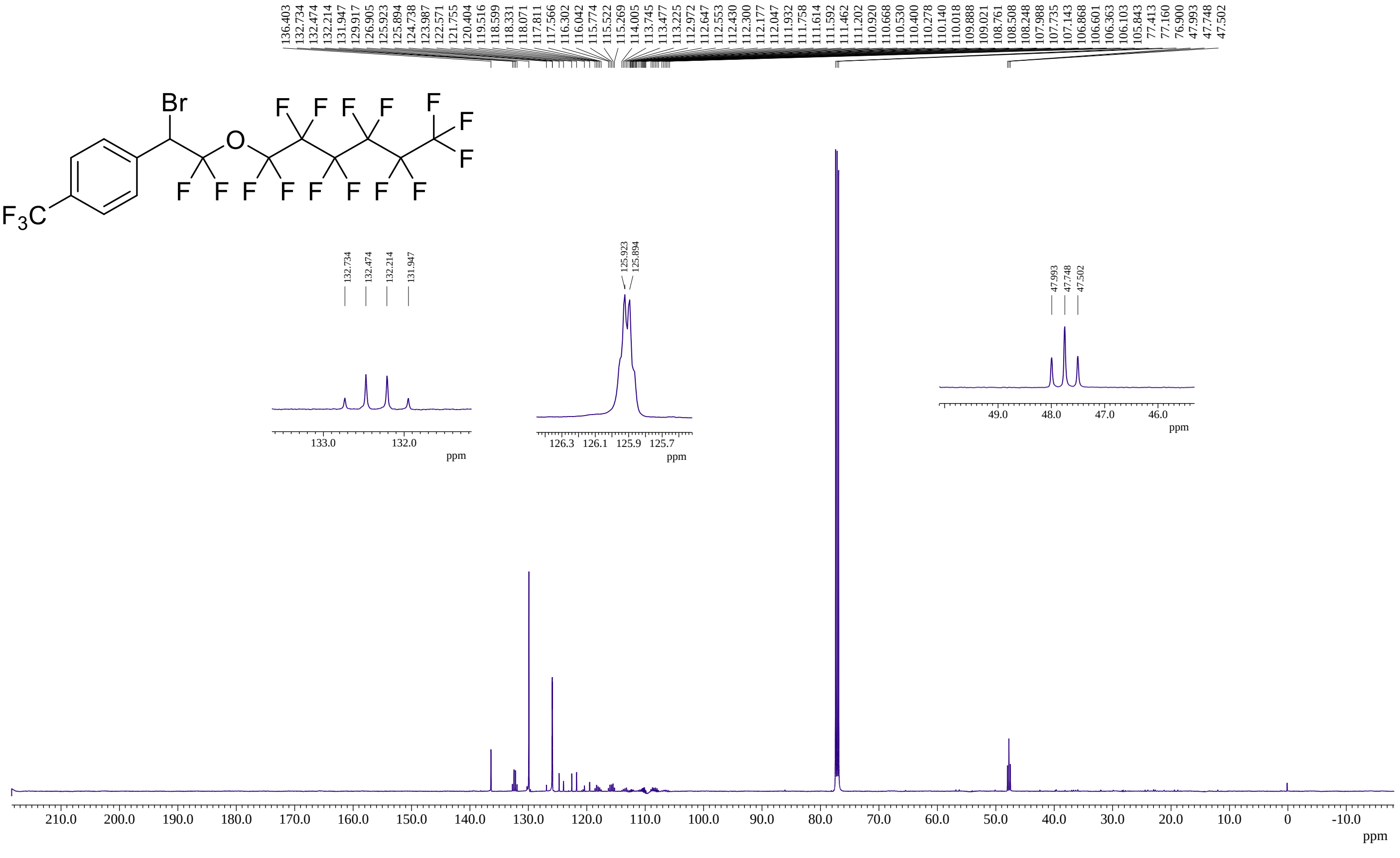

^{19}F NMR (282 MHz, $CDCl_3$): **4ja**

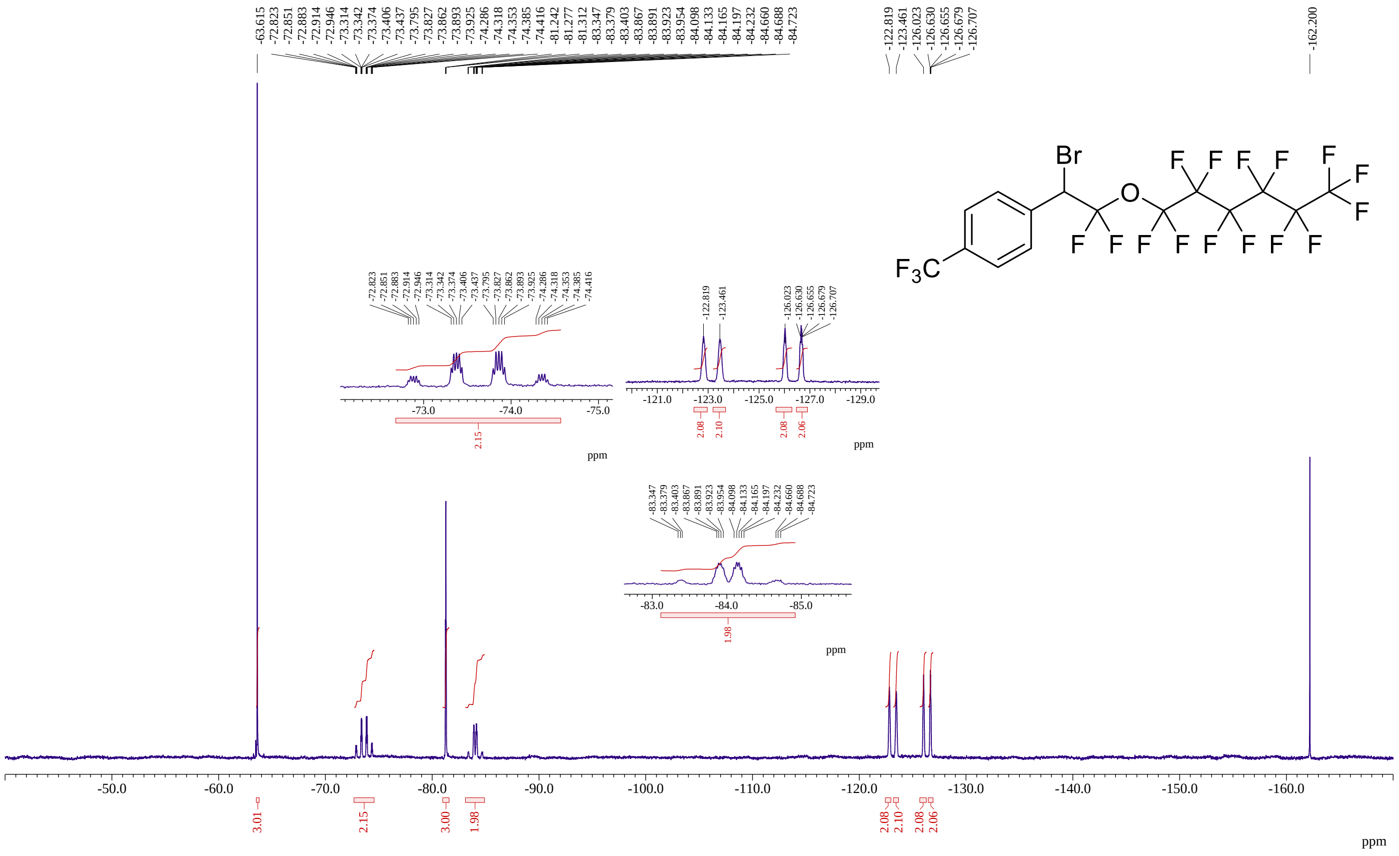

^{1}H NMR (500 MHz, $CDCl_3$): **4ka**

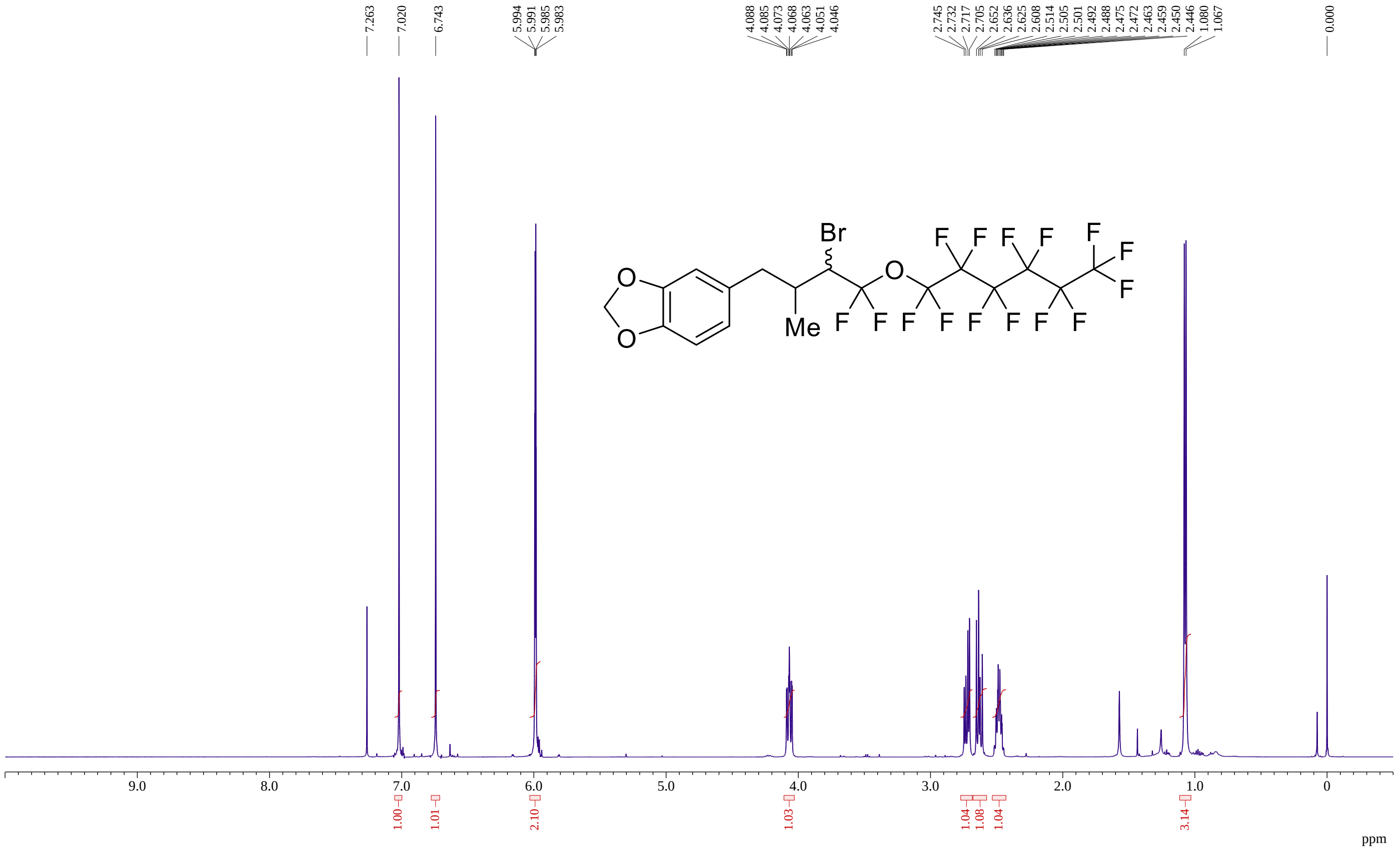

^{13}C NMR (126 MHz, $CDCl_3$): **4ka**

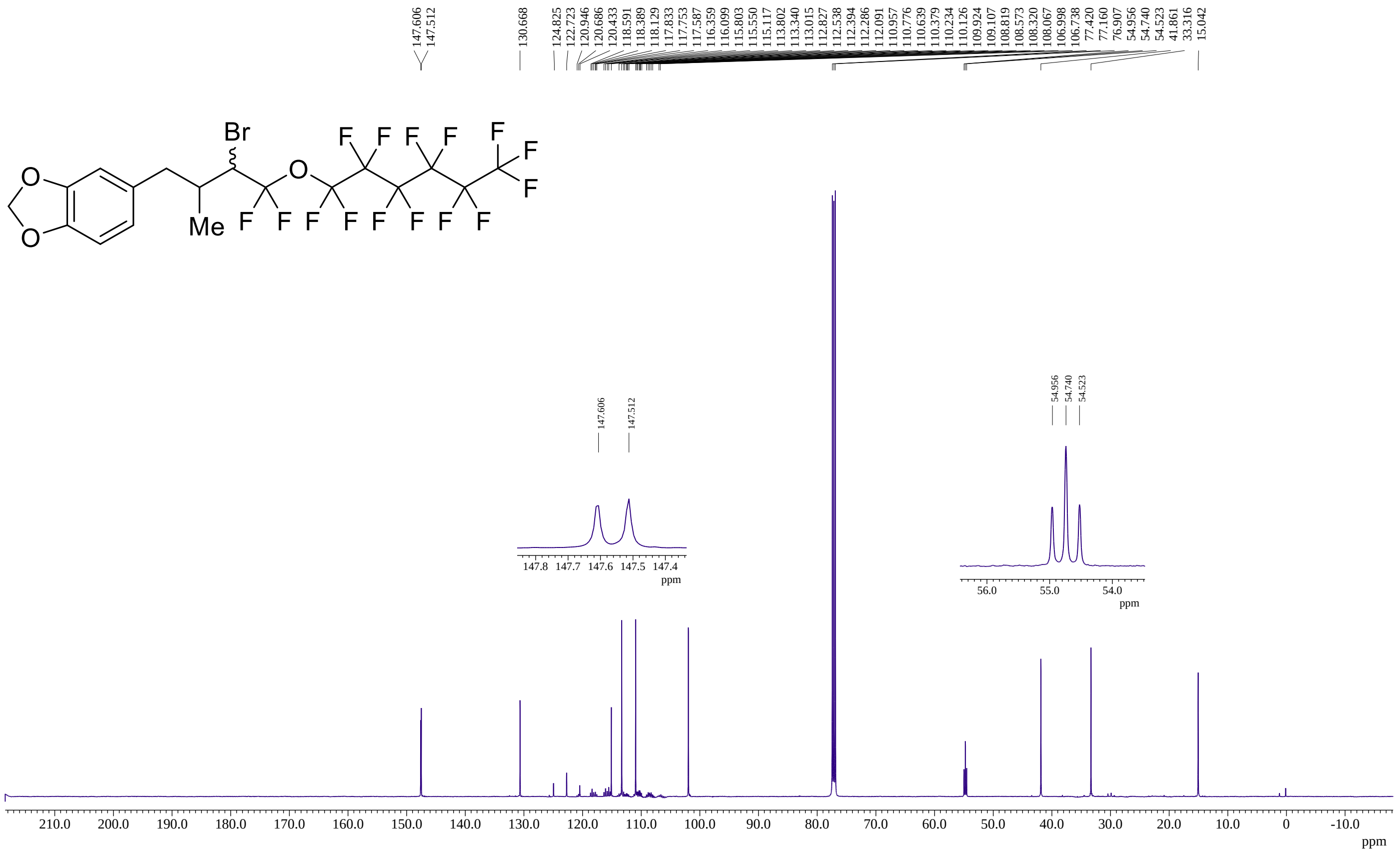

^{19}F NMR (282 MHz, $CDCl_3$): **4ka**

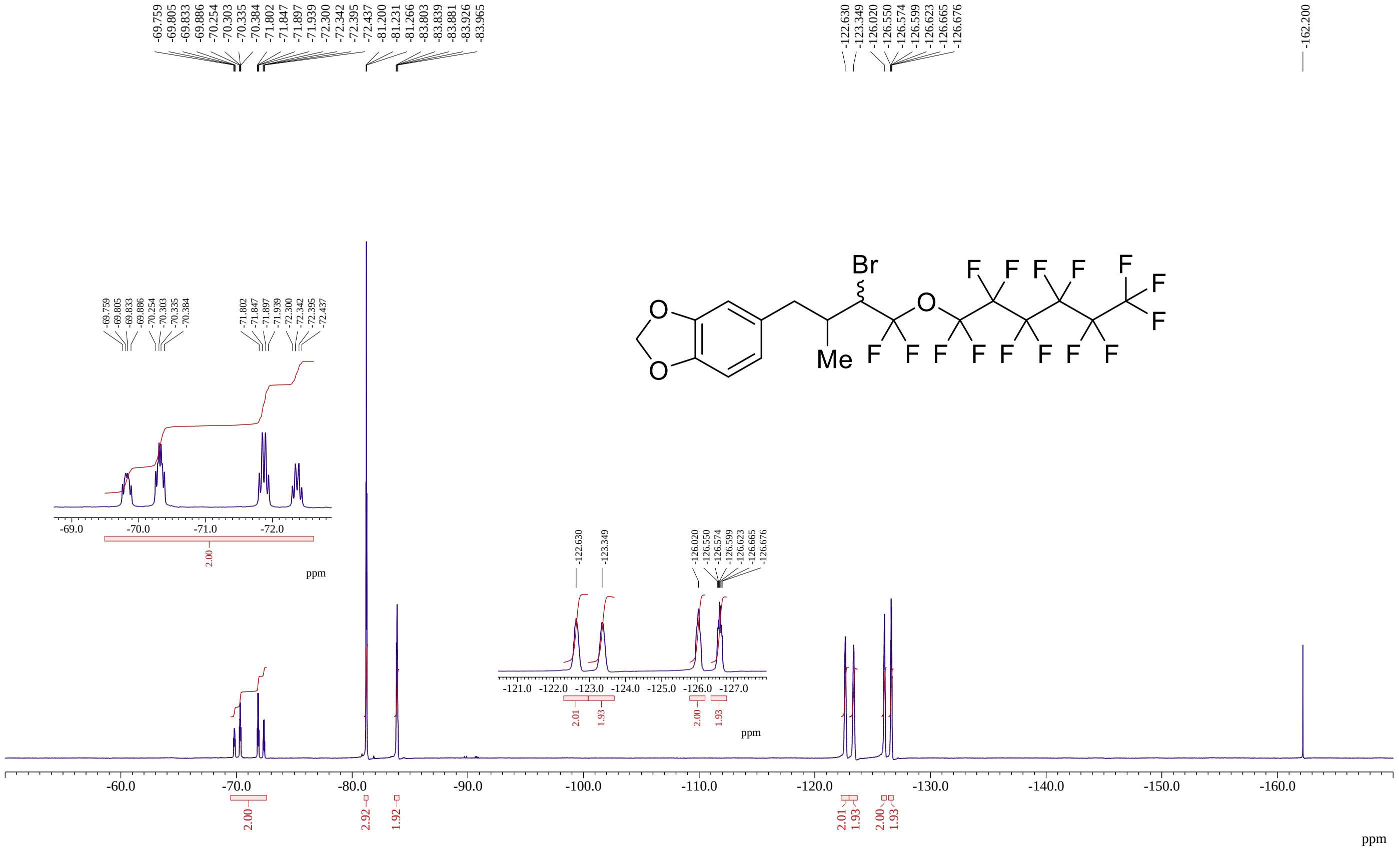

^{1}H NMR (500 MHz, $CDCl_3$): **4la**

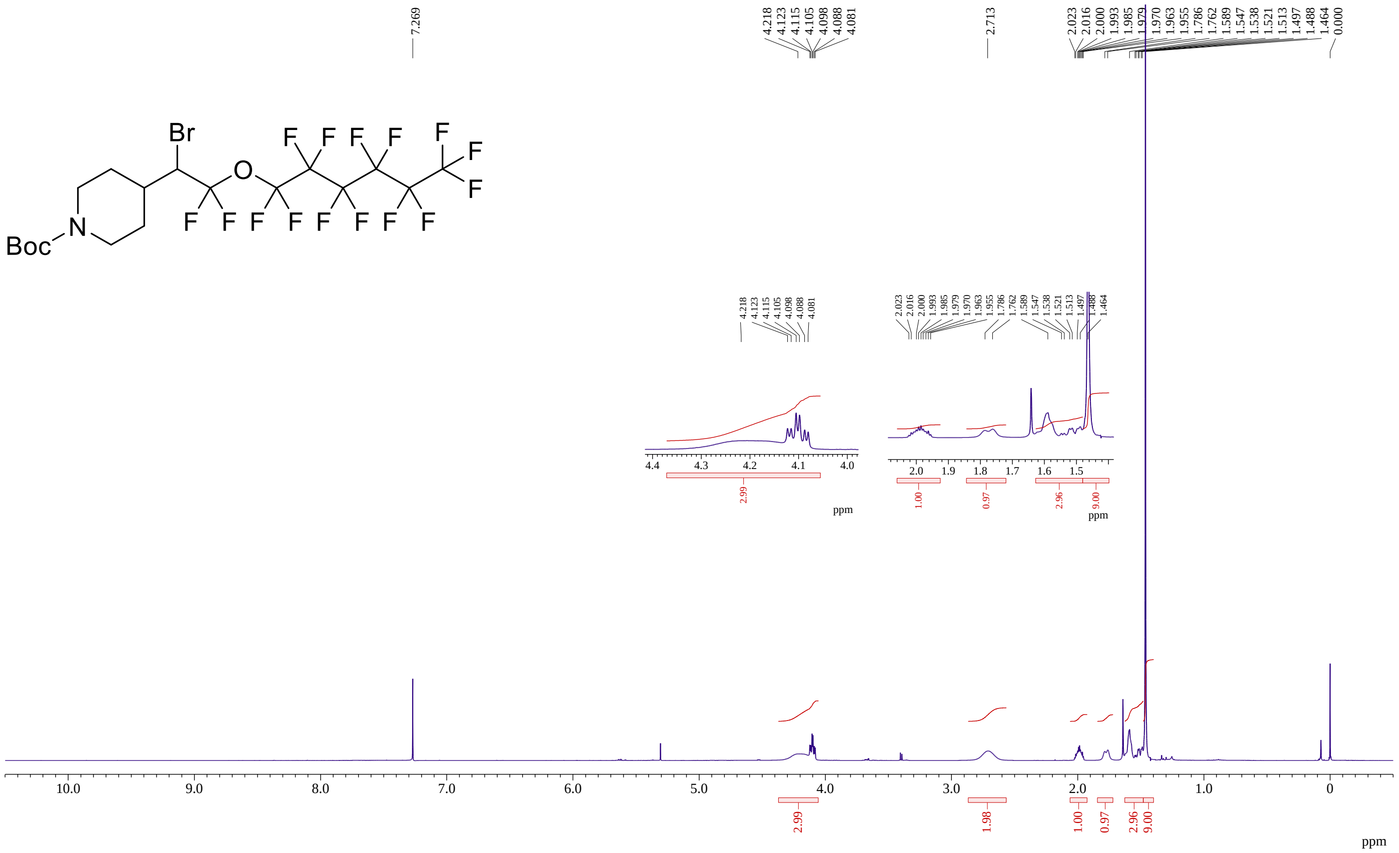

^{13}C NMR (126 MHz, $CDCl_3$): **4la**

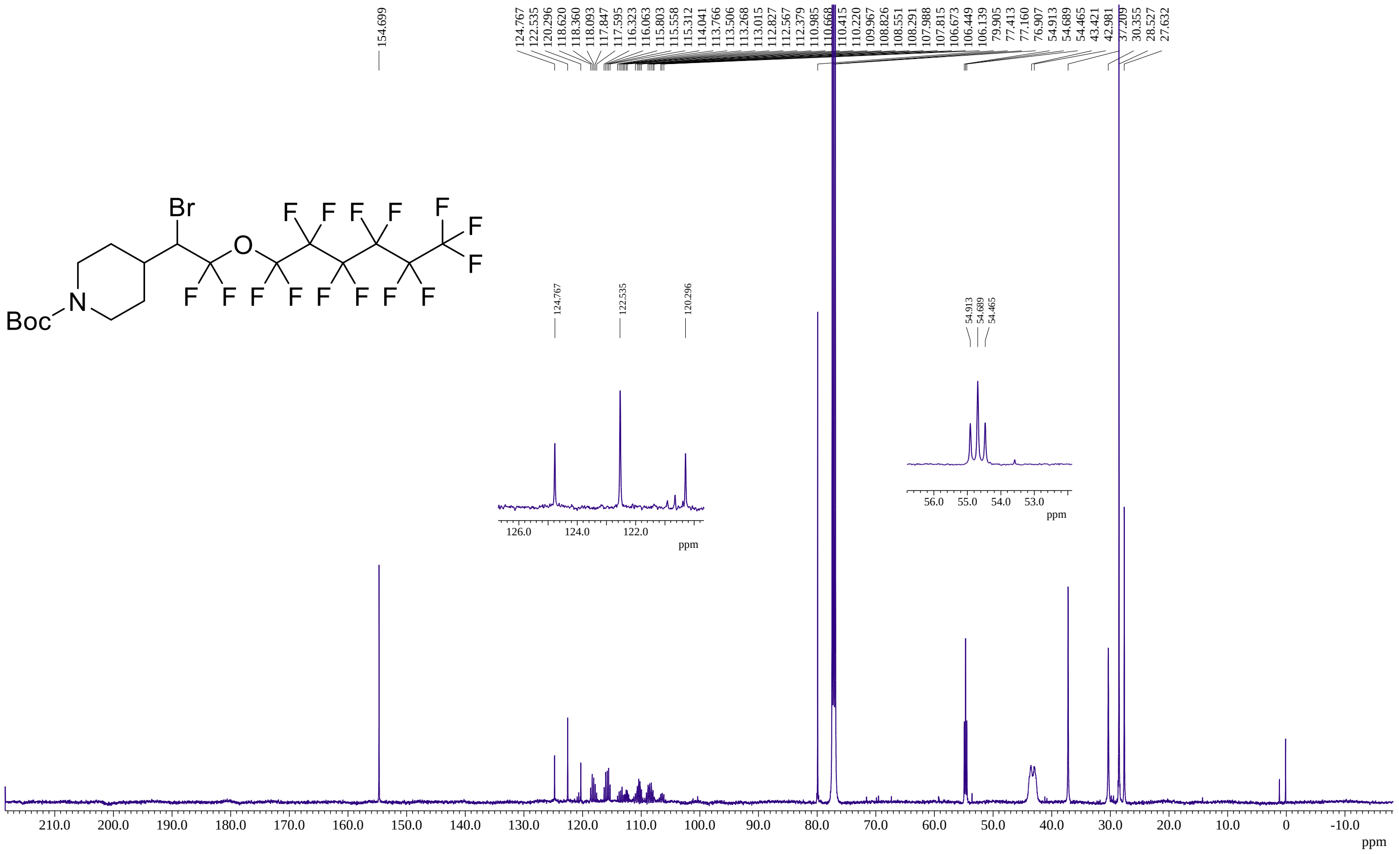

^{19}F NMR (282 MHz, $CDCl_3$): **4la**

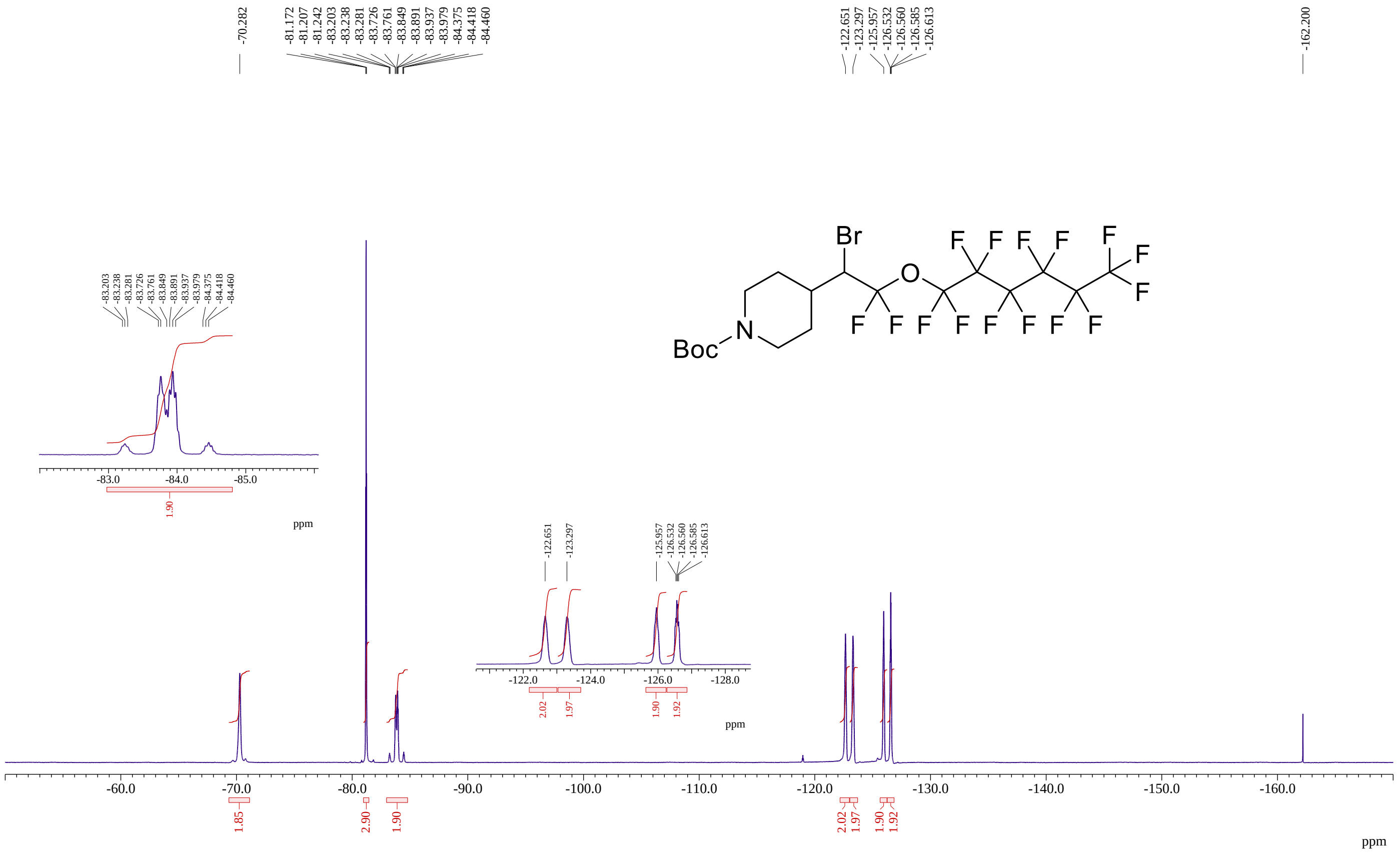

^{1}H NMR (500 MHz, $CDCl_3$): **4ma**

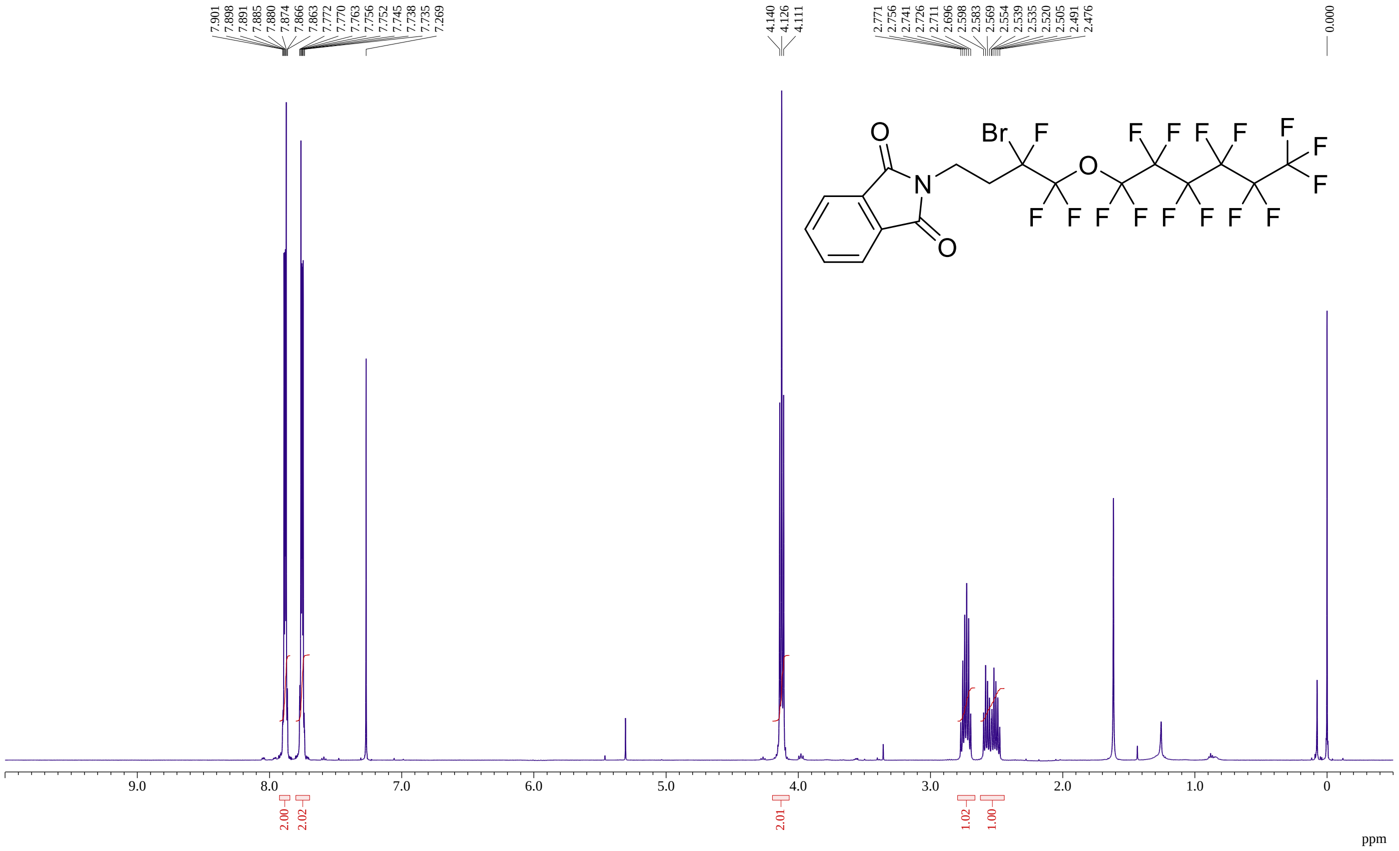

^{13}C NMR (126 MHz, $CDCl_3$): **4ma**

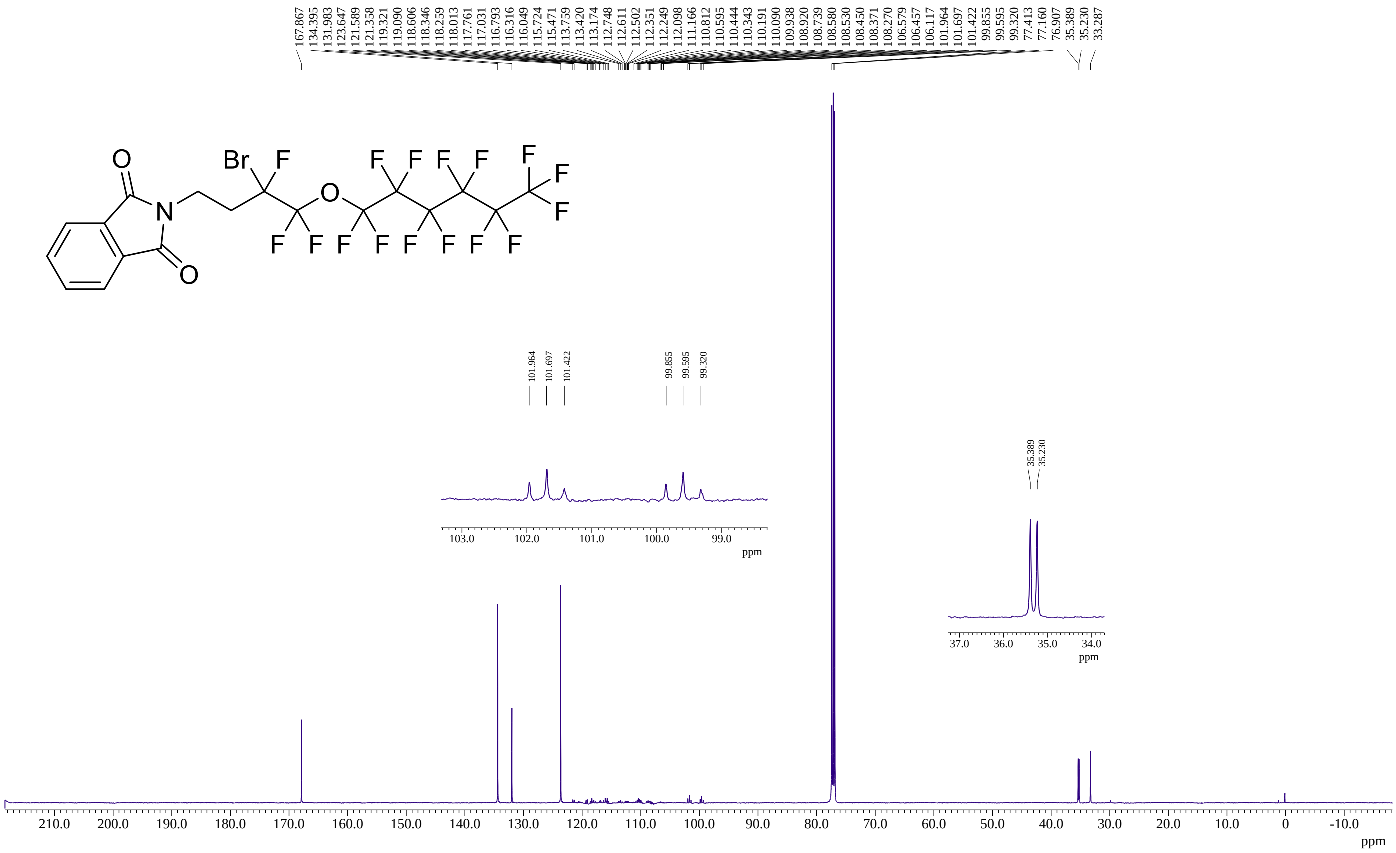

^{19}F NMR (282 MHz, $CDCl_3$): **4ma**

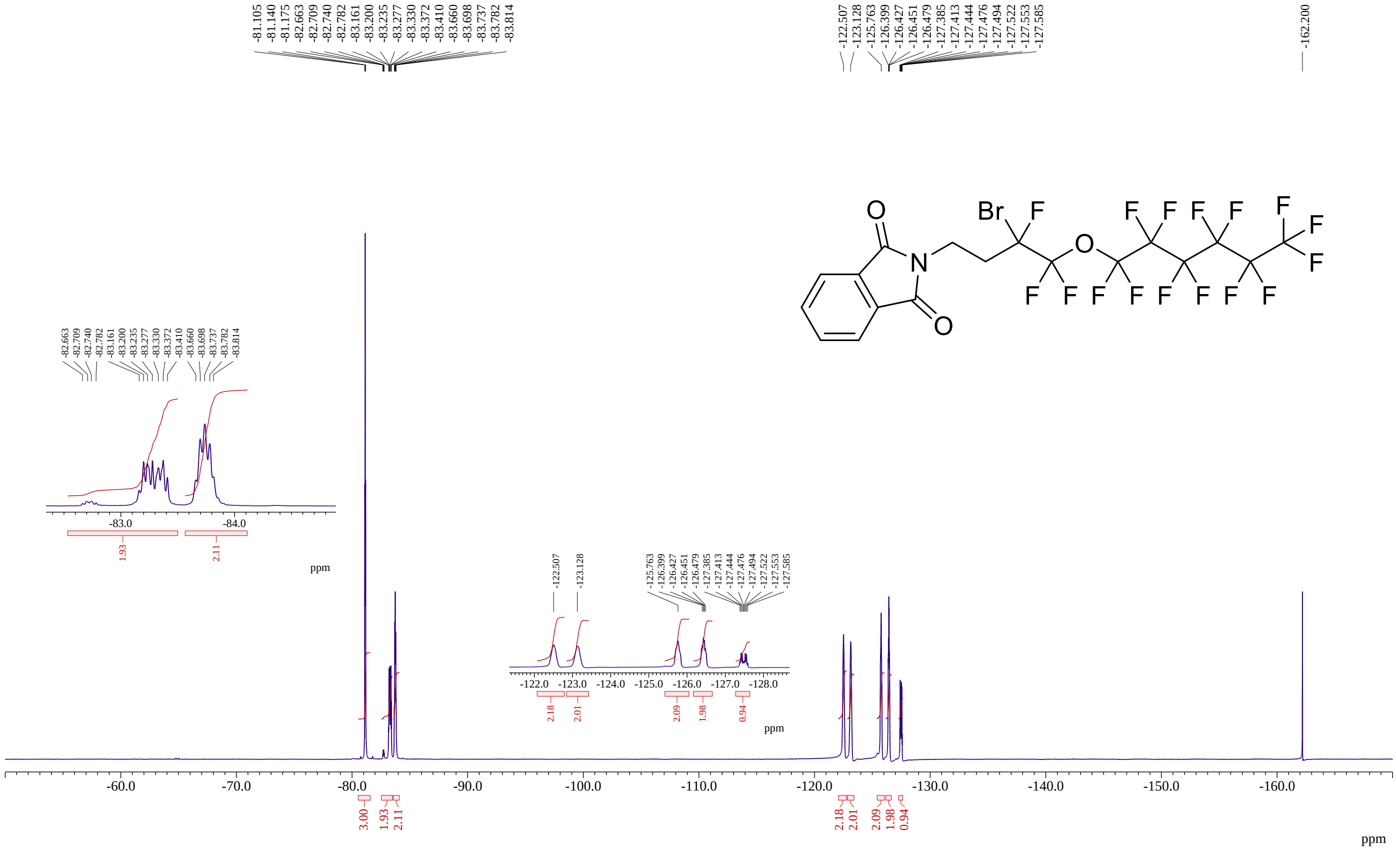

^{1}H NMR (500 MHz, CDCl$_3$): **4sa**

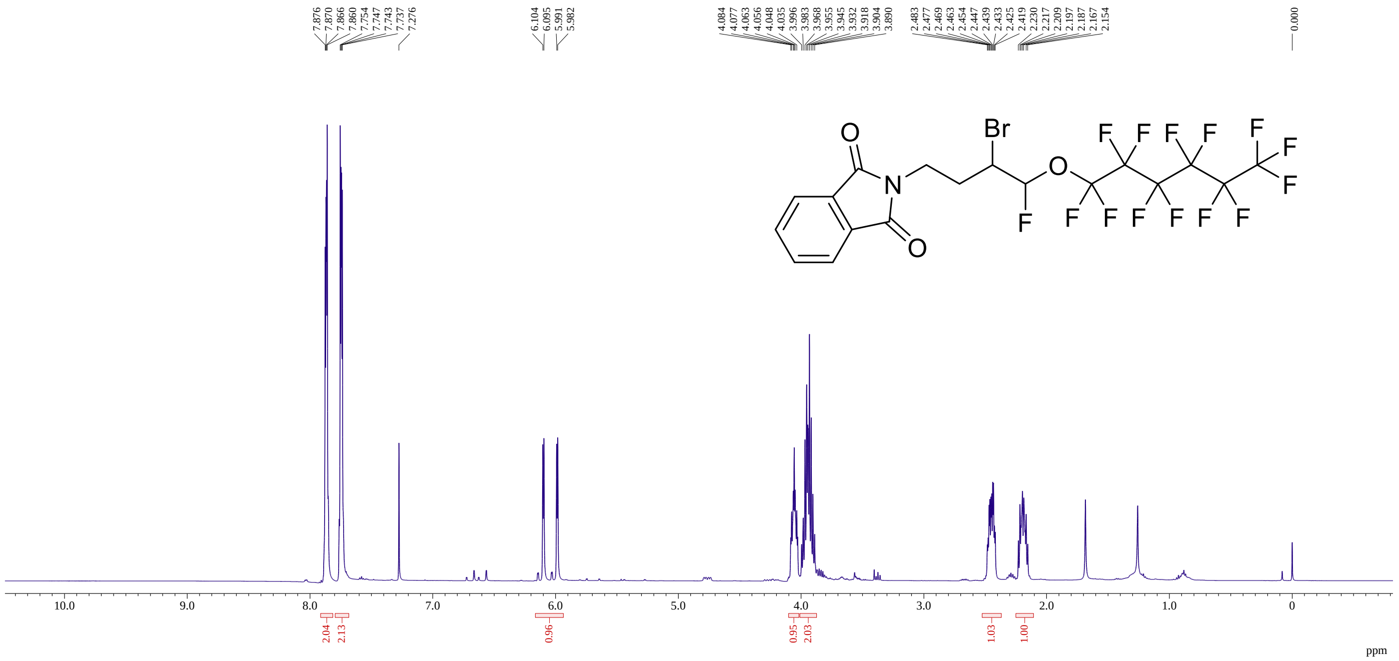

^{13}C NMR (126 MHz, CDCl$_3$): **4sa**

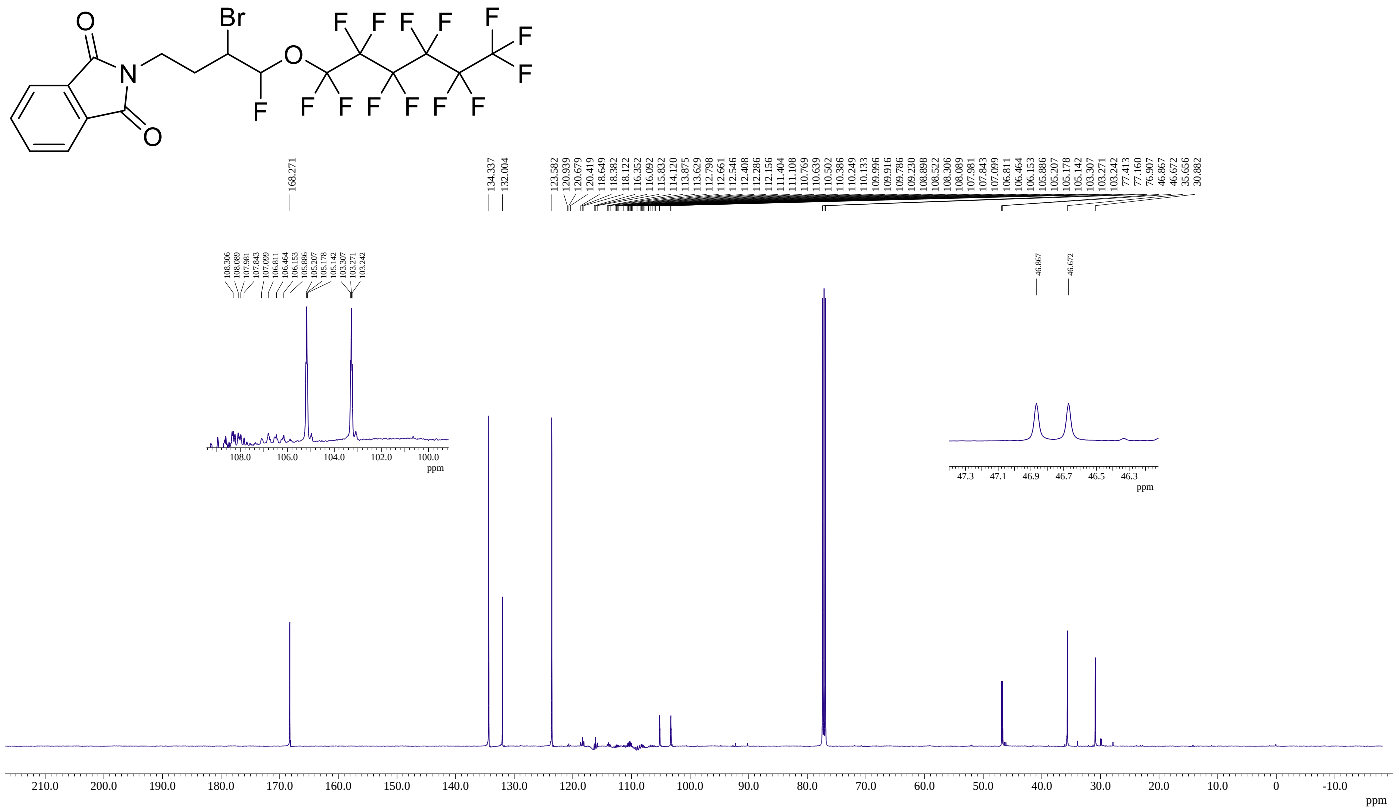

^{19}F NMR (282 MHz, $CDCl_3$): **4sa**

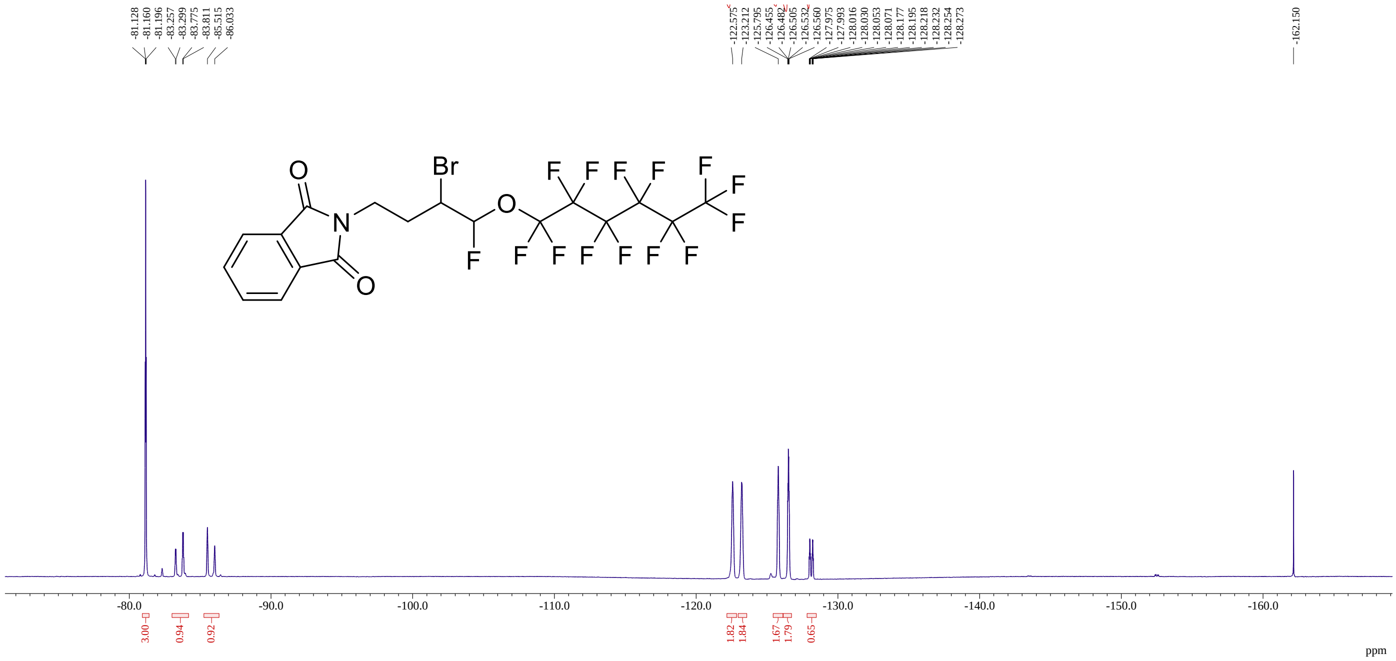

^{1}H NMR (500 MHz, $CDCl_3$): **4ta**

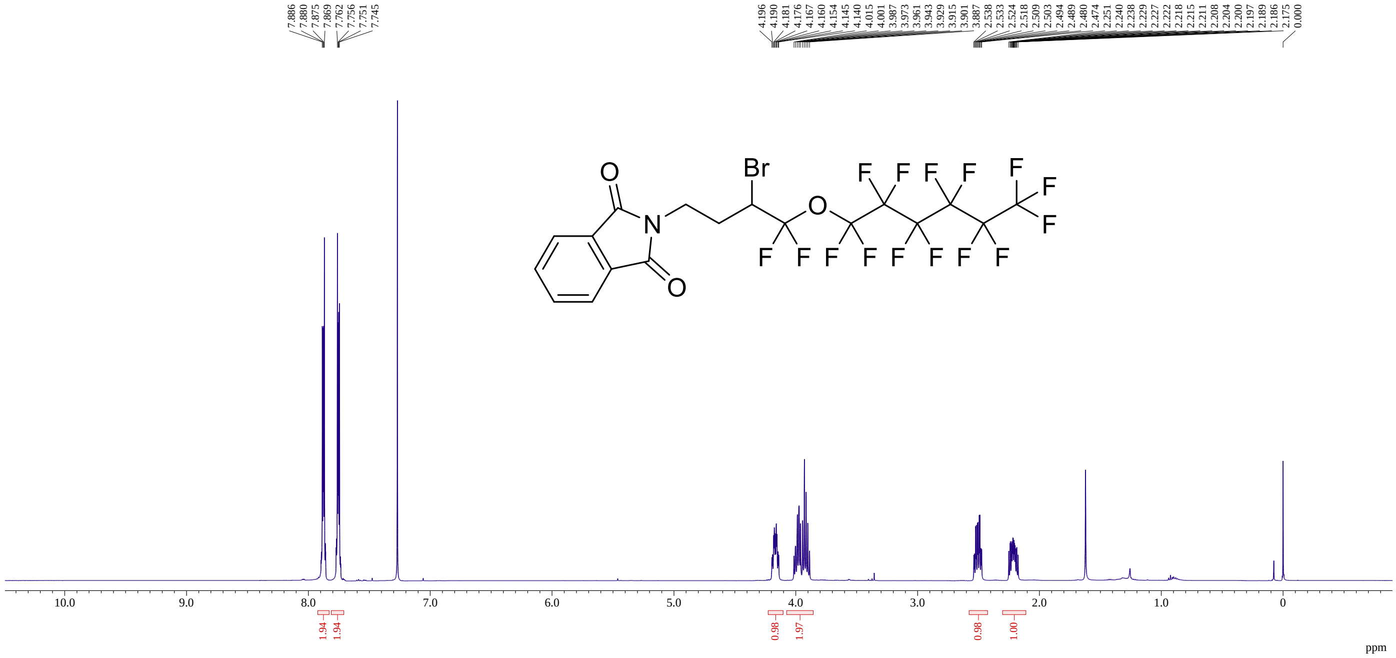

^{13}C NMR (126 MHz, $CDCl_3$): **4ta**

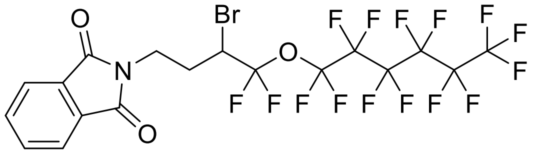

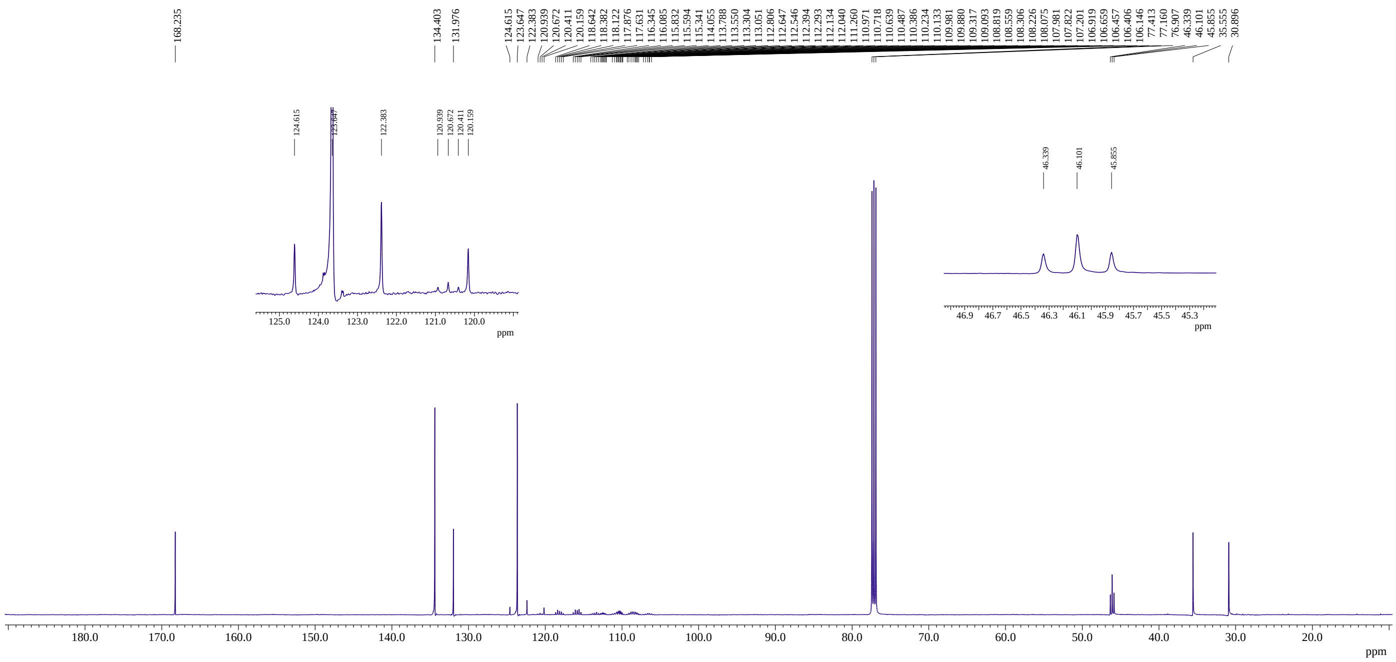

^{19}F NMR (282 MHz, $CDCl_3$): **4ta**

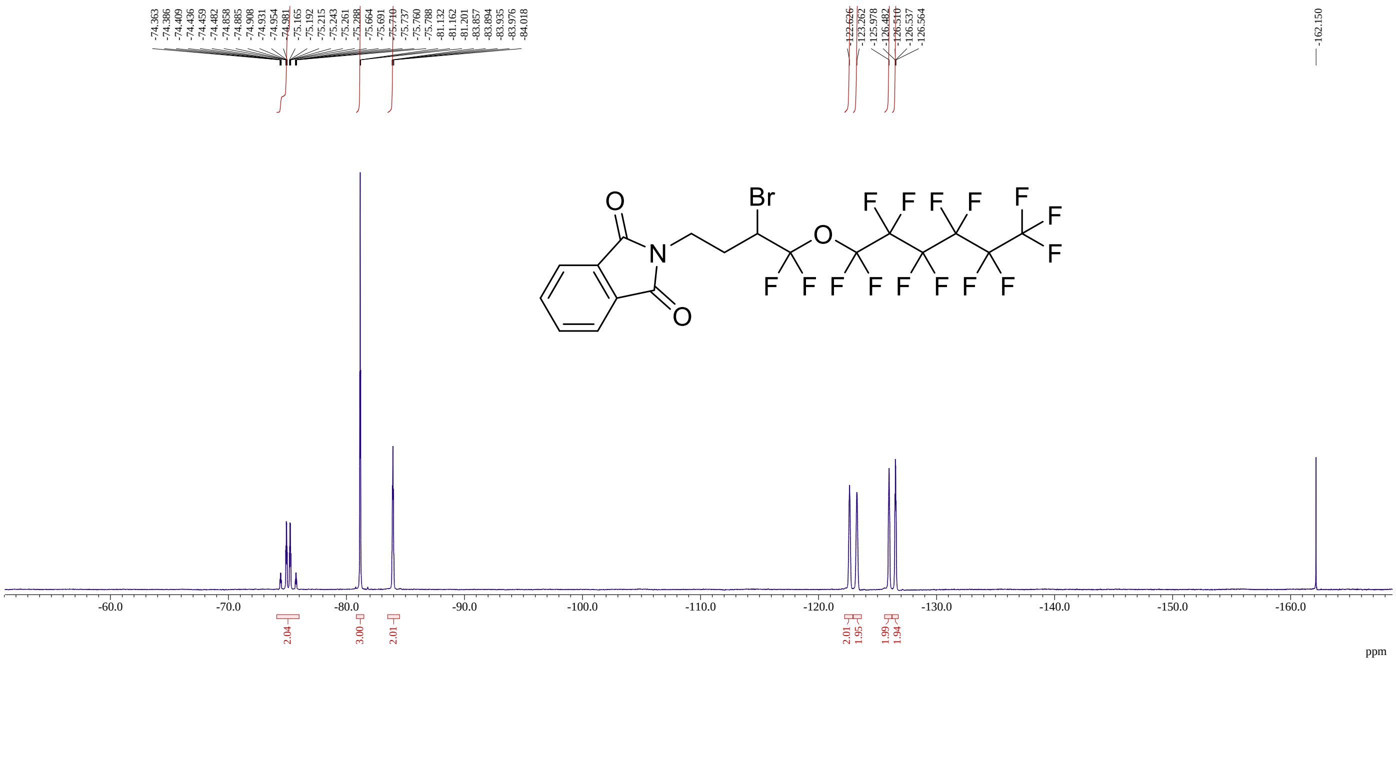

^{1}H NMR (500 MHz, $CDCl_3$): **4ab**

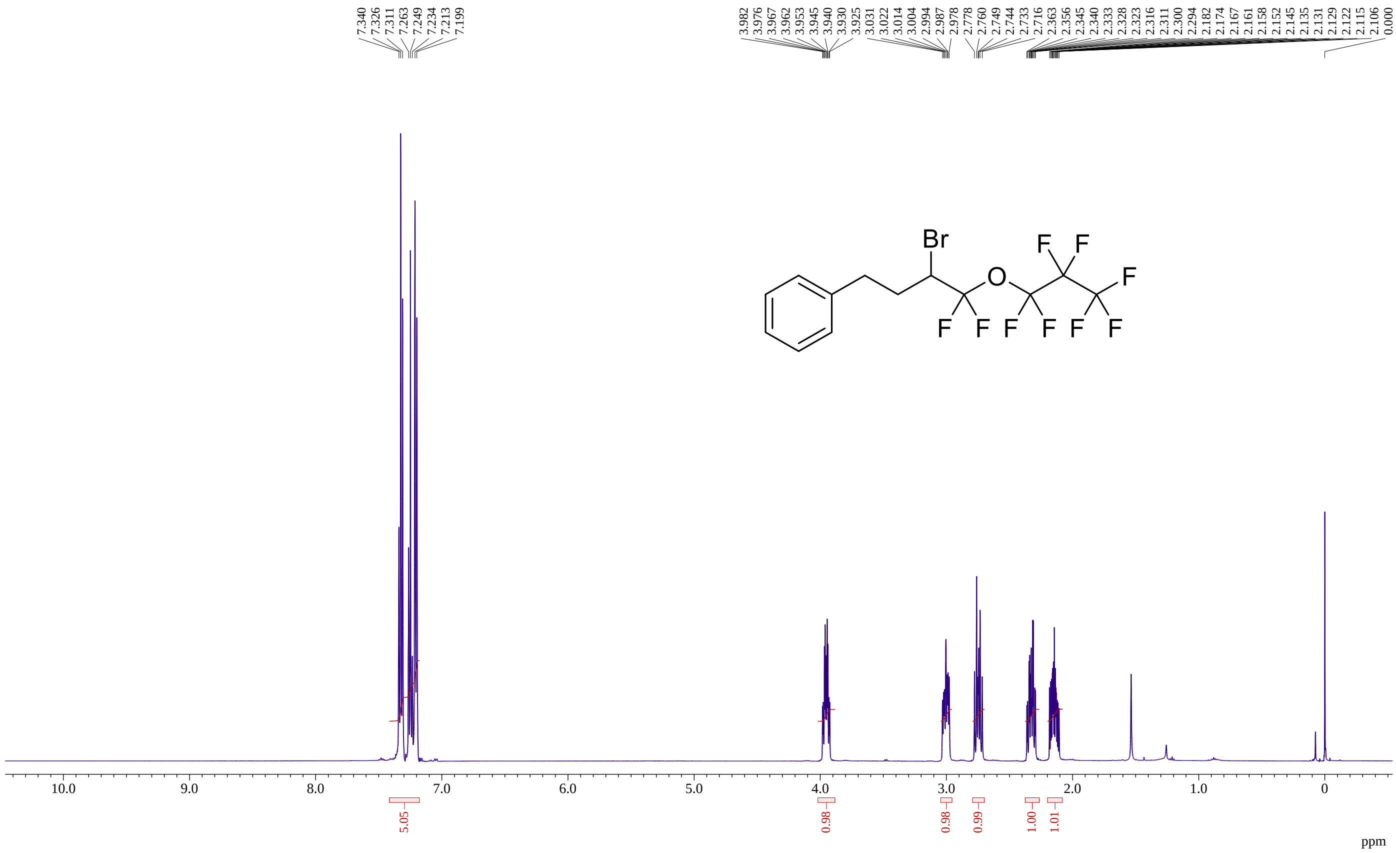

^{13}C NMR (126 MHz, $CDCl_3$): **4ab**

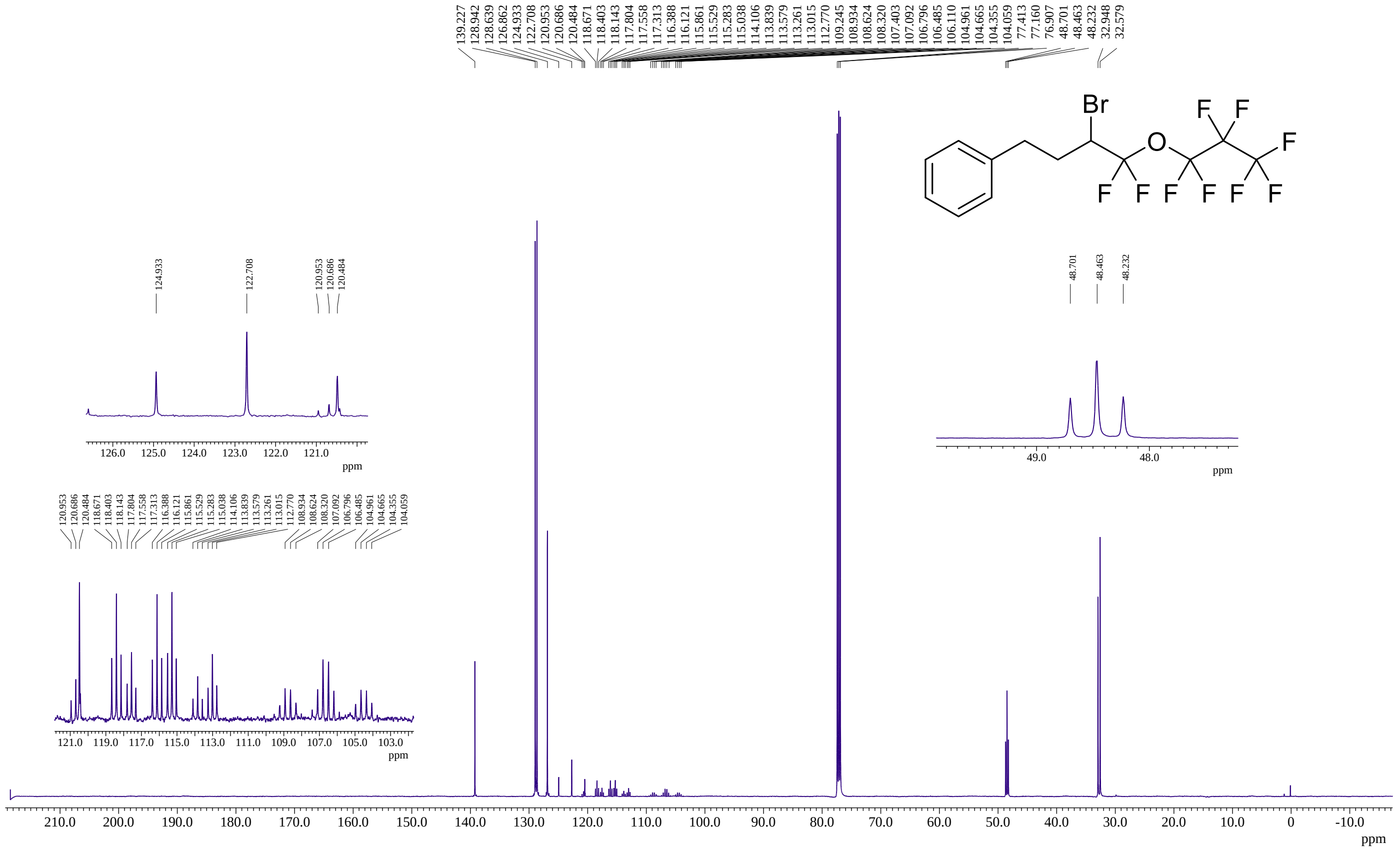

^{19}F NMR (282 MHz, $CDCl_3$): **4ab**

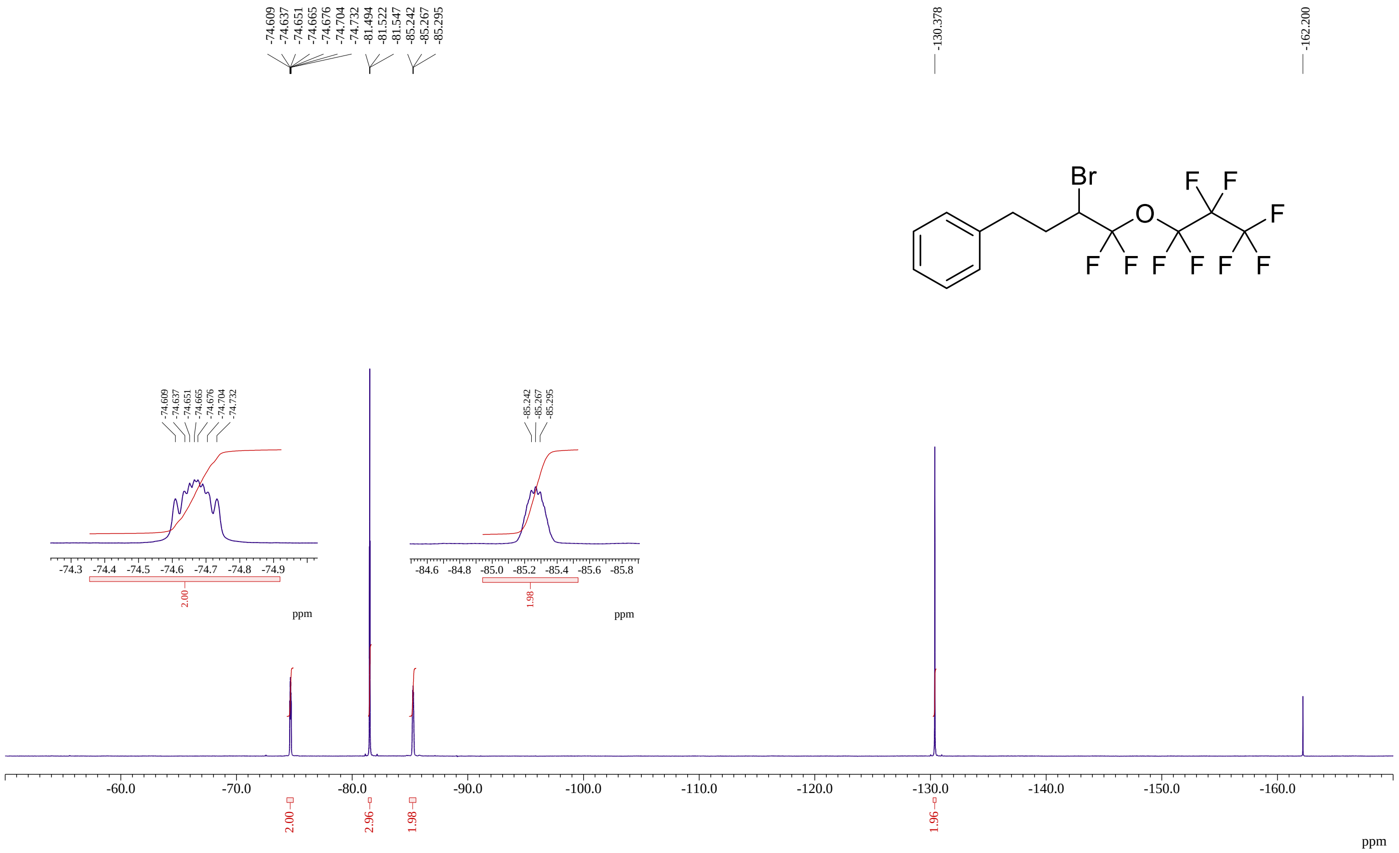

^{1}H NMR (500 MHz, $CDCl_3$): **4ac**

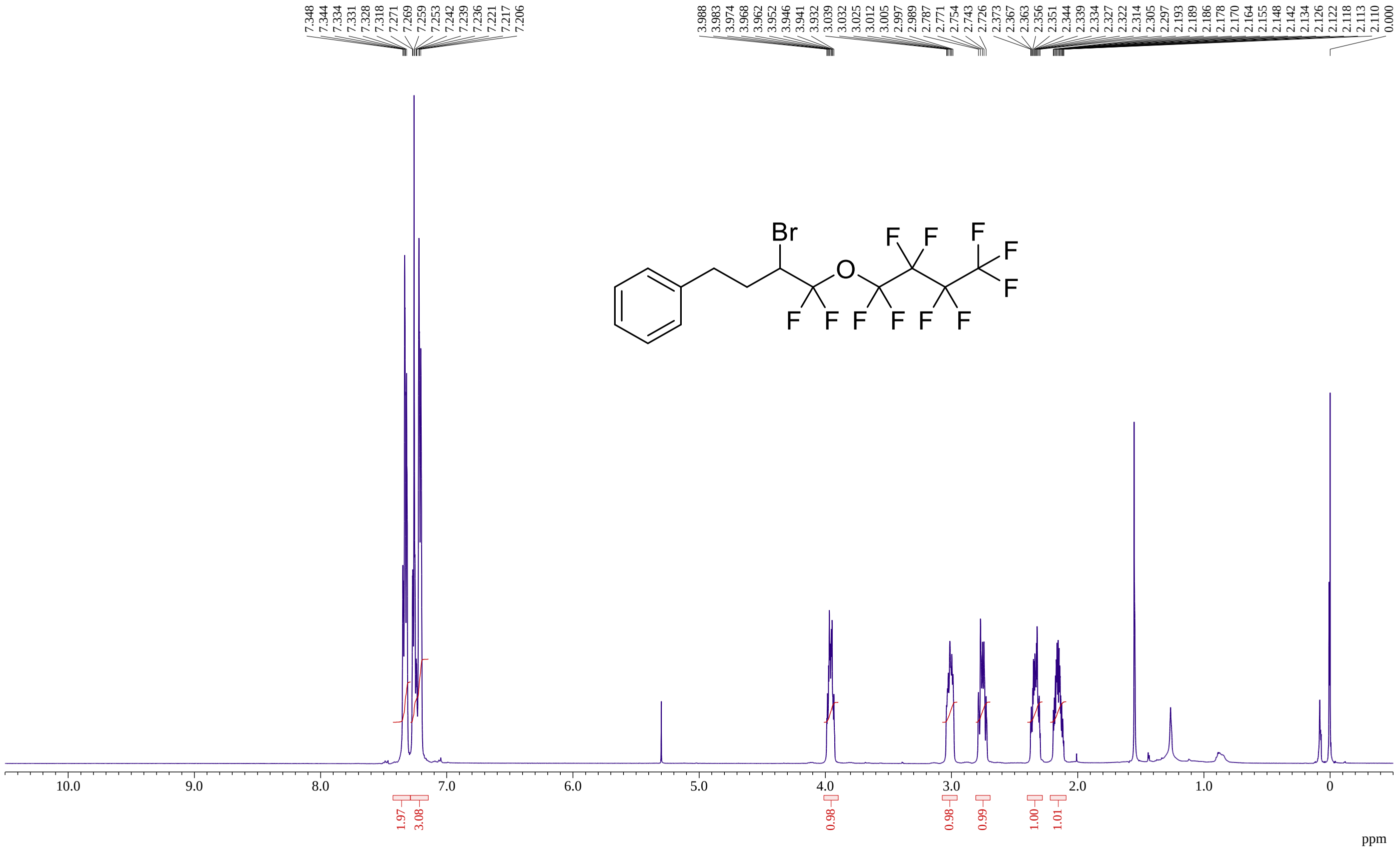

^{13}C NMR (126 MHz, $CDCl_3$): **4ac**

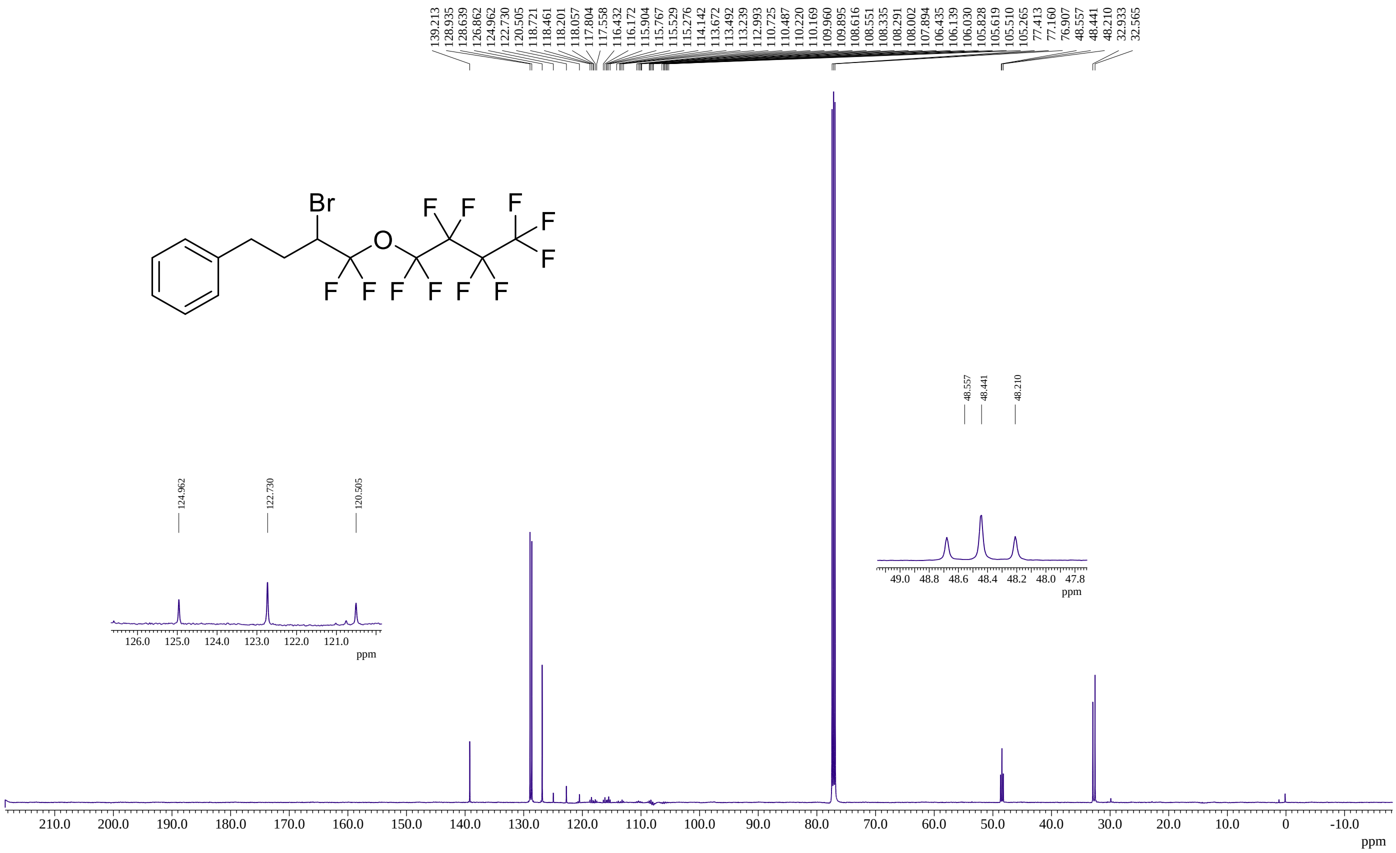

^{19}F NMR (282 MHz, $CDCl_3$): **4ac**

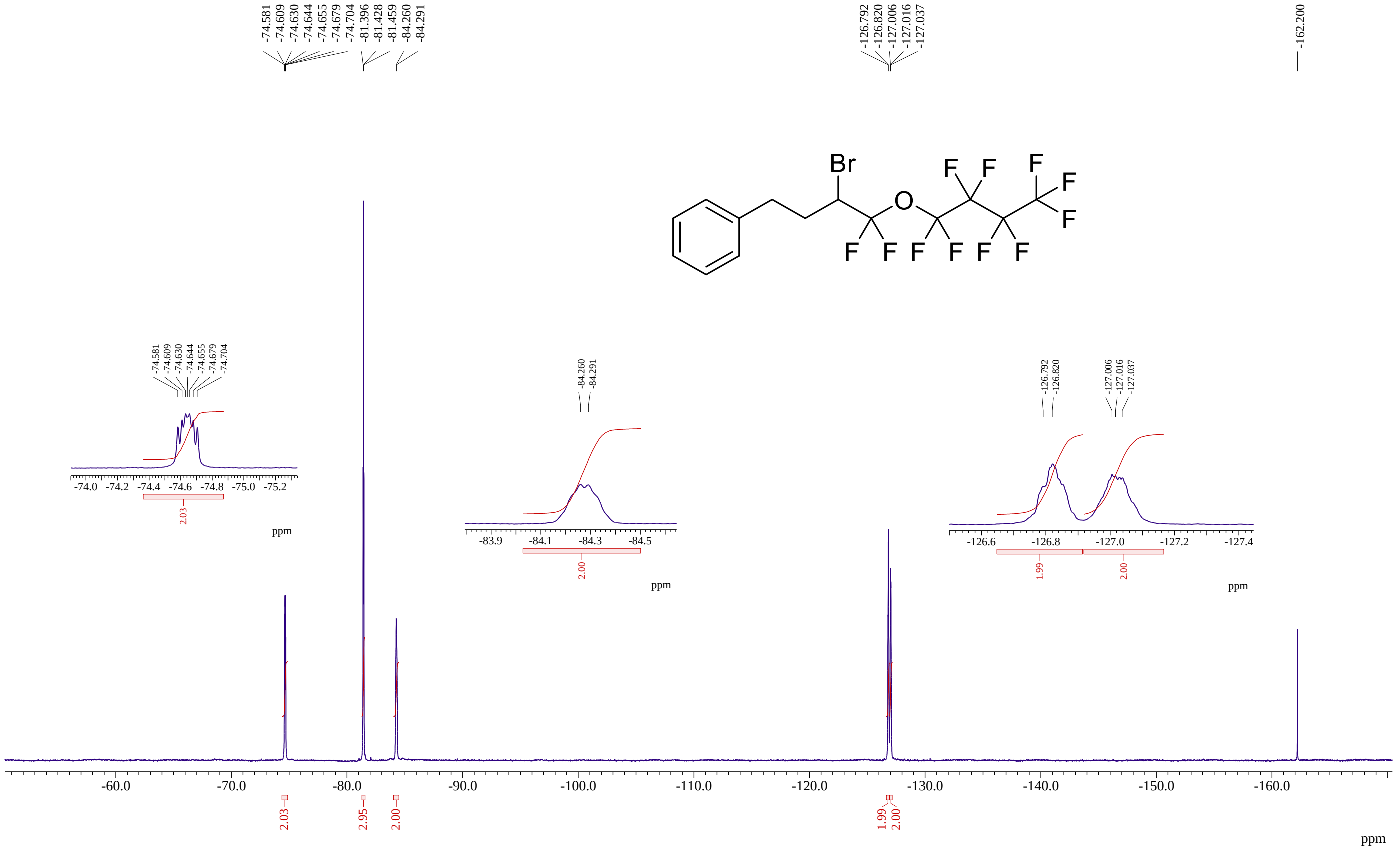

^{1}H NMR (500 MHz, $CDCl_3$): **4ad**

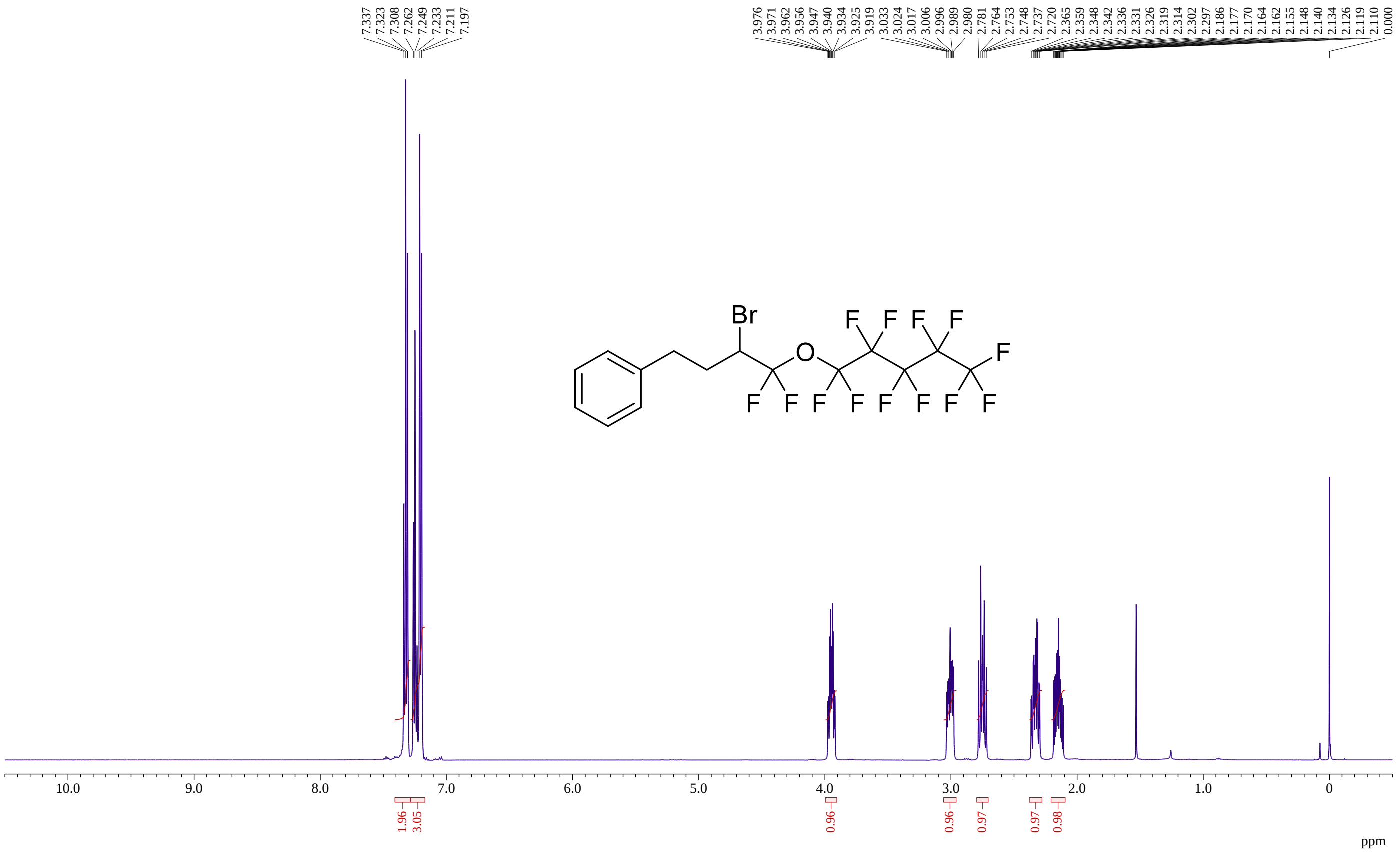

^{13}C NMR (126 MHz, $CDCl_3$): **4ad**

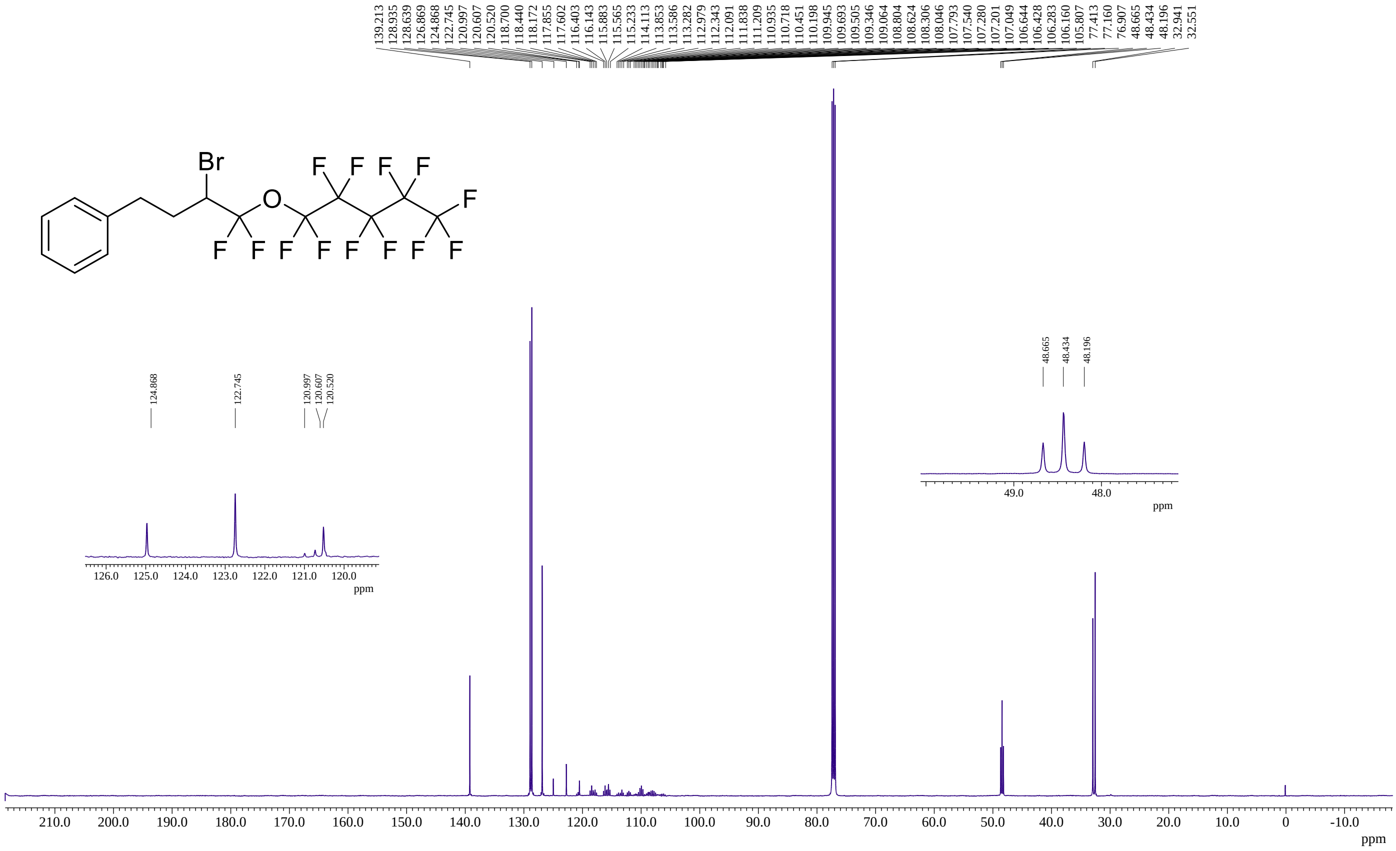

^{19}F NMR (282 MHz, $CDCl_3$): **4ad**

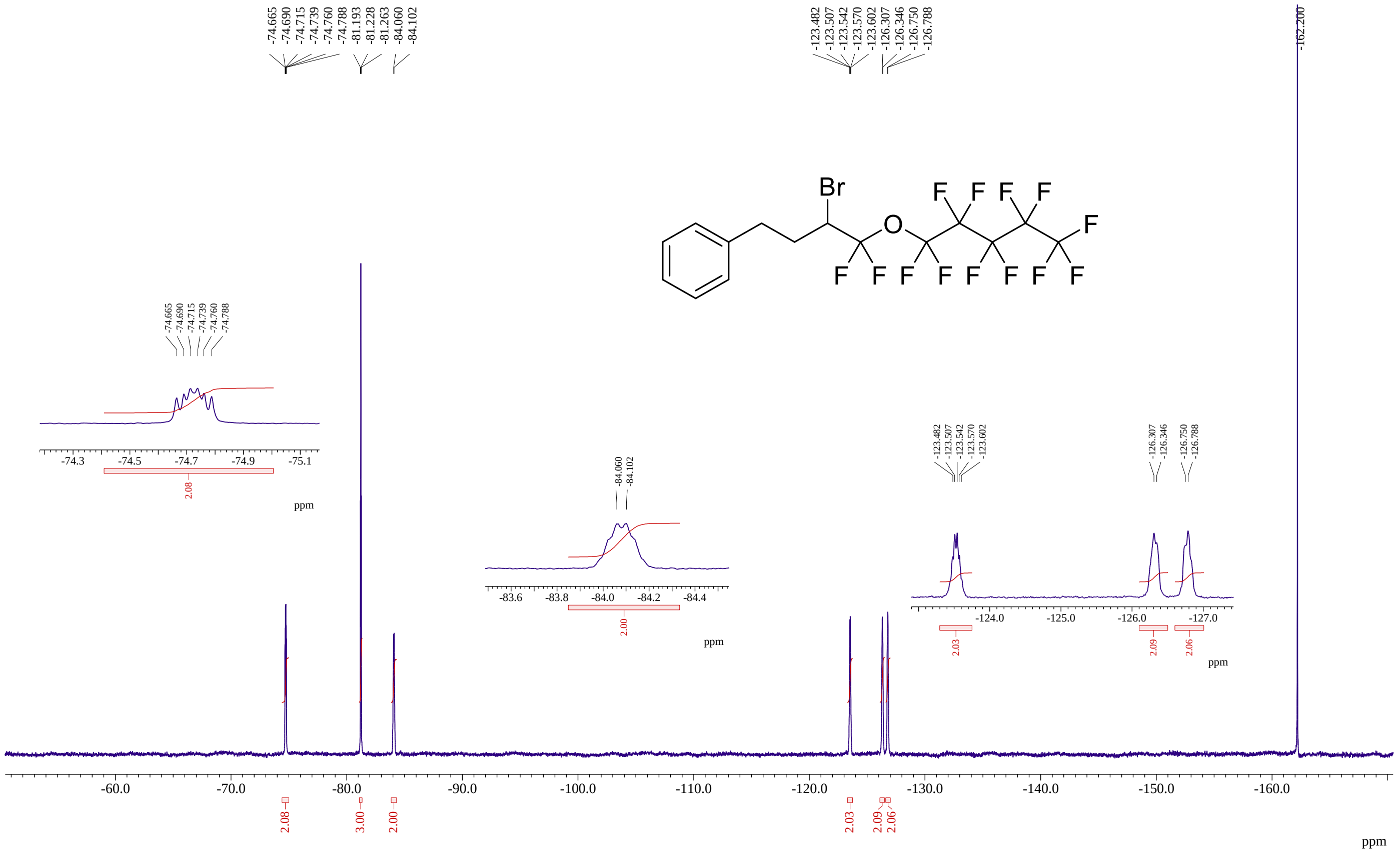

^{1}H NMR (500 MHz, $CDCl_3$): **4ae**

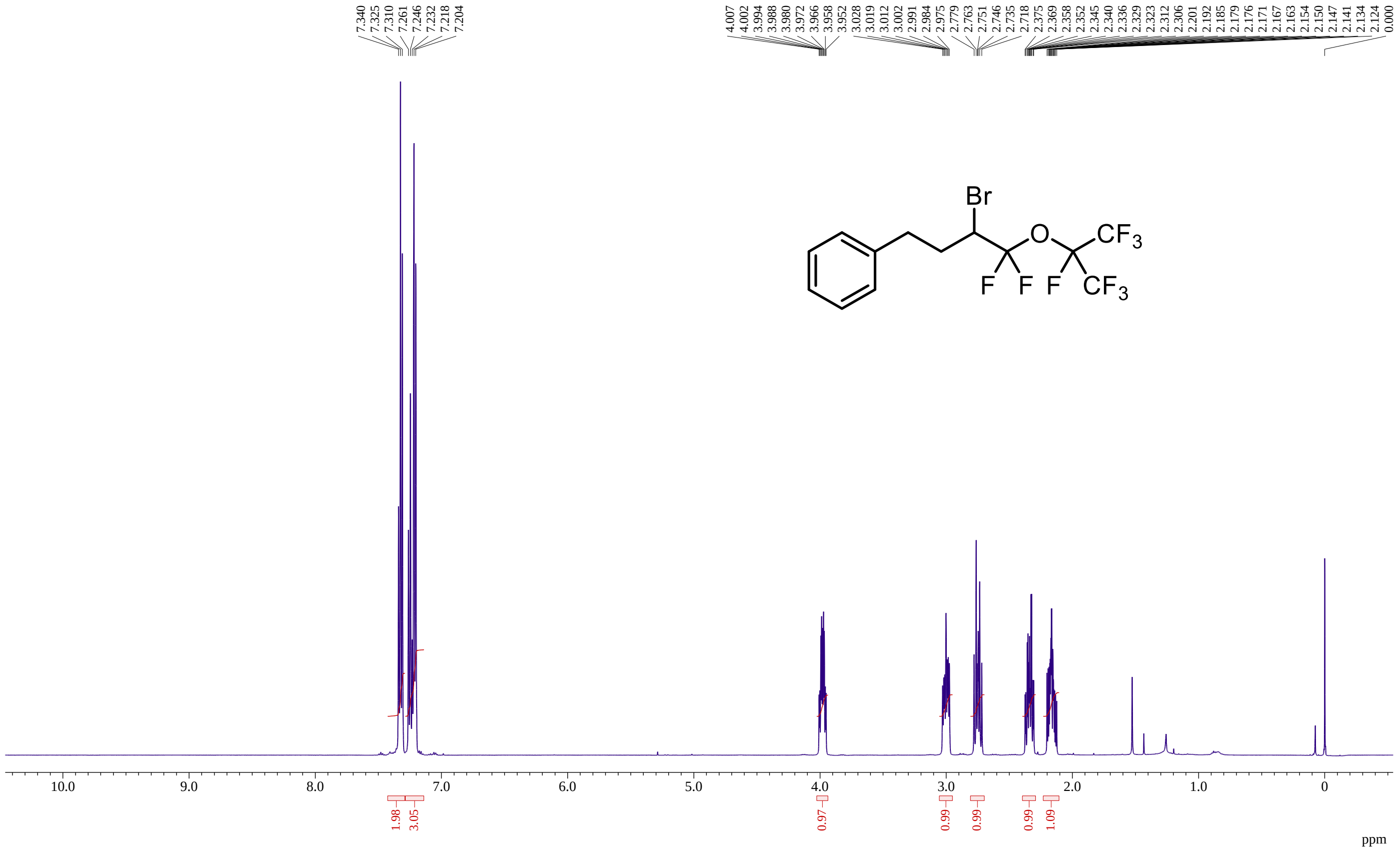

^{13}C NMR (126 MHz, $CDCl_3$): **4ae**

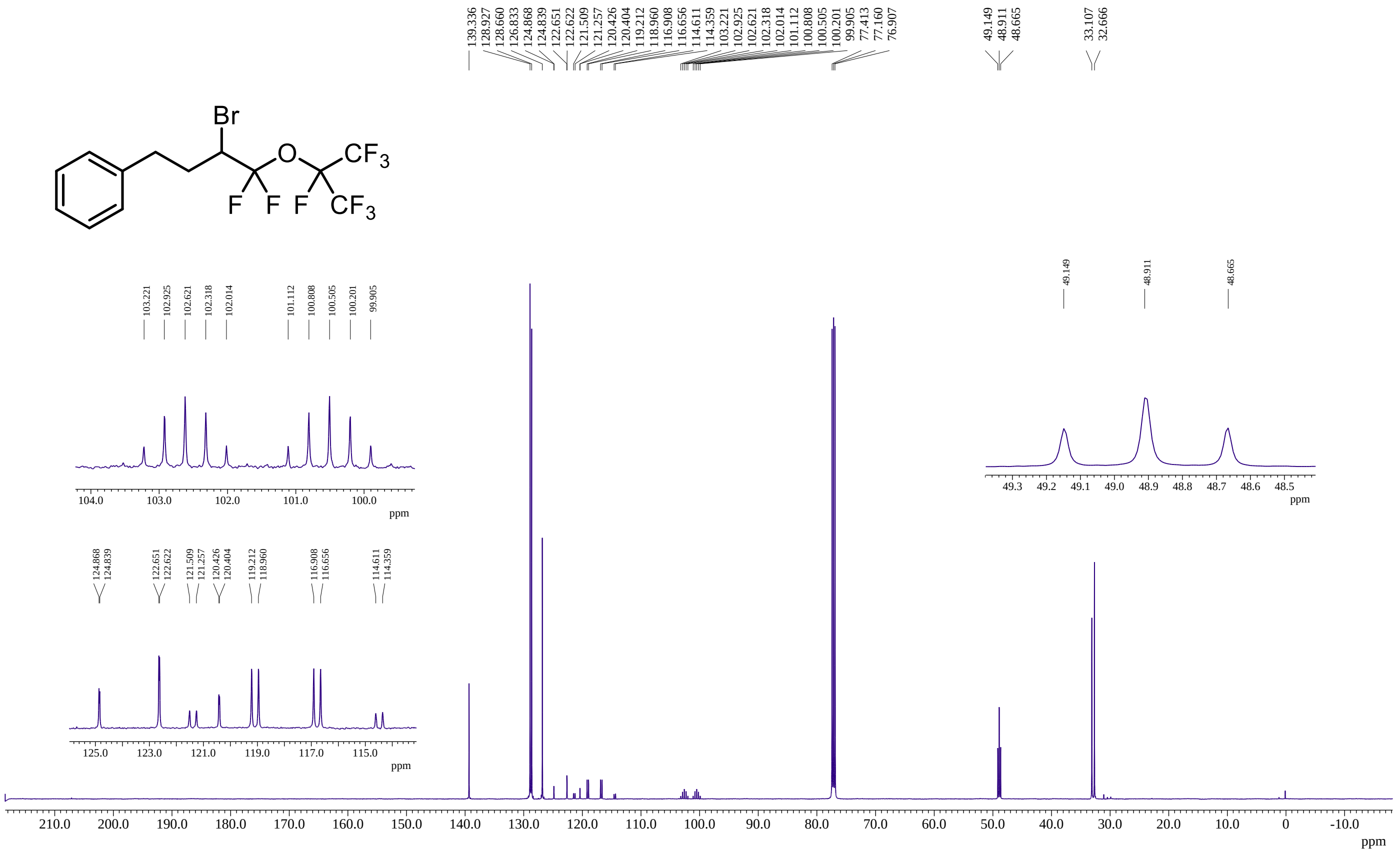

^{19}F NMR (282 MHz, $CDCl_3$): **4ae**

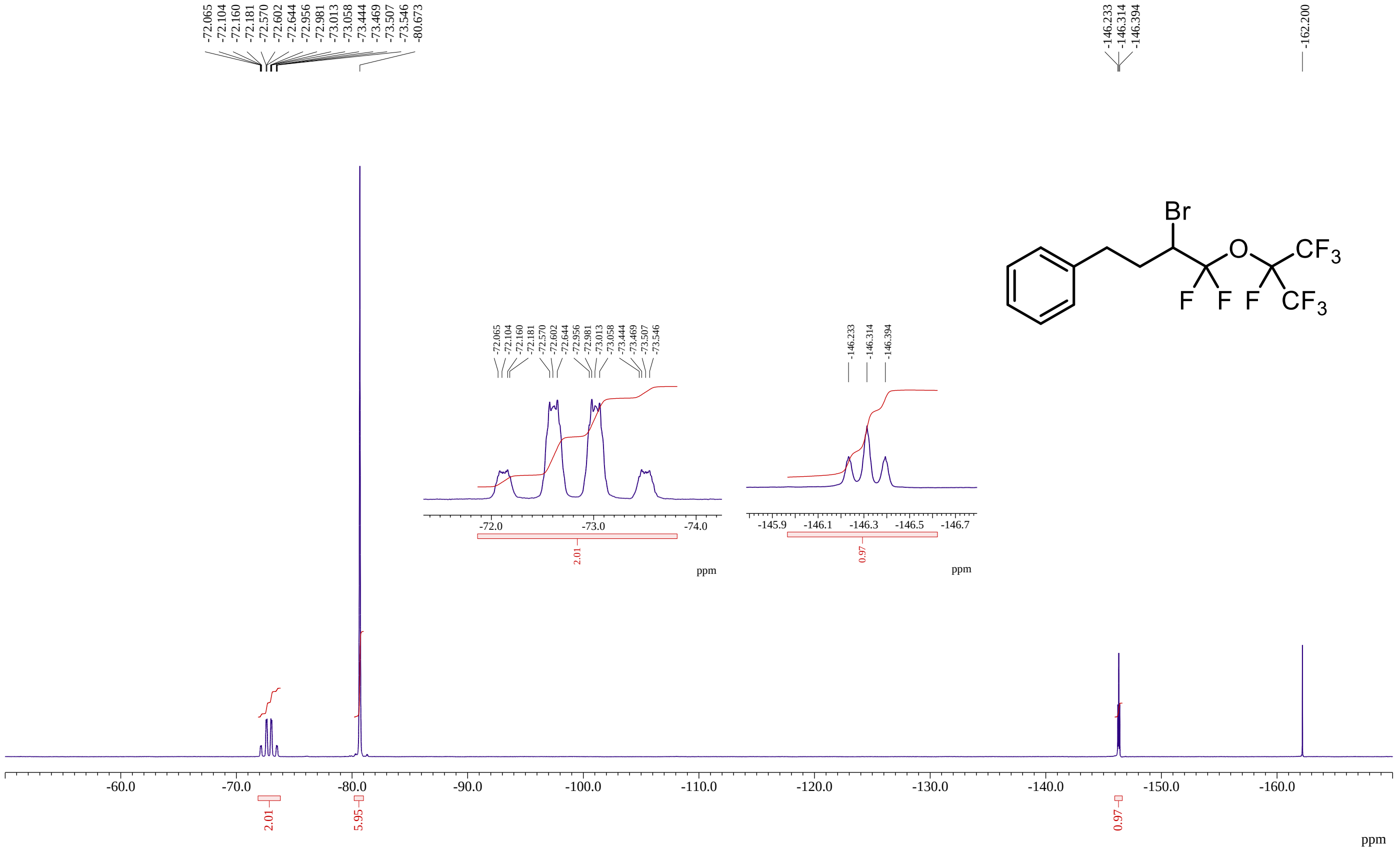

^{1}H NMR (500 MHz, $CDCl_3$): **6aa**

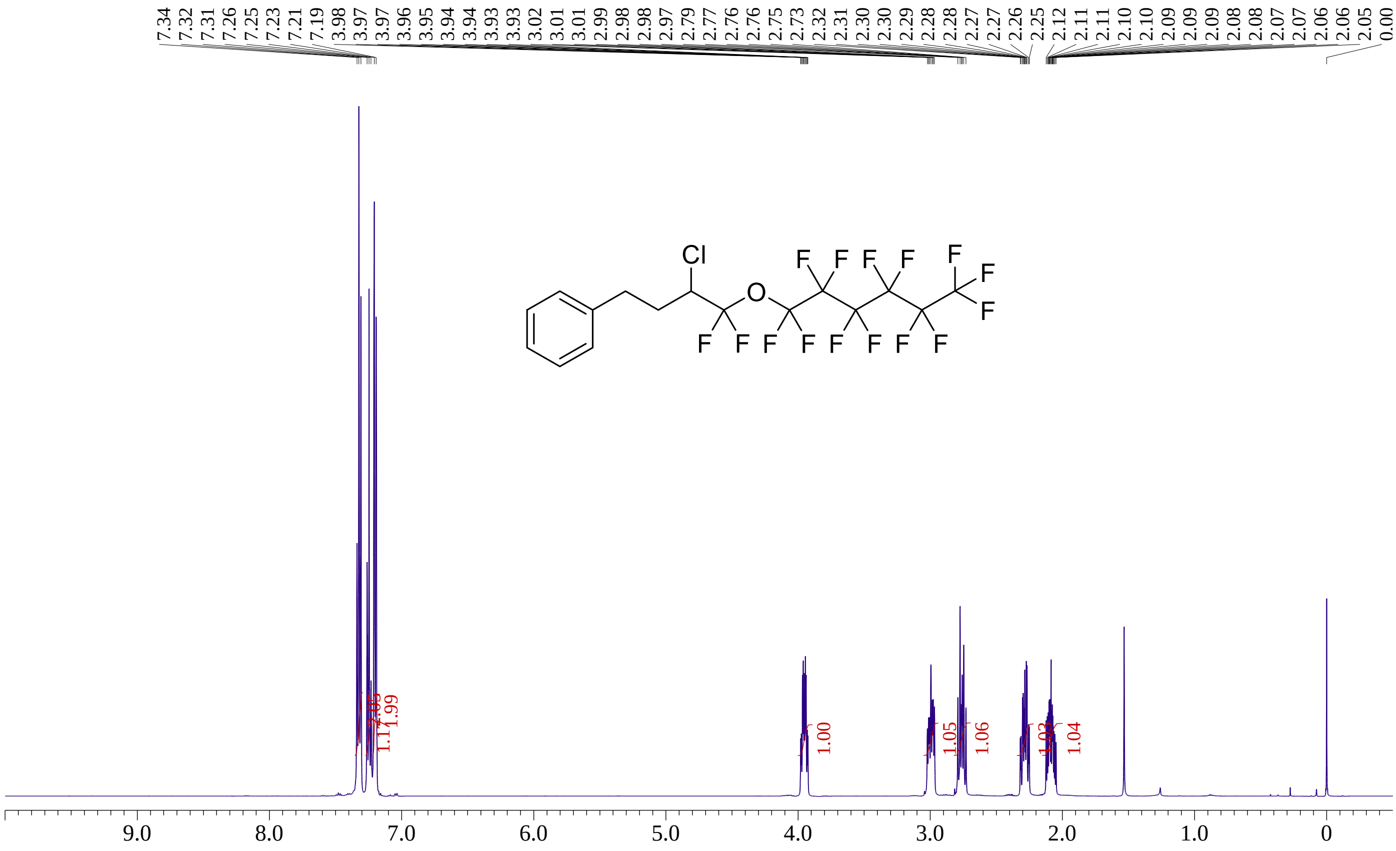

^{13}C NMR (126 MHz, $CDCl_3$): **6aa**

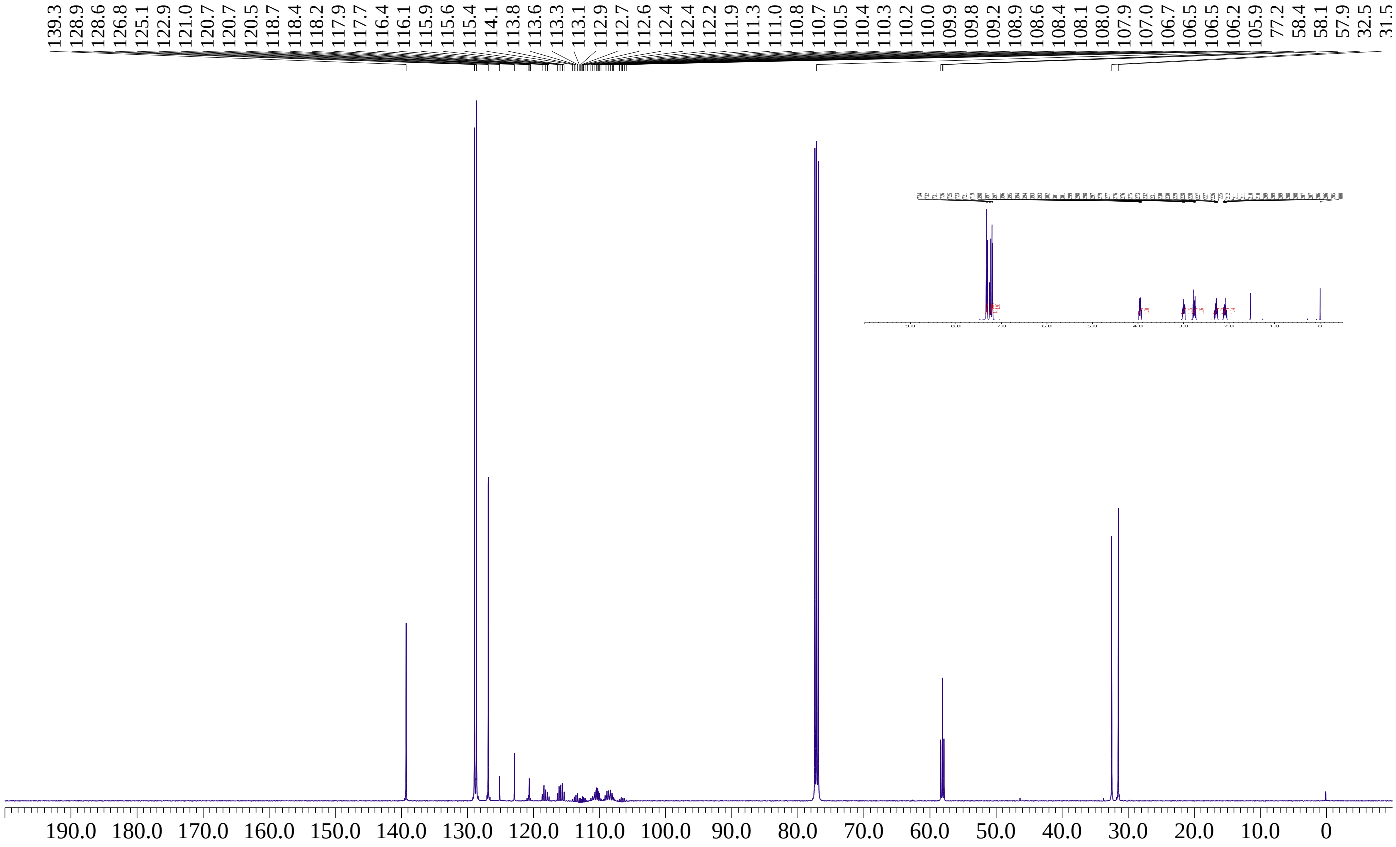

^{19}**F NMR** (282 MHz, $CDCl_3$): **6aa**

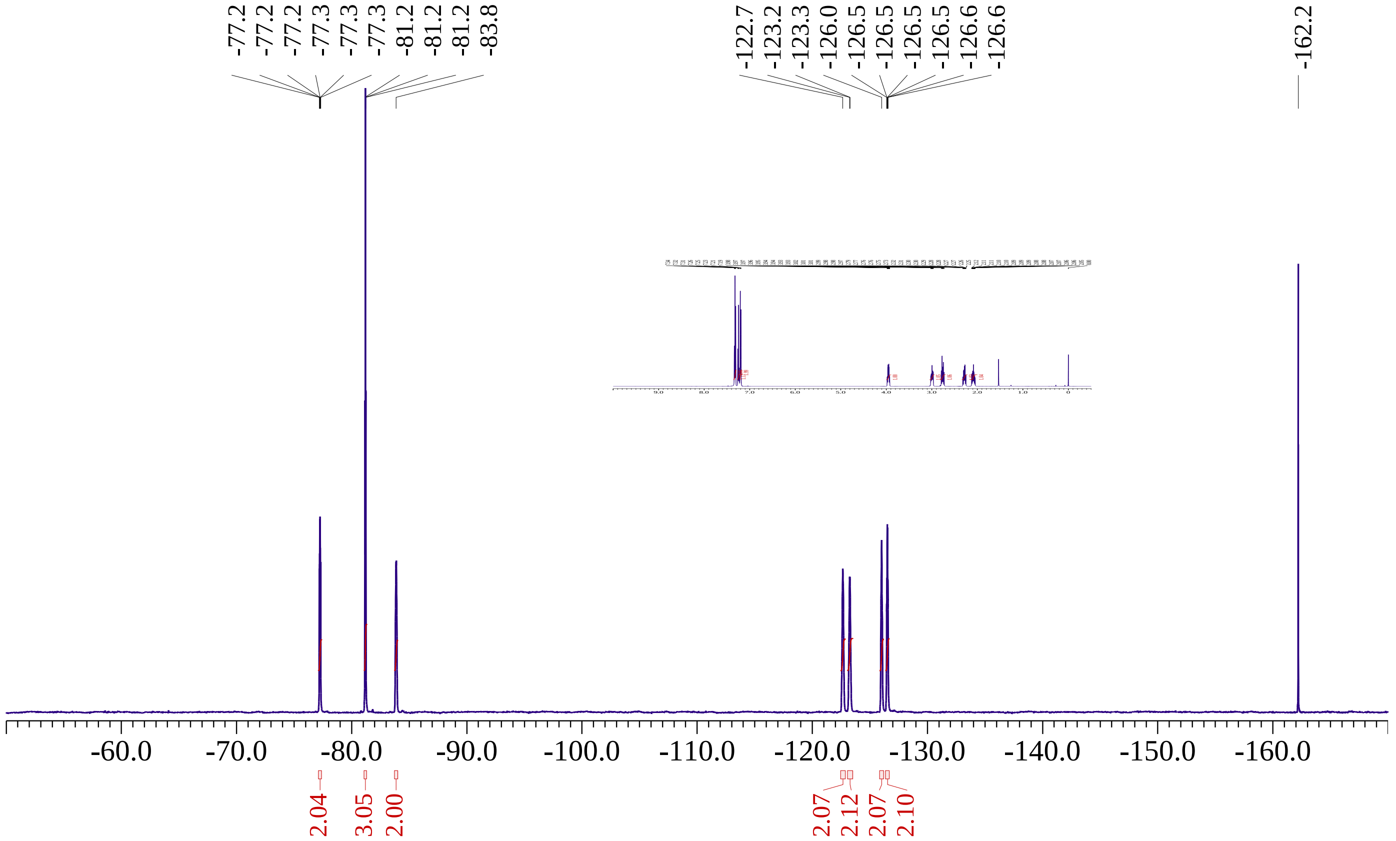

^{1}**H NMR** (500 MHz, $CDCl_3$): **6ba**

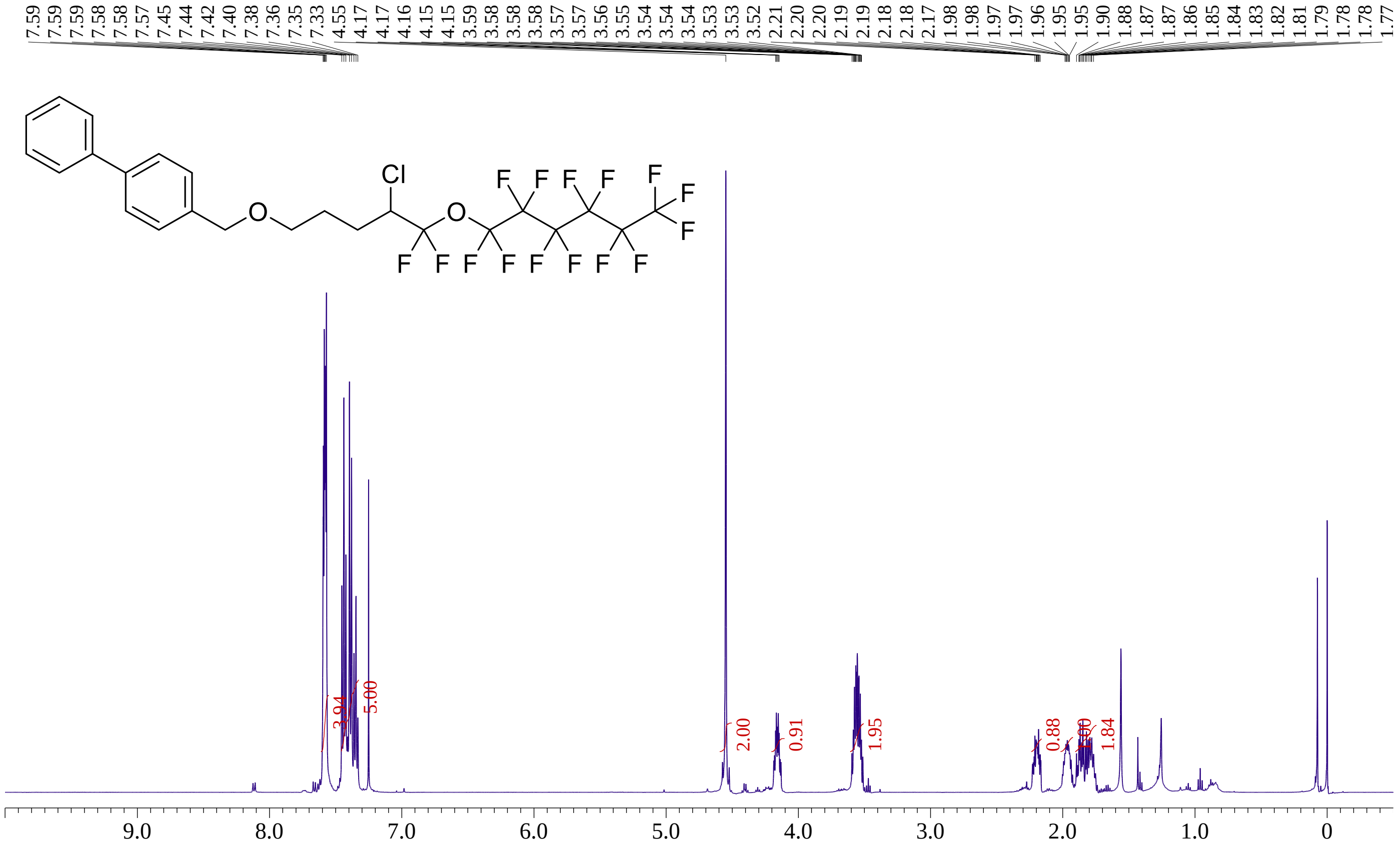

^{13}C NMR (126 MHz, $CDCl_3$): **6ba**

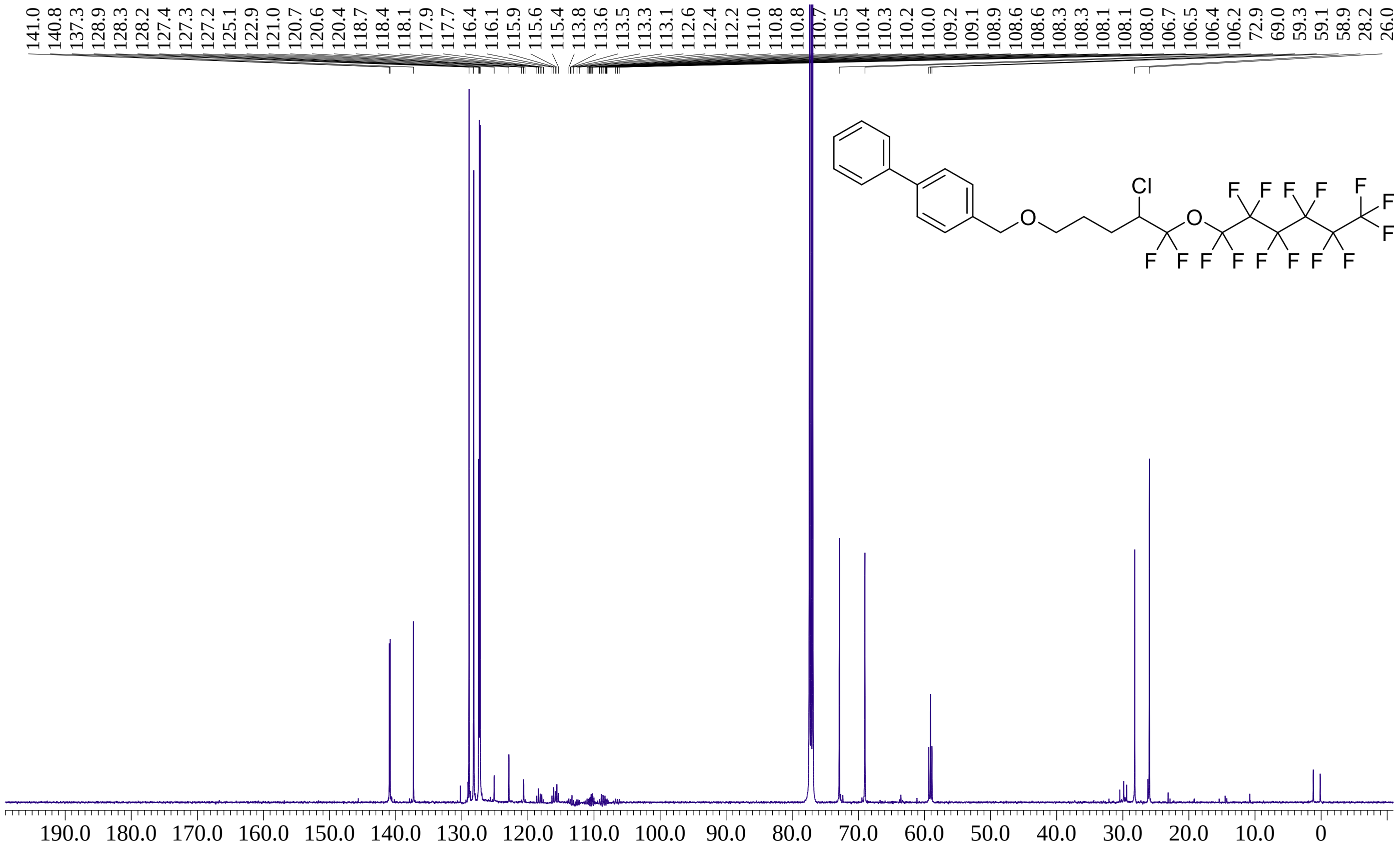

^{19}F NMR (282 MHz, $CDCl_3$): **6ba**

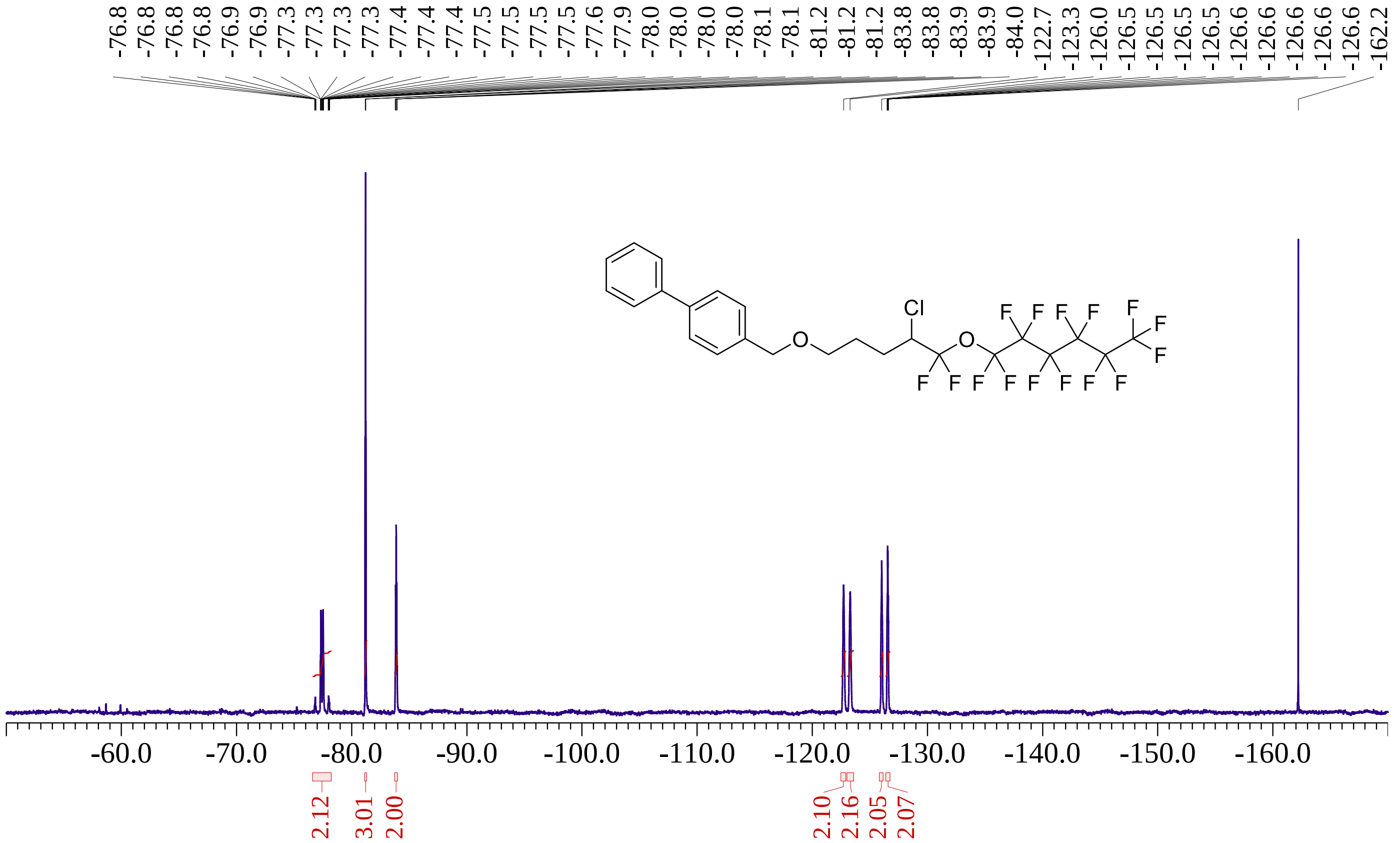

^{1}H NMR (500 MHz, $CDCl_3$): **6da**

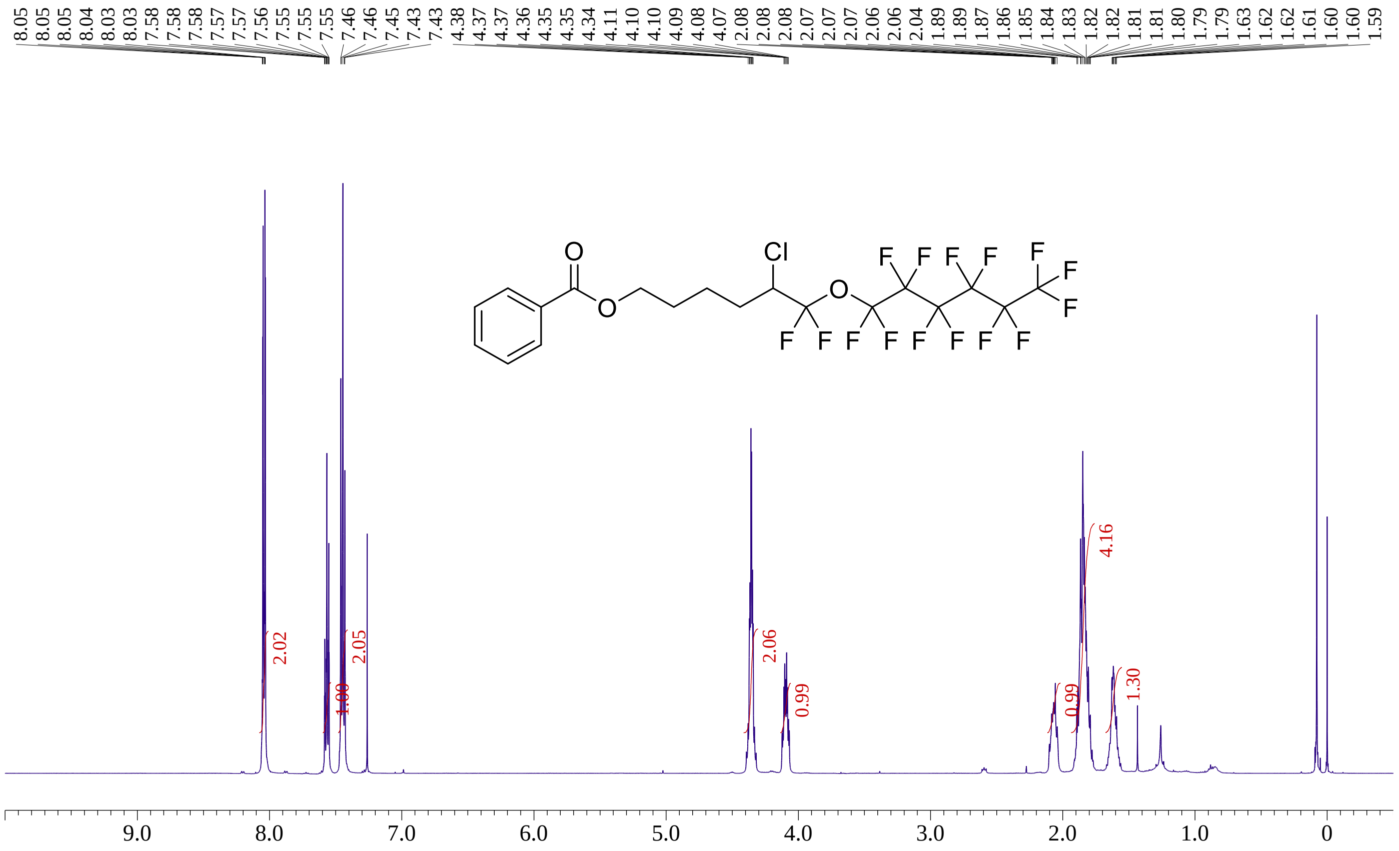

^{13}C NMR (126 MHz, $CDCl_3$): **6da**

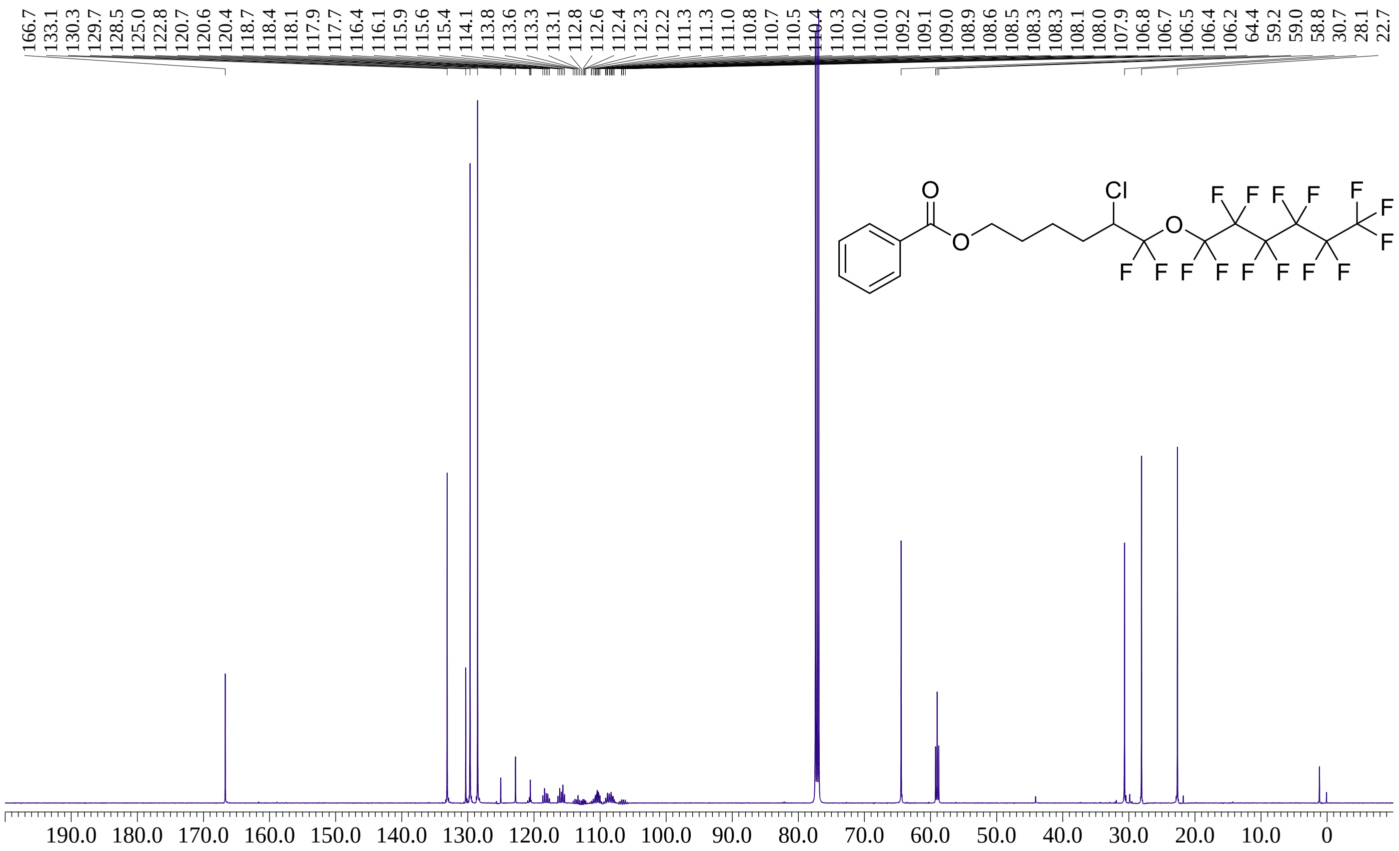

[19]**F NMR** (282 MHz, $CDCl_3$): **6da**

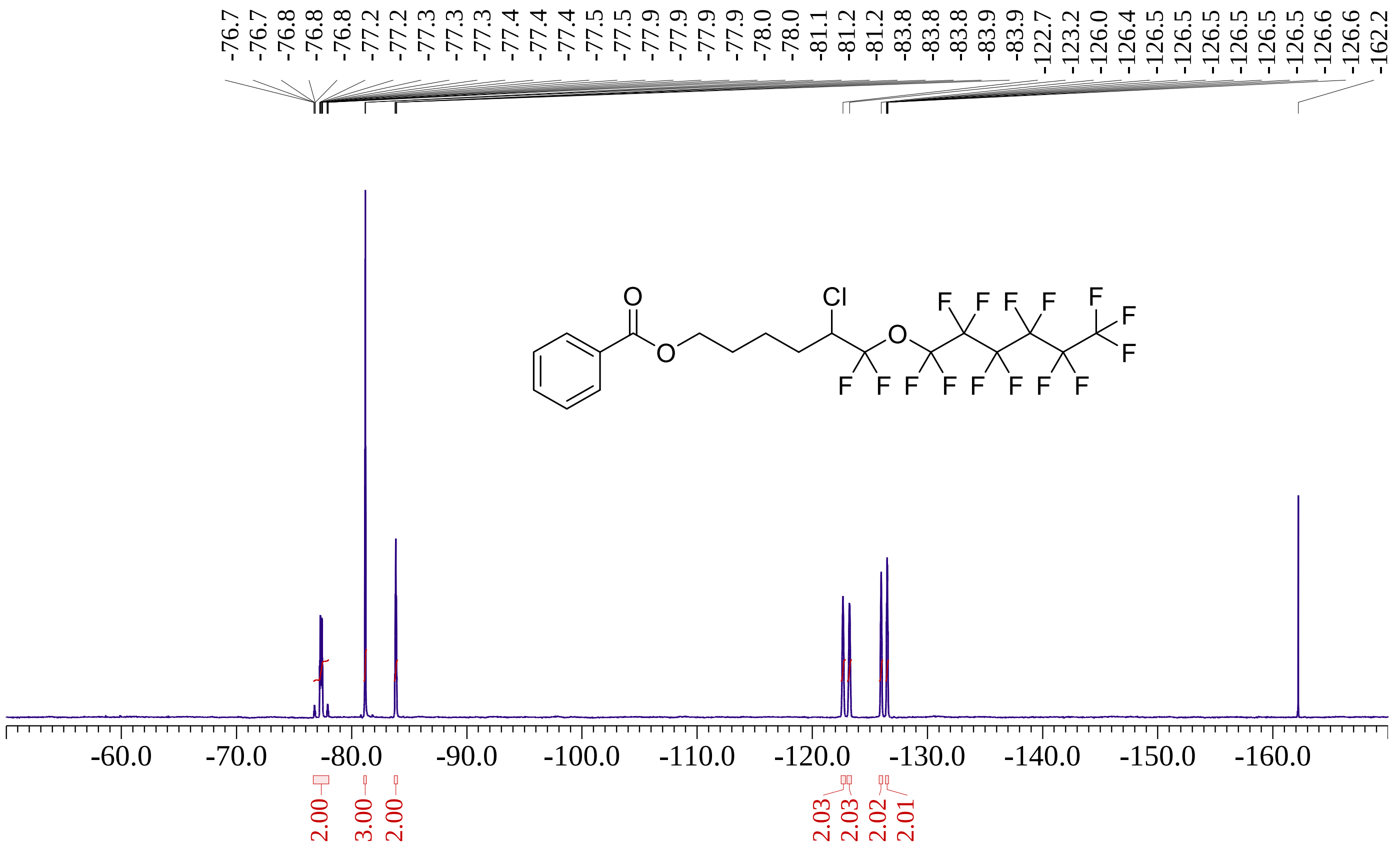

[1]**H NMR** (500 MHz, $CDCl_3$): **6ea**

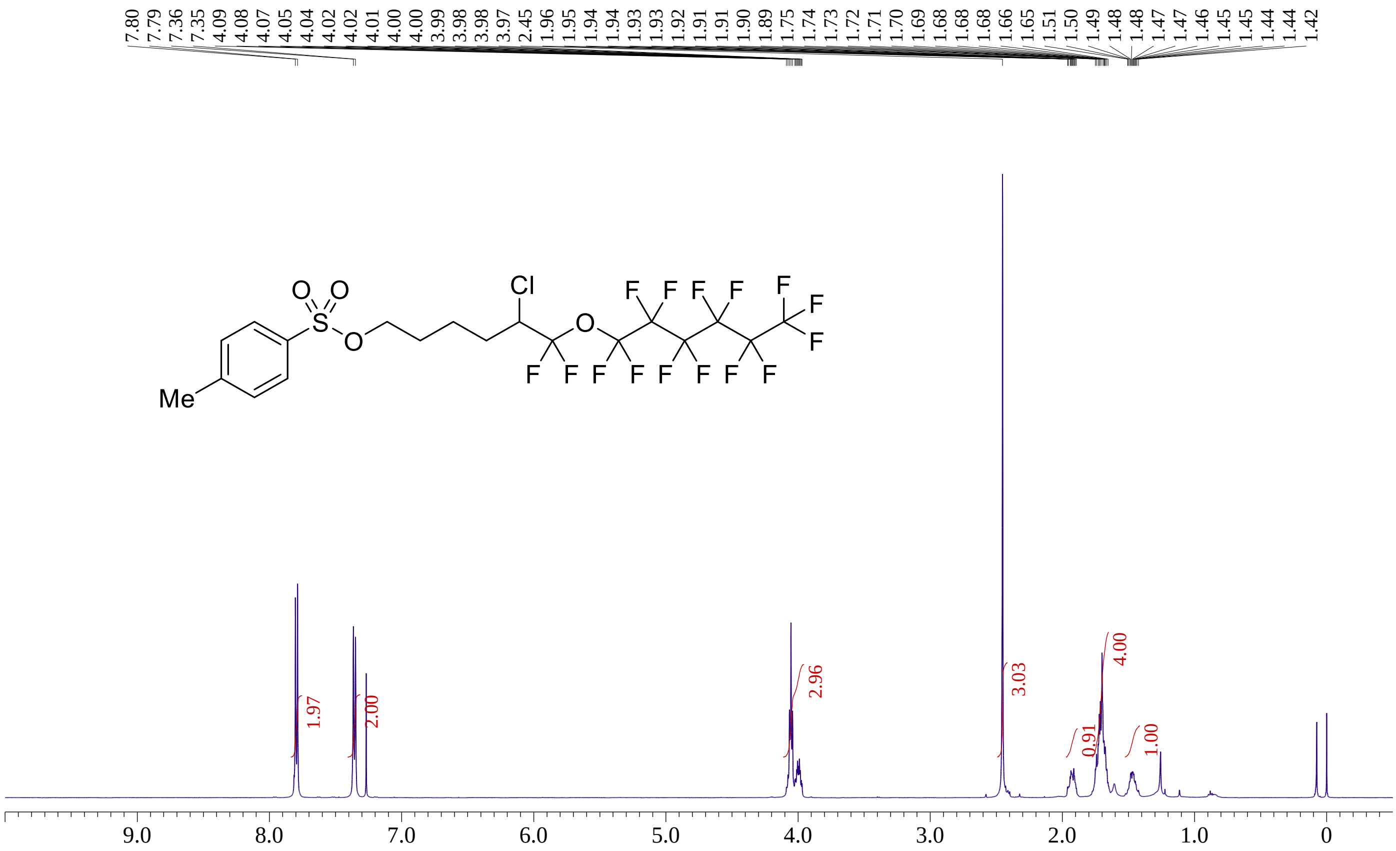

^{13}C NMR (126 MHz, $CDCl_3$): **6ea**

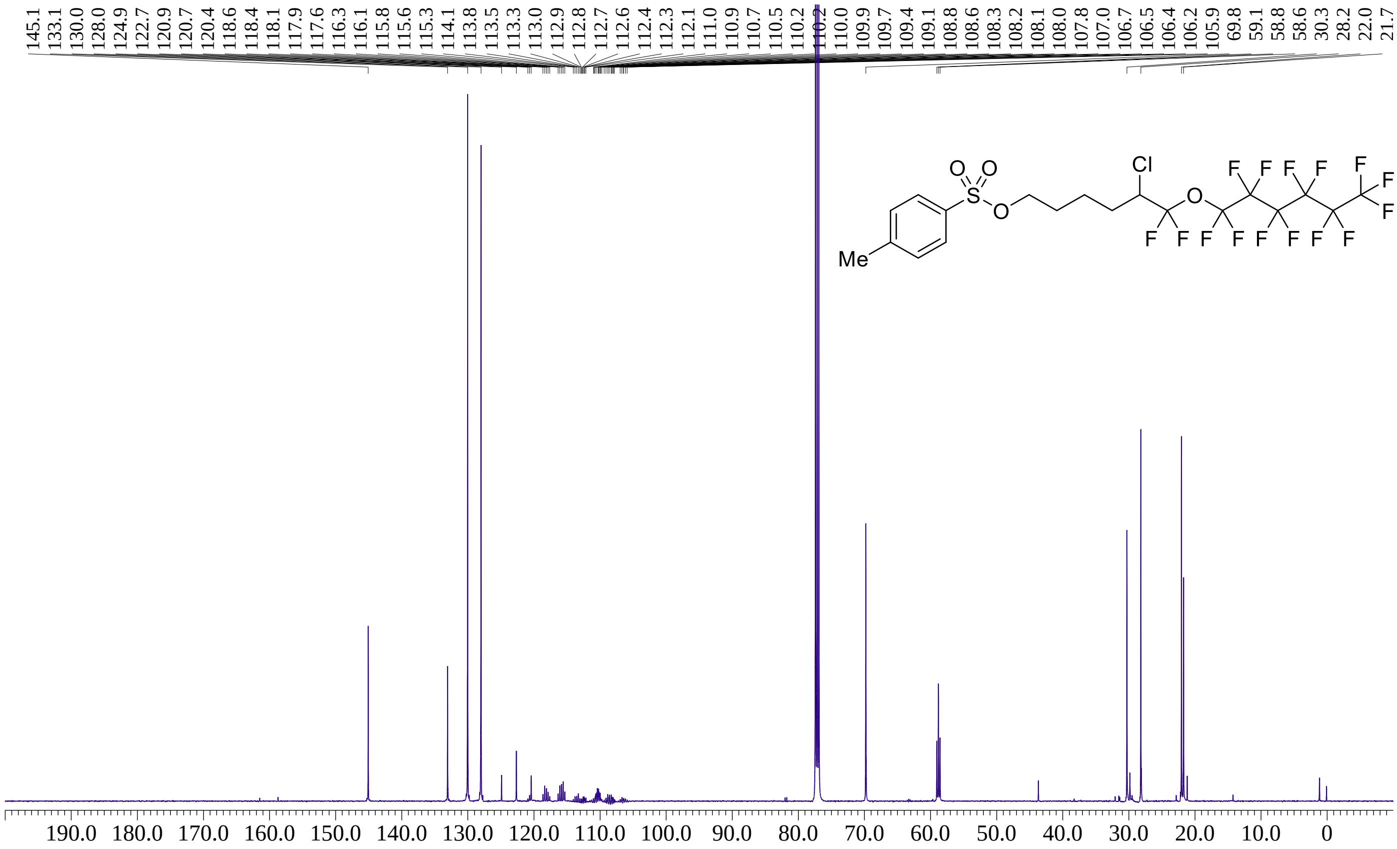

^{19}F NMR (282 MHz, $CDCl_3$): **6ea**

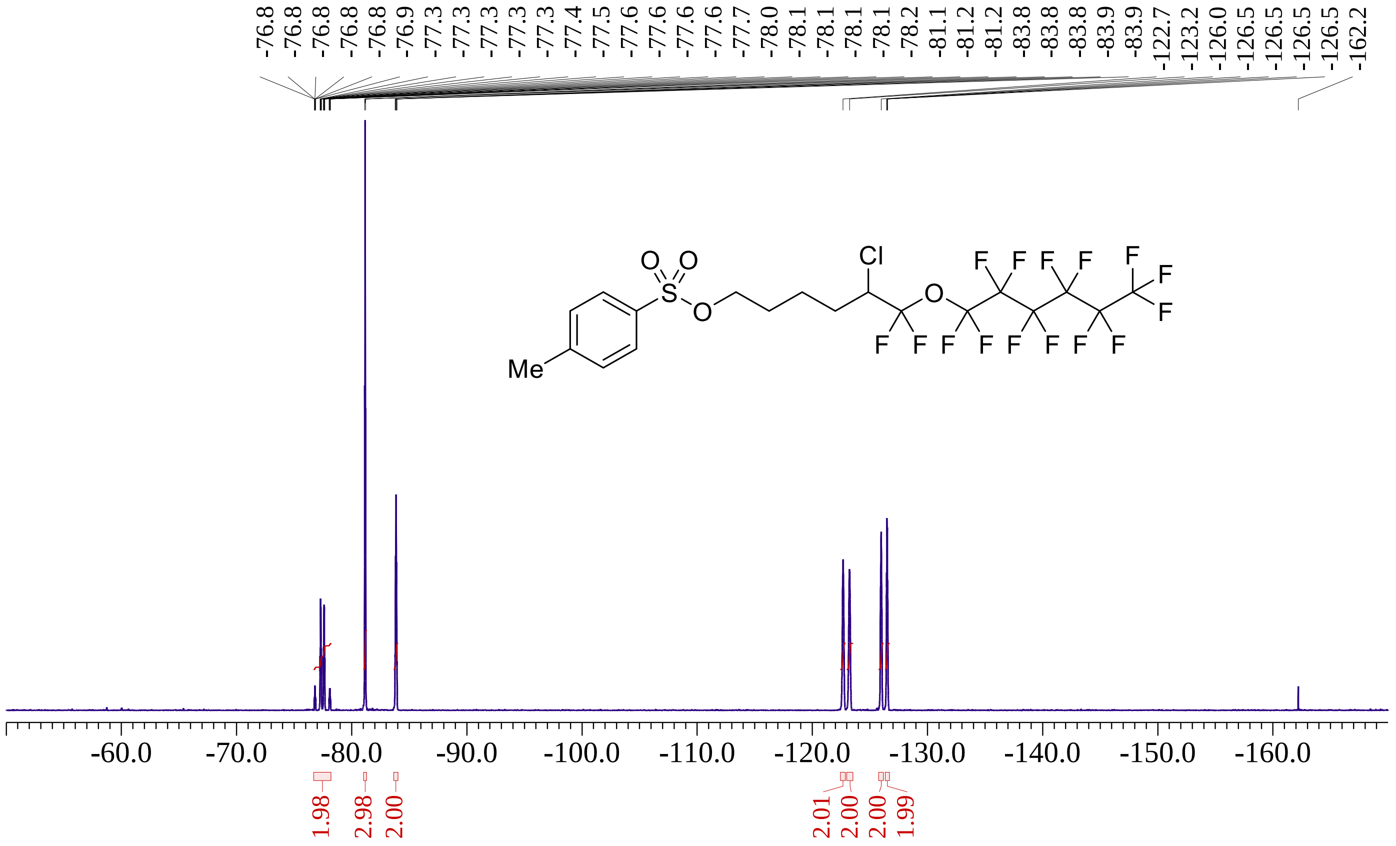

[1]**H NMR** (500 MHz, CDCl$_3$): **6ta**

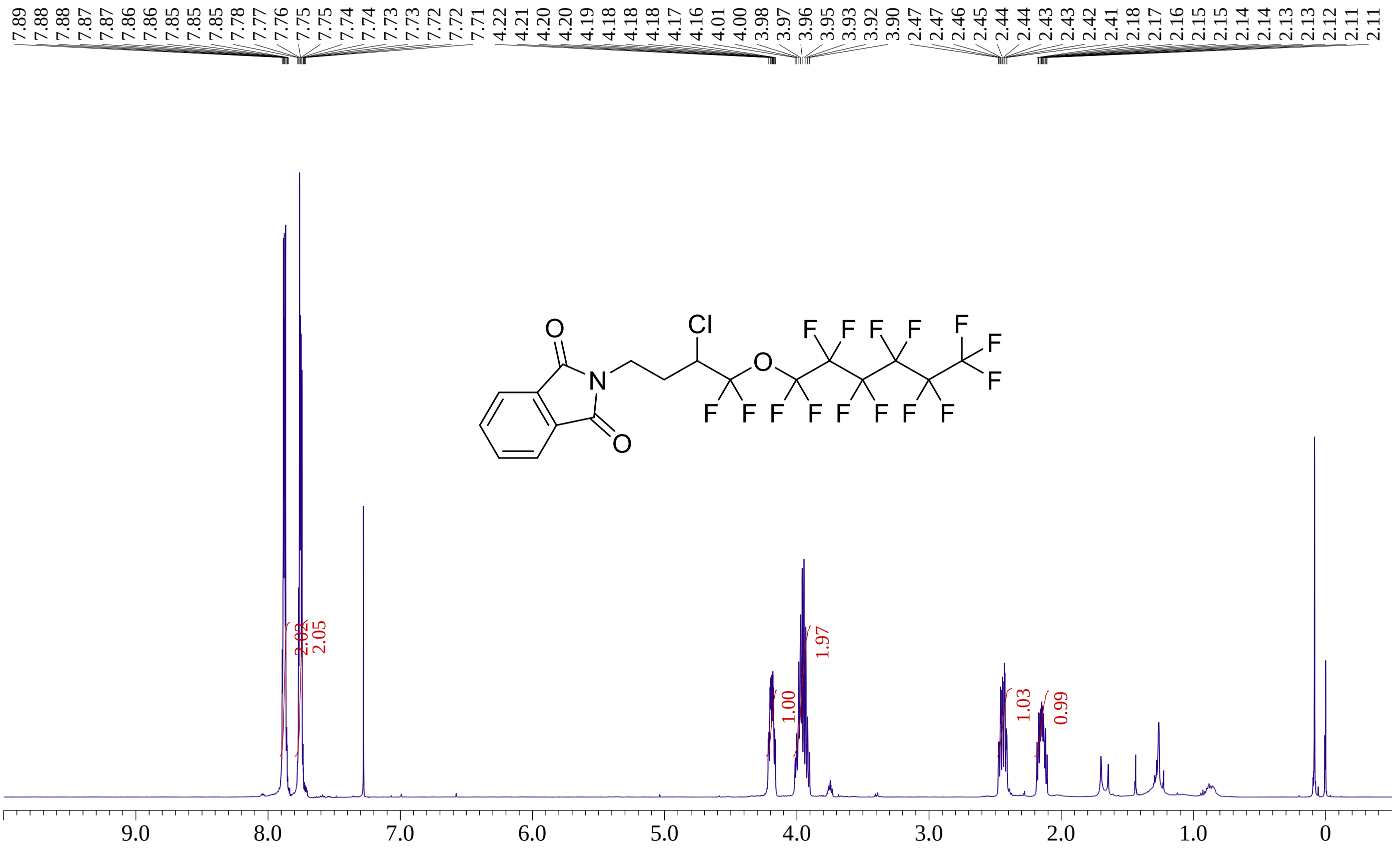

[13]**C NMR** (126 MHz, CDCl$_3$): **6ta**

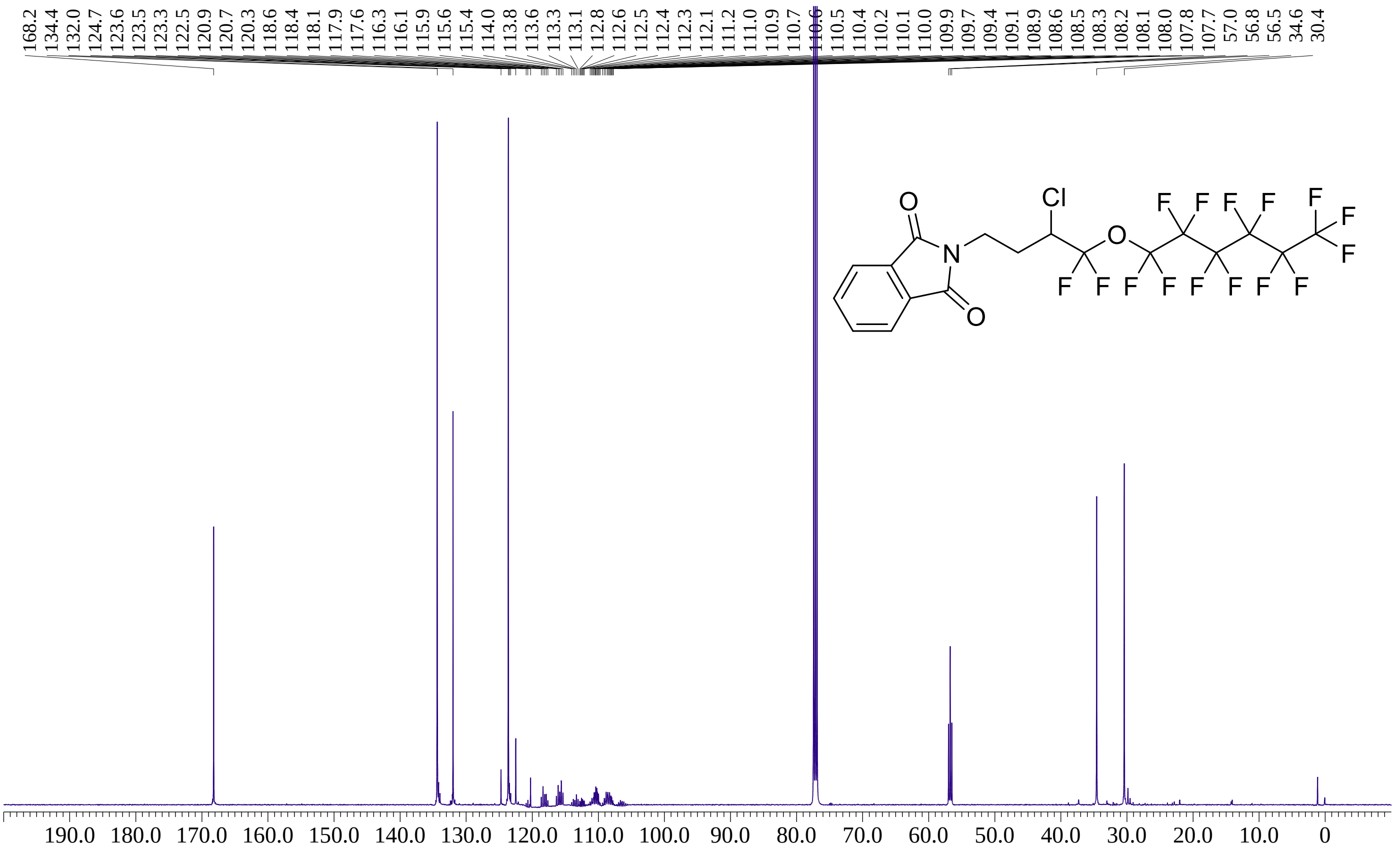

^{19}**F NMR** (282 MHz, $CDCl_3$): **6ta**

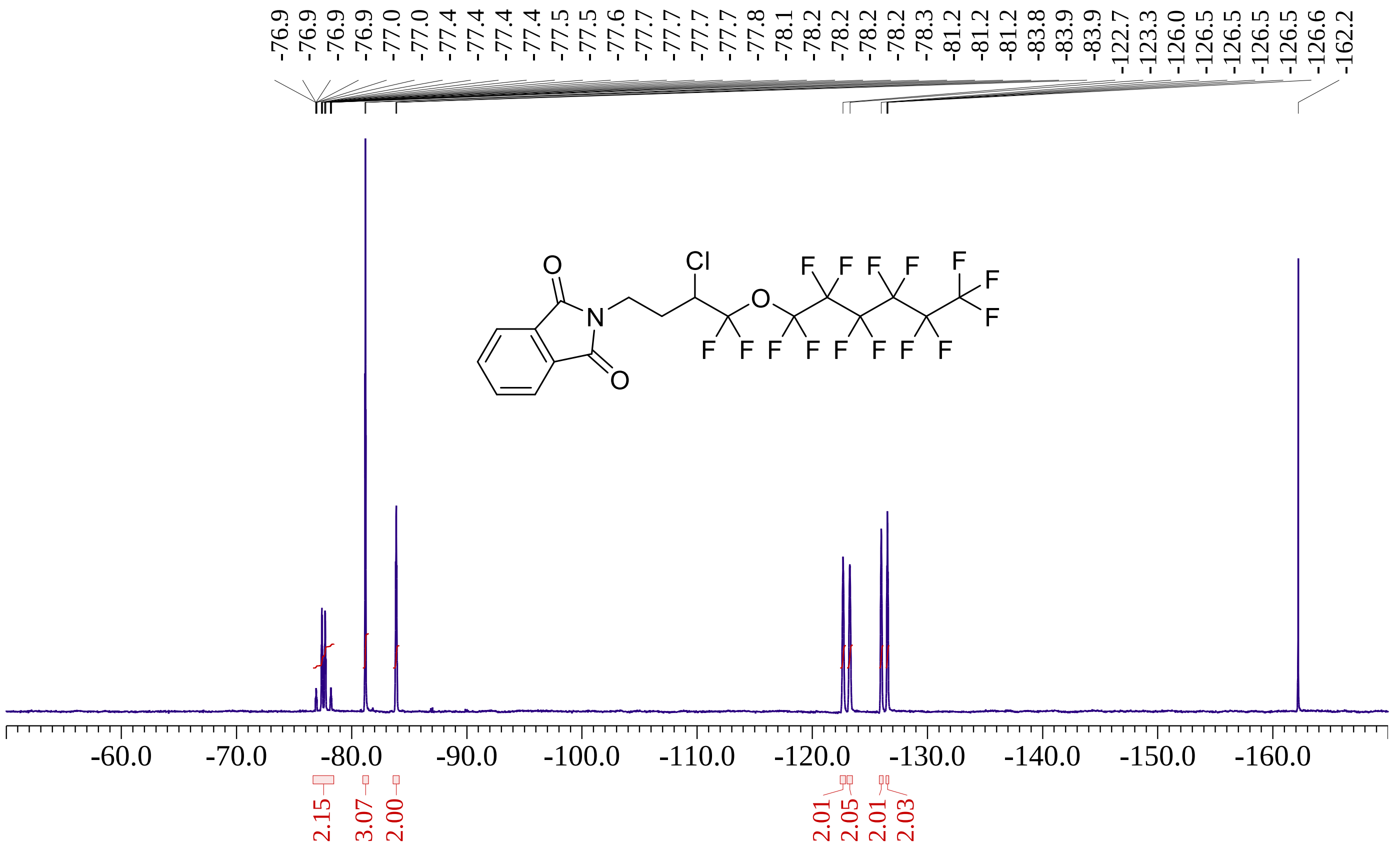

^{1}**H NMR** (500 MHz, $CDCl_3$): **7**

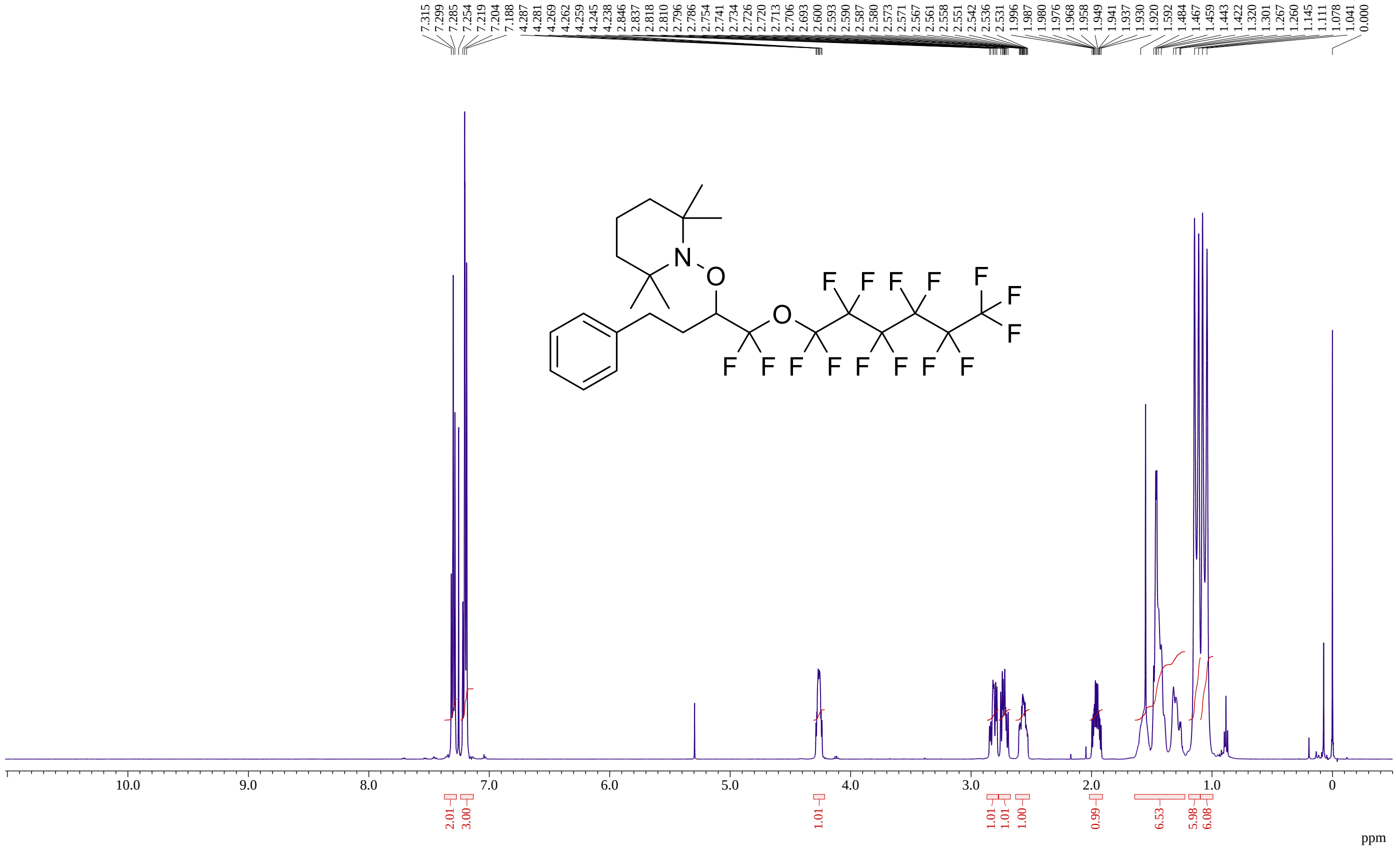

^{13}C NMR (126 MHz, $CDCl_3$): **7**

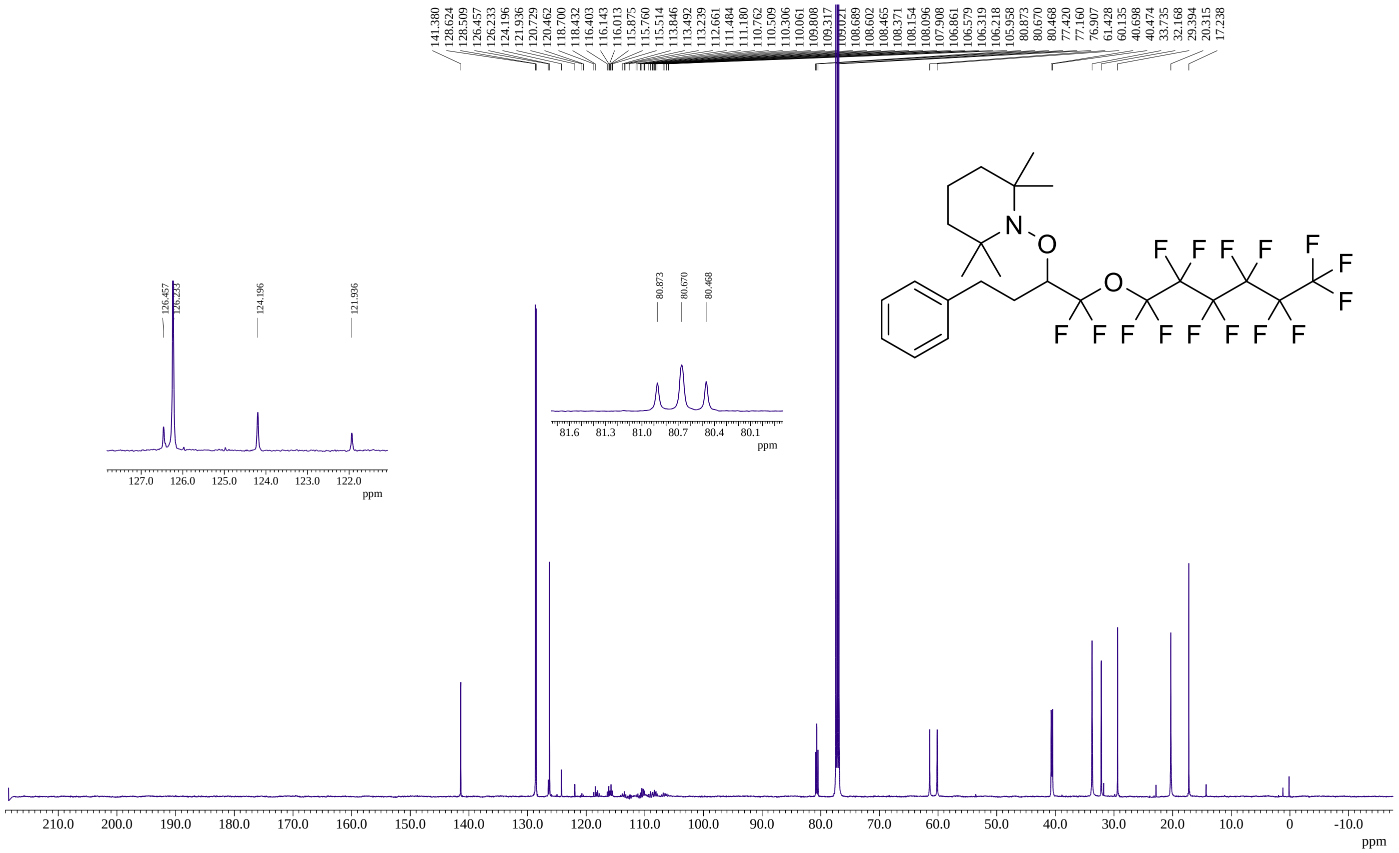

^{19}F NMR (282 MHz, $CDCl_3$): **7**

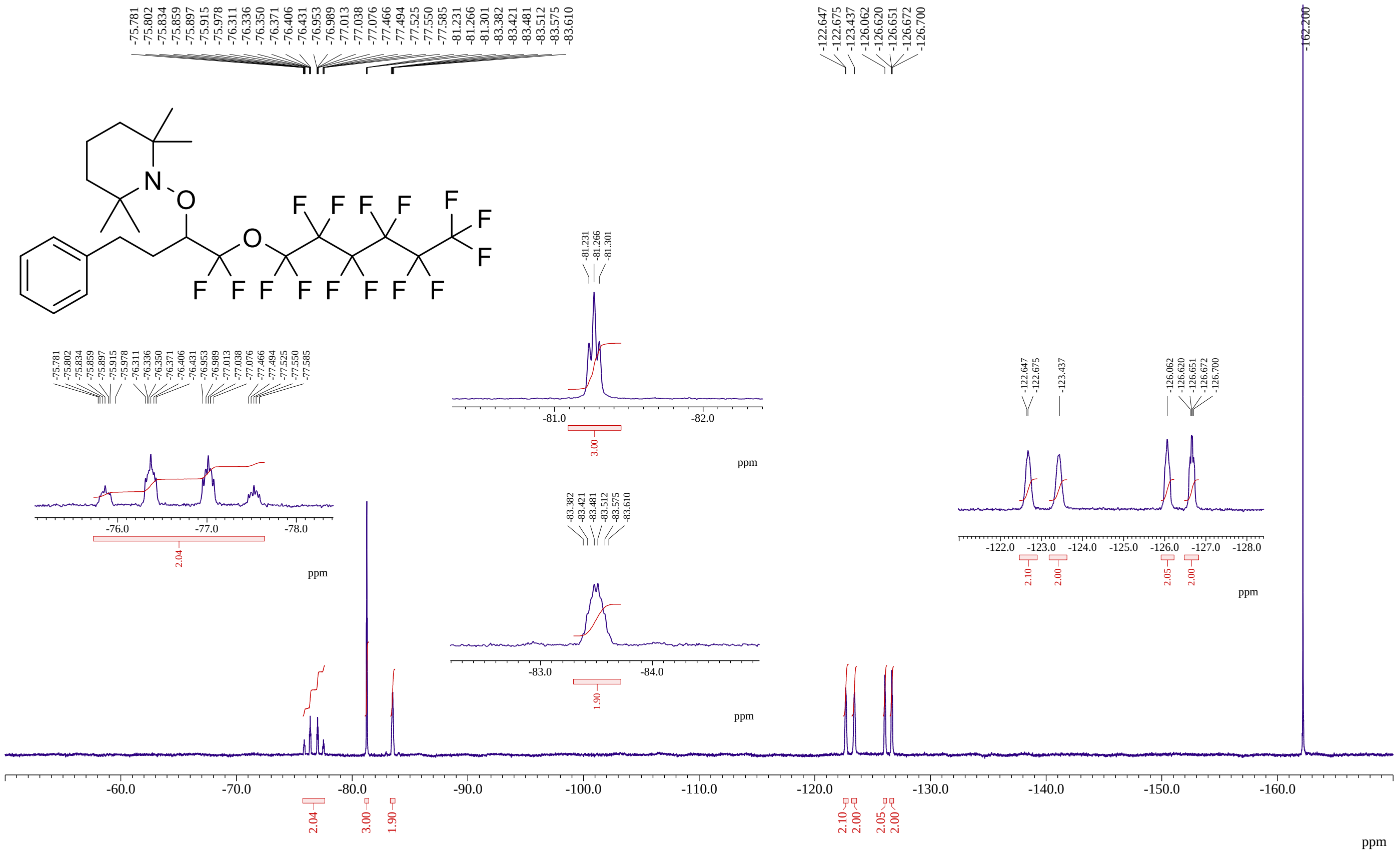

^{1}H NMR (500 MHz, $CDCl_3$): **8**

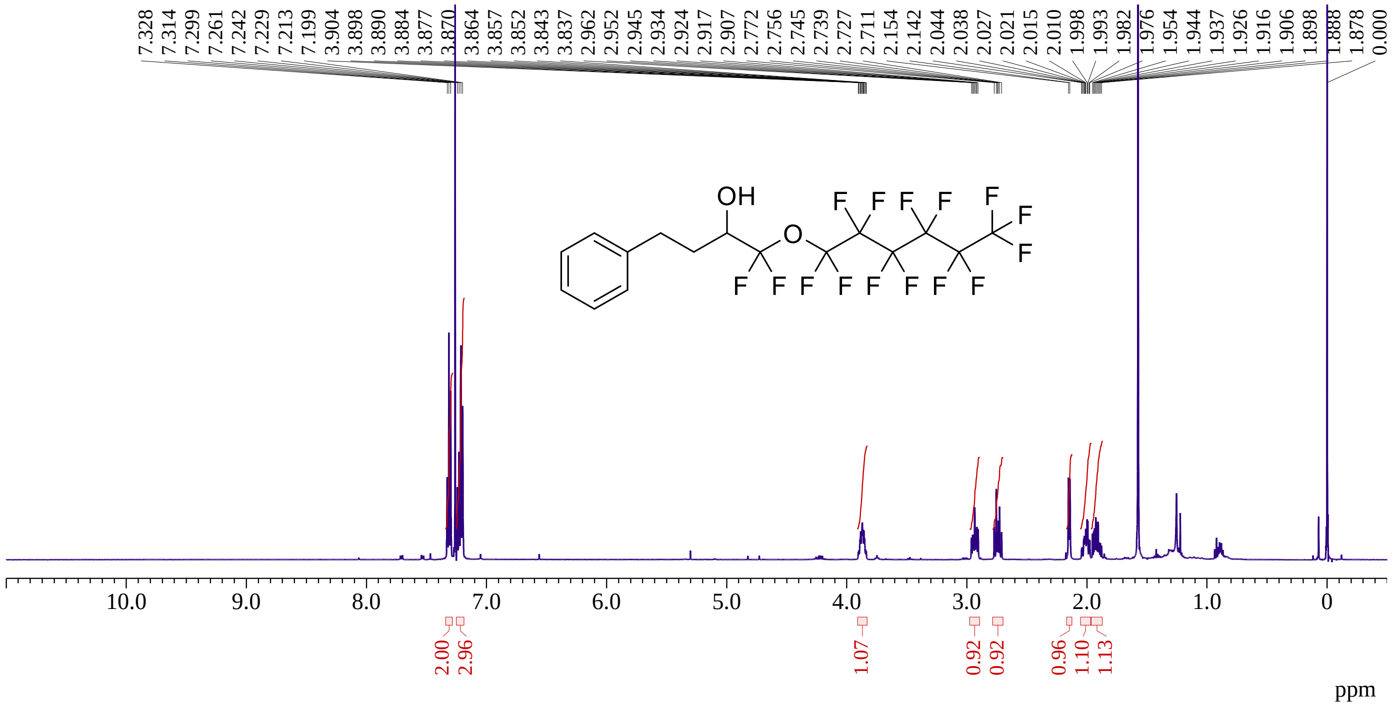

^{13}C NMR (126 MHz, $CDCl_3$): **8**

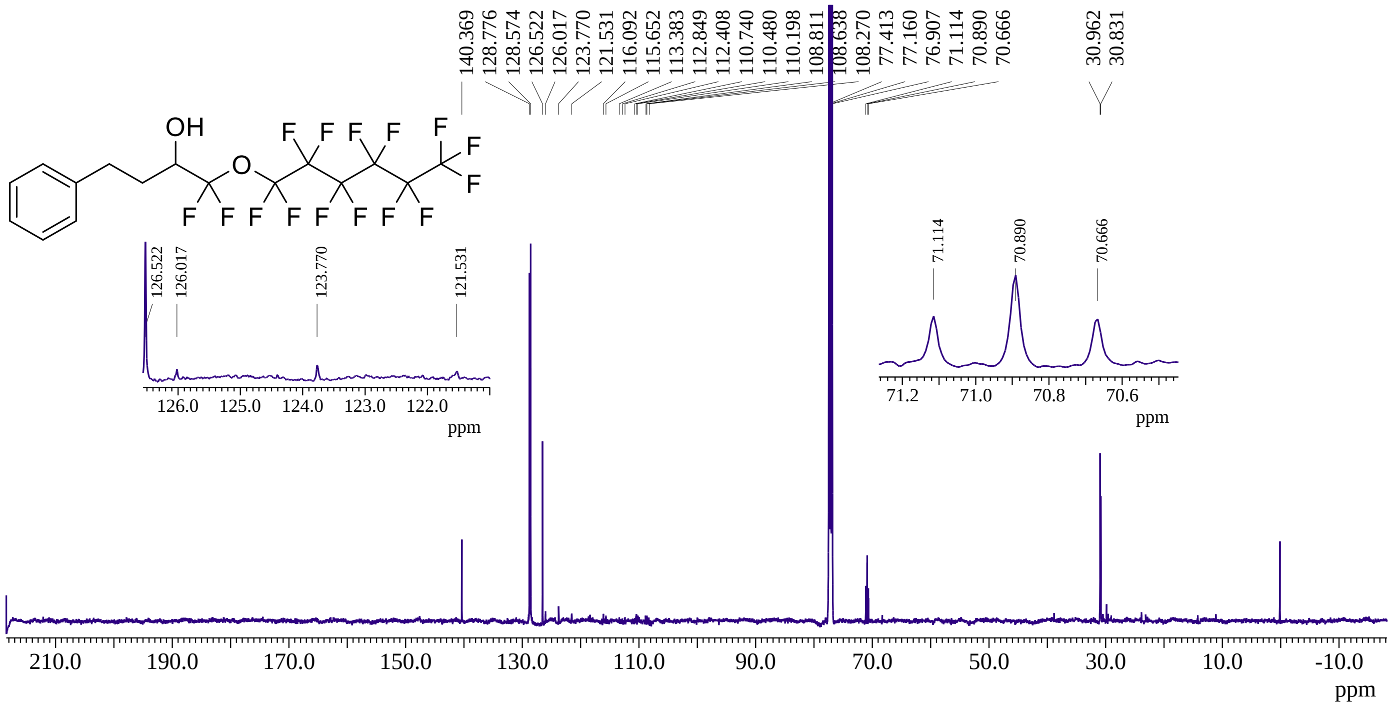

^{19}**F NMR** (282 MHz, $CDCl_3$): **8**

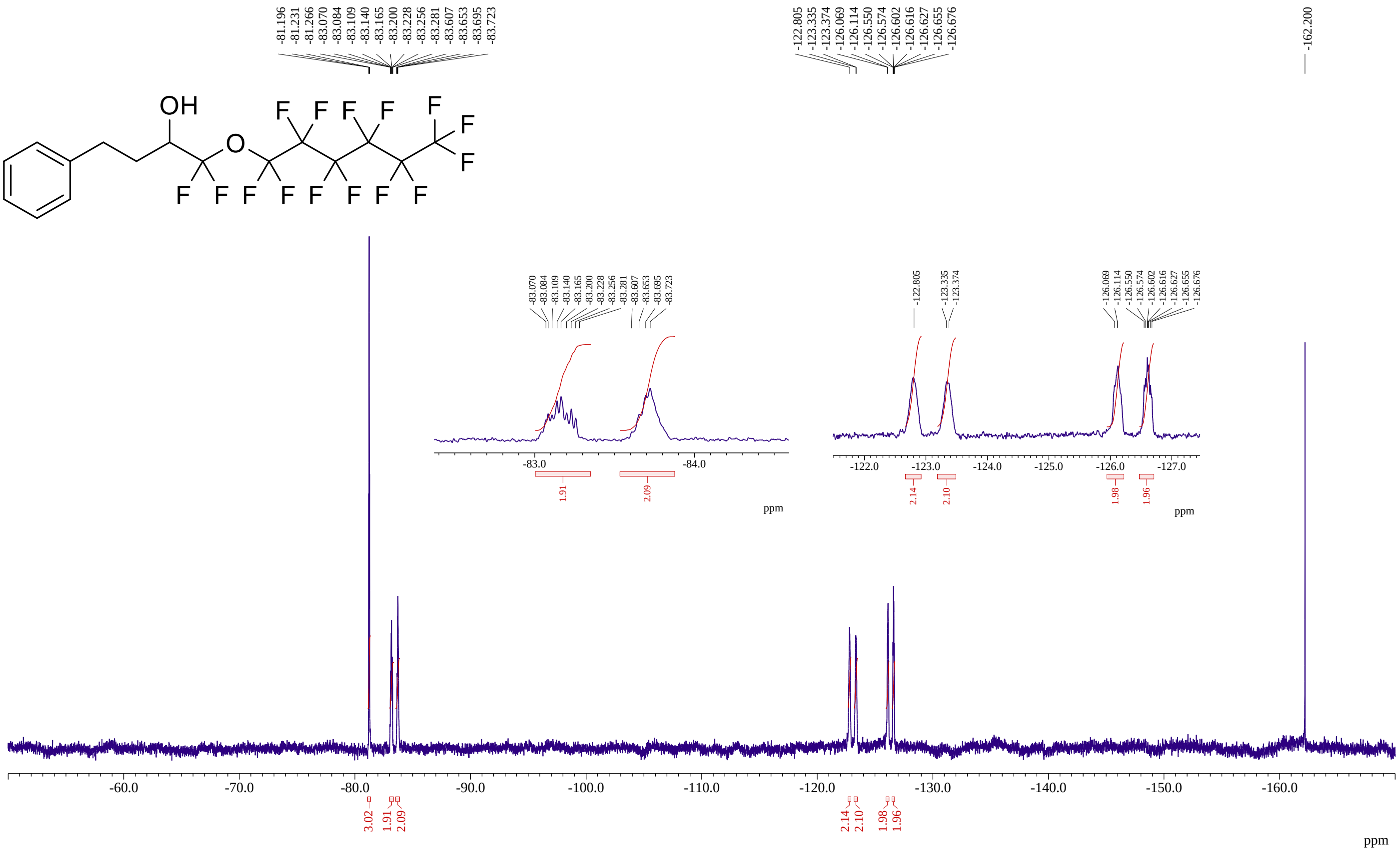

^{1}**H NMR** (500 MHz, $CDCl_3$): **9**

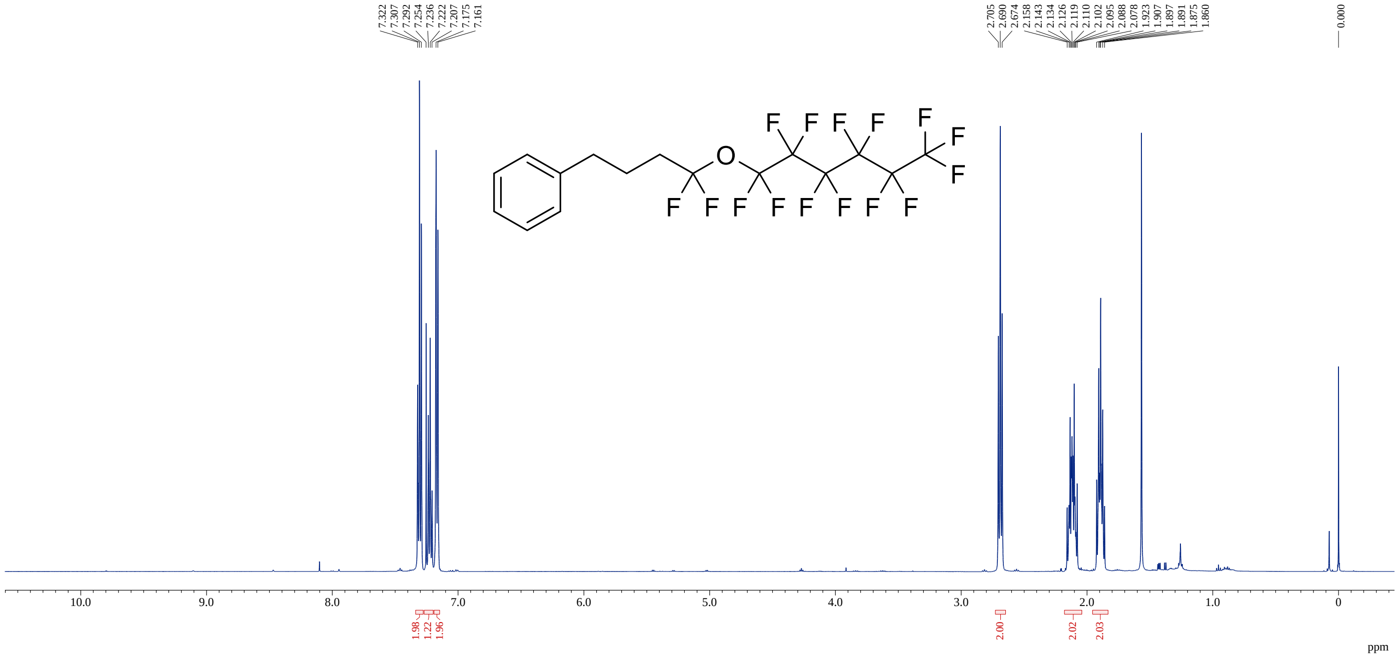

^{13}C NMR (126 MHz, $CDCl_3$): **9**

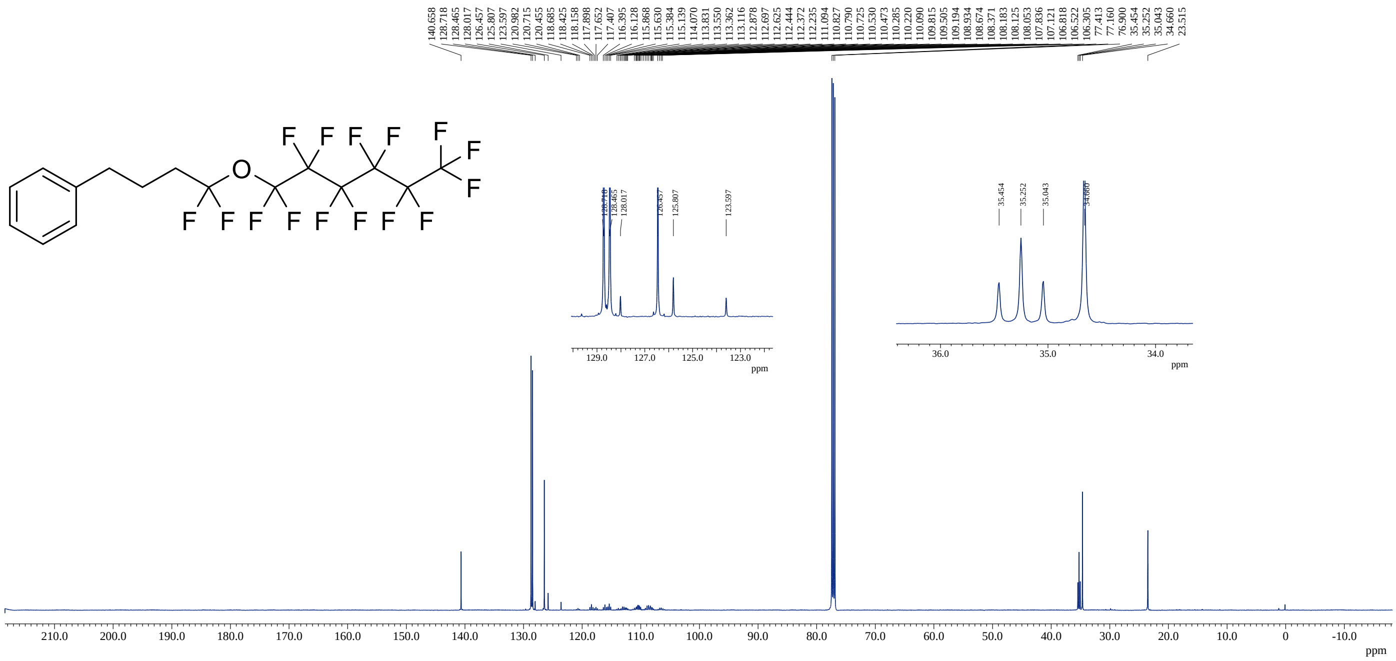

^{19}F NMR (282 MHz, $CDCl_3$): **9**

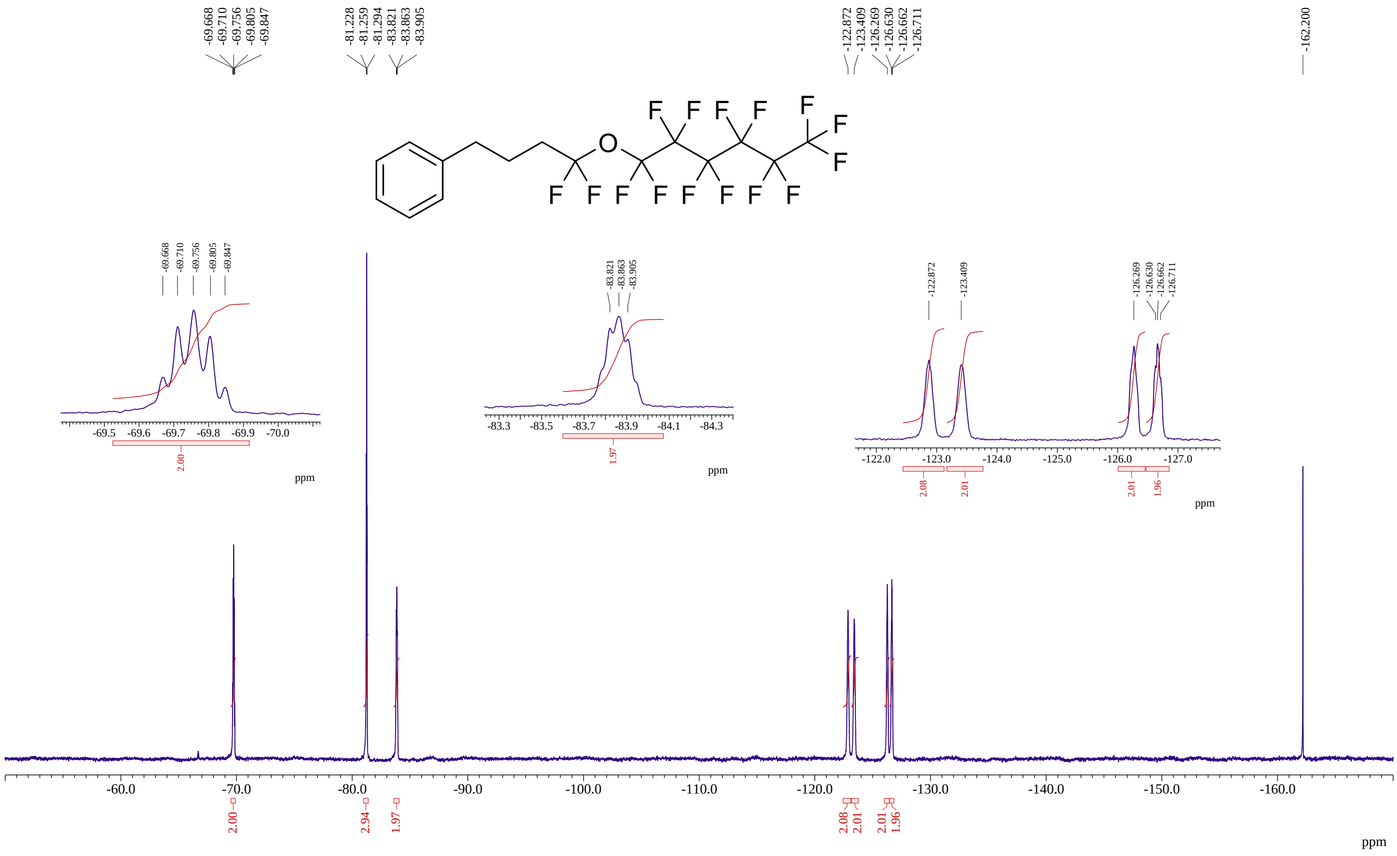

^{1}H NMR (500 MHz, CDCl$_3$): **10**

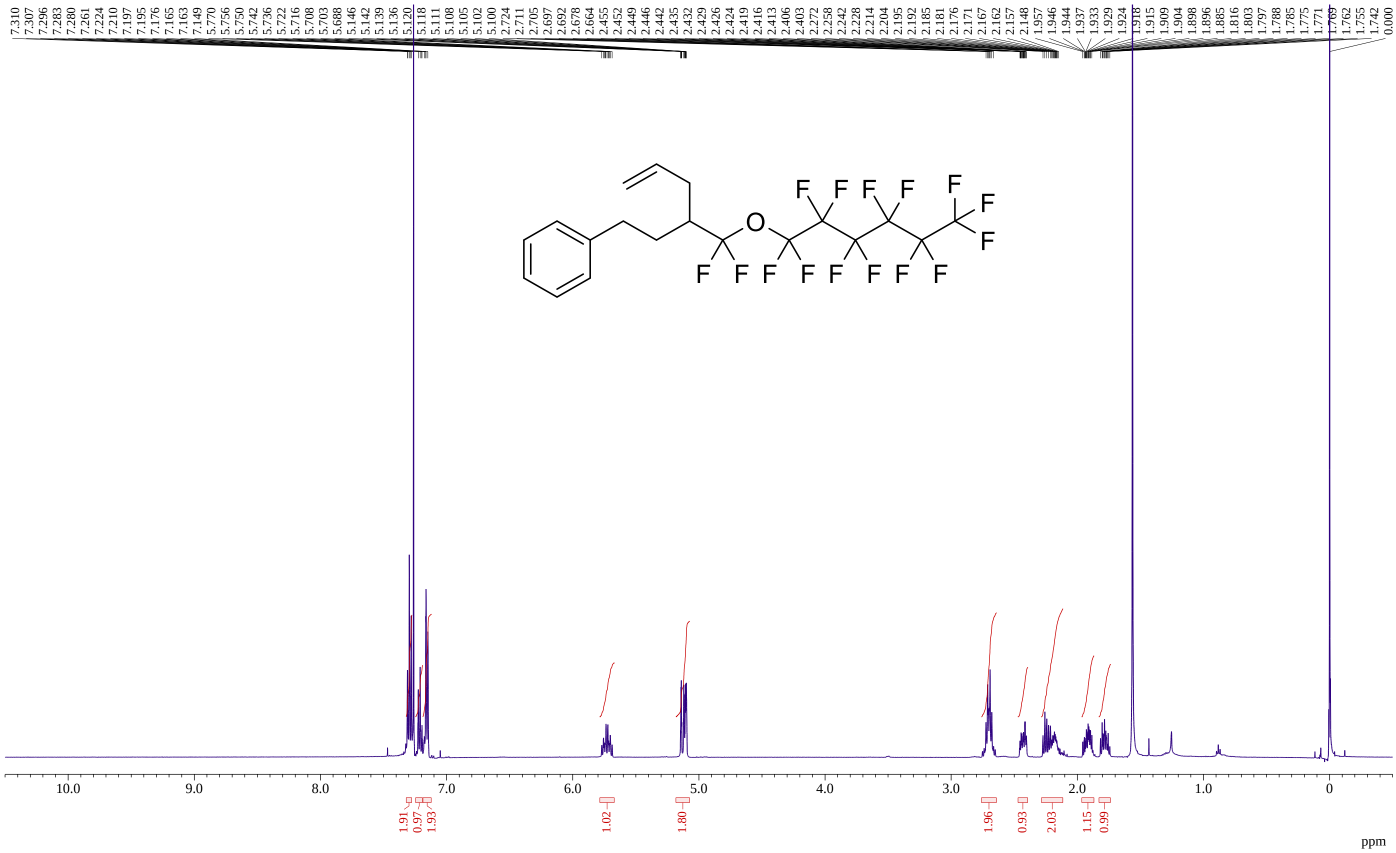

^{13}C NMR (126 MHz, CDCl$_3$): **10**

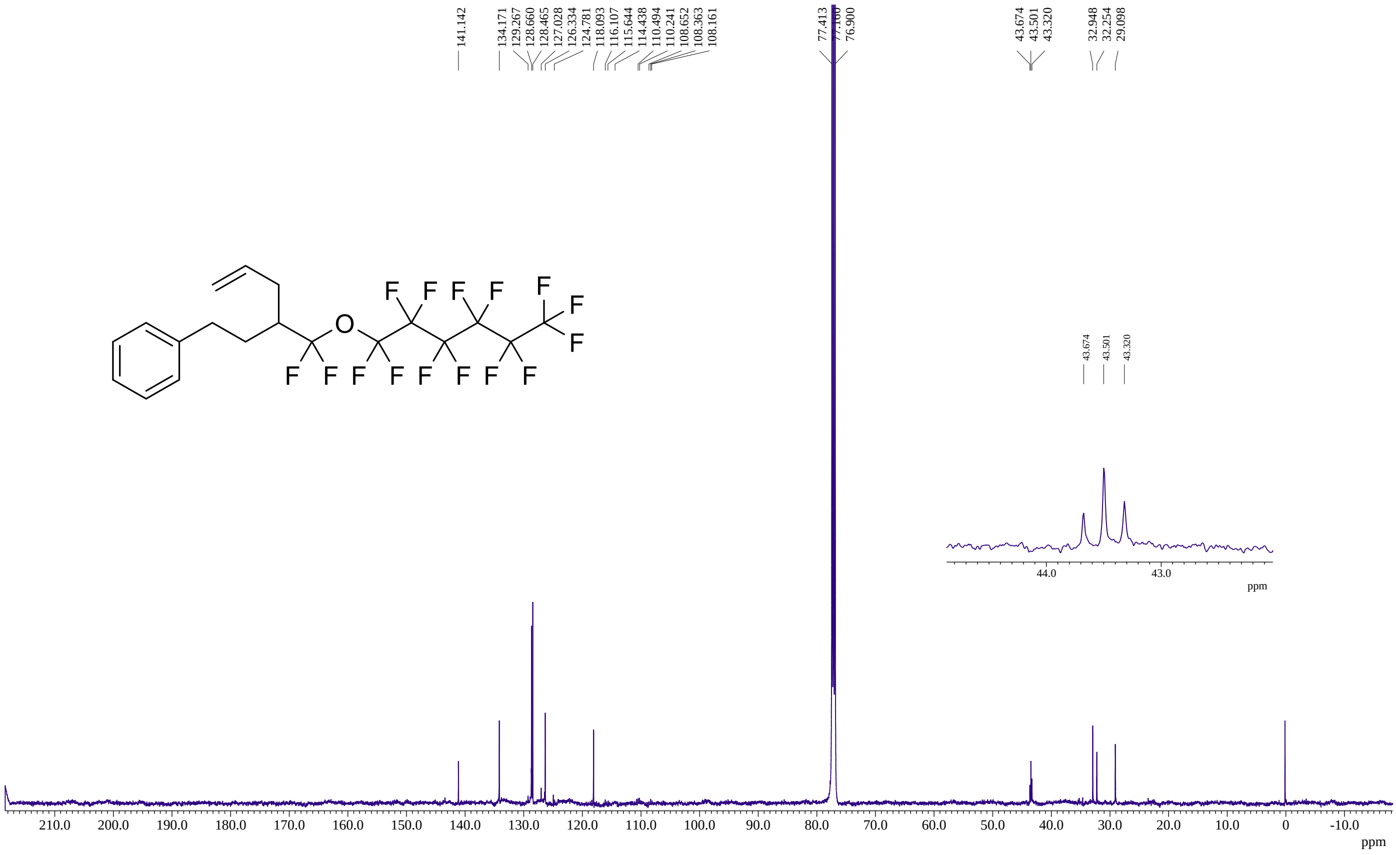

^{19}F NMR (282 MHz, $CDCl_3$): **10**

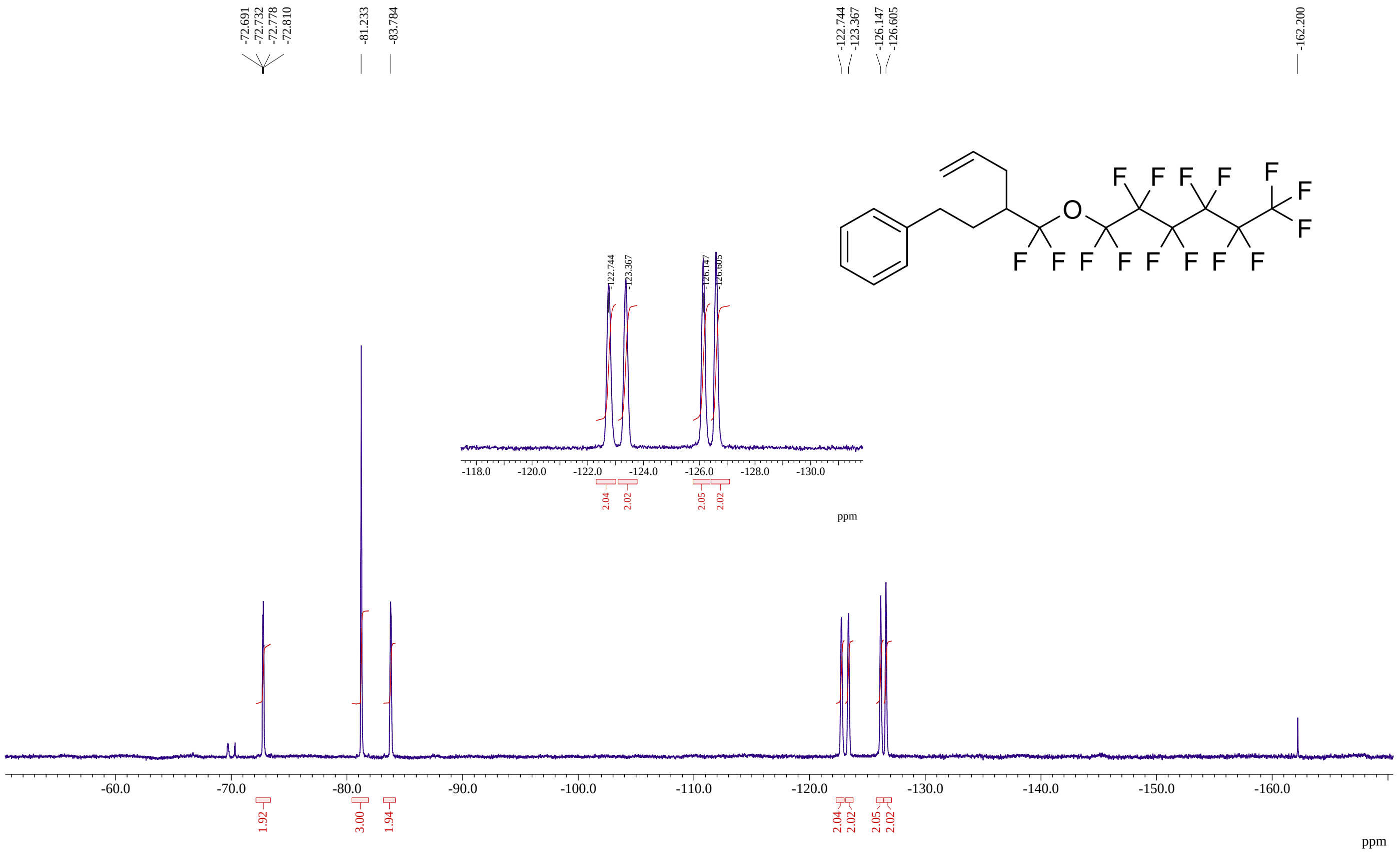

^{1}H NMR (500 MHz, $CDCl_3$): **11**

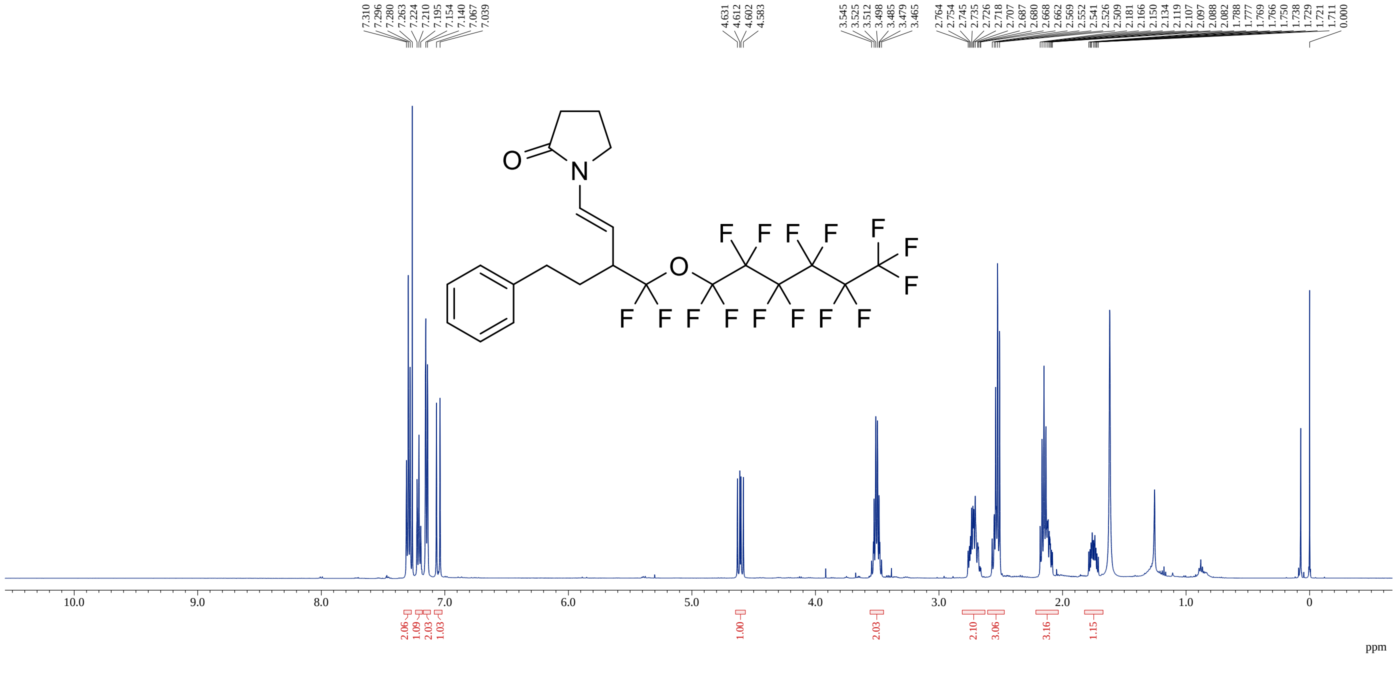

^{13}C NMR (126 MHz, $CDCl_3$): **11**

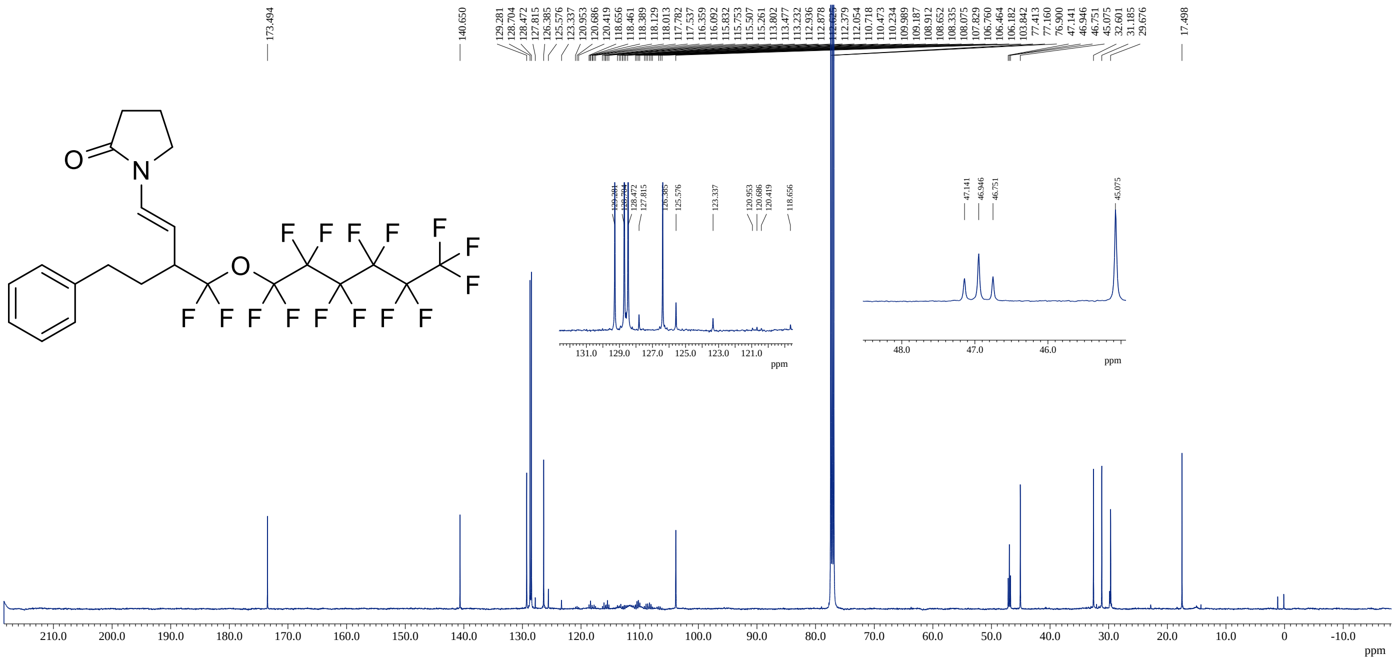

^{19}F NMR (282 MHz, $CDCl_3$): **11**

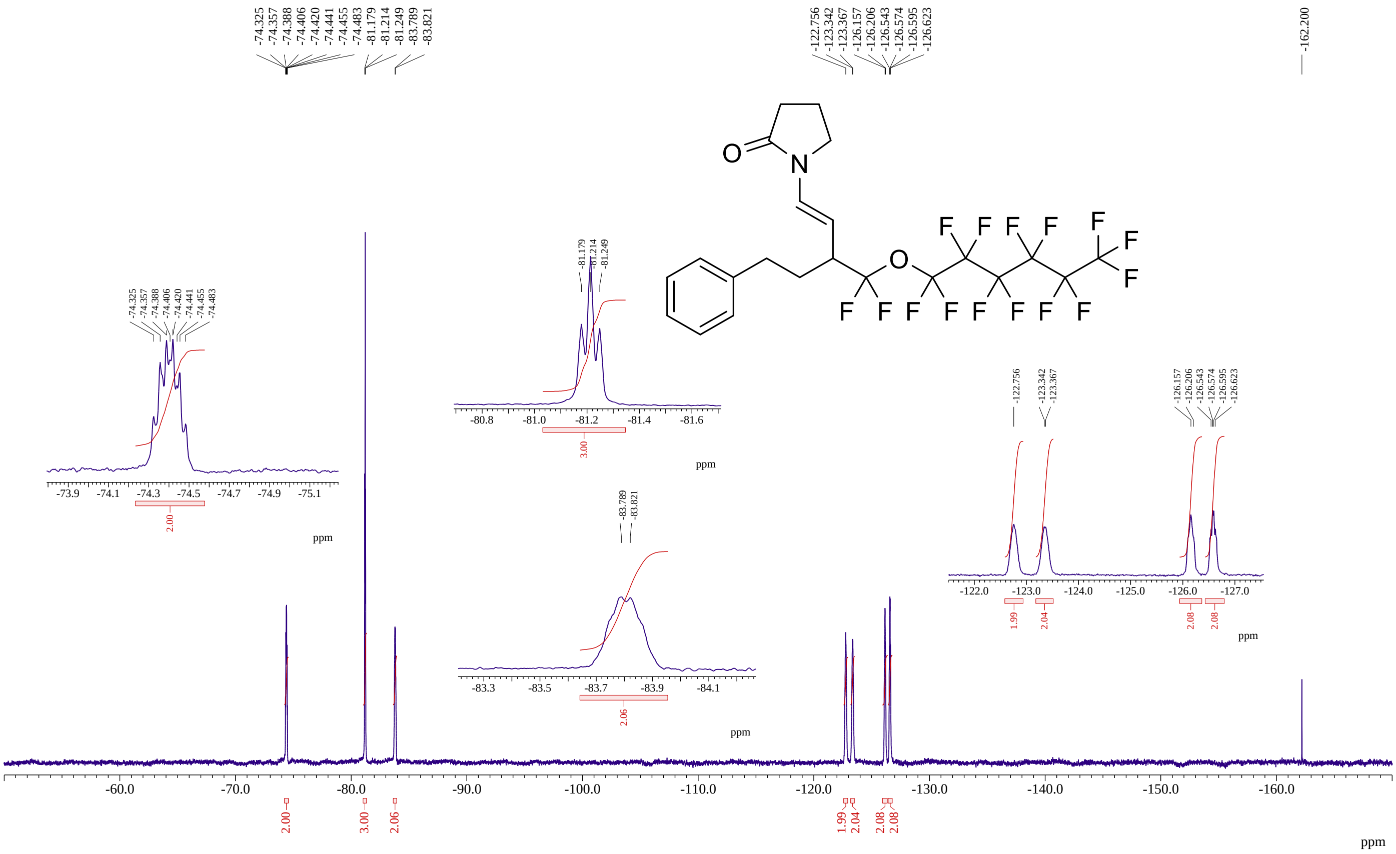

^{1}**H NMR** (500 MHz, $CDCl_3$): **12**

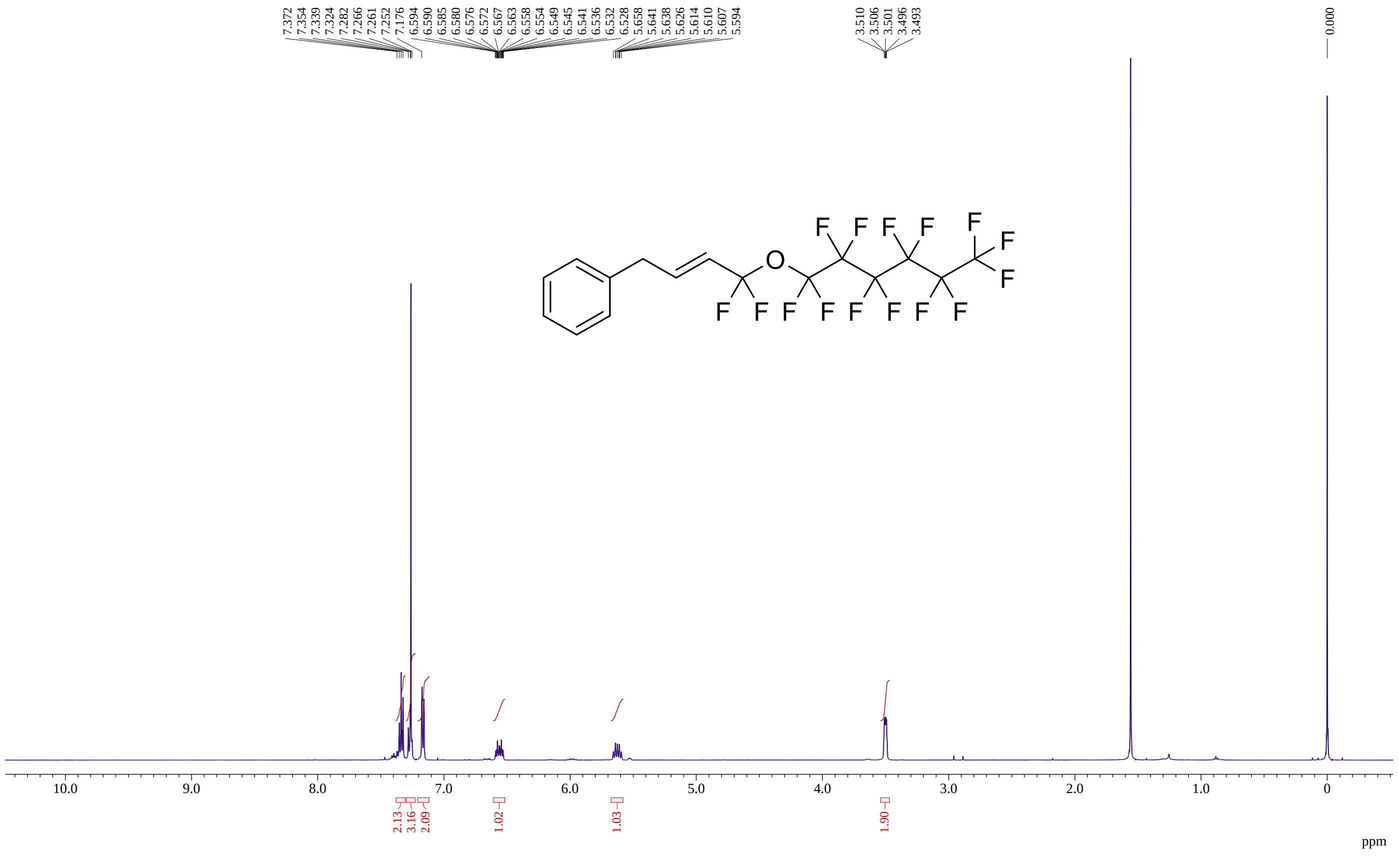

^{13}**C NMR** (126 MHz, $CDCl_3$): **12**

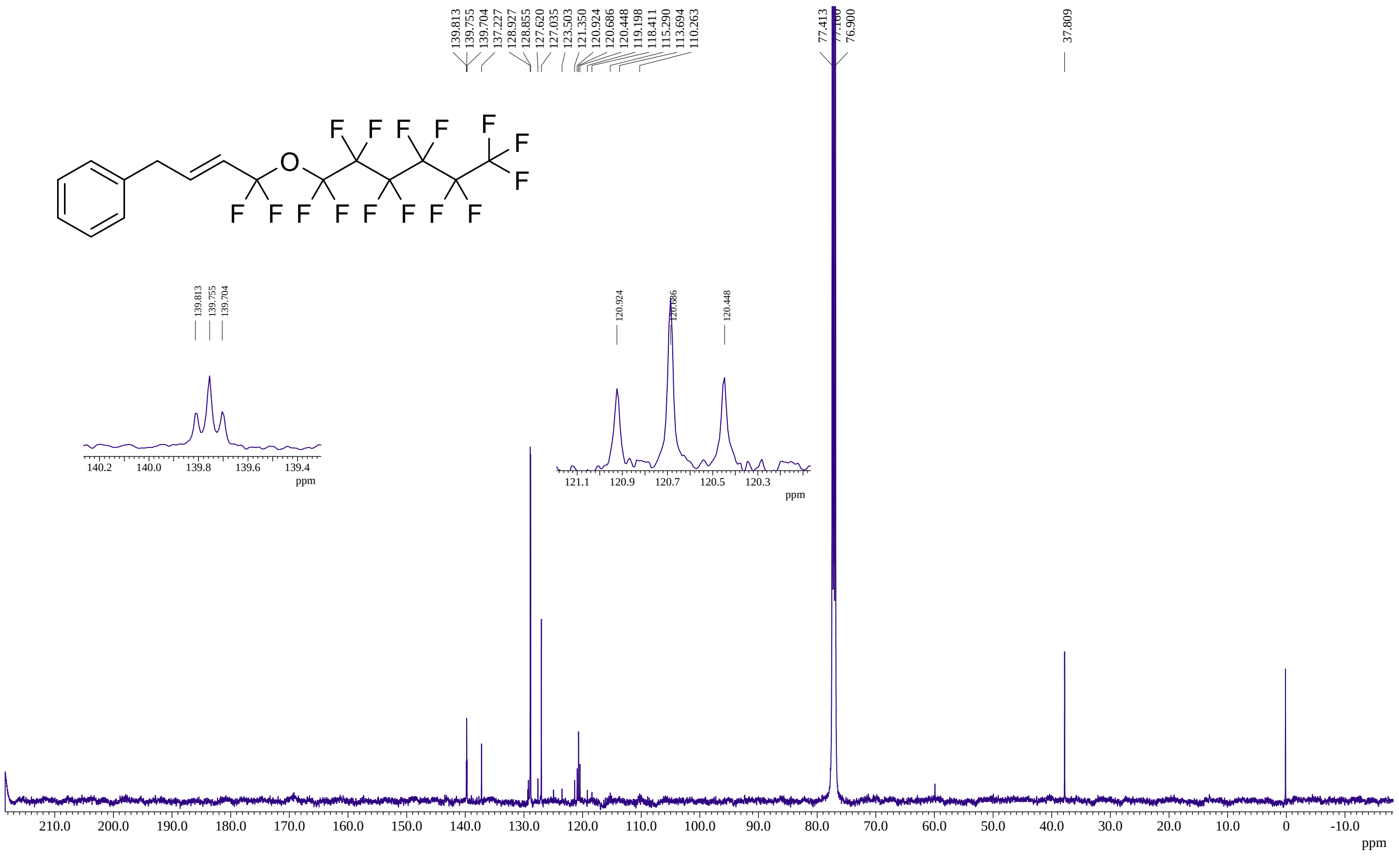

^{19}F NMR (282 MHz, $CDCl_3$): **12**

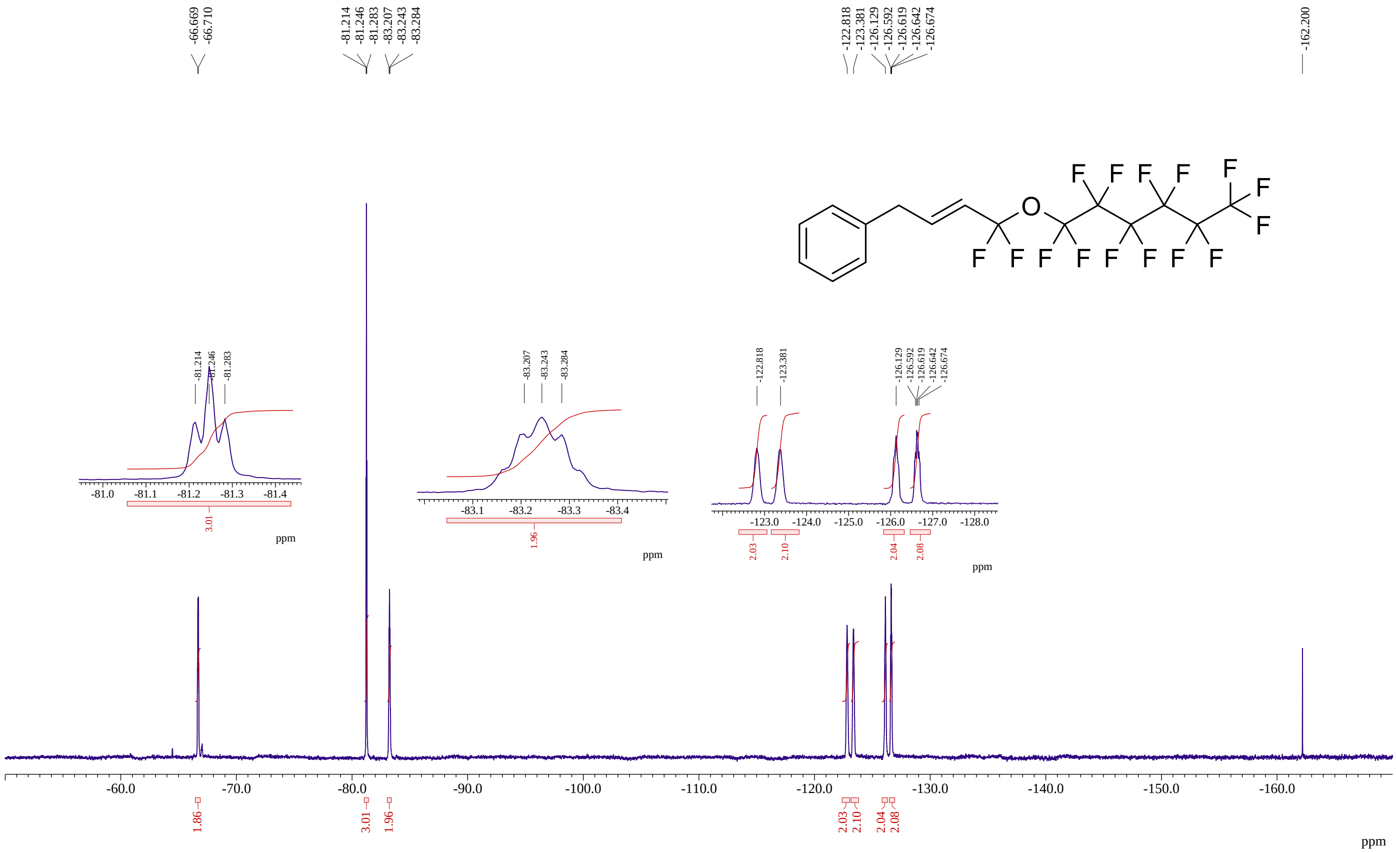

Supplement: SC-015-D4SC02084G-s001 [file SC-015-D4SC02084G-s001.pdf]
